# 4-Oxo-7-Halosubstituted Phenanthroline Diamides Bifunctional Reactivity: A Synthetic Pathway to Unsymmetrical Polydentate Ligands

**DOI:** 10.3390/ijms27177907

**Published:** 2026-09-04

**Authors:** Roman V. Zonov, Nane A. Avagyan, Pavel S. Lemport, Vitaly A. Roznyatovsky, Victor N. Khrustalev, Yulia V. Nelyubina, Yuri A. Ustynyuk, Valentine G. Nenajdenko

**Affiliations:** 1 Department of Chemistry, Lomonosov Moscow State University, Leninskie Gory 1 Bld. 3, 119991 Moscow, Russia; roman.zonoff@yandex.ru (R.V.Z.); nane.avakyan@mail.ru (N.A.A.); lemport.pavel@yandex.ru (P.S.L.); vit.rozn@nmr.chem.msu.ru (V.A.R.); yuriustynyuk@gmail.com (Y.A.U.); 2 Department of General and Inorganic Chemistry, Peoples’ Friendship University of Russia (RUDN University), Miklukho-Maklay Str. 6, 117198 Moscow, Russia; vnkhrustalev@gmail.com; 3 Zelinsky Institute of Organic Chemistry, Russian Academy of Sciences, Leninsky Prospect 47, 119991 Moscow, Russia; 4 Advanced Engineering School, ITMO University, 9 Lomonosova Str., 191002 St. Petersburg, Russia; yulia.v.nelyubina@gmail.com

**Keywords:** nucleophilic substitution, fluorine, phenanthroline, ligand, DFT, X-ray, NMR

## Abstract

An efficient approach to unsymmetrical 1,10-phenanthroline-2,9-dicarboxamides based on the bifunctional reactivity of 4-oxo-7-haloderivatives is reported. These substrates exhibit orthogonal reactivity, enabling selective nucleophilic aromatic substitution at the C^7^ position alongside electrophilic functionalization of the 4-oxo group under mild conditions. Reactions with a broad range of C-, N-, and O-nucleophiles afford 4-oxo-7-functionalized products in high yields, while subsequent transformations of azido- and hydroxy-substituted derivatives further demonstrate the synthetic versatility of this platform. The observed chemoselectivity is reflected in the highly selective O-functionalization of the 4-oxogroup, with alkylation proceeding under mild conditions. The developed strategy provides a general route to structurally diverse unsymmetrical derivatives and highlights the potential of 4-oxo-7-halo-systems as bifunctional building blocks.

## 1. Introduction

The nucleophilic aromatic substitution reaction is one of the most convenient methods for the functionalization of heteroaromatic compounds. Fluorinated aromatics are widely used for this aim because in this case S_N_Ar reactions proceed under milder conditions than in the case of their chlorosubstituted analogs [1]. From this point of view, a number of strategies and reagents for the implementation of nucleophilic fluorination have been developed [2,3,4,5,6,7].

1,10-Phenanthroline derivatives constitute an important class of π-deficient heterocycles widely used in coordination chemistry, photonics, and materials science [8,9,10,11]. The introduction of additional coordinating centers into the phenanthroline framework, such as amide oxygen atoms, affords 1,10-phenanthroline-2,9-dicarboxamides (DAPhen), which form stable complexes with *f*-block elements [12,13,14,15]. These ligands are therefore considered promising components of extraction systems for the separation of lanthanides and actinides from spent nuclear fuel [16,17,18]. In addition, structural modification of the phenanthroline core enables tuning of both the electronic properties of the ligands and the photophysical behavior of their metal complexes [19,20,21,22,23].

It is worth mentioning some examples of unsymmetrically substituted DAPhen, which may have interesting extraction properties due to the presence of two different groups of amide substituents [24,25]. However, their synthesis sometimes gives rather low yields. Regarding photophysical properties, unsymmetric 4-oxo-7-fluoro-DAPhen showed the highest total quantum yield for the europium nitrate complex compared to other unsubstituted and 4,7-symmetrically substituted ligands [21]. Thus, the synthesis of unsymmetrically substituted ligands of this class is a promising task.

Previously, we used 4,7-difluorosubstituted DAPhen as starting compounds for the synthesis of symmetric and unsymmetric ligands of various structures via the S_N_Ar reaction [26]. At the same time, the hydrolytic lability of the C–F bond was exploited to efficient synthesize 4-oxo-7-fluoro-DAPhen [27]. Further study of the hydrolysis reaction of the C-Hal bond in 4,7-dichloro- and 4,7-difluoro-DAPhen also allowed us to obtain their 4-oxo-7-chloro- and 4-oxo-7-hydroxy-derivatives [28].

The obtained 4-oxo derivatives exist in oxo/hydroxy tautomeric equilibrium similar to that of pyridines [29,30,31]. Such tautomerism strongly influences the chemical and coordination properties of these compounds by affecting the basicity and nucleophilicity of the heterocyclic nitrogen atom [32,33,34,35,36]. Consequently, selective N- and O-functionalization remains challenging [37,38,39,40]. Existing methods for the synthesis of 4,7-alkoxy-substituted DAPhen from 4,7-oxosubstituted precursors often require harsh conditions or additional synthetic steps [41,42].

Herein, we demonstrate that 4-oxo-7-halosubstituted DAPhen serve as bifunctional synthetic platforms enabling orthogonal transformations of the phenanthroline core. We investigated S_N_Ar reactions of 4-oxo-7-fluoro-DAPhen derivatives with C-, N-, and O-nucleophiles and demonstrated selective electrophilic functionalization of the 4-oxogroup under mild conditions. The developed approach provides efficient access to structurally diverse unsymmetrical DAPhen ligands and reveals the unique chemoselective behavior of these heterocyclic systems.

## 2. Results and Discussions

**Figure 1:**
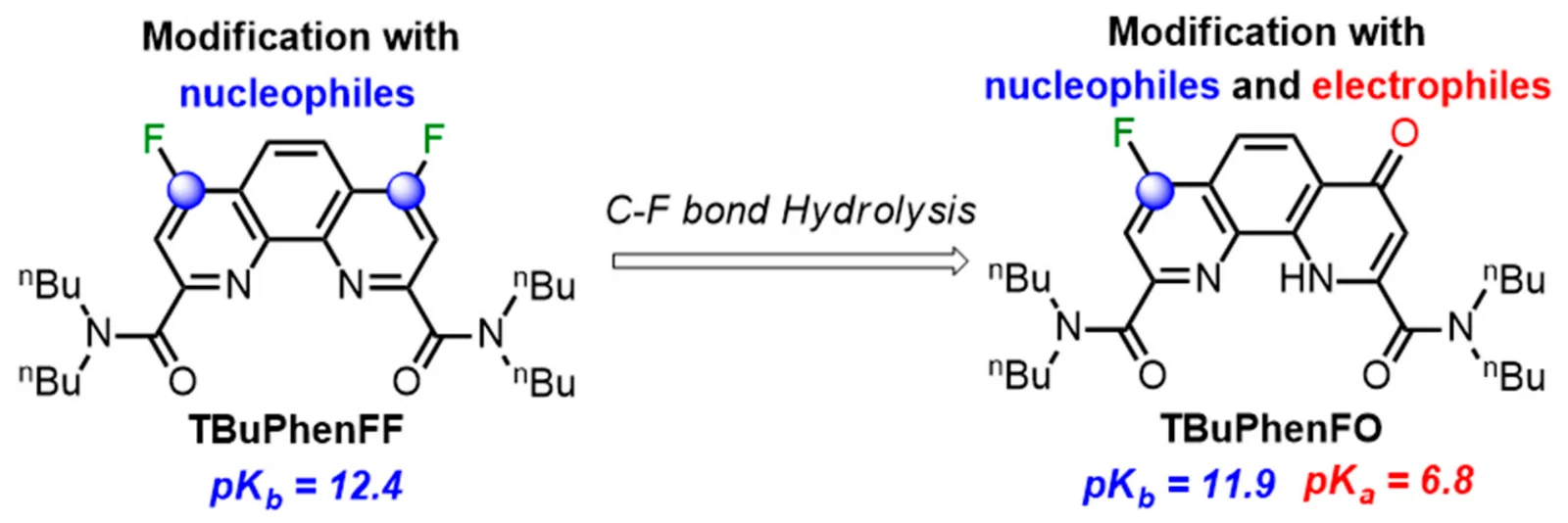
Comparison of 4,7-difluoro- and 4-oxo-7-fluoro-DAPhen.

In our previous work [26], we described an approach to the 4,7-functionalization of phenanthroline diamides using a nucleophilic aromatic substitution reaction of the fluorine atom. It is evident that the 4-oxo-7-fluorosubstituted DAPhen considered in this work differ from these compounds in their physicochemical properties. Hydrolysis by the C-F bond also leads to the appearance of the N-H center of acidity and changes in the characteristics of pKb and pKa (see the Experimental Section for the calculation method). We assumed that changing these parameters also allows modification of the ligand structure by using reactions with both nucleophiles and electrophiles (Figure 1).

### 2.1. Modification of 4-Oxo-7-Halo-DAPhen with Nucleophiles

**Scheme 1:**
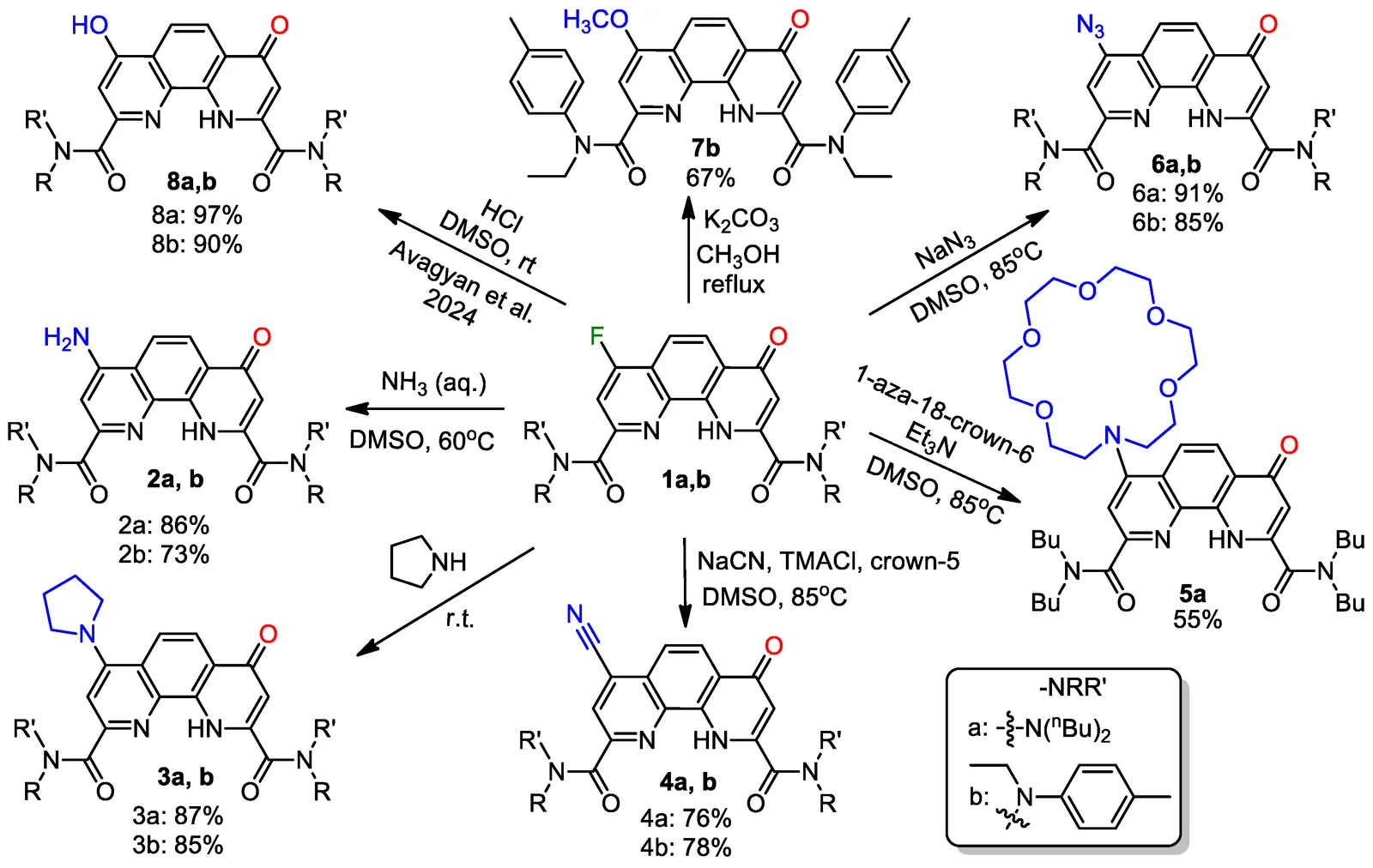
Reactions of 4-oxo-7-fluorosubstituted DAPhen’s 1 with N-, O- and C-nucleophiles [28].

We set out to synthesize unsymmetrical 4-oxo-7-substituted phenanthroline diamides using S_N_Ar reaction of fluorine in diamides **1**. Thus, diamides **1** were involved in reactions with C-, O-, and N-nucleophiles of various types (Scheme 1). Due to the presence of fluorine in the starting diamides, the reaction progress can be monitored easily using ^19^F NMR spectroscopy. All these transformations can be performed efficiently under mild conditions. The reaction of **1** with pyrrolidine proceeds at room temperature using pyrrolidine as both the reagent and the solvent. Reaction with 1-aza-18-crown-6 ether was conducted in the presence of Et_3_N as an HF acceptor. Reaction of **1** with NaCN and NaN_3_ proceeds in DMSO at 85 °C. It is interesting to note that by carrying out these transformations, it is possible to introduce either donor or acceptor functional groups into the phenanthroline core, which is valuable when adjusting the ligand’s properties.

**Figure 2:**
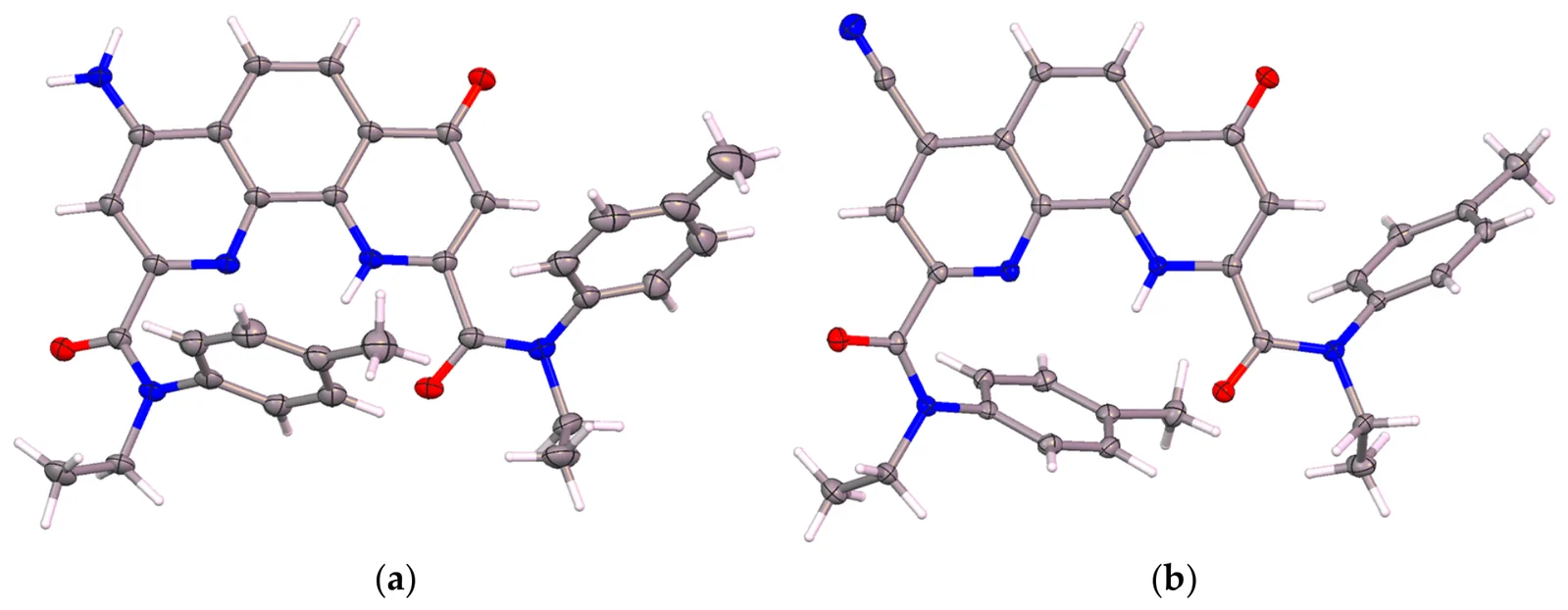
X-ray structures of (**a**) **2b** and (**b**) **4b**.

The structures of compounds **2b** and **4b** were also confirmed by the single-crystal X-ray diffraction method. The molecular structures of the compounds, as determined by the single-crystal X-ray diffraction method, are shown in Figure 2. It is interesting to note that in the crystal lattice **2b**, the amino groups are “surrounded” by other ligand molecules via hydrogen bonds.

**Scheme 2:**
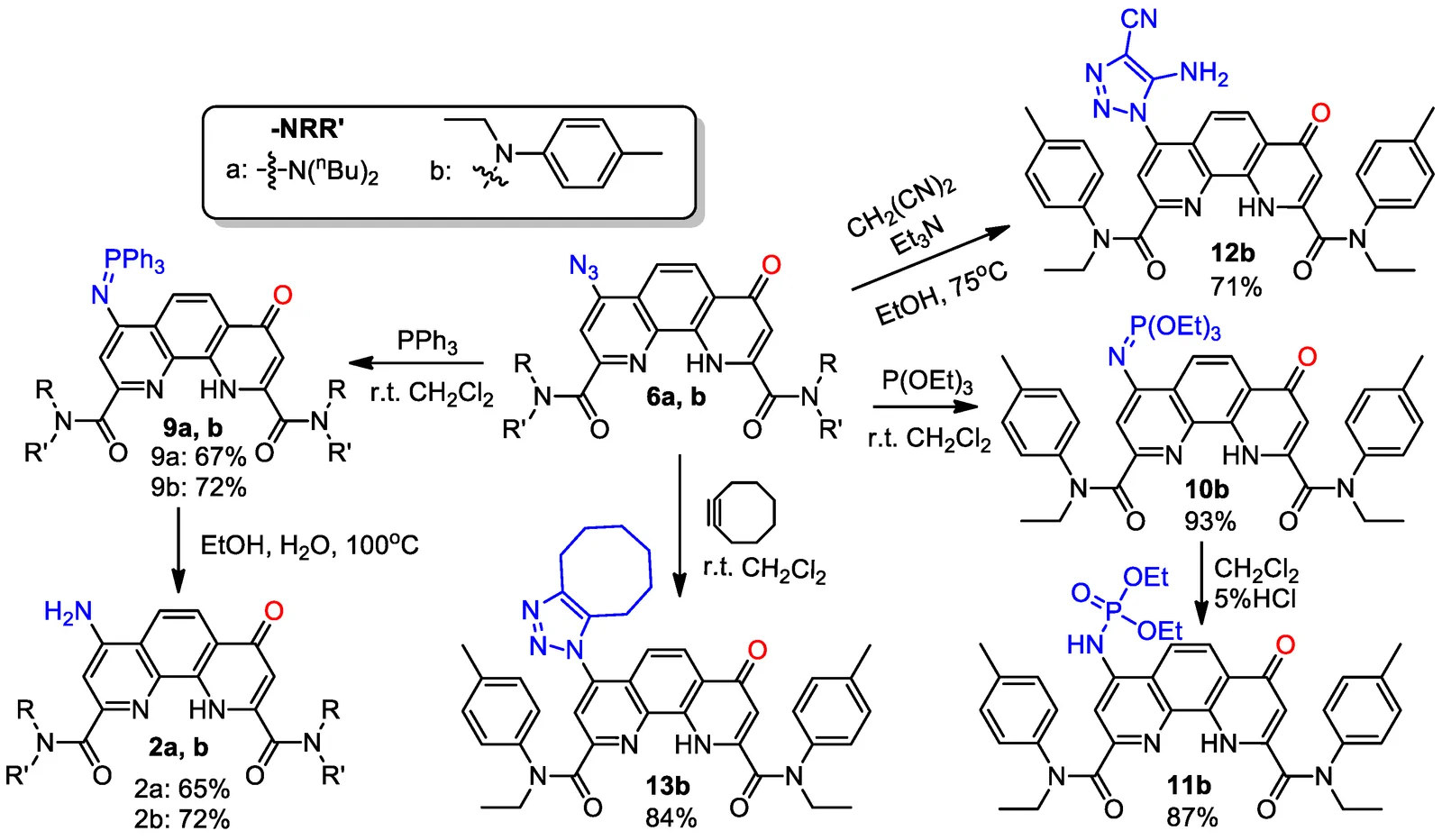
Transformations of azides **6**.

The presence of azide group in compounds **6** allows for their further modification [43,44,45]. Thus, the reaction of azides **6a**,**b** with phosphorus nucleophiles yielded the corresponding products **9** and **10**, which are able to undergo further transformations, including hydrolysis and hydration to **2** and **11** respectively. In addition, azide **6b** was subjected to SPAAC reaction to form triazole **13**, as well as to a reaction with malononitrile in the presence of a base to form functionalized triazole **12** (Scheme 2). None of the reactions described affect the 4-oxo group and can serve as useful instrument for the preparation of functionalized DAPhen derivatives containing a 4-oxo group.

**Scheme 3:**
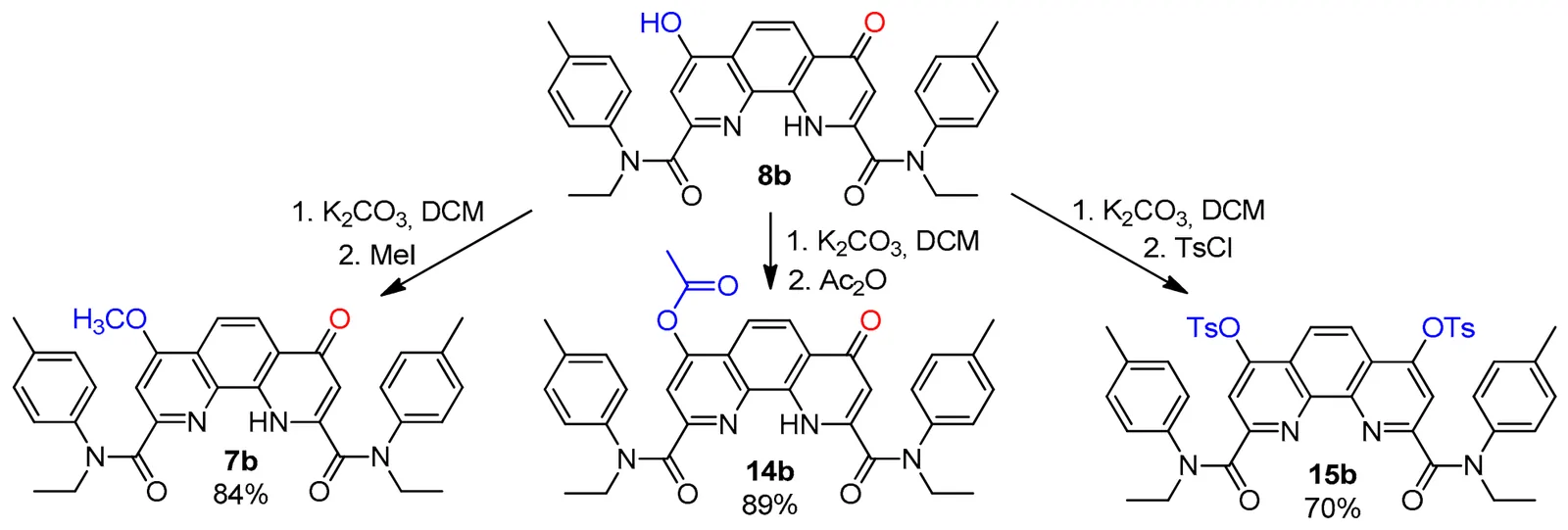
Further transformations of diamides **8b**.

The synthetic utility of the obtained compounds was further demonstrated using compound **8b** as a representative example (Scheme 3). Treatment of **8b** with appropriate electrophiles afforded the corresponding O-alkylated, O-acylated, and O-tosylated derivatives. Notably, deprotonation of **8b** generates an ambident anion that, in principle, could react either by the oxygen atom of the 4-oxo group or through the heterocyclic nitrogen atom. However, functionalization in all cases occurred exclusively at the oxygen center.

It is important to note that for all of the compounds **2**–**14**, the 4-oxo-form is the most stable tautomeric form, which is also confirmed by NMR spectroscopy data. The obtained 4-oxo-7-substituted diamides are characterized by the presence of a characteristic broad signal in the downfield region of 10–11 ppm, corresponding to the NH protons of the oxo form, and, more significantly, by a characteristic signal in the 5.5–6.5 ppm, corresponding to the H^3^ proton of the phenanthroline nucleus. In the ^13^C NMR spectrum, a characteristic signal in the ~175 ppm region, corresponding to the C^4^=O carbon atom, is also present for this class of compounds (see ESI, Figures S1–S65).

**Scheme 4:**
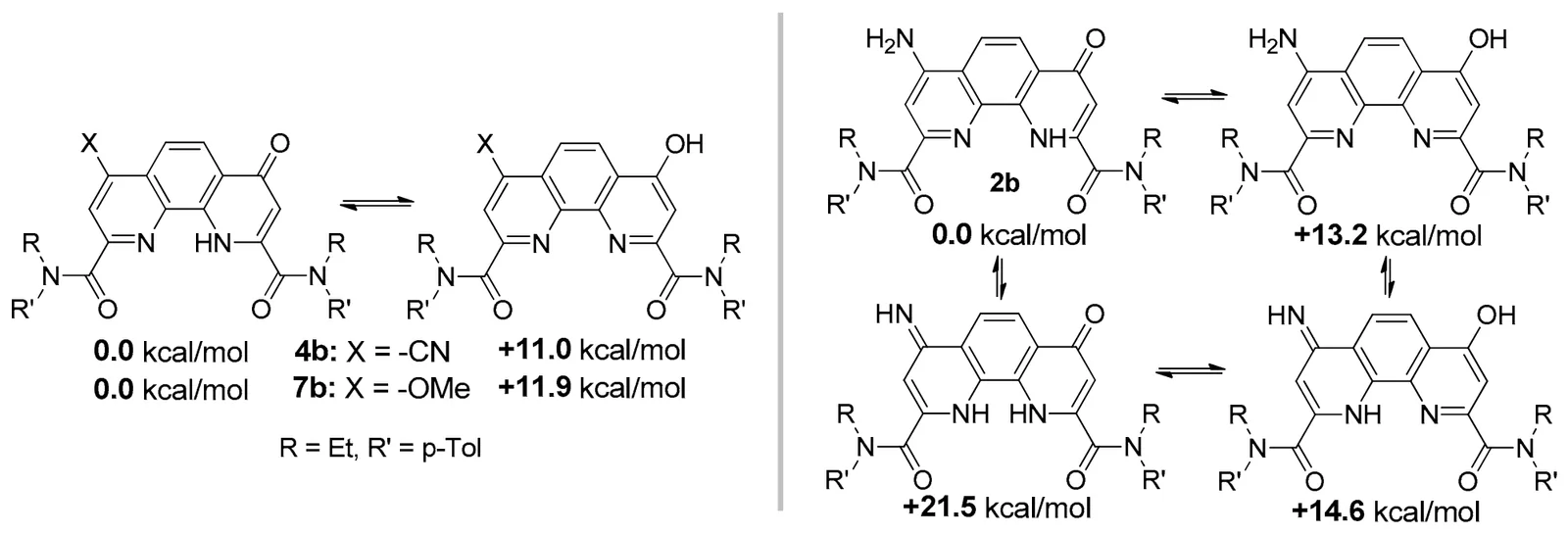
Relative energies of the **2b**, **4b** and **7b** tautomers.

To evaluate the influence of the electronic nature of the substituent at the 7-position, the tautomeric equilibria of the cyano- and methoxy-substituted derivatives **4b** and **7b** were examined (Scheme 4). DFT calculations performed at the B3LYP/6-31G(d,p) level revealed that the oxo-tautomer remains energetically preferred in both cases. However, the calculated energy difference between the oxo- and hydroxy-forms is reduced for the cyano-substituted derivative, indicating a significant substituent effect on the tautomeric equilibrium. This trend is consistent with the electron-withdrawing nature of the cyano-group, which decreases the basicity of the heterocyclic nitrogen atom and consequently stabilizes the hydroxy-tautomer relative to the oxo-form. Particular attention was paid to the 4-oxo-7-amino-substituted diamides **2**, for which both oxo-hydroxy and amino–imino tautomeric equilibria are possible. As a result, four tautomeric forms may be considered for these unsymmetrical derivatives (Scheme 4). Despite the increased number of accessible tautomeric structures, calculations indicate a clear preference for the oxo-amino form which is consistent with experimental X-ray (see Figure 2a) and NMR data (see ESI, Figures S4 and S5).

**Figure 3:**
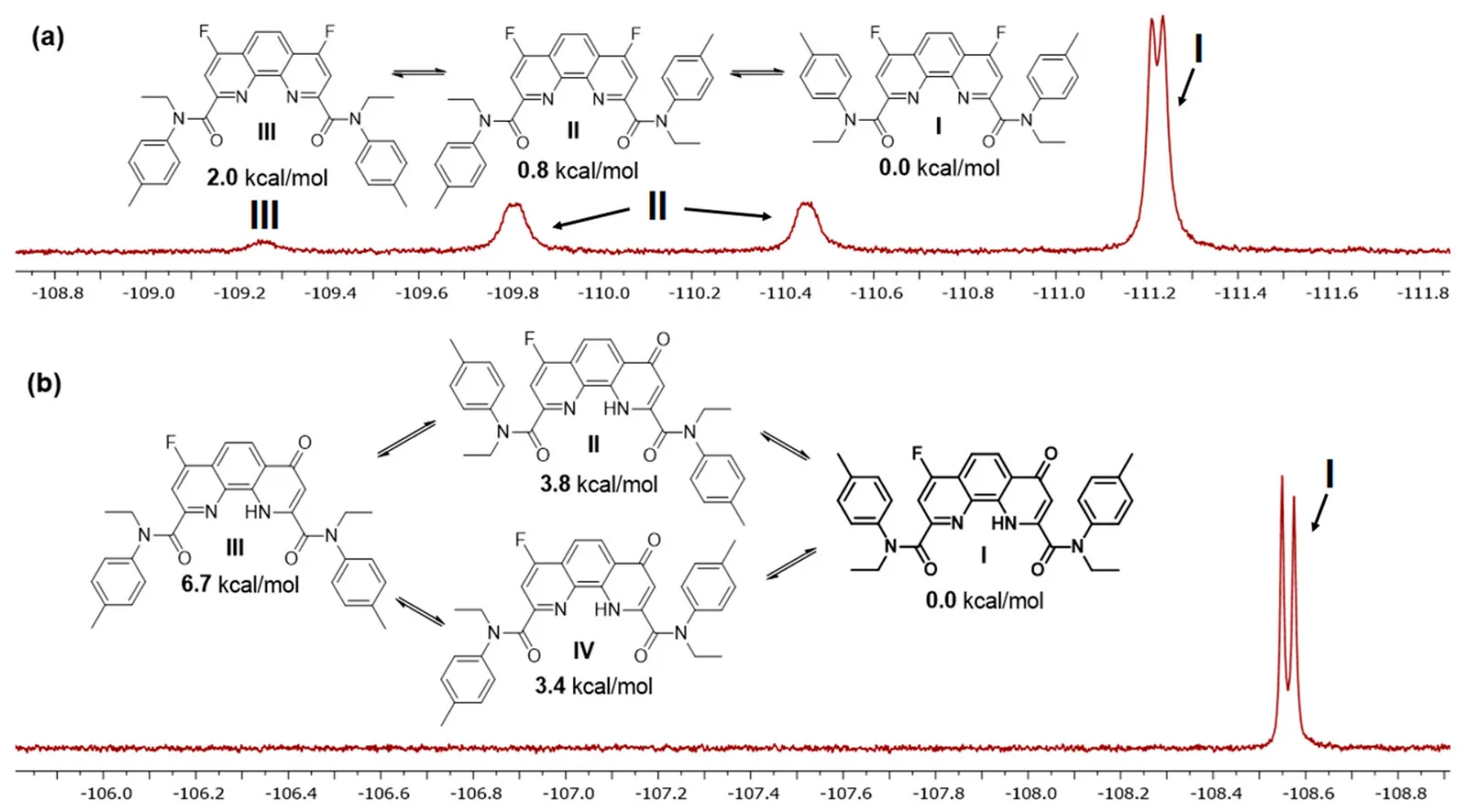
Comparison of ^19^F NMR spectra of (**a**) 4,7-difluoro- and (**b**) 4-oxo-7-fluoro-DAPhen **1b**.

The considered tautomeric equilibria can significantly affect the conformational behavior of the ligands being studied. For DAPhen-type ligands, hindered rotation around the amide bonds typically leads to the formation of three conformers: major symmetric conformer (0.0 kcal/mol relative energy, conformer I), minor symmetric conformer III, and unsymmetric conformer II (Figure 3a).

In the case of 4-oxo-substituted ligands, the presence of an NH proton in the phenanthroline core can significantly influence the relative energies of these conformations due to steric and electronic effects (Figure 3b). The indicated equilibria can be seen in the emerging features of the ^19^F NMR spectra of these compounds [46,47,48,49]. Using unsymmetric 4-oxo-7-fluorosubstituted ligand **1b** and its 4,7-difluorosubstituted analog as examples, we performed a computational conformational analysis. The corresponding barriers to rotation for potentially hindered bonds, the amide bond and the C_Phen_–C=O bond, as well as the relative energies for each of the three possible conformers associated with rotation around the amide bond, were calculated (see ESI, Table S2).

The 4-oxosubstituted ligands are distinguished by a significantly larger energy difference between the accessible conformers. This effect primarily arises from the steric influence of the NH group within the phenanthroline core, which destabilizes specific conformational arrangements and shifts the equilibrium toward a single preferred conformer. As a consequence, conformational exchange has a much smaller impact on the NMR spectra, resulting in substantially reduced signal-broadening compared to analogous ligands lacking the 4-oxogroup. This conclusion is in good agreement with the experimentally observed NMR spectra of the 4-oxo-substituted derivatives. It is worth noting that the relative energies of the conformers of compounds under consideration are also significantly lower than the relative energies of various tautomeric forms. The Cartesian coordinates and energetic parameters of optimized structure are provided in ESI (pp. S84–S134).

### 2.2. Modification of 4-Oxo-7-Halo-DAPhen with Electrophiles

**Figure 4:**
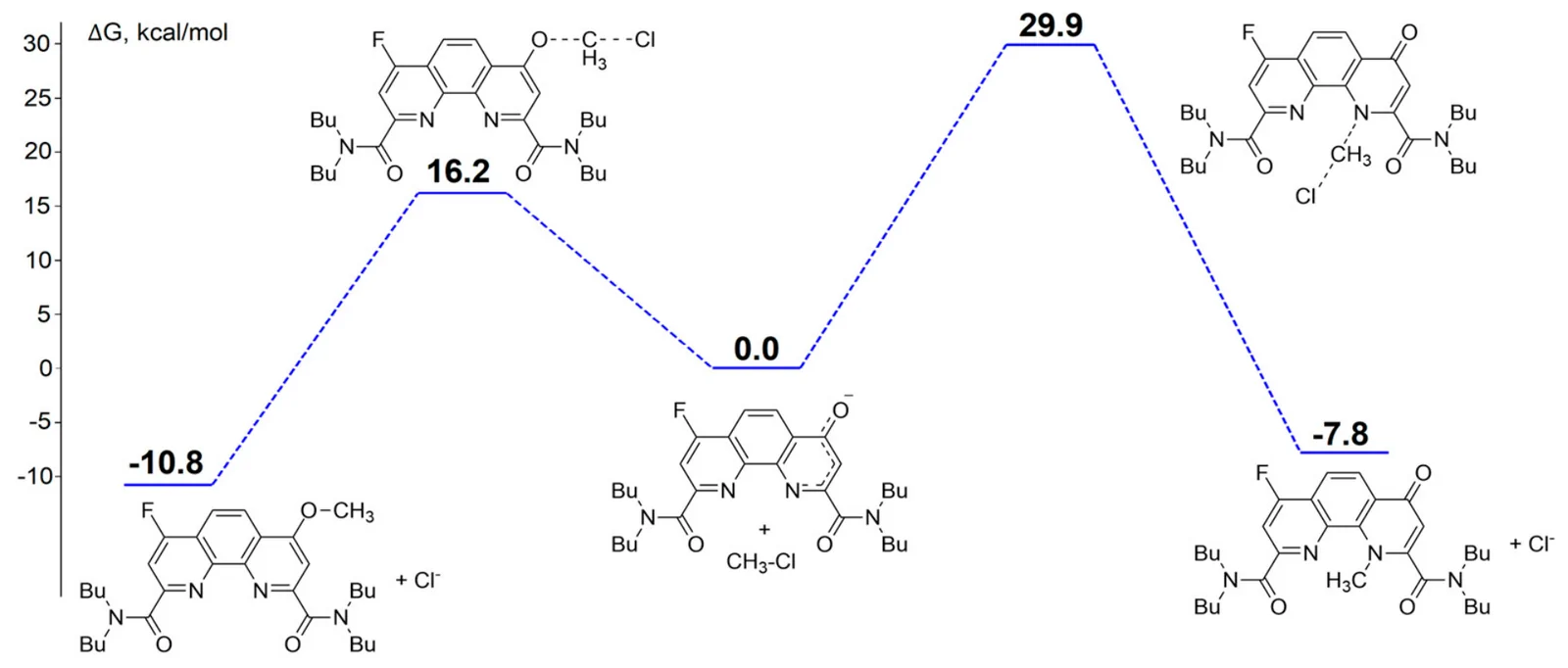
Energy profile alkylation reaction of anion **1a**.

The pronounced calculated NH-acidity of the 4-oxosubstituted ligands under consideration led us to consider the modification reactions of this group. We performed quantum-chemical modeling of the alkylation reaction of compound **1a** to study the reaction mechanism involving methyl chloride as an electrophilic agent, given the simplicity of modeling this reaction (B3LYP-D3BJ/6-31G(d,p), CPCM(CHCl_3_) theoretical level) (Figure 4). Steric factors and energy characteristics of the conformation through which the reaction proceeds play a significant role in the energy difference between the transition states of O- and N-alkylation. From the presented profile, it can be seen that O-alkylation is characterized by a lower activation barrier and is also thermodynamically favorable.

The presented calculations can also be compared with similar calculations for 4-oxo-pyridones which fragment can be found in the structure of **1a** [50]. Unlike pyridones, the energy profile of N- and O-alkylation of 4-oxo-DAPhen is characterized by a greater energy difference between the transition states of N- and O-alkylation (∆∆G^≠^ = 13.7 kcal/mol in presented energetic profile). In addition, the thermodynamic product of alkylation of 4-oxo-pyridones is the product of N-alkylation, while in the case of oxo-phenanthrolinediamides, the product of O-alkylation is both a thermodynamic and kinetic reaction product (∆∆G = 3.0 kcal/mol). Thus, 4-oxo-substituted phenanthrolinediamides can be universal starting compounds for selective functionalization by oxygen atoms.

**Scheme 5:**
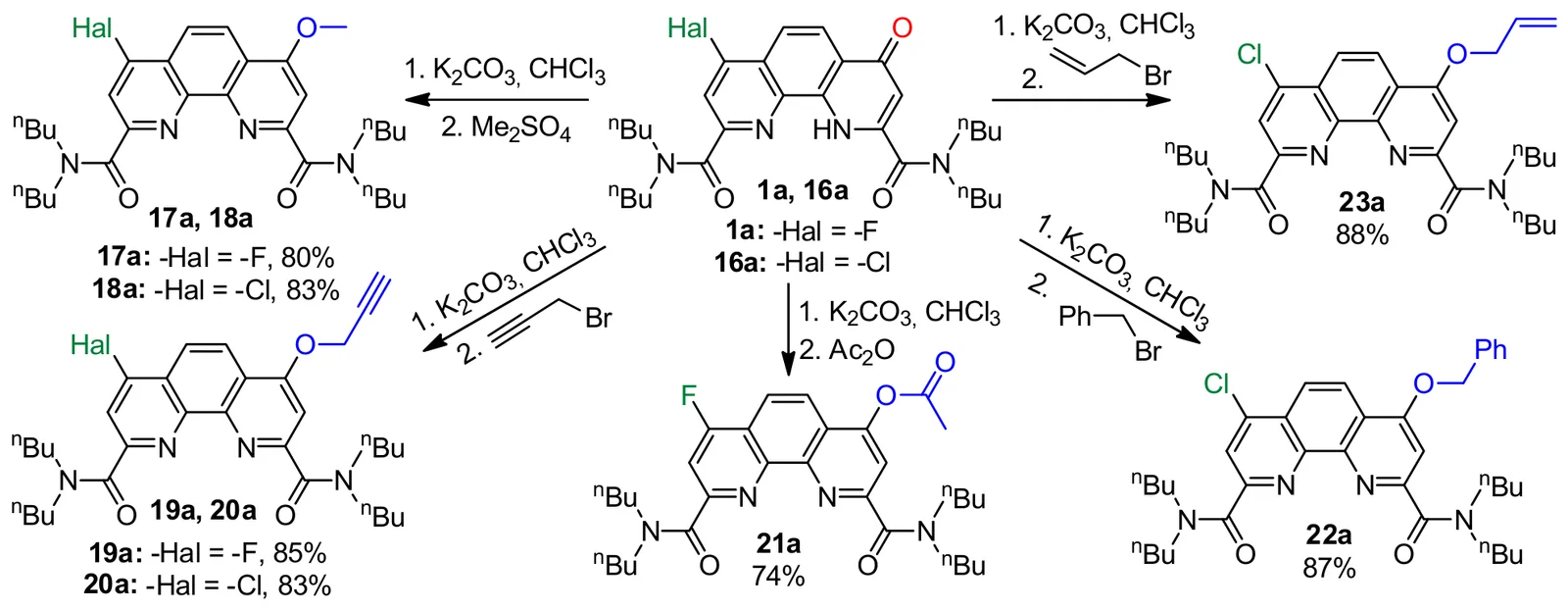
Alkylation and acylation of **1a** and **16a**.

The feasibility of such a reaction via the oxo group was also demonstrated for ligand **1a** to give compounds **17a**, **19a** and **21a** in high yields under mild reaction conditions using potassium carbonate as a base (Scheme 5), offering an alternative to 4,7-functionalization of **1a** via stepwise substitution of the fluorine [26]. To demonstrate the generality of the alkylation method for the 4-oxo-group, we also carried out similar transformations on 4-oxo-7-chloro-phenanthrolinediamide **16a**. Chlorosubstituted phenanthroline diamides are of particular interest as versatile synthetic precursors to 4,7-dicyano-DAPhen ligands, a class of extractants known for their high selectivity in Am/Cm separation [22]. Despite their potential synthetic utility, unsymmetrical chlorosubstituted DAPhen derivatives have not been reported to date. In this context, 4-oxo-7-chloro-phenanthroline diamide **16a** provides a unique entry to such architectures and was employed as a key intermediate for the synthesis of the unsymmetrical ligands **18a**, **20a**, **22a** and **23a** (see ESI, Figures S66–S96).

**Figure 5:**
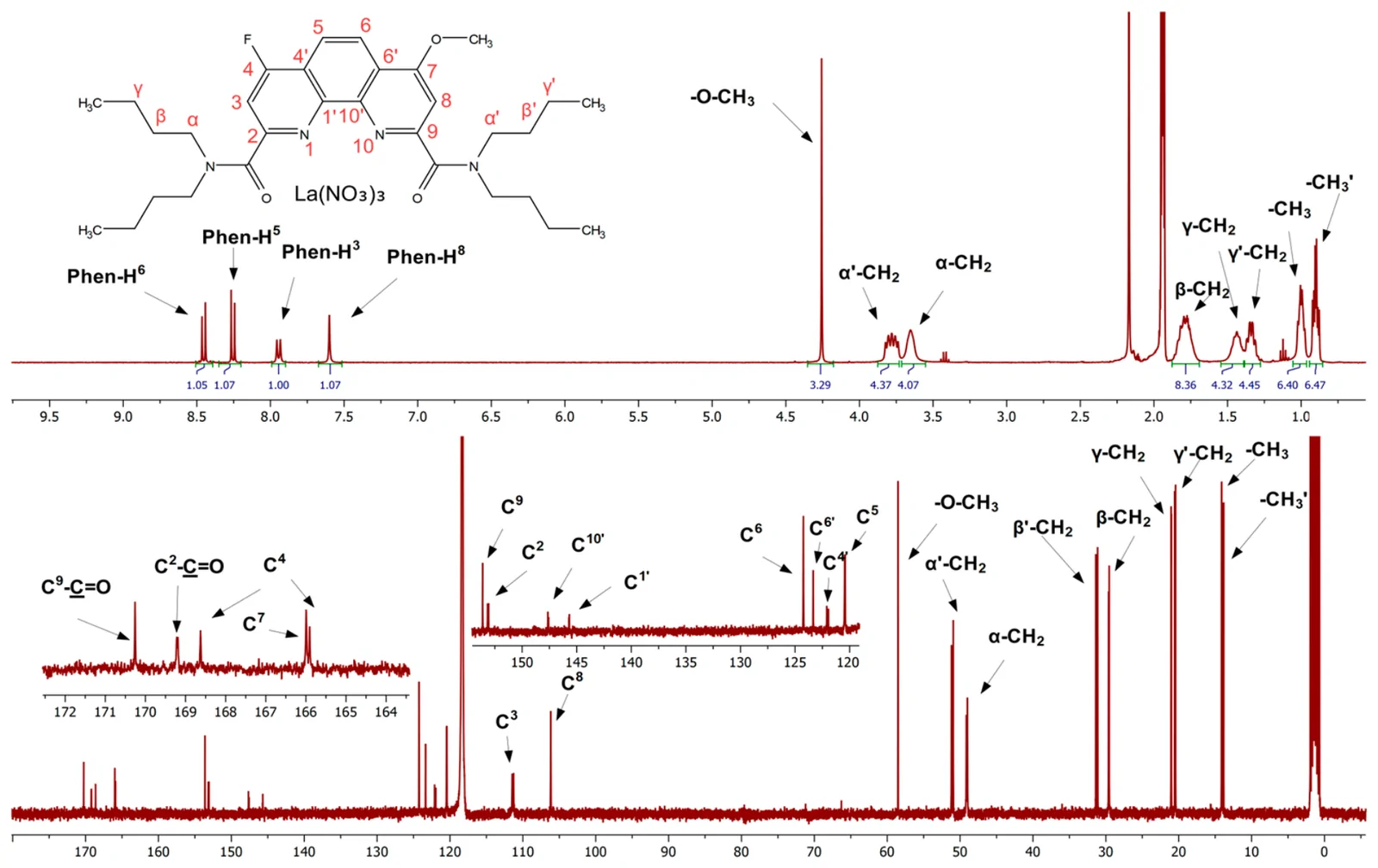
Complete assignment in the ^1^H and ^13^C NMR spectra of complexes **17a** with La(NO_3_)_3._

To demonstrate the ability of synthesized compounds to act as polydentate ligands toward *f*-block elements, we synthesized the individual complex of ligand **17a** with lanthanum trinitrate and confirmed its structure using a combination of spectroscopic methods. The ^1^H and ^13^C NMR spectra of this complex, with fully assigned signals, are shown in Figure 5. The assignment was performed using 2D NMR spectroscopy methods (see SI, Figures S93–S95).

From the point of view of studying the practical properties of DAPhen ligands, their physicochemical characteristics, such as logP and the pKb, play a significant role. We calculated these characteristics for a wide range of known ligands, differing in both substituents in the amide function and substituents in the 4,7-position. The complete table of calculated physicochemical characteristics is presented in ESI, Table S3.

**Figure 6:**
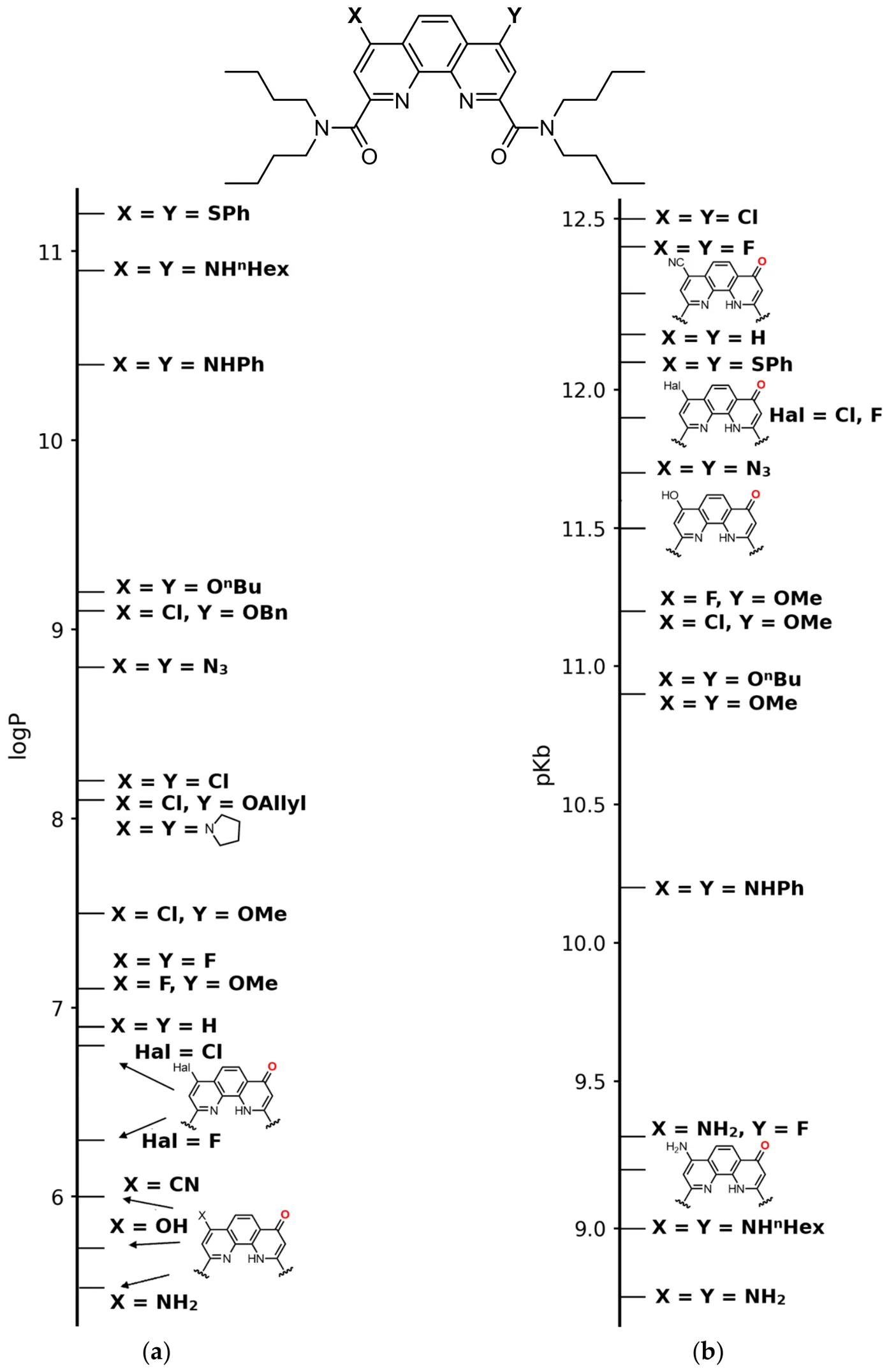
Comparison of calculated (**a**) logP and (**b**) pKb for selected model ligands.

From the data obtained, it is most important to note that a change in substituents in the amide function mainly affects the calculated logP value. However, the 4.7-functionalization can have an effect on both the logP value and the pKb value. Based on this, by choosing the dibutylamide-substituted DAPhen ligand as a model, we constructed the corresponding logP and pKb space for various 4,7-functionalized ligands, including those considered in this work (Figure 6). Thus, the implementation of 4,7-functionalization methods allows you to adjust the logP value within 5–6 units (approximating the same amide substituents), and the pKb value within 3–4 units. In the case of 4-oxo-substituted ligands, a change in the substituents at the 7-position can also have a significant effect on the basicity value.

The developed method is a universal approach for the synthesis of unsymmetric ligands with different structures; the presence of fluorine and chlorine atoms allows additional modification of the structure of tetradentate ligands. In addition, the developed 4, 7-functionalization methods make it possible to significantly vary the physicochemical characteristics of the obtained ligands, thus adjusting their extraction properties. A total of 26 examples of tetradentate ligands were obtained using this approach.

## 3. Materials and Methods

### 3.1. Materials

Inorganic/organic reagents and solvents were of analytical grade. Deuterated solvents CDCl_3_, C_6_D_6_, CD_3_OD, and CD_3_CN for NMR spectra registration were purchased from Sigma-Aldrich, Co. (St. Louis, MO, USA) and used without further purification. All syntheses were performed in an argon inert atmosphere. Dimethyl sulfoxide and dichloromethane were purified by distillation over calcium hydride prior to use. Acetonitrile, chloroform, diethyl ether, hexane and ethyl acetate for the synthesis were purified according to known procedures. Initial 1,10-phenanthroline-2,9-dicarboxamides **1**, **8** and **16** were synthesized according to known procedures [27,28].

### 3.2. Methods

NMR spectra were recorded using standard 5 mm sample tubes on an Agilent 400-MR spectrometer (Agilent Technologies, Santa Clara, CA, USA) with operating frequencies of 400 MHz (^1^H), 100 MHz (^13^C), 376 MHz (^19^F) and 162 MHz (^31^P). For compound **15b** NMR spectra were recorded using standard 5 mm sample tubes on a QOne ANS600 (Q.One Instruments, Wuhan, China) with operating frequencies of 600 MHz (^1^H), 151 MHz (^13^C). Preliminary ^1^H and ^19^F NMR spectra were recorded using a Magritek Spinsolve 60 MHz magnetic WELL spectrometer (Magritek, Aachen, Germany). Structural assignments were made with additional information from gCOSY, gHSQC, and gHMBC experiments. IR spectra in the solid state were recorded on a Nicolet iS5 FTIR spectrometer (Thermo Fisher Scientific, Waltham, MA, USA) using an internal reflectance attachment with diamond optical element attenuated total reflection (ATR) with a 45° angle of incidence. The resolution is 4 cm^−1^; the number of scans is 32. HRMS ESI mass spectra were recorded on a LCMS-IT-TOF (Shimadzu, Kyoto, Japan) in the negative and positive ion modes. Resolution of the instrument is >10,000 according to the manufacturer. LabSolution software (version 3.80.410, Shimadzu, Kyoto, Japan) was used to acquire and process MS data. The pKa and logP values were calculated using the MolGPka server (https://xundrug.cn/molgpka, accessed on 8 July 2026) [51].

The conformational search was performed using the program CREST 3.0 [52]. Quantum chemical calculations of tautomeric forms and conformational analysis were performed by the ORCA 6.0.1 program [53] DFT method, B3LYP functional, basis 6-31G(d,p). For modeling the alkylation reaction process, Gaussian-16 software [54] was used, with the DFT method, B3LYP (GD3BJ) functional, 6-31G(d,p) basis set. The starting geometries for the transition state search either were located by scan procedures or by a guess based on the structure of the previously found TS. The transition states were subsequently fully optimized as saddle points of the first order, employing the Berny algorithm. Frequency calculations were carried out to confirm the nature of the stationary points, yielding one imaginary frequency for all transition states. The solvent influence was accounted for by carrying out optimizations in PCM theory in chloroform.

Single crystals of **2b** were obtained upon slow isothermal (25 °C) recrystallization of corresponding substances from mixture MeCN/C_6_H_6_. Single crystals of **4b** were obtained upon slow isothermal (25 °C) recrystallization of corresponding substances from mixture CH_3_OH/C_6_H_6._

X-ray diffraction data for **2b** were collected on a four-circle Rigaku Synergy S diffractometer (Rigaku Corporation, Wrocław, Poland, 2021) equipped with a HyPix6000HE detector (*T* = 100 K, *λ*Cu*K*α-radiation, graphite monochromator, kappa geometry, shutterless *φ* and *ω*-scan mode). The data were integrated and corrected for absorption by the CrysAlisPro program (version 38.41) [55]. The structures were determined by direct methods and refined by full-matrix least squares technique on *F*^2^ with anisotropic displacement parameters for non-hydrogen atoms. For details, see Table S1. The hydrogen atoms of the amino groups in **2b** were localized in the difference-Fourier map and isotropically refined with fixed displacement parameters [*U*_iso_(H) = 1.2*U*_eq_(N) and 1.5*U*_eq_(O)]. The other hydrogen atoms were placed in calculated positions and refined within a riding model with fixed isotropic displacement parameters [*U*_iso_(H) = 1.5*U*_eq_(C) for the CH_3_-groups and 1.2*U*_eq_(C) for the other groups]. All calculations were carried out using the SHELXTL-2019 program [56].

X-ray diffraction data for **4b** were collected at 100 K with a Bruker APEXII Quazar CCD diffractometer (Bruker AXS GmbH, Karlsruhe, Germany), using graphite monochromated Mo-Kα radiation (λ = 0.71073 Å). Using Olex2 [57], the structure was solved with the ShelXT [56] structure solution program using Intrinsic Phasing and refined with the XL [58] refinement package using Least Squares minimization against F^2^_hkl_ in anisotropic approximation for non-hydrogen atoms. Hydrogen atoms of the NH group and lattice water molecules were located from the difference Fourier synthesis, the positions of other hydrogen atoms were calculated, and they all were refined in the isotropic approximation within the riding model. Crystal data and structure refinement parameters are given in Table S1.

Crystallographic data for **2b** and **4b** were deposited in the Cambridge Crystallographic Data Center, CCDC 2429885 (**2b**). CCDC 2423617 (**4b**). Copies of this information may be obtained free of charge from the Director, CCDC, 12 Union Road, Cambridge CB2 1EZ, UK (Fax: +44-1223-336033; e-mail: deposit@ccdc.cam.ac.uk or www.ccdc.cam.ac.uk).

### 3.3. Synthesis and Analytical Data

*General Procedure for Synthesis of Diamides **2**.*

*Method 1:* An aqueous ammonia solution (~13.3 M) (300 μL, 4 mmol) was added to a solution of the corresponding diamide **1** (0.1 mmol) in DMSO (2.5 mL). The mixture was stirred at 60 °C for 36 h until the reaction was complete (control by NMR ^19^F), after which the resulting solution was poured into ice. The resulting mixture was extracted with CH_2_Cl_2_ (3 × 20 mL). The organic phases were combined, washed with water (3 × 20 mL) and brine (1 × 20 mL), dried over sodium sulfate, filtered, and the solvent was evaporated. The residue was washed with diethyl ether (20 mL).

*Method 2:* The corresponding diamide **9** (0.1 mmol) was dissolved in a mixture of 1.5 mL of ethanol and 0.5 mL of water. The mixture was heated to 100 °C and stirred for 72 h. After the end of the reaction (control by ^31^P NMR), the reaction mixture was filtered, the solid residue was washed with MTBE (5 mL) and the solid residue was left to dry at room temperature.

*7-Amino-N^2^,N^2^,N^9^,N^9^-tetrabutyl-4-oxo-1,4-dihydro-1,10-phenanthroline-2,9-dicarboxamide* (**2a**). Yield 45 mg (86%) (method 1), 34 mg (65%) (method 2), white powder; mp 96–99 °C; ^1^H NMR (400 MHz, CDCl_3_) δ 10.50 (br s, 1H, NH), 8.23 (d, *J* = 9.1 Hz, 1H, Phen^5,6^), 7.60 (d, *J* = 9.1 Hz, 1H, Phen^5,6^), 6.92 (s, 1H), 6.54 (s, 1H), 5.23 (s, 2H, -NH_2_), 3.58–3.46 (m, 6H, -N-CH_2_-), 3.29 (t, *J* = 7.7 Hz, 2H, -N-CH_2_-), 1.76–1.59 (m, 8H), 1.50–1.35 (m, 4H), 1.32–1.20 (m, 2H), 1.18–1.08 (m, 2H), 1.03–0.91 (m, 6H, -CH_3_), 0.90–0.80 (m, 3H, -CH_3_), 0.75 (t, *J* = 7.3 Hz, 3H, -CH_3_); ^13^C NMR (101 MHz, CDCl_3_) 178.2 (C=O), 169.3 (C=O), 164.7 (C=O), 154.9, 151.6, 141.8, 138.7, 137.3, 124.6, 121.1, 118.4, 115.7, 111.3, 105.1, 48.6 (-N-CH_2_-), 45.3 (-N-CH_2_-), 31.1, 29.8, 20.5, 20.0, 14.1, 13.7; IR (ν, cm^−1^): 3403, 3349, 3324 (N-H), 2956, 2930, 2867 (C-H stretching vibrations), 1614 (C=O), 1584, 1547, 1519 (C=C, C=N); HRMS (ESI-TOF) (*m*/*z*) [M + Na]^+^ calcd for [C_30_H_43_N_5_O_3_Na]^+^ 544.3258 found 544.3256.

*7-Amino-N^2^,N^9^-diethyl-4-oxo-N^2^,N^9^-di-p-tolyl-1,4-dihydro-1,10-phenanthroline-2,9-dicarboxamide* (**2b**). Yield 39 mg (73%) (method 1), 38 mg (72%) (method 2), yellowish powder; mp 278–281 °C; ^1^H NMR (400 MHz, CDCl_3_) δ 10.35 (br s, 1H, NH), 8.00 (d, *J* = 9.0 Hz, 1H, Phen^5,6^), 7.41 (d, *J* = 9.0 Hz, 1H, Phen^5,6^), 7.19–7.08 (m, 5H, Ar-H + Phen^8^), 7.05–6.95 (m, 4H, Ar-H), 5.92 (s, 1H, Phen^3^), 5.00 (br s, 2H, -NH_2_), 4.12–3.96 (m, 4H, -N-CH_2_-), 2.31 (s, 3H, Ar-CH_3_), 2.21 (s, 3H, Ar-CH_3_), 1.37–1.27 (m, 6H, -CH_3_); ^13^C NMR (101 MHz, CDCl_3_) δ 178.0 (C=O), 168.0 (C=O), 163.4 (C=O), 153.3, 150.7, 141.0, 140.3, 139.1, 138.4, 138.3, 137.2, 136.5, 130.8, 129.9, 127.9, 127.0, 124.1, 121.5, 118.1, 115.0, 114.7, 106.8, 46.8 (-N-CH_2_-), 45.7 (-N-CH_2_-), 21.2 (Ar-CH_3_), 21.2 (Ar-CH_3_), 12.9 (-CH_3_), 12.7 (-CH_3_); IR (ν, cm^−1^) 3335, 3313, 3224 (N-H), 2971, 2931 (C-H stretching vibrations), 1652, 1647, 1619, (C=O), 1584, 1558, 1525, 1510, (C=C, C=N); HRMS (ESI-TOF) (*m*/*z*) [M + H]^+^ calcd for [C_32_H_32_N_5_O_3_]^+^ 534.2500, found 534.2511.

*General Procedure for Synthesis of Diamides **3**.* The corresponding diamide **1** (0.2 mmol) was dissolved in pyrrolidine (2 mL, 1.73 g, ∼24 mmol), and the reaction mixture was stirred at room temperature for 4 h (control by NMR ^19^F). The organic solvent was evaporated; the resulting residue was dissolved in ethyl acetate (20 mL) and washed with saturated sodium bicarbonate solution (20 mL), water (20 mL), and brine (20 mL). The combined organic fractions were dried over sodium sulfate and the solvent was evaporated under reduced pressure.

*N^2^,N^2^,N^9^,N^9^-Tetrabutyl-4-oxo-7-(pyrrolidin-1-yl)-1,4-dihydro-1,10-phenanthroline-2,9-dicarboxamide* (**3a**). Yield 100 mg (87%), yellow powder; mp 89–92 °C; ^1^H NMR (400 MHz, CDCl_3_) δ 10.68 (br s, 1H, NH), 8.11 (d, *J* = 9.4 Hz, 1H, Phen^5,6^), 8.08 (d, *J* = 9.4 Hz, 1H, Phen^5,6^), 6.76 (s, 1H, Phen^3,8^), 6.52 (s, 1H, Phen^3,8^), 3.81–3.74 (m, 4H, -N-CH_2_-), 3.58–3.45 (m, 6H, -N-CH_2_-), 3.32–3.24 (m, 2H, -N-CH_2_-), 2.15–2.04 (m, 4H, -CH_2_-), 1.77–1.56 (m, 10H, -CH_2_-), 1.52–1.38 (m, 4H, -CH_2_-), 1.20–1.08 (m, 2H, -CH_2_-), 1.01 (t, *J* = 7.4 Hz, 6H, -CH_3_), 0.91–0.80 (m, 3H, -CH_3_), 0.74 (t, *J* = 7.4 Hz, 3H, -CH_3_); ^13^C NMR (101 MHz, CDCl_3_) δ 178.2 (C=O), 169.5 (C=O), 164.7 (C=O), 154.1, 153.2, 142.0, 139.9, 137.1, 123.9, 120.4, 120.2, 118.8, 110.8, 103.9, 52.5 (-N-CH_2_-), 48.5 (-N-CH_2_-), 45.2 (-N-CH_2_-), 31.1, 29.7, 26.1, 20.5, 19.9, 14.0, 13.7; IR (ν, cm^−1^) 3249 (N-H), 2955, 2930, 2871 (C-H stretching vibrations), 1641, 1618 (C=O), 1583, 1538, 1522 (C=C, C=N); HRMS (ESI-TOF) (*m*/*z*) [M + Na]^+^ cacld for [C_34_H_49_N_5_NaO_3_]^+^ 598.3728, found 598.3734.

*N^2^,N^9^-Diethyl-4-oxo-7-(pyrrolidin-1-yl)-N^2^,N^9^-di-p-tolyl-1,4-dihydro-1,10-phenanthroline-2,9-dicarboxamide* (**3b**). Yield 99 mg (85%), yellow powder; mp 258–260 °C; ^1^H NMR (400 MHz, C_6_D_6_) δ 10.78 (br s, 1H, NH), 8.17 (d, *J* = 9.3 Hz, 1H, Phen^5^), 7.41–7.29 (m, 3H, Phen^6^, Ar^2,6^), 7.18 (s, 1H, Phen^8^), 7.02 (d, J = 7.9 Hz, 2H, Ar^3,5^), 6.72–6.57 (m, 4H, Ar^2,3,5,6^), 6.10 (s, 1H, Phen^3^), 3.99 (q, *J* = 7.1 Hz, 2H, -N-CH_2_), 3.75 (q, *J* = 7.1 Hz, 2H, -N-CH_2_), 2.83–2.70 (m, 4H, -N-CH_2_^Pyrr^-), 1.91 (s, 3H, Ar-CH_3_), 1.85 (s, 3H, Ar-CH_3_), 1.28–1.15 (m, 3H, -CH_3_), 1.15–1.02 (m, 7H, CH_3_, -CH_2_^Pyrr^- + -CH_3_); ^13^C NMR (101 MHz, C_6_D_6_) δ 177.1 (C^4^=O), 168.1 (C^9′^=O), 163.6 (C^2′^=O), 152.6 (C^7^), 142.0 (Ar-C^1^), 139.7 (Phen-C^2^), 139.5 (Ar-C^1^), 139.4 (Phen-C^9^), 137.5 (Ar-C^4^), 137.1 (Phen-C^1′,10′^), 135.8 (Ar-C^4^), 130.2 (Ar-C^3,5^), 129.8 (Ar-C^3,5^), 128.0 (Ar-C^2,6^), 126.8 (Ar-C^2,6^), 124.0 (Phen-C^4′^), 120.0 (Phen-C^6′^), 119.4 (Phen-C^5,6^), 114.9 (Phen-C^3^), 105.9 (Phen-C^8^), 51.3 (-N-CH_2_^Pyrr^-), 46.1 (-N-CH_2_), 45.5 (-N-CH_2_), 25.2 (-CH_2_^Pyrr^-), 20.6 (Ar-CH_3_), 20.4 (Ar-CH_3_), 12.7 (CH_3_), 12.2 (CH_3_); IR (ν, cm^−1^) 3324 (N-H), 3028, 2975, 2935, 2868 (C-H stretching vibrations), 1643, 1621, 1605 (C=O), 1575, 1525, 1510 (C=C, C=N); HRMS (ESI-TOF) (*m*/*z*) [M + Na]^+^ calcd for [C_36_H_37_N_5_O_3_Na]^+^ 610.2789, found 610.2794.

*General Procedure for Synthesis of Diamides **4**.* A solution of the corresponding diamide **1** (0.06 mmol), 15-crown-5 (0.01 mmol) and tetramethylammonium chloride (0.01 mmol) in 2 mL of DMSO in an argon atmosphere was mixed at 85 °C until homogeneity was achieved. After achieving homogeneity, sodium cyanide (0.3 mmol) and 0.5 mL DMSO were added and stirred at the same temperature for 2 h. After this, the reaction mixture was poured into water, extracted with ethyl acetate (2 × 30 mL) and dried over sodium sulfate, and the solvent was evaporated under reduced pressure. The residue was purified by column chromatography using the eluent hexane → hexane:ethyl acetate (3:2).

*N^2^,N^2^,N^9^,N^9^-Tetrabutyl-7-cyano-4-oxo-1,4-dihydro-1,10-phenanthroline-2,9-dicarboxamide* (**4a**). Yield 24 mg (76%), yellow powder; *R_f_* = 0.4 (Hexane:EtOAc (7:3)); mp 111–113 °C; ^1^H NMR (400 MHz, CDCl_3_) δ 10.49 (br s, 1H, NH), 8.57 (d, *J* = 9.0 Hz, 1H, Phen^5,6^), 8.13 (s, 1H, Phen^8^), 8.00 (d, *J* = 9.0 Hz, 1H, Phen^5,6^), 6.63 (s, 1H, Phen^3^), 3.63–3.45 (m, 6H, -N-CH_2_-), 3.37–3.25 (m, 2H, -N-CH_2_-), 1.77–1.60 (m, 8H), 1.50–1.34 (m, 4H), 1.19–1.08 (m, 2H), 1.04–0.84 (m, 10H), 0.76 (t, *J* = 7.3 Hz, 3H, -CH_3_); ^13^C NMR (101 MHz, CDCl_3_) δ 177.6 (C=O), 166.4 (C=O), 164.1 (C=O), 153.6, 141.8, 137.9, 137.0, 127.5, 127.1, 127.1, 125.3, 120.5, 119.1, 114.5, 112.6, 48.8 (-N-CH_2_-), 45.9 (-N-CH_2_-), 31.2, 29.6, 20.4, 20.0, 14.0, 13.7; IR (ν, cm^−1^) 3367 (N-H), 2955, 2929, 2871 (C-H stretching vibrations), 2234 (C≡N), 1616 (C=O), 1590, 1519 (C=C, C=N); HRMS (ESI-TOF) (*m*/*z*) [M + Na]^+^ calcd for [C_31_H_41_N_5_O_3_Na]^+^ 554.3102, found 554.3111.

*7-Cyano-N^2^,N^9^-diethyl-4-oxo-N^2^,N^9^-di-p-tolyl-1,4-dihydro-1,10-phenanthroline-2,9-dicarboxamide* (**4b**). Yield 24 mg (78%), yellow powder; *R_f_* = 0.5 (EtOAc); mp 253–255 °C; ^1^H NMR (400 MHz, C_6_D_6_) δ 10.26 (br s, 1H, NH), 8.12 (d, *J* = 8.7 Hz, 1H, Phen^5,6^), 7.95 (s, 1H, Phen^8^), 7.19 (d, *J* = 8.7 Hz, 1H, Phen^5,6^), 7.10–6.97 (m, 4H, Ar), 6.76–6.63 (m, 4H, Ar), 6.01 (s, 1H, Phen^3^), 3.87 (q, *J* = 7.1 Hz, 2H, -N-CH_2_-), 3.76 (q, *J* = 7.1 Hz, 2H, -N-CH_2_-), 1.95 (s, 3H, Ar-CH_3_), 1.90 (s, 3H, Ar-CH_3_), 1.20–1.05 (m, 6H, -CH_3_); ^13^C NMR (101 MHz, C_6_D_6_) δ 176.7 (C=O), 165.4 (C=O), 163.2 (C=O), 152.2, 141.1, 139.7, 139.4, 138.6, 137.3, 137.0, 136.8, 130.8, 130.4, 128.5, 128.2, 127.9, 127.3, 127.1, 125.2, 119.9, 118.5, 116.4, 114.8, 46.8 (-N-CH_2_), 45.9 (-N-CH_2_), 20.9 (Ar-CH_3_), 20.8 (Ar-CH_3_), 12.7 (-CH_3_), 12.5 (-CH_3_); IR (ν, cm^−1^) 3334 (N-H), 3062, 3034, 2966, 2929, 2871 (C-H stretching vibrations), 2234 (C≡N), 1629 (C=O), 1596, 1550, 1528, 1510 (C=C, C=N); HRMS (ESI-TOF) (*m*/*z*) [M + Na]^+^ calcd for [C_33_H_29_N_5_O_3_Na]^+^ 566.2163, found 566.2168.

*N^2^,N^2^,N^9^,N^9^-Tetrabutyl-4-oxo-7-(1,4,7,10,13-pentaoxa-16-azacyclooctadecan-16-yl)-1,4-dihydro-1,10-phenanthroline-2,9-dicarboxamide* (**5a**). A solution of 1-aza-18-crown-6 (320 mg, 1.24 mmol) and triethylamine (31 mg, 0.31 mmol) in DMSO (3 mL) was added to a solution of diamide **1a** (162 mg, 0.31 mmol) in DMSO (3 mL). The resulting mixture was stirred at 85 °C for 24 h. After the end of the reaction (control by NMR ^19^F), the reaction mixture was poured into ice and extracted with ethyl acetate (6 × 30 mL), and the combined organic fractions were washed with water (3 × 30 mL) and brine (30 mL). The combined organic fractions were dried over sodium sulfate and filtered, and the solvent was evaporated. The resulting solid residue was purified by column chromatography using EtOAc→EtOAc:EtOH (3:1) as an eluent. Yield 130 mg (55%), yellow oil; *R_f_* = 0.1 (EtOAc:EtOH (9:1)); ^1^H NMR (400 MHz, CDCl_3_) δ 10.51 (br s, 1H, NH), 8.23 (d, *J* = 9.2 Hz, 1H, Phen^5,6^), 8.02 (d, *J* = 9.2 Hz, 1H, Phen^5,6^), 7.25 (s, 1H, Phen), 6.54 (s, 1H, Phen), 3.80 (t, *J* = 5.5 Hz, 4H, -N-CH_2_-), 3.75–3.61 (m, 20H, -N-CH_2_- + -O-CH_2_-), 3.57–3.47 (m, 6H, -N-CH_2_- + -O-CH_2_-), 3.28 (t, *J* = 7.6 Hz, 2H, -N-CH_2_-), 1.79–1.69 (m, 2H), 1.69–1.56 (m, 6H), 1.52–1.35 (m, 4H), 1.24–1.05 (m, 4H), 1.04–0.82 (m, 9H), 0.73 (t, *J* = 7.3 Hz, 3H, -CH_3_); ^13^C NMR (101 MHz, CDCl_3_) δ 178.2 (C=O), 169.0 (C=O), 164.8 (C=O), 157.8, 154.5, 141.7, 139.6, 137.5, 124.3, 124.3, 121.2, 119.7, 111.4, 111.2, 70.9, 70.8, 70.8, 70.6, 69.2, 52.9, 48.7, 45.5, 31.2, 29.8, 20.5, 20.0, 14.1, 13.7; IR (ν, cm^−1^) 3356 (N-H), 2954, 2929, 2868 (C-H stretching vibrations), 1618, 1580 (C=O), 1517, 1456 (C=C, C=N); HRMS (ESI-TOF) (*m*/*z*) [M + Na]^+^ calcd for [C_42_H_65_N_5_O_8_Na]^+^ 790.4725, found 790.4721

*General Procedure for Synthesis of Diamides **6***

Sodium azide (195 mg, 3 mmol) was added to the solution of the corresponding diamide **1** (0.6 mmol) in DMSO (15 mL). The mixture was stirred at 85 °C for 5 h (control by ^19^F NMR); after the end of the reaction, the resulting solution was poured into ice. The resulting mixture was extracted with ethyl acetate (3 × 20 mL). The combined organic fractions were washed with water (3 × 20 mL) and brine (20 mL). After that, the organic fractions were dried over sodium sulfate, filtered and evaporated. The resulting residue was additionally washed with a hexane (10 mL).

*7-Azido-N^2^,N^2^,N^9^,N^9^-tetrabutyl-4-oxo-1,4-dihydro-1,10-phenanthroline-2,9-dicarboxamide* (**6a**). Yield 299 mg (91%), yellow powder; mp 142 °C (decomp.); ^1^H NMR (400 MHz, CDCl_3_) δ 10.41 (br s, 1H, NH), 8.36 (d, *J* = 9.1 Hz, 1H, Phen^5,6^), 7.89 (d, *J* = 9.1 Hz, 1H, Phen^5,6^), 7.56 (s, 1H, Phen^3,8^), 6.59 (s, 1H, Phen^3,8^), 3.61–3.56 (m, 2H, -N-CH_2_-), 3.55–3.47 (m, 4H, -N-CH_2_-), 3.36 (t, *J* = 7.7 Hz, 2H, -N-CH_2_-), 1.82–1.72 (m, 2H, -CH_2_-), 1.70–1.58 (m, 4H, -CH_2_-), 1.52–1.34 (m, 4H, -CH_2_-), 1.33–1.22 (m, 2H, -CH_2_-), 1.21–1.11 (m, 2H, -CH_2_-), 1.06–0.94 (m, 6H, -CH_3_), 0.92–0.82 (m, 3H, -CH_3_), 0.81–0.73 (m, 3H, -CH_3_); ^13^C NMR (101 MHz, CDCl_3_) δ 178.0 (C=O), 167.7 (C=O), 164.5 (C=O), 154.6, 148.2, 141.7, 138.7, 136.7, 125.3, 123.8, 122.4, 117.0, 112.0, 111.0, 48.8 (-N-CH_2_-), 45.9 (-N-CH_2_-), 31.3, 29.8, 20.6, 20.1, 14.1 (-CH_3_), 13.76 (-CH_3_); IR (ν, cm^−1^): 3045, 2956, 2929, 2807 (C-H stretching vibrations), 2121 (-N_3_), 1640, 1620, 1607 (C=O), 1566, 1556, 1518 (C=C, C=N); HRMS (ESI-TOF) (*m*/*z*) [M + Na]^+^ calcd for [C_30_H_41_N_7_NaO_3_]^+^ 570.3163 found 570.3170.

*7-Azido-N^2^,N^9^-diethyl-4-oxo-N^2^,N^9^-di-p-tolyl-1,4-dihydro-1,10-phenanthroline-2,9-dicarboxamide* (**6b**). Yield 285 mg (85%), yellow powder; mp 194 °C (decomp.); ^1^H NMR (400 MHz, C_6_D_6_) δ 10.52 (br s, 1H, NH), 8.20 (d, *J* = 9.1 Hz, 1H, Phen), 7.66 (s, 1H, Phen), 7.26–7.19 (m, 3H, Phen + Ar), 7.05 (d, *J* = 8.0 Hz, 2H, Ar), 6.80–6.64 (m, 4H, Ar), 6.08 (s, 1H, Phen), 3.95 (q, *J* = 7.1 Hz, 2H, -N-CH_2_), 3.78 (q, *J* = 7.1 Hz, 2H, -N-CH_2_), 1.97 (s, 3H, Ar-CH_3_), 1.92 (s, 3H, Ar-CH_3_), 1.20 (t, *J* = 6.6 Hz, 3H, -CH_3_), 1.13 (t, *J* = 7.0 Hz, 3H, -CH_3_); ^13^C NMR (101 MHz, C_6_D_6_) δ 177.0 (C=O), 166.6 (C=O), 163.4 (C=O), 153.3, 147.3, 139.8, 139.5, 138.5, 138.4, 136.7, 130.7, 130.4, 128.5, 127.2, 125.3, 124.3, 122.1, 116.3, 116.0, 112.2, 46.7 (-N-CH_2_-), 46.0 (-N-CH_2_-), 21.0 (Ar-CH_3_), 20.8 (Ar-CH_3_), 12.9 (-CH_3_), 12.6 (-CH_3_); IR (ν, cm^−1^) 3359, 3322 (N-H), 3034, 2974, 2934, 2872 (C-H stretching vibrations), 2161, 2120 (-N_3_), 1645, 1623 (C=O), 1589, 1553, 1527, 1512 (C=C, C=N); HRMS (ESI-TOF) (*m*/*z*) [M + Na]^+^ calcd for [C_32_H_29_N_7_O_3_Na]^+^ 582.2225, found 582.2228.

*Large-scale synthesis of diamide* **6b**.

Sodium azide (325 mg, 5 mmol, 5 mol equiv.) was added to the suspension of the **1b** (537 mg, 1 mmol) in DMSO (10 mL). The mixture was stirred at 85 °C for 5 h (control by ^19^F NMR); after the end of the reaction, the resulting solution was poured into ice. The resulting mixture was extracted with ethyl acetate (3 × 50 mL). The combined organic fractions were washed with water (5 × 50 mL) and brine (50 mL). After that, the organic fractions were dried over sodium sulfate, filtered and evaporated. The resulting residue was additionally washed with a hexane (15 mL). Yield 459 mg (82%).

*N^2^,N^9^-Diethyl-7-methoxy-4-oxo-N^2^,N^9^-di-p-tolyl-1,4-dihydro-1,10-phenanthroline-2,9-dicarboxamide* (**7b**). *Method 1:* Corresponding diamide **1b** (54 mg, 0.1 mmol) was added to the suspension of potassium carbonate (35 mg, 0.25 mmol) in methanol (3 mL) and stirred at 50 °C for 8 h. After the end of the reaction (control by NMR ^19^F), the reaction mixture was concentrated, diluted with ethyl acetate (25 mL), and then washed with an aqueous solution of acetic acid (25 mL, pH = 4), water (25 mL), and saturated sodium chloride solution (25 mL). The organic fraction was dried over sodium sulfate and filtered, and then the organic solvent was evaporated. *Method 2:* Diamide **8b** (28 mg, 0.05 mmol) was added to the suspension of potassium carbonate (8 mg, 0.055 mmol) in methylene chloride (1 mL). The resulting mixture was stirred at room temperature for 30 min, after which a solution of methyl iodide (8 mg, 0.055 mmol) in methylene chloride (1 mL) was added. The solution was stirred by boiling in a closed vial for 20 h. After the end of the reaction (TLC control), the organic solvent was evaporated and the resulting solid residue was washed with water (15 mL) and extracted with methylene chloride (3 × 20 mL). The combined organic fractions were dried over sodium sulfate, filtered and evaporated. Yield 37 mg (67%) (*method 1*), 23 mg (84%) (*method 2*), yellowish powder; *R_f_* = 0.16 (Hexane:EtOAc (7:3)); mp 135–137 °C; ^1^H NMR (400 MHz, CDCl_3_) δ 10.29 (br s, 1H, NH), 8.09 (d, *J* = 9.0 Hz, 1H, Phen^5,6^), 7.83 (d, *J* = 9.0 Hz, 1H, Phen^5,6^), 7.35 (s, 1H, Phen^8^), 7.18–7.09 (m, 4H, Ar-H), 7.07–6.97 (m, 4H, Ar-H), 5.93 (s, 1H, Phen^3^), 4.11–3.96 (m, 7H, -N-CH_2_- + -OCH_3_), 2.30 (s, 3H, Ar-CH_3_), 2.24 (s, 3H, Ar-CH_3_), 1.38–1.30 (m, 6H, -CH_3_); ^13^C NMR (101 MHz, CDCl_3_) δ 177.4 (C=O), 166.9, 162.8, 162.6, 153.6, 140.4, 139.7, 138.5, 137.9, 137.7, 136.2, 136.1, 130.3, 129.5, 127.4, 126.4, 123.9, 122.1, 121.1, 115.8, 114.3, 103.1, 55.8 (-OCH_3_), 46.3 (-N-CH_2_-), 45.3 (-N-CH_2_-), 20.7 (Ar-CH_3_), 12.3 (-CH_3_), 12.1 (-CH_3_); IR (ν, cm^−1^): 3331 (N-H), 3032, 2973, 2931, 2870 (C-H stretching vibrations), 1645, 1621 (C=O), 1592, 1558, 1511 (C=C, C=N); HRMS (ESI-TOF) (*m*/*z*) [M + Na]^+^ calcd for [C_33_H_32_N_4_NaO_4_]^+^ 571.2316 found 571.2335.

*General Procedure for Synthesis of Diamides **9.*** A solution of triphenylphosphine (55 mg, 0.21 mmol) in methylene chloride (5 mL) was added to a solution of diamide **6** (0.2 mmol) in methylene chloride (3 mL) at 5 °C. After that, the reaction mixture was stirred at room temperature overnight. After the end of the reaction (control by ^31^P NMR), the reaction mixture was evaporated, and the resulting solid residue was washed with diethyl ether, filtered and dried.

*N^2^,N^2^,N^9^,N^9^-Tetrabutyl-4-oxo-7-((triphenylphosphoranylidene)amino)-1,4-dihydro-1,10-phenanthroline-2,9-dicarboxamide* (**9a**). Yield 105 mg (67%), yellow powder; mp 164–166 °C; ^1^H NMR (400 MHz, CDCl_3_) δ 8.61 (d, *J* = 9.1 Hz, 1H, Phen^5,6^), 8.23 (d, *J* = 9.1 Hz, 1H, Phen^5,6^), 7.90–7.70 (m, 6H, Ar), 7.64–7.38 (m, 9H), 6.57 (s, 1H, Phen^3,8^), 6.34 (s, 1H, Phen^3,8^), 3.53–3.32 (m, 6H, -N-CH_2_-), 3.00 (t, *J* = 7.7 Hz, 2H, -N-CH_2_-), 1.70–1.51 (m, 6H, -CH_2_-), 1.46–1.14 (m, 10H, -CH_2_-), 1.08–0.84 (m, 9H, -CH_2_- + -CH_3_), 0.59 (t, *J* = 7.3 Hz, 3H, -CH_3_); ^13^C NMR (101 MHz, CDCl_3_) δ 178.6 (C=O), 169.8 (C=O), 164.9 (C=O), 158.1, 154.3, 141.8, 139.0, 139.0, 137.2, 132.7, 129.2, 128.4, 128.2, 124.5, 120.5, 119.5, 110.5, 110.2, 110.0, 49.3 (-N-CH_2_-), 48.1 (-N-CH_2_-), 45.5 (-N-CH_2_-), 44.6 (-N-CH_2_-), 30.9, 30.7, 29.7, 29.6, 29.4, 21.3, 20.4, 20.3, 19.9, 19.7, 14.0 (-CH_3_), 13.8 (-CH_3_), 13.6 (-CH_3_); ^31^P NMR (162 MHz, CDCl_3_) δ 10.1; IR (ν, cm^−1^) 3349 (N-H), 2955, 2929, 2870 (C-H stretching vibrations), 1615 (C=O), 1568, 1518, 1474, 1456, 1403 (C=C, C=N, P=N), 1107, 1077 (P-N-C), 718, 692 (P-C); HRMS (ESI-TOF) (*m*/*z*) [M + Na]^+^ calcd for [C_48_H_56_N_5_O_3_PNa]^+^ 804.4013, found 804.4018.

*N^2^,N^9^-Diethyl-4-oxo-N^2^,N^9^-di-p-tolyl-7-((triphenylphosphoranylidene)amino)-1,4-dihydro-1,10-phenanthroline-2,9-dicarboxamide* (**9b**). Yield 114 mg (72%), yellow powder; mp 165–167 °C; ^1^H NMR (400 MHz, C_6_D_6_) δ 10.71 (br s, 1H, NH), 8.78 (d, *J* = 9.0 Hz, 1H, Phen^5,6^), 8.53 (d, *J* = 9.0 Hz, 1H, Phen^5,6^), 7.71–7.63 (m, 6H), 7.09–6.91 (m, 13H), 6.71–6.65 (m, 2H), 6.65–6.59 (m, 2H), 3.87 (q, *J* = 7.1 Hz, 2H, -N-CH_2_-), 3.77 (q, *J* = 7.1 Hz, 2H, -N-CH_2_-), 1.96 (s, 3H, Ar-CH_3_), 1.90 (s, 3H, Ar-CH_3_), 1.14–1.06 (m, 6H, -CH_3_); ^13^C NMR (101 MHz, C_6_D_6_) δ 177.8 (C=O), 168.7 (C=O), 164.0 (C=O), 158.2, 139.8, 139.7, 139.2, 137.8, 137.6, 135.8, 132.9, 132.8, 132.4, 132.4, 130.6, 130.0, 129.2, 129.0, 127.2, 125.2, 121.0, 120.1, 115.1, 112.9, 112.8, 46.5 (-N-CH_2_-), 45.5 (-N-CH_2_-), 21.0 (CH_3_), 20.8 (CH_3_), 13.0 (CH_3_), 12.62 (CH_3_); ^31^P NMR (162 MHz, C_6_D_6_) δ 9.2; IR (ν, cm^−1^) 3329 (N-H), 3055, 3033, 2973, 2932, 2871 (C-H stretching vibrations), 1650, 1645, 1616 (C=O), 1574, 1568, 1519, 1511, 1402 (C=C, C=N, P=N), 1108, 1079 (P-N-C), 717, 692 (P-C); HRMS (ESI-TOF) (*m*/*z*) [M + Na]^+^ calcd for [C_50_H_44_N_5_O_3_PNa]^+^ 816.3074, found 816.3078.

*Triethyl (2,9-bis(ethyl(p-tolyl)carbamoyl)-7-oxo-7,10-dihydro-1,10-phenanthrolin-4-yl)phosphorimidate* (**10b**). Triethylphosphite (17 mg, 0.1 mmol) was added to a solution of corresponding diamide **6b** (50 mg, 0.089 mmol) in methylene chloride (3 mL). The resulting solution was stirred at room temperature overnight, after which the organic solvent was evaporated, and the resulting solid residue was washed with hexane (10 mL). Yield 58 mg (93%), yellow oil; ^1^H NMR (400 MHz, CDCl_3_) δ 10.43 (br s, 1H, NH), 8.07 (d, *J* = 8.9 Hz, 1H, Phen^5,6^), 7.99 (d, *J* = 10.4 Hz, 1H, Phen^5,6^), 7.31 (s, 1H, Phen^3,8^), 7.20–7.12 (m, 4H, Ar-H), 7.09–7.04 (m, 4H, Ar-H), 5.97 (s, 1H, Phen^3,8^), 4.29–4.18 (m, 6H, -O-CH_2_-), 4.11–4.00 (m, 4H, -N-CH_2_-), 2.34 (s, 3H, Ar-CH_3_), 2.24 (s, 3H, Ar-CH_3_), 1.47–1.40 (m, 9H, -CH_3_), 1.38–1.31 (m, 6H, -CH_3_); ^13^C NMR (101 MHz, CDCl_3_) δ 178.3 (C=O), 163.8 (C=O), 159.1 (C=O), 140.1, 139.2, 138.2, 137.0, 130.7, 130.7, 130.6, 129.8, 129.7, 127.9, 127.0, 120.2, 119.4, 114.3, 113.9, 112.6, 108.8, 64.8 (-O-CH_2_-), 46.6 (-N-CH_2_-), 45.4 (-N-CH_2_-), 21.2 (Ar-CH_3_), 21.16 (Ar-CH_3_), 16.29 (-CH_3_), 13.0 (-CH_3_). 12.7 (-CH_3_); ^31^P NMR (162 MHz, CDCl_3_) δ 1.5; IR (ν, cm^−1^): 3330 (N-H), 2971, 2925, 2870, 2850 (C-H stretching vibrations), 1647, 1620, 1607 (C=O), 1589, 1579, 1525, 1511, 1492, 1429 (C=C, C=N, P=N), 1147, 1126, 1107 (P-N-C), 1056, 1017, 975 (P-O-Alk); HRMS (ESI-TOF) (*m*/*z*) [M + Na]^+^ calcd for [C_38_H_44_N_5_O_6_PNa]^+^ 720.2921, found 720.2925.

*Diethyl (2,9-bis(ethyl(p-tolyl)carbamoyl)-7-oxo-7,10-dihydro-1,10-phenanthrolin-4-yl)phosphoramidate* (**11b**). A 5% hydrochloric acid solution (2 mL) was added to the solution of the corresponding diamide **10b** (48 mg, 0.069 mmol) in methylene chloride (2 mL) and the resulting mixture was stirred at room temperature for 2 h. After that, the organic phase was separated, the aqueous phase was extracted with methylene chloride (3 × 5 mL), the combined organic fractions were dried over sodium sulfate, and the solvent was evaporated. Yield 40 mg (87%), yellowish powder; mp 163–165 °C; ^1^H NMR (400 MHz, CDCl_3_) δ 10.41 (br s, 1H, NH), 8.11 (d, *J* = 9.0 Hz, 1H, Phen^5,6^), 7.96 (d, *J* = 9.0 Hz, 1H, Phen^5,6^), 7.76 (m, 1H, -NH-P), 7.64 (s, 1H, Phen^8^), 7.18–7.07 (m, 4H, Ar-H), 7.07–6.96 (m, 4H, Ar-H), 5.95 (s, 1H, Phen^3^), 4.29–4.17 (m, 2H, -N-CH_2_-), 4.17–3.96 (m, 6H, -O-CH_2_- + -N-CH_2_-), 2.30 (s, 3H, Ar-CH_3_), 2.20 (s, 3H, Ar-CH_3_), 1.44–1.25 (m, 12H, -CH_3_); ^13^C NMR (101 MHz, CDCl_3_) δ 177.9 (C=O), 167.6 (C=O), 163.4 (C=O), 153.6, 145.5, 140.5, 139.0, 138.5, 138.4, 137.0, 136.7, 130.8, 130.0, 128.0, 127.0, 124.0, 122.8, 120.3, 120.1, 115.6, 114.9, 109.6, 63.9 (d, *J* = 5.1 Hz, -OCH_2_-), 46.8 (-N-CH_2_-), 45.5 (-N-CH_2_-), 21.2 (Ar-CH_3_), 21.1 (Ar-CH_3_), 16.3 (d, *J* = 6.4 H, -CH_3_), 13.0 (-CH_3_), 12.7 (-CH_3_);^31^P NMR (162 MHz, CDCl_3_) δ 0.0; IR (ν, cm^−1^): 3324, 3194 (N-H), 3033, 2977, 2931, 2871 (C-H stretching vibrations), 1683, 1651, 1646, 1621 (C=O), 1557, 1526, 1510 (C=C, C=N), 1255, 1219, 1166 (P=O), 1137, 1123, (P-O-Alk), 966 (P-N); HRMS (ESI-TOF) (*m*/*z*) [M + H]^+^ calcd for [C_36_H_41_N_5_O_6_P]^+^ 670.2789, found 670.2806.

*7-(5-Amino-4-cyano-1H-1,2,3-triazol-1-yl)-N^2^,N^9^-diethyl-4-oxo-N^2^,N^9^-di-p-tolyl-1,4-dihydro-1,10-phenanthroline-2,9-dicarboxamide* (**12b**). A solution of malonodinitrile (7 mg, 0.106 mmol) in ethanol (1 mL) and triethylamine (1 mg, 0.009 mmol) was added to a solution of the corresponding azide **6b** (50 mg, 0.09 mmol) in ethanol (2 mL). The mixture was stirred at 75 °C for 24 h, after which the solvent was evaporated. The resulting residue was washed with ether, filtered and additionally washed with ether and dried at room temperature. Yield 40 mg (71%), dark-yellow powder; mp 209–212 °C; ^1^H NMR (400 MHz, CDCl_3_) δ 11.03–9.91 (m, 1H, NH), 8.30–7.75 (m, 1H, Phen), 7.66–7.30 (m, 1H, Phen), 7.24–6.51 (m, 8H, Ar-H), 5.94 (s, 1H, Phen), 5.34 (s, 1H, Phen), 4.19–3.90 (m, 4H, -N-CH_2_-), 3.34–2.94 (m, 2H, -NH_2_), 2.28 (s, 3H, Ar-CH_3_), 2.10 (s, 3H, Ar-CH_3_), 1.41–1.28 (m, 6H, -CH_3_); ^13^C NMR (101 MHz, CDCl_3_) δ 177.8 (C=O), 168.0 (C=O), 163.3 (C=O), 151.3, 145.3, 140.7, 139.0, 138.4, 138.1, 137.1, 136.8, 136.6, 130.8, 129.9, 127.9, 127.0, 123.9, 122.3, 120.9, 118.0, 115.9, 114.7, 114.3, 108.7, 106.7, 46.9 (-N-CH_2_-), 46.8 (-N-CH_2_-), 21.7 (Ar-CH_3_), 21.2 (Ar-CH_3_), 12.9 (-CH_3_), 12.7 (-CH_3_); IR (ν, cm^−1^): 3404, 3321, 3221 (N-H), 2921, 2851 (C-H stretching vibrations), 2188, 2148, (CN), 1651, 1622, 1604, (C=O), 1583, 1549, 1509, 1451, 1429, 1402, (C=C, C=N); HRMS (ESI-TOF) (*m*/*z*) [M + H]^+^ calcd for [C_35_H_32_N_9_O_3_]^+^ 626.2623, found 626.2637.

*N^2^,N^9^-Diethyl-7-(4,5,6,7,8,9-hexahydro-1H-cycloocta[d][1,2,3]triazol-1-yl)-4-oxo-N^2^,N^9^-di-p-tolyl-1,4-dihydro-1,10-phenanthroline-2,9-dicarboxamide* (**13b**). A solution of cyclooctyne (6 mg, 0.55 mmol) in dichloromethane (1 mL) was added to a solution of the starting azide **6b** (28 mg, 0.05 mmol) in dichloromethane (1 mL) at 25 °C, and the mixture was stirred at room temperature overnight. The organic solvent was then evaporated, and the resulting solid residue was washed with diethyl ether, filtered, and dried at room temperature. Yield 28 mg (84%), yellowish powder; mp 134–137 °C; ^1^H NMR (400 MHz, C_6_D_6_) δ 10.39 (br s, 1H, NH), 8.13 (d, *J* = 9.0 Hz, 1H, Phen^6^), 8.00 (s, 1H, Phen^8^), 7.09 (d, *J* = 7.8 Hz, 2H, Ar), 6.99 (d, *J* = 7.9 Hz, 2H, Ar), 6.85 (d, *J* = 9.0 Hz, 1H, Phen^5^), 6.76 (d, *J* = 8.2 Hz, 2H, Ar), 6.70 (d, *J* = 8.2 Hz, 2H, Ar), 6.00 (s, 1H, Phen^3^), 3.86 (q, *J* = 7.1 Hz, 2H, -N-CH_2_-), 3.73 (q, *J* = 7.1 Hz, 2H, -N-CH_2_), 2.89 (t, *J* = 6.4 Hz, 2H, -CH_2_-), 2.02 (t, *J* = 6.0 Hz, 2H, -CH_2_-), 1.93 (s, 3H, Ar-CH_3_), 1.85 (s, 3H, Ar-CH_3_), 1.61–1.55 (m, 2H, -CH_2_-), 1.22–1.15 (m, 4H, -CH_2_-), 1.15–1.05 (m, 8H, -CH_2_- + -CH_3_); ^13^C NMR (101 MHz, C_6_D_6_) δ 176.8 (C^4^=O), 166.0 (C^9^-C=O), 163.4 (C^2^-C=O), 153.4, 145.0 (C^Triaz^), 142.4, 141.4, 139.9, 139.5, 139.1, 138.5, 136.9, 136.8, 135.4 (C^Triaz^), 130.7 (C^Ar^), 130.5 (C^Ar^), 128.5 (C^Ar^), 127.2 (C^Ar^), 126.5 (Phen^6^), 125.8 (Phen^6′^), 125.1 (Phen^4′^), 122.1 (Phen^8^), 116.8 (Phen^5^), 116.2 (Phen^3^), 46.7 (-N-CH_2_-), 46.0 (-N-CH_2_-), 28.3, 26.9, 25.8, 25.3, 24.8 (Triaz-CH_2_-), 21.7, 21.0 (Ar-CH_3_), 20.8 (Ar-CH_3_), 12.8 (-CH_3_), 12.6 (-CH_3_); IR (ν, cm^−1^) 3334 (N-H), 3056, 3032, 2925, 2853 (C-H stretching vibrations), 1652, 1646, 1621 (C=O), 1600, 1575, 1526, 1510 (C=C, C=N); HRMS (ESI-TOF) (*m*/*z*) [M + Na]^+^ calcd for [C_40_H_42_N_7_O_3_]^+^ 668.3344, found 668.3356.

*2,9-Bis(ethyl(p-tolyl)carbamoyl)-7-oxo-7,10-dihydro-1,10-phenanthrolin-4-yl acetate* (**14b**). A solution of the diamide (80 mg, 0.15 mmol) in methylene chloride (2 mL) was added to a suspension of potassium carbonate (23 mg, 0.165 mmol) in methylene chloride (2 mL). The resulting mixture was stirred for 15 min, after which acetic anhydride (17 mg, 0.165 mmol) was added. The solution was stirred for 3 h (reaction monitored by TLC, eluent—EtOAc). Afterward, it was washed with water (3 × 5 mL), the organic fraction was dried over sodium sulfate and filtered, and the organic solvent was evaporated. Yield 77 mg (89%), yellowish powder; *R_f_* = 0.6 (EtOAc); mp 132–133 °C; ^1^H NMR (400 MHz, CDCl_3_) δ 10.26 (br s, 1H, NH), 8.15 (d, *J* = 9.0 Hz, 1H, Phen^5,6^), 7.91 (s, 1H, Phen^8^), 7.59 (d, *J* = 9.0 Hz, 1H, Phen^5,6^), 7.15 (t, *J* = 7.9 Hz, 4H), 7.06 (d, *J* = 7.9 Hz, 2H), 7.00 (d, *J* = 7.9 Hz, 2H), 5.93 (s, 1H, Phen^3^), 4.05 (q, *J* = 7.1 Hz, 4H, -N-CH_2_-), 2.46 (s, 3H), 2.32 (s, 3H), 2.22 (s, 3H), 1.25–1.39 (m, 6H); ^13^C NMR (101 MHz, CDCl_3_) δ 177.7, 167.6, 166.5, 163.2, 154.8, 153.7, 140.6, 140.3, 139.1, 139.0, 138.6, 136.9, 136.7, 130.9, 130.2, 128.1, 126.9, 124.3, 123.0, 116.3, 115.6, 115.3, 46.8 (-N-CH_2_-), 45.8 (-N-CH_2_-), 21.2, 21.2, 21.2, 12.9 (-CH_3_), 12.7 (-CH_3_); IR (ν, cm^−1^): 3354 (N-H); 3032, 2976, 2943, 2872 (C-H stretching vibrations); 1773 (C=O^ester^); 1632, 1602 (C=O^amide^); 1559, 1533, 1510 (C=C, C=N); HRMS (ESI-TOF) (*m*/*z*) [M + H]^+^ calcd for [C_34_H_33_N_4_O_5_]^+^ 577.2445, found 577.2451.

*2,9-Bis(ethyl(p-tolyl)carbamoyl)-1,10-phenanthroline-4,7-diyl-bis(4-methylbenzenesulfonate)* (**15b**). 4-oxo-7-hydroxy-phenanthroline diamide (28 mg, 0.05 mmol) was added to a suspension of potassium carbonate (16 mg, 0.11 mmol) in methylene chloride (2 mL). The resulting mixture was stirred at room temperature for 30 min, after which tosyl chloride (21 mg, 0.11 mmol) was added. The resulting solution was stirred for 24 h. After the reaction was complete (monitored by TLC, eluent—EtOAc:Hexane (1:1)), the reaction mixture was washed with water (5 mL). The organic fraction was dried over sodium sulfate and filtered, and the organic solvent was evaporated. The resulting solid residue was further washed with a hexane:ether (1:1) mixture. Yield 30 mg (70%), light-colored powder; *R_f_* = 0.65 (EtOAc:Hexane(1:1)); mp 107–110 °C; ^1^H NMR (600 MHz, C_6_D_6_) δ 8.27 (s, 1H, Phen), 8.21 (d, J = 7.8 Hz, 2H), 7.69–7.63 (m, 2H), 7.56 (d, J = 9.2 Hz, 1H, Phen^5,6^), 7.37 (s, 1H, Phen), 7.31 (d, J = 9.2 Hz, 1H, Phen^5,6^), 7.21 (d, J = 8.0 Hz, 2H), 7.13 (d, J = 7.7 Hz, 2H), 7.07 (d, J = 8.0 Hz, 2H), 6.99 (d, J = 8.0 Hz, 2H), 6.91 (d, J = 7.8 Hz, 2H), 6.71 (d, J = 8.0 Hz, 2H), 3.90 (q, J = 7.2 Hz, 2H, -N-CH_2_-), 3.85 (q, J = 7.0 Hz, 2H, -N-CH_2_-), 2.18 (s, 3H), 1.98 (s, 3H), 1.95 (s, 3H), 1.78 (s, 3H), 1.20–1.11 (m, 6H, -CH_3_); ^13^C NMR (151 MHz, C_6_D_6_) δ 174.9, 165.8, 161.3, 155.8, 154.5, 146.6, 143.4, 142.7, 141.0, 140.5, 140.0, 138.5, 138.3, 137.3, 136.4, 132.7, 131.8, 130.6, 129.1, 129.1, 128.8, 127.2, 127.1, 123.8, 123.3, 121.9, 118.7, 117.3, 117.2, 113.4, 47.2, 46.0, 21.3, 21.1, 21.0, 12.9, 12.6; IR (ν, cm^−1^) 3060, 3036, 2974, 2928, 2872 (C-H stretching vibrations), 1656, 1651 (C=O), 1594, 1573, 1511 (C=C, C=N); HRMS (ESI-TOF) calcd for [C_46_H_42_N_4_NaO_8_S_2_]^+^ 865.2336 found 865.2335.

*N^2^,N^2^,N^9^,N^9^-Tetrabutyl-4-fluoro-7-methoxy-1,10-phenanthroline-2,9-dicarboxamide* (**17a**). Diamide **1a** (37 mg, 0.07 mmol) was added to a suspension of potassium carbonate (29 mg, 0.21 mmol) in chloroform (1 mL); the resulting mixture was stirred for 30 min, after which dimethyl sulfate (33 mg, 0.35 mmol) was added. The resulting mixture was stirred at 60 °C for 6 h, after which it was purified using column chromatography eluent hexane→hexane:ethyl acetate (4:1). Yield 30 mg (80%), white powder. The spectral data are consistent with those described in the literature [26].

*N^2^,N^2^,N^9^,N^9^-Tetrabutyl-4-chloro-7-methoxy-1,10-phenanthroline-2,9-dicarboxamide* (**18a**). Diamide **16a** (162 mg, 0.3 mmol) was added to a suspension of potassium carbonate (124 mg, 0.9 mmol) in chloroform (5 mL). The resulting mixture was stirred for 30 min, after which dimethyl sulfate (114 mg, 0.9 mmol) was added. The resulting mixture was stirred at 60 °C for 5 h, after which it was purified using column chromatography eluent hexane→hexane:ethyl acetate (4:1). Yield 138 mg (83%), white powder; *R_f_* = 0.27 (Hexane:EtOAc (7:3)); mp 102–105 °C; ^1^H NMR (400 MHz, CDCl_3_) δ 8.35 (d, *J* = 9.3 Hz, 1H, Phen^5,6^), 8.23 (d, *J* = 9.3 Hz, 1H, Phen^5,6^), 8.06 (s, 1H), 7.44 (s, 1H), 4.13 (s, 3H, -OCH_3_), 3.75–3.61 (m, 4H, -N-CH_2_-), 3.60–3.49 (m, 4H, -N-CH_2_-), 1.82–1.65 (m, 8H), 1.53–1.39 (m, 4H), 1.13–0.96 (m, 10H), 0.72–0.61 (-CH_3_); ^13^C NMR (101 MHz, CDCl_3_) δ 168.7, 167.6, 163.1, 156.4, 154.3, 145.4, 145.2, 143.4, 127.3, 123.6, 122.2, 121.6, 121.4, 103.6, 56.4, 49.3, 46.8, 31.3, 30.0, 29.9, 20.6, 20.6, 20.1, 14.1, 13.7; IR (ν, cm^−1^) 2956, 2928, 2870 (C-H stretching vibrations) 1629, 1608 (C=O), 1575, 1541 (C=C, C=N); HRMS (ESI-TOF) (*m*/*z*) [M + H]^+^ cacld for [C_31_H_44_ClN_4_O_3_]^+^ 555.3096, found 555.3088.

*N^2^,N^2^,N^9^,N^9^-Tetrabutyl-4-fluoro-7-(prop-2-yn-1-yloxy)-1,10-phenanthroline-2,9-dicarboxamide* (**19a**). 4-oxo-7-fluoro-phenanthroline diamide **1a** (31 mg, 0.06 mmol) was added to a suspension of potassium carbonate (25 mg, 0.18 mmol) in chloroform (1 mL); the resulting mixture was stirred for 30 min, after which propargyl bromide (36 mg, 0.3 mmol). The resulting mixture was stirred at 60 °C for 12 h, after which it was diluted with chloroform (10 mL) and washed with water (10 mL). The organic phase was dried over sodium sulfate and filtered, and the organic solvent was evaporated. Yield 29 mg (85%). The spectral data are consistent with those described in the literature [26].

*N^2^,N^2^,N^9^,N^9^-Tetrabutyl-4-chloro-7-(prop-2-yn-1-yloxy)-1,10-phenanthroline-2,9-dicarboxamide* (**20a**). Diamide **16a** (33 mg, 0.06 mmol) was added to a suspension of potassium carbonate (25 mg, 0.18 mmol) in chloroform (1 mL). The resulting mixture was stirred for 30 min, after which propargyl bromide (36 mg, 0.3 mmol) was added. The resulting mixture was stirred at 60 °C for 18 h, after which it was diluted with chloroform (10 mL) and washed with water (10 mL). The organic phase was dried over sodium sulfate and filtered, and the organic solvent was evaporated. Yield 29 mg (83%), white powder; *R_f_* = 0.45 (Hexane:EtOAc (7:3)); mp 110–113 °C; ^1^H NMR (400 MHz, CDCl_3_) δ 8.37 (d, *J* = 9.3 Hz, 1H, Phen^5,6^), 8.25 (d, *J* = 9.3 Hz, 1H, Phen^5,6^), 8.08 (s, 1H), 7.54 (s, 1H), 5.03 (d, *J* = 2.5 Hz, 2H, -O-CH_2_-), 3.71–3.61 (m, 4H, -N-CH_2_-), 3.60–3.51 (m, 4H, -N-CH_2_-), 2.64 (t, *J* = 2.4 Hz, 1H, -CH), 1.78–1.67 (m, 8H), 1.52–1.39 (m, 4H), 1.12–0.95 (m, 10H), 0.72–0.61 (m, 6H); ^13^C NMR (101 MHz, CDCl_3_) δ 168.5, 167.6, 161.0, 156.2, 154.4, 145.5, 143.5, 127.3, 123.7, 122.1, 121.9, 121.4, 104.7, 77.3, 56.8, 49.3, 46.8, 31.3, 30.0, 29.9, 20.6, 20.1, 20.1, 14.2, 13.8, 13.8; IR (ν, cm^−1^) 3210 (≡C-H stretching vibrations), 2958, 2928, 2871 (C-H stretching vibrations), 2120 (-C≡C-), 1639, 1620 (C=O), 1576, 1540 (C=C, C=N); HRMS (ESI-TOF) (*m*/*z*) [M + H]^+^ cacld for [C_33_H_44_ClN_4_O_3_]^+^ 579.3096, found 579.3088.

*2,9-Bis(dibutylcarbamoyl)-7-fluoro-1,10-phenanthrolin-4-yl acetate* (**21a**). Diamide **1a** (31 mg, 0.06 mmol) was added to a suspension of potassium carbonate (21 mg, 0.15 mmol) in chloroform (1 mL); the resulting mixture was stirred for 30 min, after which acetic anhydride (25 mg, 0.24 mmol) was added. The resulting mixture was stirred at room temperature for 2 days (control by TLC, eluent Hexane:EtOAc (7:3)), after which it was diluted with chloroform (10 mL) and washed with an aqueous solution of potassium carbonate (10 mL). The organic phase was dried over sodium sulfate and filtered, and the organic solvent was evaporated. Yield 25 mg (74%), white powder; *R_f_* = 0.4 (Hexane:EtOAc (7:3)); mp 94–96 °C; ^1^H NMR (400 MHz, CDCl_3_) δ 8.14 (d, *J* = 9.2 Hz, 1H, Phen^5^), 8.03 (d, *J* = 9.2 Hz, 1H, Phen^6^), 7.91 (s, 1H, Phen^3^), 7.75 (d, *J* = 9.8 Hz, 1H, Phen^8^), 3.71–3.62 (m, 2H, -N-CH_2_-), 3.61–3.51 (m, 6H, -N-CH_2_-), 2.53 (s, 3H, -COCH_3_), 1.79–1.71 (m, 8H, -CH_2_-), 1.52–1.40 (m, 4H, -CH_2_-), 1.14–1.03 (m, 4H, -CH_2_-), 1.04–0.98 (m, 6H, -CH_3_), 0.73–0.65 (m, 6H, -CH_3_). ^13^C NMR (101 MHz, CDCl_3_) δ 167.8 (-O-C=O), 167.6 (C^2^=O), 167.3 (d, *J* = 3.5 Hz, C^9^=O), 165.7 (d, *J* = 267.4 Hz, C^7^), 156.7 (d, *J* = 7.3 Hz, C^9^), 155.9 (C^2^), 154.8 (C^4^), 146.5 (d, *J* = 3.8 Hz, C^1′^), 145.7 (d, *J* = 4.2 Hz, C^10′^), 123.0 (C^4′^), 120.7 (d, *J* = 1.4 Hz, C^5^), 119.7 (d, *J* = 13.2 Hz, C^6′^), 119.4 (d, *J* = 5.9 Hz, C^6^), 116.1 (C^3^), 109.1 (d, *J* = 16.3 Hz, C^8^), 49.2 (-N-CH_2_-), 49.1 (-N-CH_2_-), 46.7 (-N-CH_2_-), 46.5 (-N-CH_2_-), 31.2 (-CH_2_-), 31.1 (-CH_2_-), 29.8 (-CH_2_-), 21.1 (-COCH_3_), 20.4 (-CH_2_-), 20.0 (-CH_2_-), 14.0 (-CH_3_), 13.6 (-CH_3_), 13.6 (-CH_3_); IR (ν, cm^−1^) 2953, 2929, 2862 (C-H stretching vibrations), 1782 (C=O^ester^), 1637, 1625, 1615 (C=O^amide^), 1544, 1474, 1455 (C=C, C=N); HRMS (ESI-TOF) (*m*/*z*) [M + H]^+^ cacld for [C_32_H_44_FN_4_O_4_]^+^ 567.3341, found 567.3332.

*4-(Benzyloxy)-N^2^,N^2^,N^9^,N^9^-tetrabutyl-7-chloro-1,10-phenanthroline-2,9-dicarboxamide* (**22a**). Diamide **16a** (33 mg, 0.06 mmol) was added to the suspension of potassium carbonate (25 mg, 0.18 mmol) in chloroform (1 mL); the resulting mixture was stirred for 30 min, after which benzyl bromide (51 mg, 0.3 mmol) was added. The resulting mixture was stirred at 60 °C for 12 h, after which it was diluted with chloroform (10 mL) and washed with water (10 mL). The organic phase was dried over sodium sulfate and filtered, and the organic solvent was evaporated. Yield 31 mg (87%), yellowish powder; *R_f_* = 0.52 (Hexane:EtOAc (7:3)); mp 90–93 °C; ^1^H NMR (400 MHz, CDCl_3_) δ 8.42 (d, *J* = 9.3 Hz, 1H, Phen^5,6^), 8.25 (d, *J* = 9.3 Hz, 1H, Phen^5,6^), 8.08 (s, 1H, Phen^3,8^), 7.60–7.52 (m, 3H, Ar-H + Phen^3,8^), 7.51–7.38 (m, 3H, Ar-H), 5.39 (s, 2H, -O-CH_2_-), 3.73–3.62 (m, 4H, -N-CH_2_-), 3.62–3.47 (m, 4H, -N-CH_2_-), 1.83–1.66 (m, 8H), 1.53–1.40 (m, 4H), 1.13–0.95 (m, 10H), 0.74–0.57 (m, 6H, -CH_3_). ^13^C NMR (101 MHz, CDCl_3_) δ 168.6, 167.6, 162.1, 156.4, 154.3, 145.4, 143.4, 135.4, 129.0, 128.8, 127.9, 127.3, 123.6, 122.4, 121.7, 121.5, 104.6, 71.0 (-O-CH_2_-), 49.3 (-N-CH_2_-), 46.8, (-N-CH_2_-) 31.3, 31.3, 30.0, 29.9, 20.6, 20.6, 20.1, 20.1, 14.2, 14.1, 13.7; IR (ν, cm^−1^) 2953, 2927, 2868 (C-H stretching vibrations), 1632, 1611 (C=O), 1574, 1542 (C=C, C=N); HRMS (ESI-TOF) (*m*/*z*) [M + H]^+^ cacld for [C_37_H_48_ClN_4_O_3_]^+^ 631.3409, found 631.3397.

*4-(Allyloxy)-N^2^,N^2^,N^9^,N^9^-tetrabutyl-7-chloro-1,10-phenanthroline-2,9-dicarboxamide* (**23a**). Diamide **16a** (54 mg, 0.1 mmol) was added to a suspension of potassium carbonate (41 mg, 0.3 mmol) in chloroform (1 mL); the resulting mixture was stirred for 30 min, after which allyl bromide (60 mg, 0.5 mmol) was added. The resulting mixture was stirred at 60 °C for 12 h, after which it was diluted with chloroform (10 mL) and washed with water (10 mL). The organic phase was dried over sodium sulfate and filtered, and the organic solvent was evaporated. Yield 51 mg (88%), white powder; *R_f_* = 0.37 (Hexane:EtOAc (7:3)); mp 111–115 °C; ^1^H NMR (400 MHz, CDCl_3_) δ 8.41 (d, *J* = 9.3 Hz, 1H, H^5,6^), 8.25 (d, *J* = 9.3 Hz, 1H, H^5,6^), 8.08 (s, 1H), 7.43 (s, 1H), 6.27–6.09 (m, 1H, =CH), 5.62–5.50 (m, 1H, =CH_2_), 5.47–5.37 (m, 1H, =CH_2_), 4.92–4.81 (m, 2H, -O-CH_2_-), 3.73–3.62 (m, 4H, -N-CH_2_-), 3.61–3.52 (m, 4H, -N-CH_2_-), 1.81–1.61 (m, 8H), 1.54–1.38 (m, 4H), 1.14–0.95 (m, 10H), 0.68 (q, *J* = 7.1 Hz, 6H, -CH_3_). ^13^C NMR (101 MHz, CDCl_3_) δ 168.7 (C=O), 167.6 (C=O), 162.0, 156.4, 154.3, 145.4, 143.4, 131.7, 127.3, 123.6, 122.3, 121.6, 121.5, 119.1, 104.5, 69.7 (-O-CH_2_-), 49.3 (-N-CH_2_-), 46.8 (-N-CH_2_-), 31.3, 30.0, 29.9, 20.6, 20.6, 20.1, 14.2, 13.8, 13.7; IR (ν, cm^−1^) 2959, 2930, 2862 (C-H stretching vibrations), 1636, 1626, 1610 (C=O), 1572, 1540 (C=C, C=N); HRMS (ESI-TOF) (*m*/*z*) [M + H]^+^ cacld for [C_33_H_46_ClN_4_O_3_]^+^ 581.3253, found 581.3246.

*N^2^,N^2^,N^9^,N^9^-Tetrabutyl-4-fluoro-7-methoxy-1,10-phenanthroline-2,9-dicarboxamide lanthanum trinitrate* (**17a•**La(NO_3_)_3_). A solution of lathanum nitrate hexahydrate (22 mg, 0.05 mmol) in acetonitrile (0.5 mL) was added dropwise to a solution of 1,10-phenanthroline-2,9-dicarboxamide **17a** (27 mg, 0.05 mmol) in acetonitrile (0.5 mL). The reaction mixture was concentrated in vacuo to 1/10 of its initial volume and then treated with diethyl ether (2 mL). The resulting complex was filtered, washed with ether and dried in air to constant mass. Yield 40 mg (93%), white powder; T_decomp_ 260 °C; ^1^H NMR (400 MHz, CD_3_CN) δ 8.45 (d, *J* = 9.3 Hz, 1H, H^6^), 8.26 (d, *J* = 9.3 Hz, 1H, H^5^), 7.94 (d, *J* = 9.4 Hz, 1H, H^3^), 7.60 (s, 1H, H^8^), 4.26 (s, 3H, -OCH_3_), 3.87–3.71 (m, 4H, -N-CH_2_-), 3.70–3.51 (m, 4H, -N-CH_2_-), 1.89–1.67 (m, 8H), 1.53–1.38 (m, 4H), 1.38–1.28 (m, 4H), 1.05–0.95 (m, 6H, -CH_3_), 0.94–0.85 (m, 6H, -CH_3_); ^13^C NMR (101 MHz, CD_3_CN) δ 170.3 (C^9^=O), 169.2 (d, *J* = 3.5 Hz, C^2^=O), 167.7 (d, *J* = 273.6 Hz, C^4^), 166.0 (C^7^), 153.6 (C^9^), 153.1 (d, *J* = 9.0 Hz, C^2^), 147.6 (d, *J* = 3.8 Hz, C^1′^), 145.7 (d, *J* = 4.2 Hz, C^10′^), 124.2 (C^6^), 122.3 (C^6′^), 122.0 (d, *J* = 14.9 Hz, C^4′^), 120.4 (d, *J* = 4.5 Hz, C^5^), 111.4 (d, *J* = 20.1 Hz, C^3^), 106.2 (C^8^), 58.5 (-OCH_3_), 51.2 (-N-CH_2_-), 50.9 (-N-CH_2_-), 49.1 (-N-CH_2_-), 49.0 (-N-CH_2_-), 31.4 (-CH_2_-), 31.1 (-CH_2_-), 29.6 (-CH_2_-), 29.5 (-CH_2_-), 21.1 (-CH_2_-), 21.0 (-CH_2_-), 20.5 (-CH_2_-), 20.4 (-CH_2_-), 14.1 (-CH_3_), 14.1 (-CH_3_), 14.0 (-CH_3_), 13.8 (-CH_3_). ^19^F NMR (376 MHz, CD_3_CN) δ -102.9 (d, *J* = 9.4 Hz). IR (ν, cm^−1^): 3068, 2961, 2933, 2872 (C-H stretching vibrations), 1593 (C=O), 1581, 1559 (C=C, C=N), 1468, 1445, 1300, 1265 (NO_3_); HRMS (ESI-TOF) (*m*/*z*) [L•La(NO_3_)_2_]^+^ cacld for [C_31_H_43_FN_6_O_9_La]^+^ 801.2134, found 801.2124.

## 4. Conclusions

In this work, we used 4-oxo-7-halo-substituted phenanthroline diamides as starting compounds for the synthesis of 4,7-unsymmetrically substituted DAPhen. We obtained the corresponding reaction products of 4-oxo-7-fluoro-DAPhen with C-, O-, and N-nucleophiles of various natures, and also demonstrated the possibility of the targeted introduction of substituents, leading to various electronic effects. We also demonstrated the possibility of further functionalization of the obtained ligands using the example of the corresponding azido- and hydroxy-substituted DAPhen. In some cases, the yields in the realized transformations at the 7-position of 4-oxo-substituted DAPhen exceed 90% and do not affect the 4-oxo group. We also studied the tautomeric equilibria in 4-oxo-substituted DAPhen containing various functional groups at the 7-position and showed that, regardless of the electronic effects of the substituent, the most stable tautomeric form is the oxo-form. For 4-oxo-7-hydroxy-, 4-oxo-7-chloro- and 4-oxo-7-fluoro-, we demonstrated the possibility of the reaction proceeding via the 4-oxo group under mild conditions, which also enables the synthesis of 4-alkoxy- and 4-acetoxy-substituted DAPhen and opens the path to unsymmetric ligands. Yields in alkylation reactions reach 85–88% and, in some cases, allow for the synthesis of such alkoxy-substituted ligands without the use of column chromatography. Thus, we demonstrated that 4-oxo-7-halosubstituted DAPhen’s may be considered as bifunctional reagents to obtain highly furnished unsymmetrical polydentate ligands with a wide range of variable physico-chemical characteristics.

## Data Availability

The original contributions presented in this study are included in the article/Supplementary Materials. Further inquiries can be directed to the corresponding author.

## Supplementary Materials

The following supporting information can be downloaded at: https://www.mdpi.com/article/10.3390/ijms27177907/s1.
