# Supplementary material for: 4-Oxo-7-Halosubstituted Phenanthroline Diamides Bifunctional Reactivity: A Synthetic Pathway to Unsymmetrical Polydentate Ligands
Source: Int J Mol Sci. 2026 Sep 4;27(17):7907. doi: 10.3390/ijms27177907 (PMC13566864; doi:10.3390/ijms27177907)
Supplement: Supplementary file 1 [file ijms-27-07907-s001.zip › ijms-4519735-supplementary.pdf]

Electronic Supplementary Information

4-Oxo-7-halosubstituted phenanthroline diamides bifunctional reactivity: a synthetic pathway to unsymmetrical polydentate ligands

Roman V. Zonov,[1] Nane A. Avagyan,[1] Pavel S. Lemport,[1] Vitaly A. Roznyatovsky,[1] Victor N. Khrustalev,[2,3] Yulia V. Nelyubina,[4] Yuri A. Ustynyuk,[1] Valentine G. Nenajdenko[1,*]

[1] Department of Chemistry, Lomonosov Moscow State University, Leninskie Gory 1 bld. 3, 119991 Moscow, Russia

[2] Peoples' Friendship University of Russia (RUDN University), Miklukho-Maklay Str. 6, 117198 Moscow, Russia

[3] Zelinsky Institute of Organic Chemistry, Russian Academy of Sciences, Leninsky Prospect 47, 119991 Moscow, Russia

[4] Advanced Engineering School, ITMO University, 9 Lomonosova str., 191002 St. Petersburg, Russia

* Correspondence to: nenajdenko@gmail.com

Table of contents

1. NMR and IR spectra of synthesized compounds……………………………………..…....……S2-S74

2. XRD data…………………………………………………………………..…....…………… S75

3. Computational data…………………………………………………………..……..…. S76- S83

4. Cartesian coordinates, number of imaginary frequencies, and energetic parameters…... S84- S134

1. NMR and IR spectra of synthesized compounds

7-Amino-N2,N2,N9,N9-tetrabutyl-4-oxo-1,4-dihydro-1,10-phenanthroline-2,9-dicarboxamide (2a)

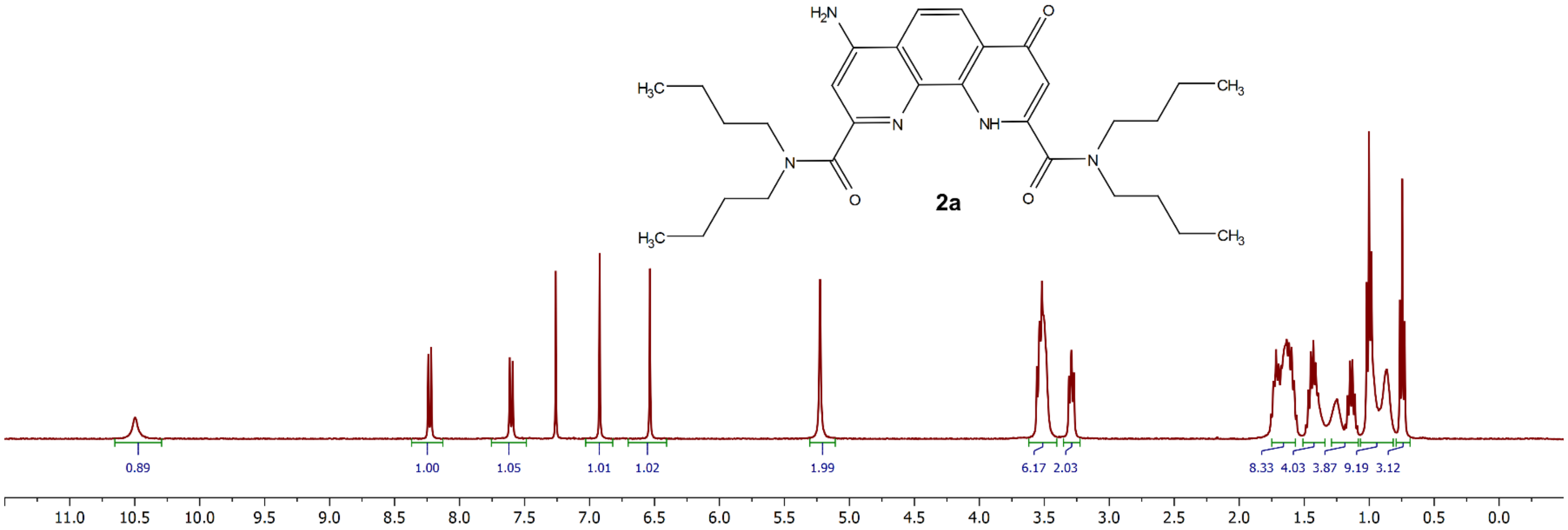

Figure S1. ^{1}H NMR spectrum of **2a** (400 MHz, $CDCl_3$)

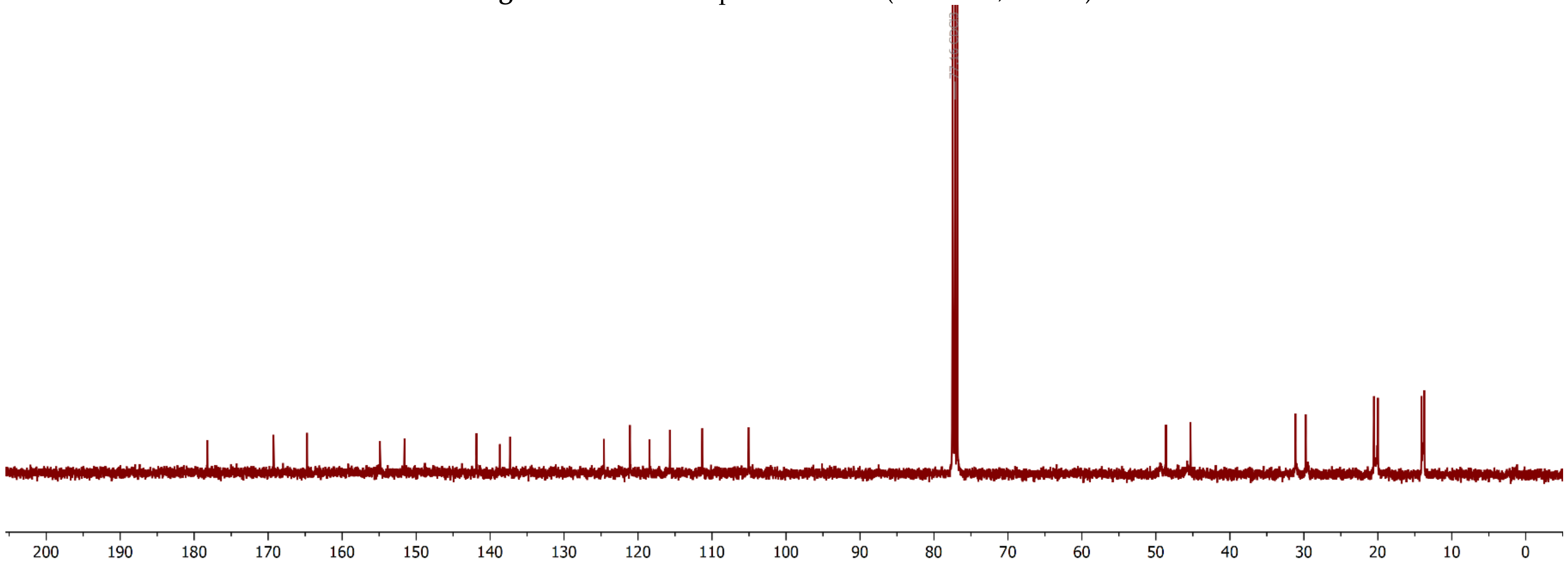

Figure S2. ^{13}C{^{1}H} NMR spectrum of **2a** (101 MHz, $CDCl_3$)

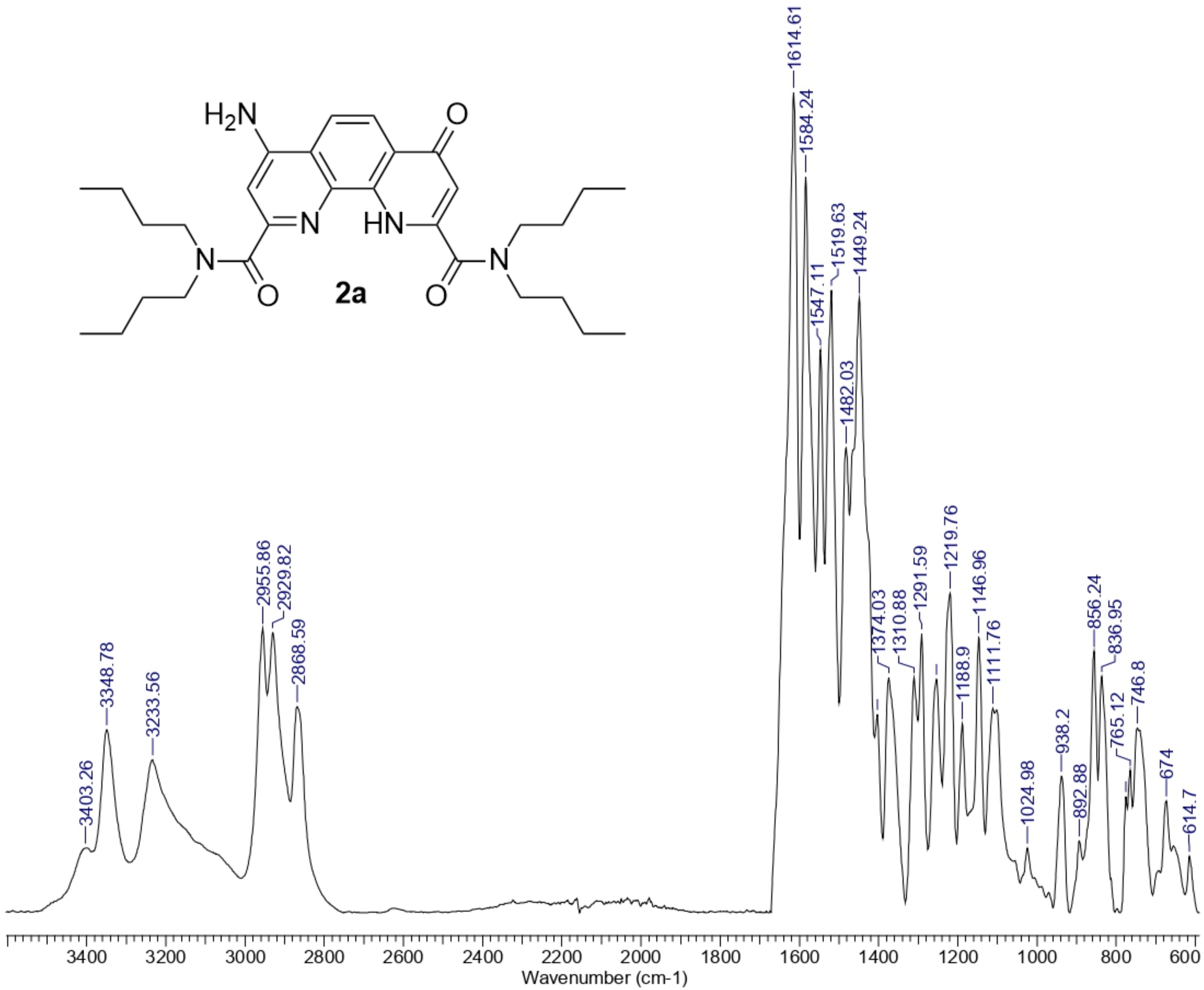

Figure S3. Solid-state IR spectrum of **2a**

7-Amino-N²,N⁹-diethyl-4-oxo-N²,N⁹-di-p-tolyl-1,4-dihydro-1,10-phenanthroline-2,9-dicarboxamide (2b)

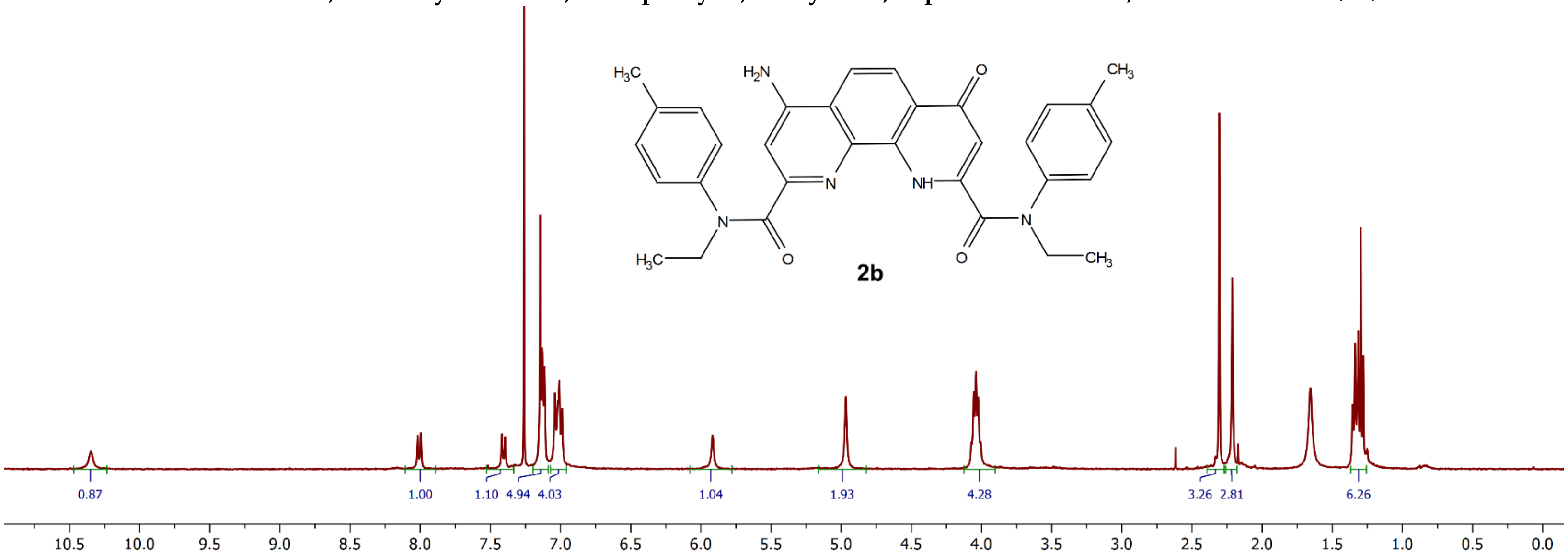

Figure S4. ^{1}H NMR spectrum of **2b** (400 MHz, $CDCl_3$)

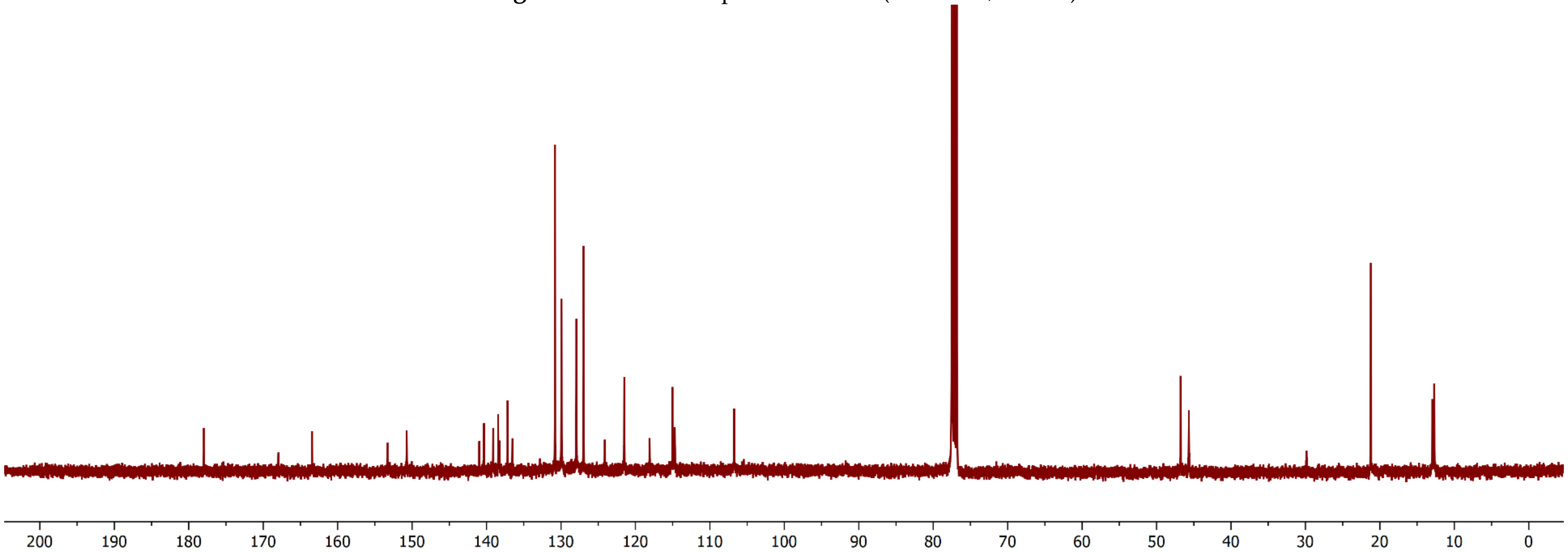

Figure S5. $^{13}C\{^{1}H\}$ NMR spectrum of **2b** (101 MHz, $CDCl_3$)

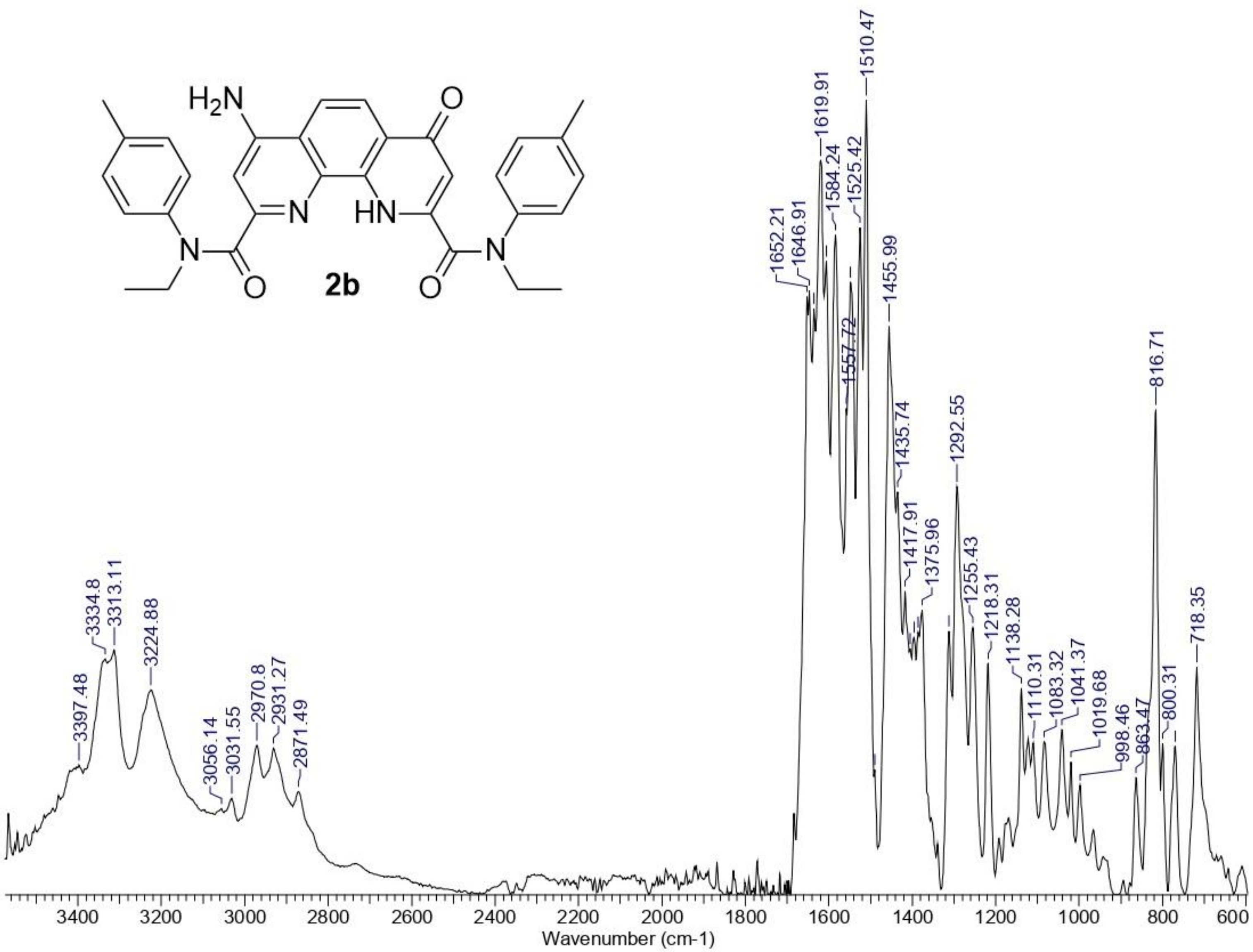

Figure S6. Solid-state IR spectrum of **2b**

N2,N2,N9,N9-Tetrabutyl-4-oxo-7-(pyrrolidin-1-yl)-1,4-dihydro-1,10-phenanthroline-2,9-dicarboxamide (3a)

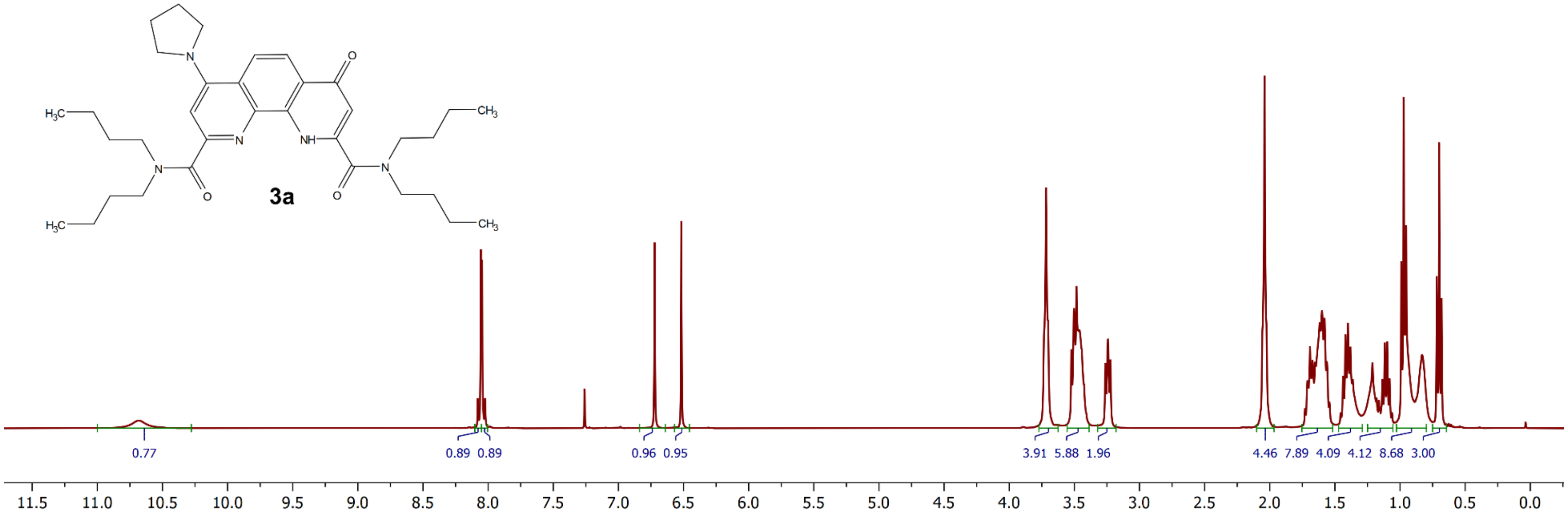

Figure S7. ^{1}H NMR spectrum of **3a** (400 MHz, $CDCl_3$)

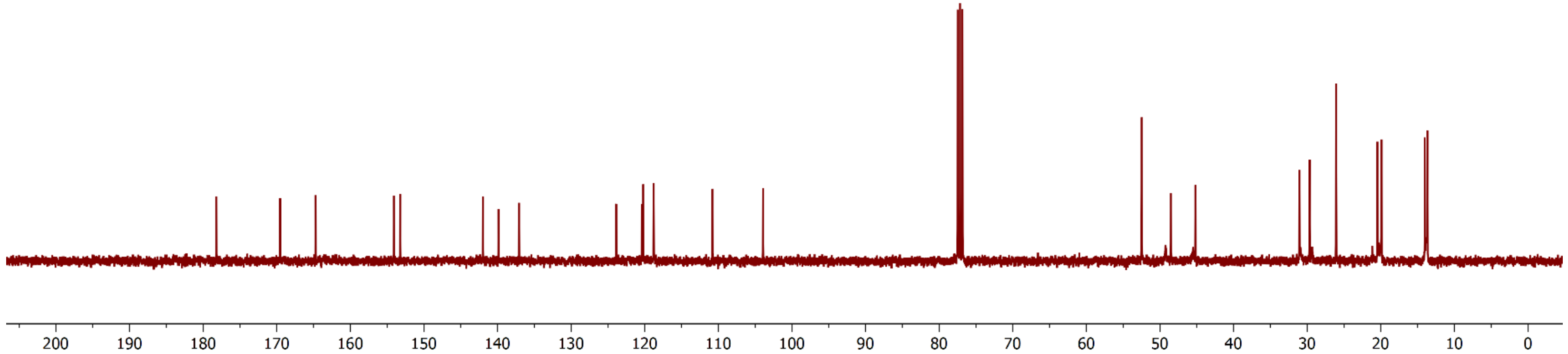

Figure S8. $^{13}C\{^{1}H\}$ NMR spectrum of **3a** (101 MHz, $CDCl_3$)

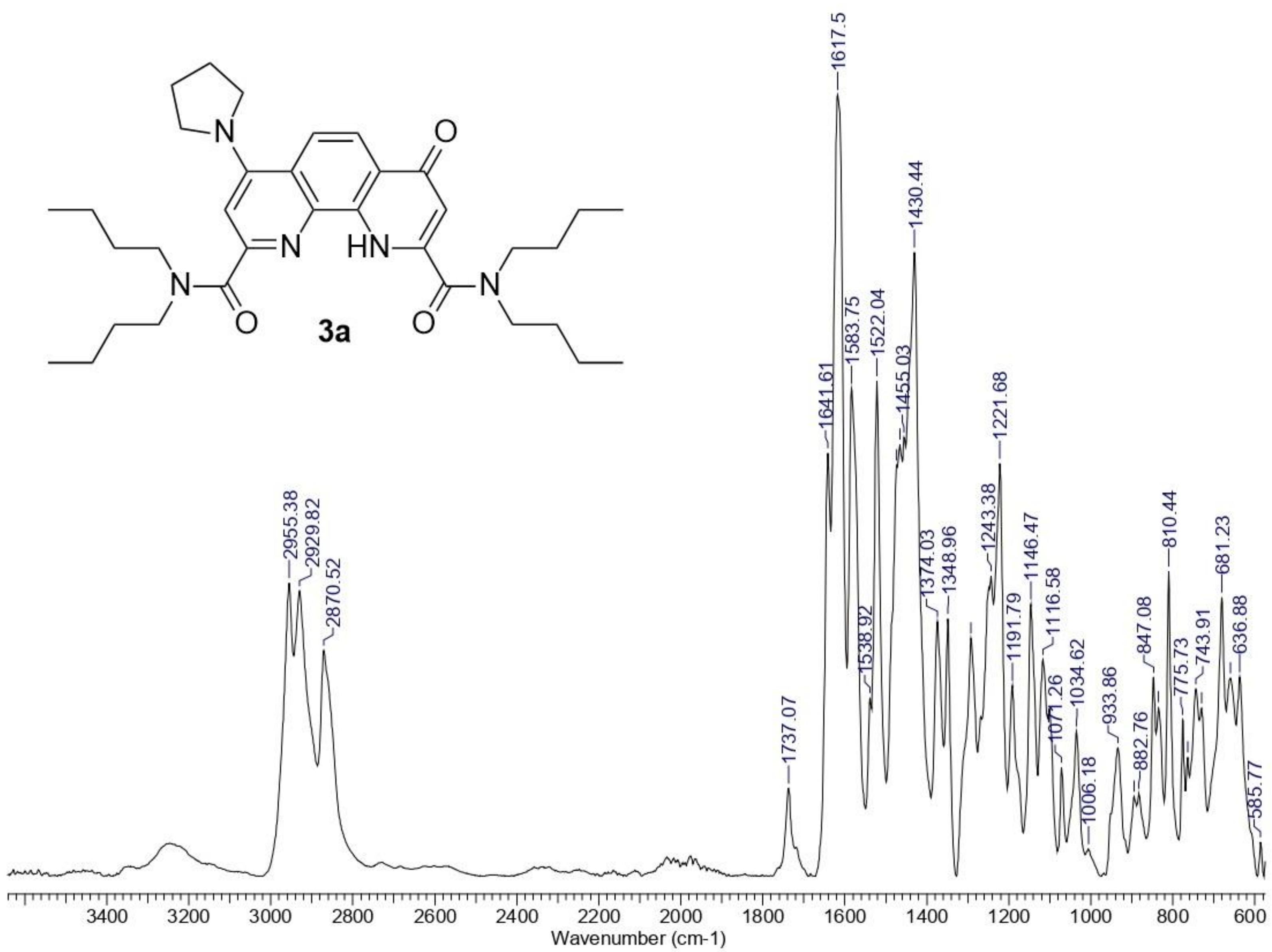

Figure S9. Solid-state IR spectrum of **3a**

N^2,N^9-Diethyl-4-oxo-7-(pyrrolidin-1-yl)-N^2,N^9-di-p-tolyl-1,4-dihydro-1,10-phenanthroline-2,9-dicarboxamide (3b)

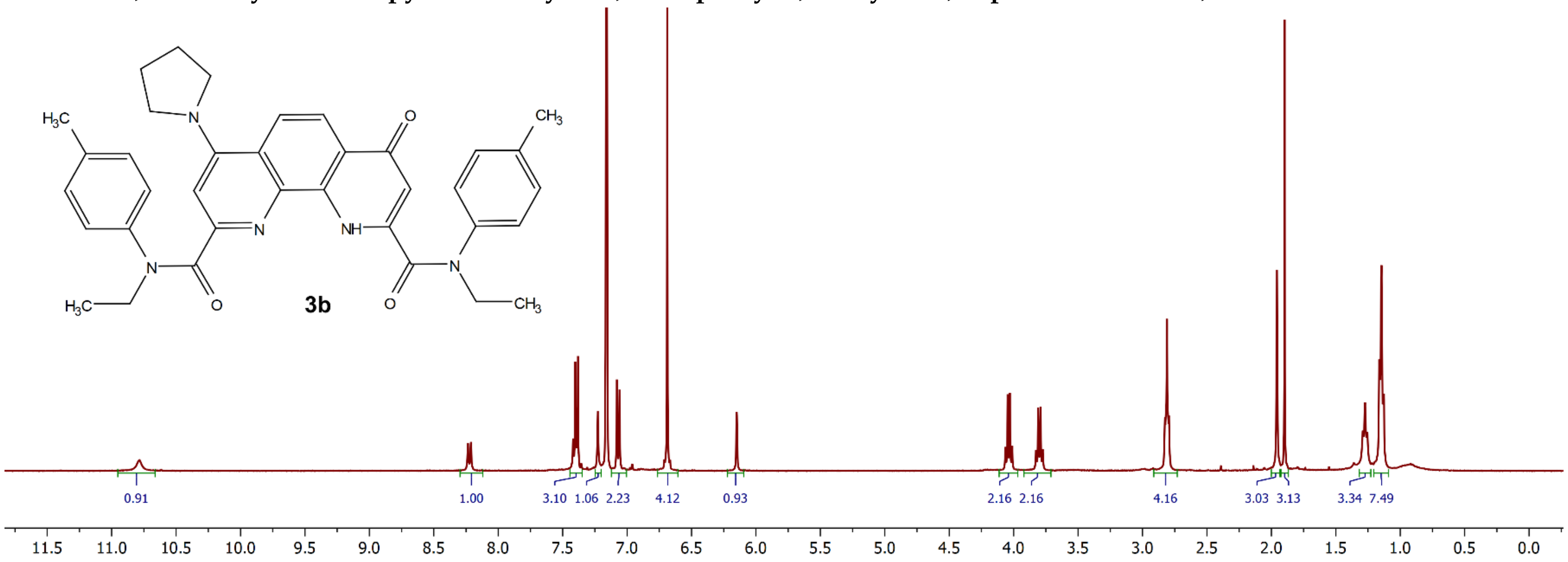

Figure S10. ^{1}H NMR spectrum of **3b** (400 MHz, C_6D_6)

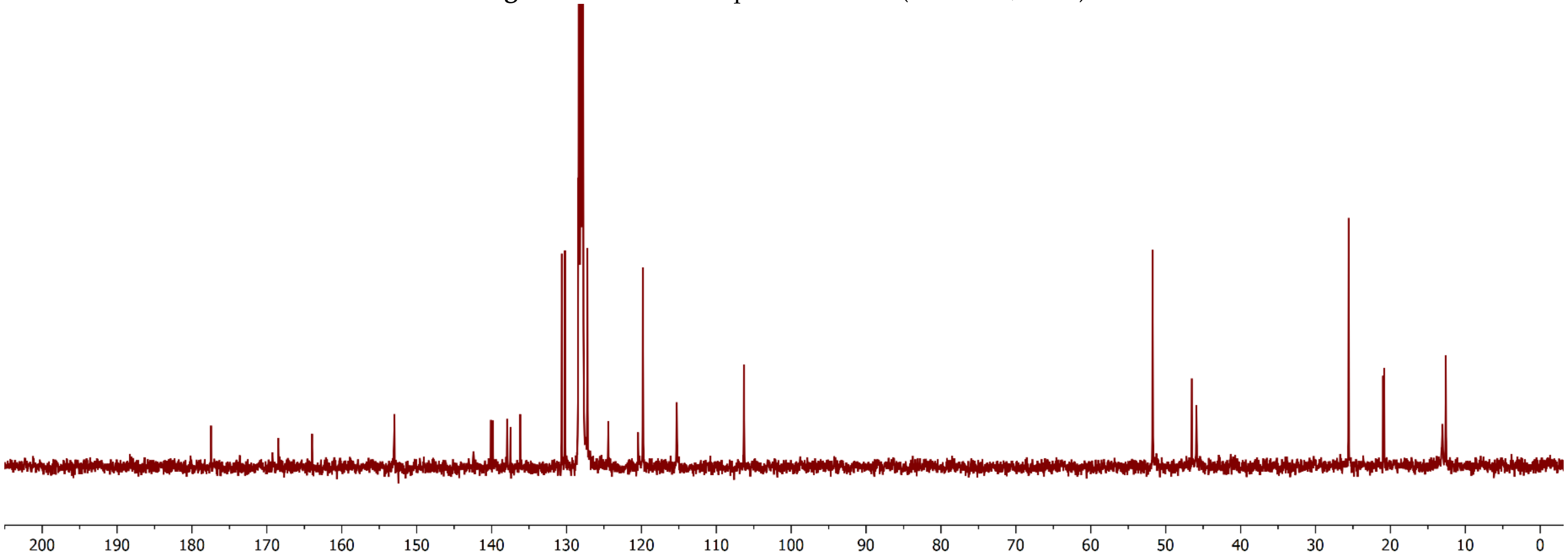

Figure S11. ^{13}C{^{1}H} NMR spectrum of **3b** (101 MHz, C_6D_6)

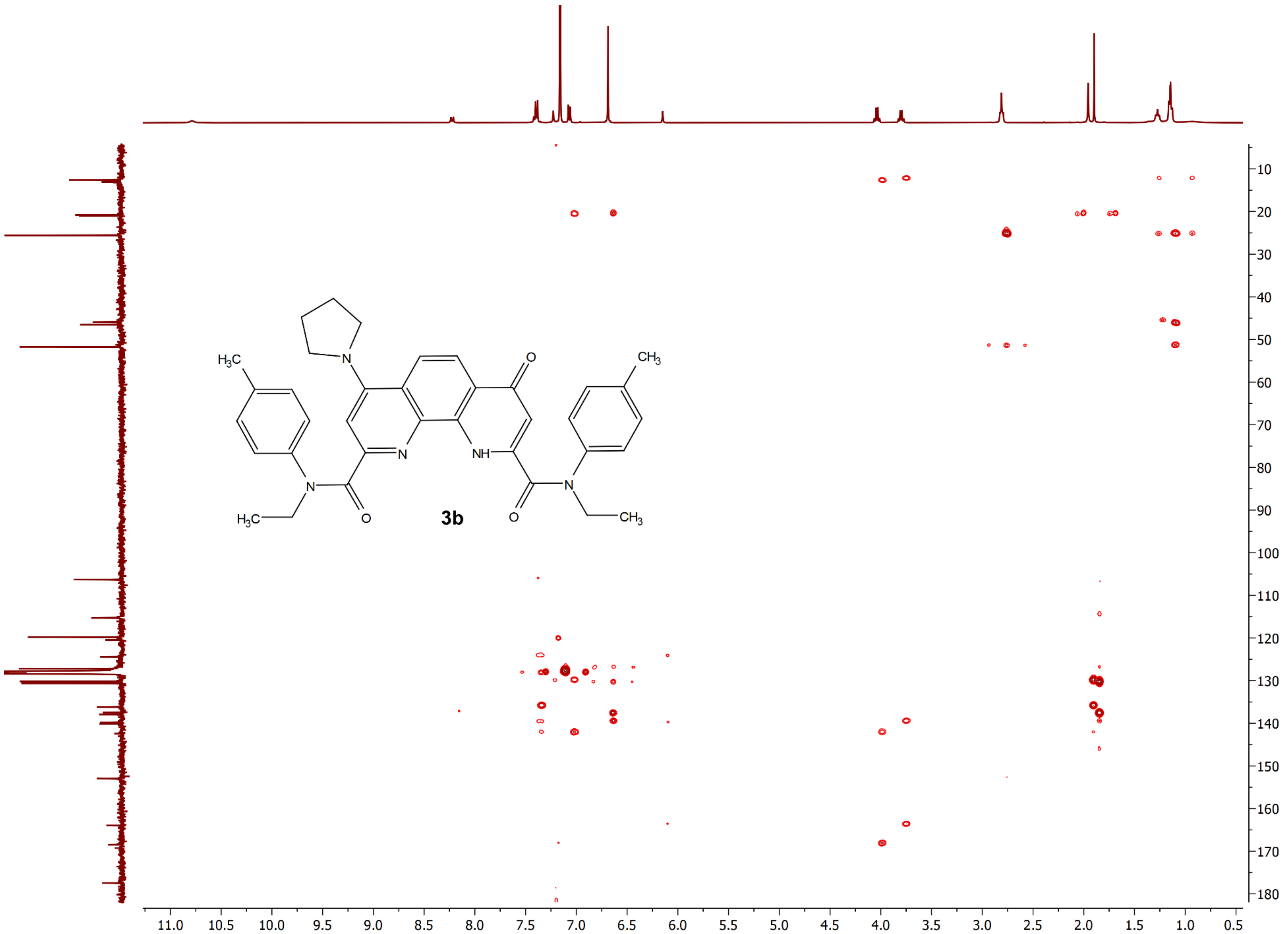

Figure S12. $^{1}H/^{13}C$ HMBC NMR spectrum of **3b** (C_6D_6)

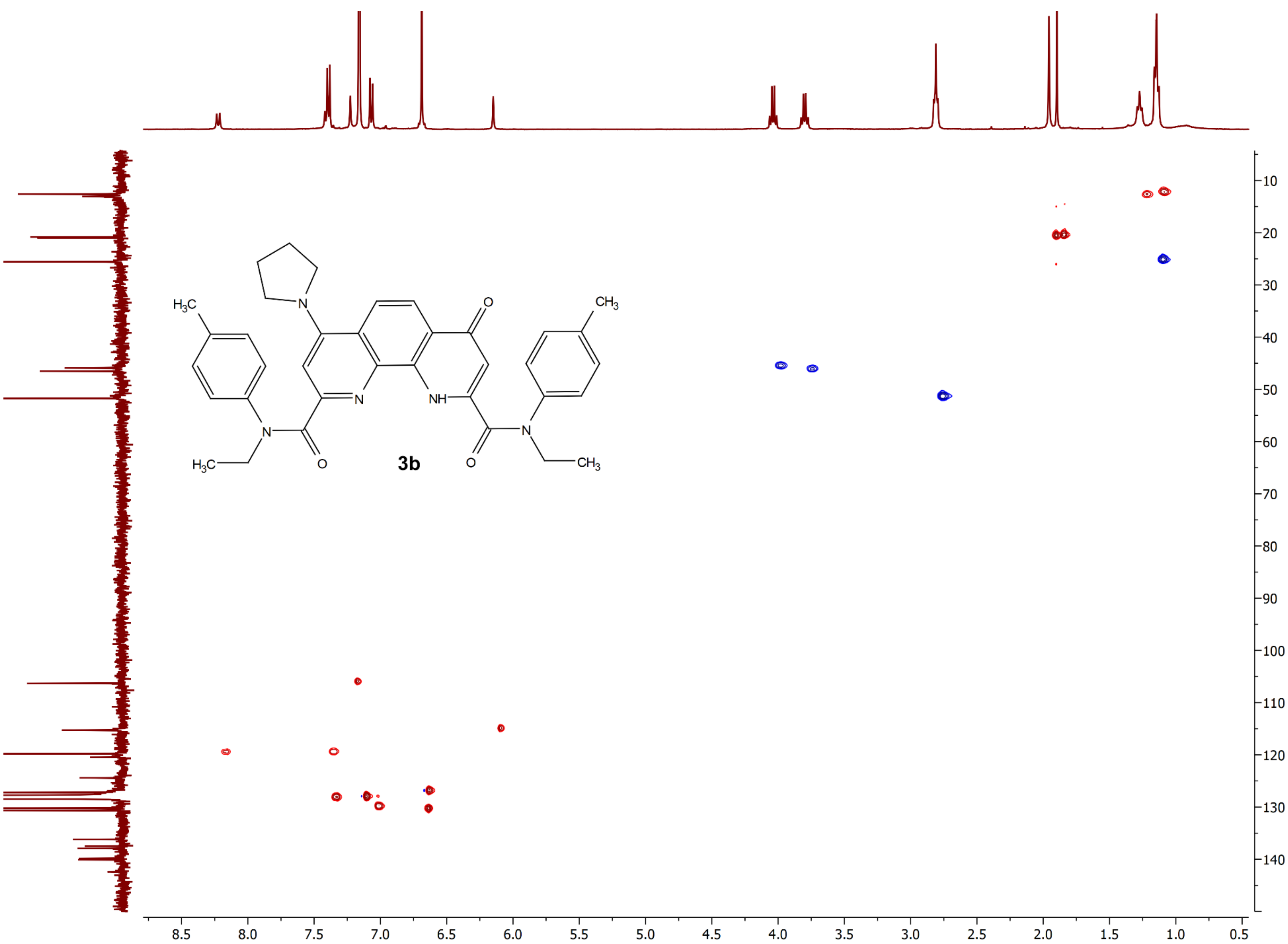

Figure S13. $^{1}H/^{13}C$ HSQC NMR spectrum of **3b** (C_6D_6)

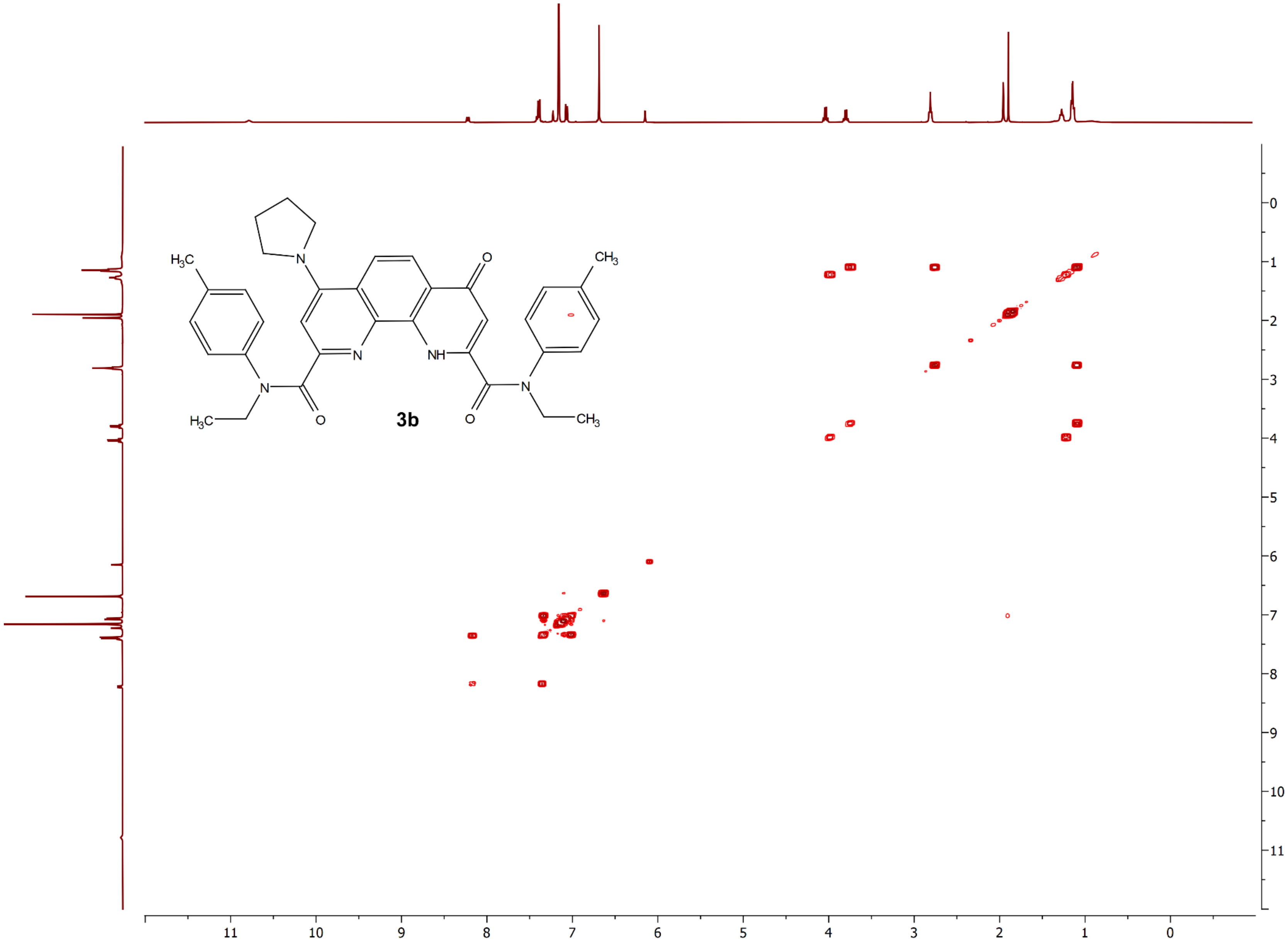

Figure S14. $^{1}H/^{1}H$ COSY NMR spectrum of **3b** (C_6D_6)

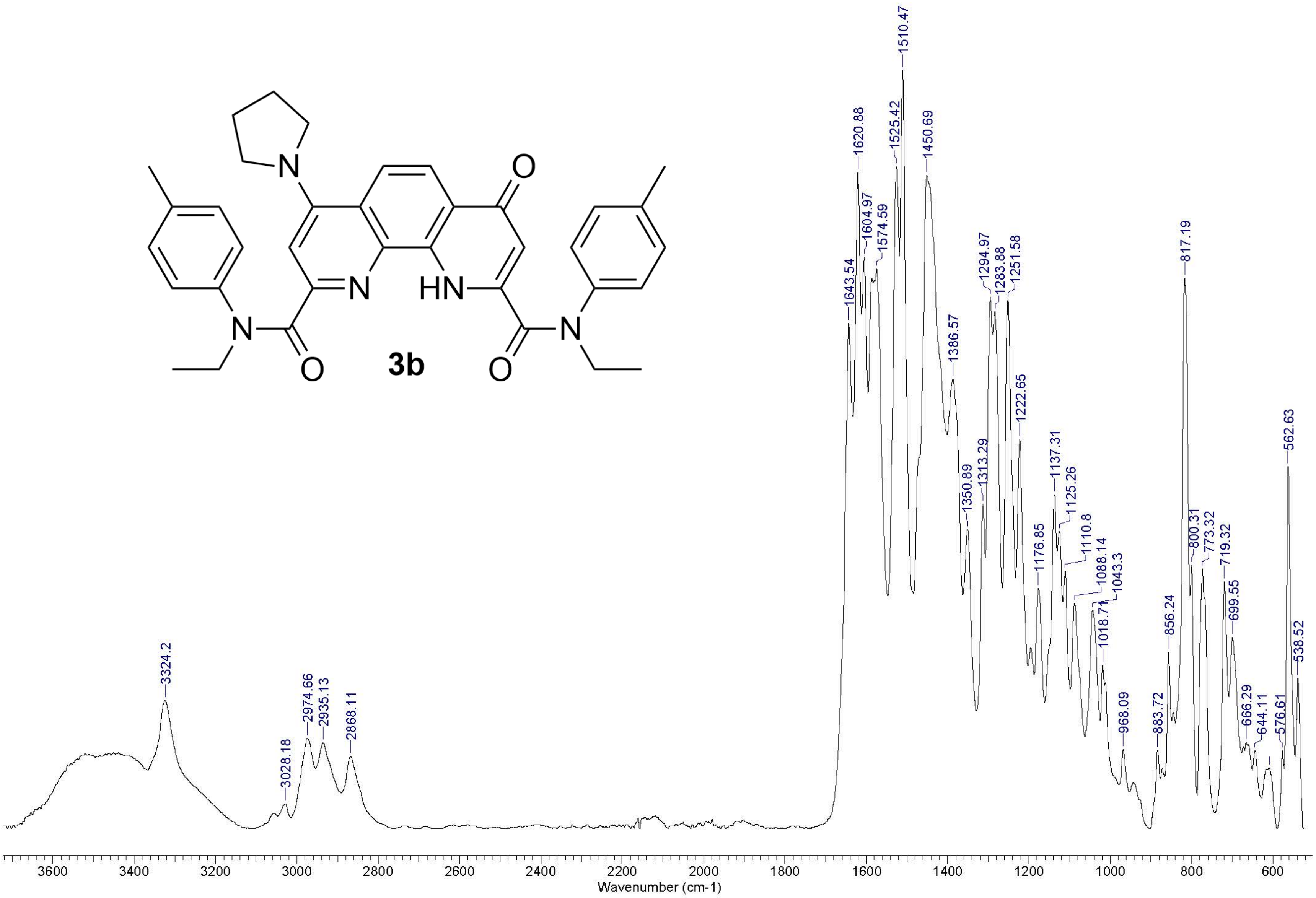

Figure S15. Solid-state IR spectrum of **3a**

N^2,N^2,N^9,N^9-Tetrabutyl-7-cyano-4-oxo-1,4-dihydro-1,10-phenanthroline-2,9-dicarboxamide (4a)

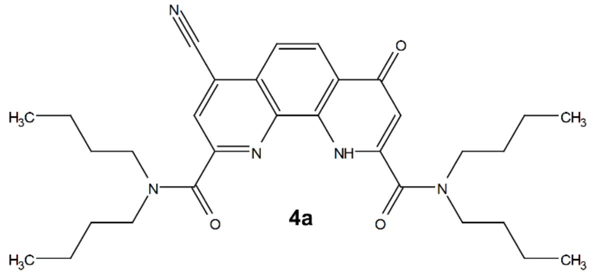

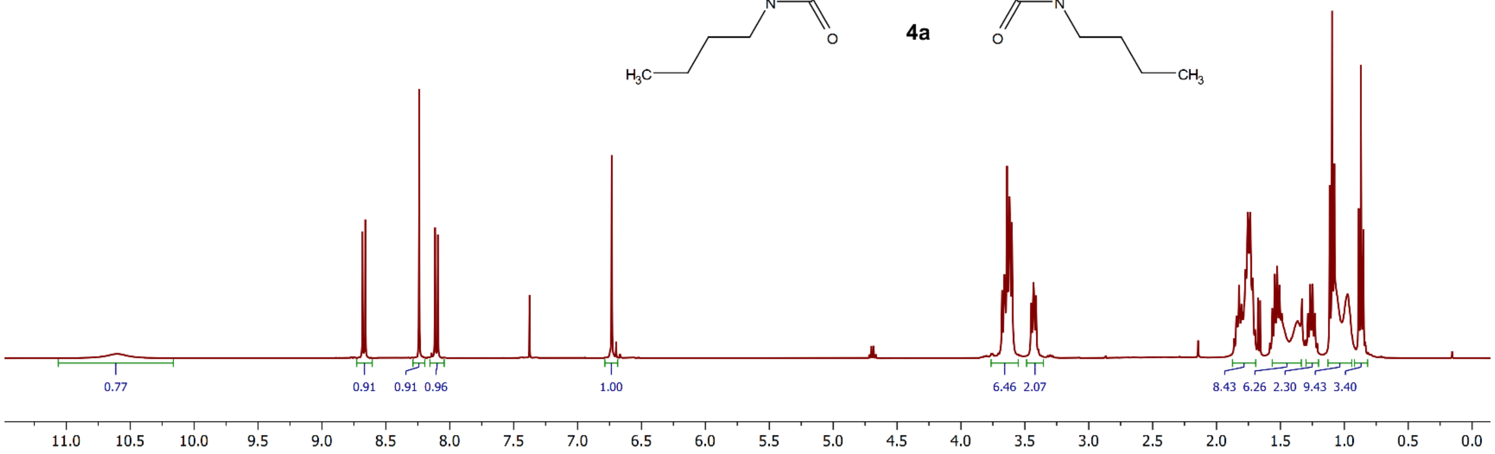

Figure S16. ^{1}H NMR spectrum of **4a** (400 MHz, $CDCl_3$)

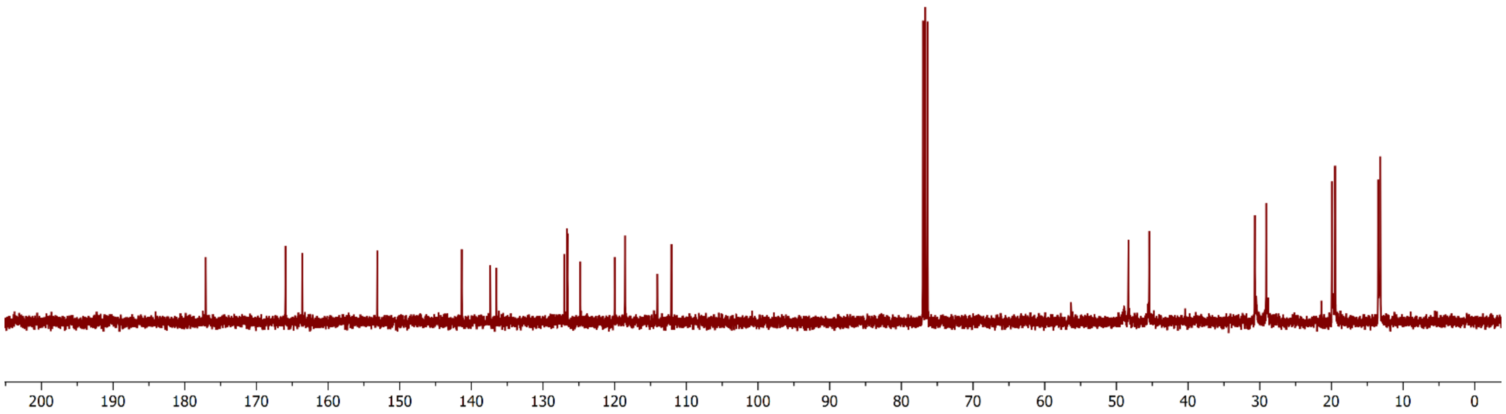

Figure S17. $^{13}C\{^1H\}$ NMR spectrum of **4a** (101 MHz, $CDCl_3$)

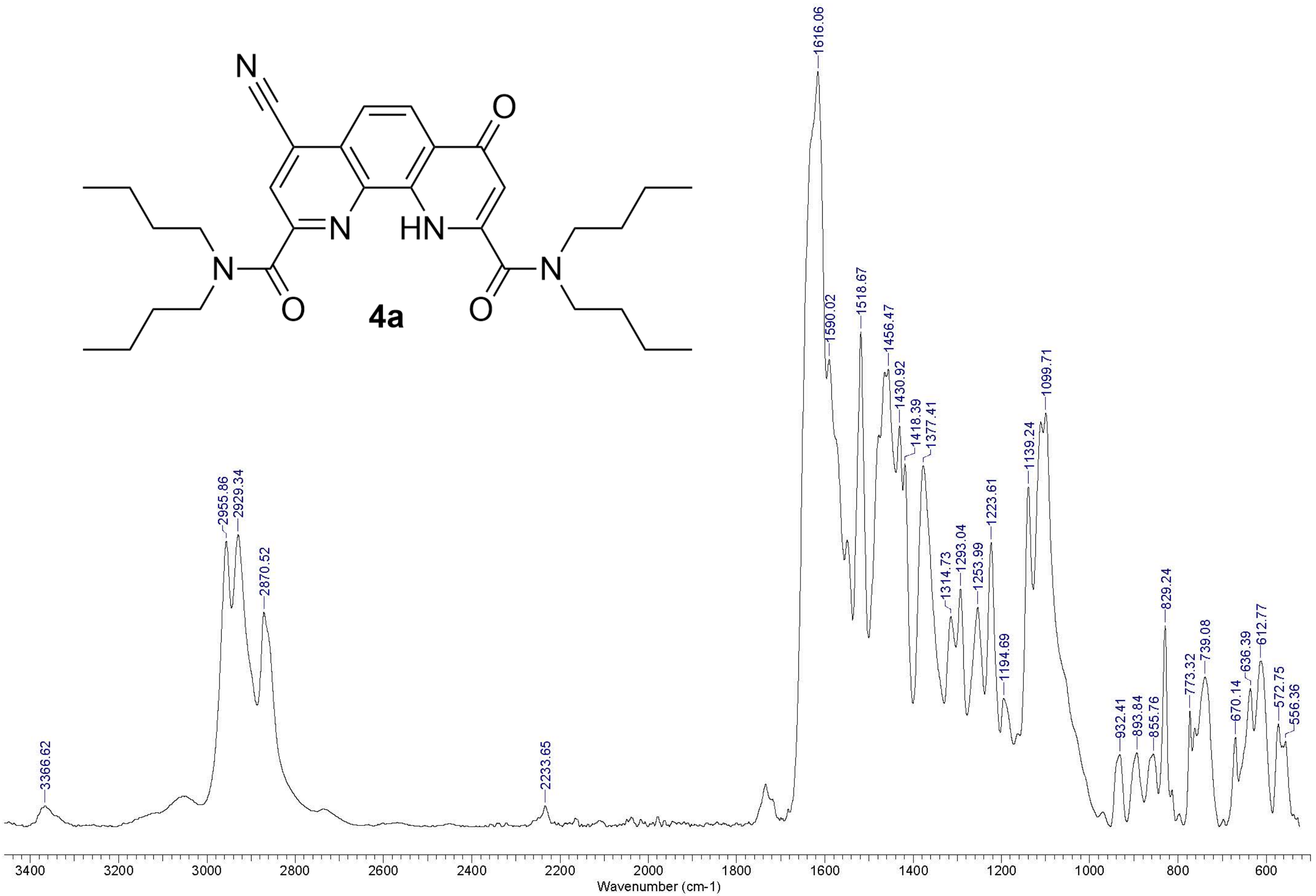

Figure S18. Solid-state IR spectrum of **4a**

7-Cyano-N²,N⁹-diethyl-4-oxo-N²,N⁹-di-p-tolyl-1,4-dihydro-1,10-phenanthroline-2,9-dicarboxamide (4b)

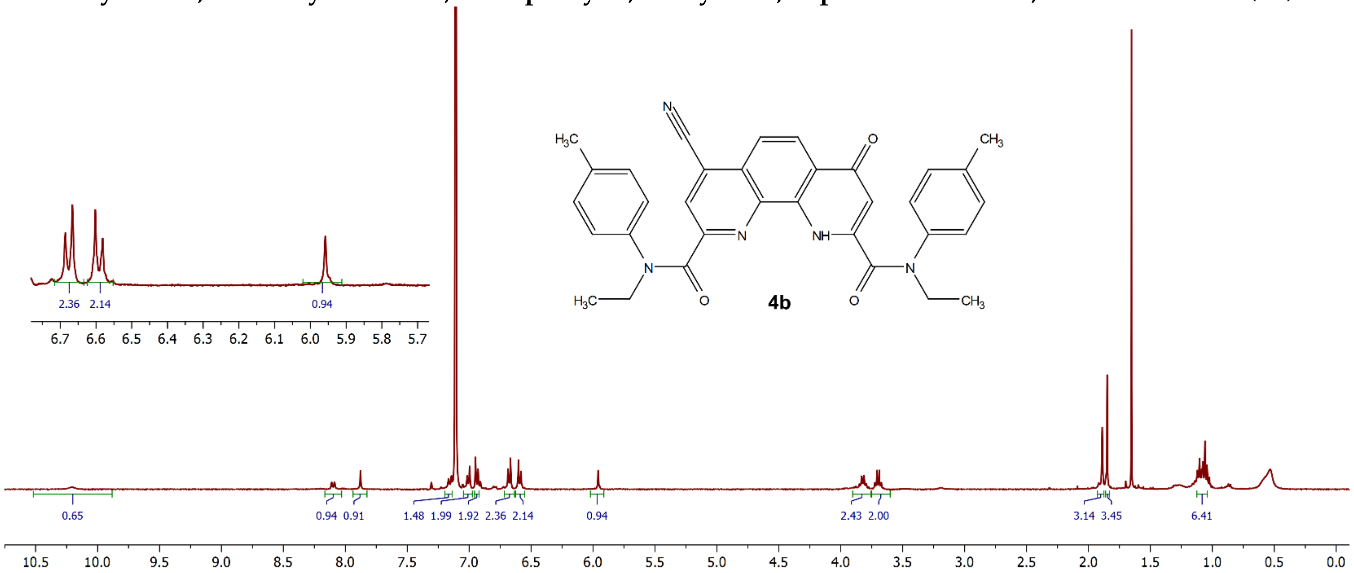

Figure S19. ^{1}H NMR spectrum of **4b** (400 MHz, C_6D_6)

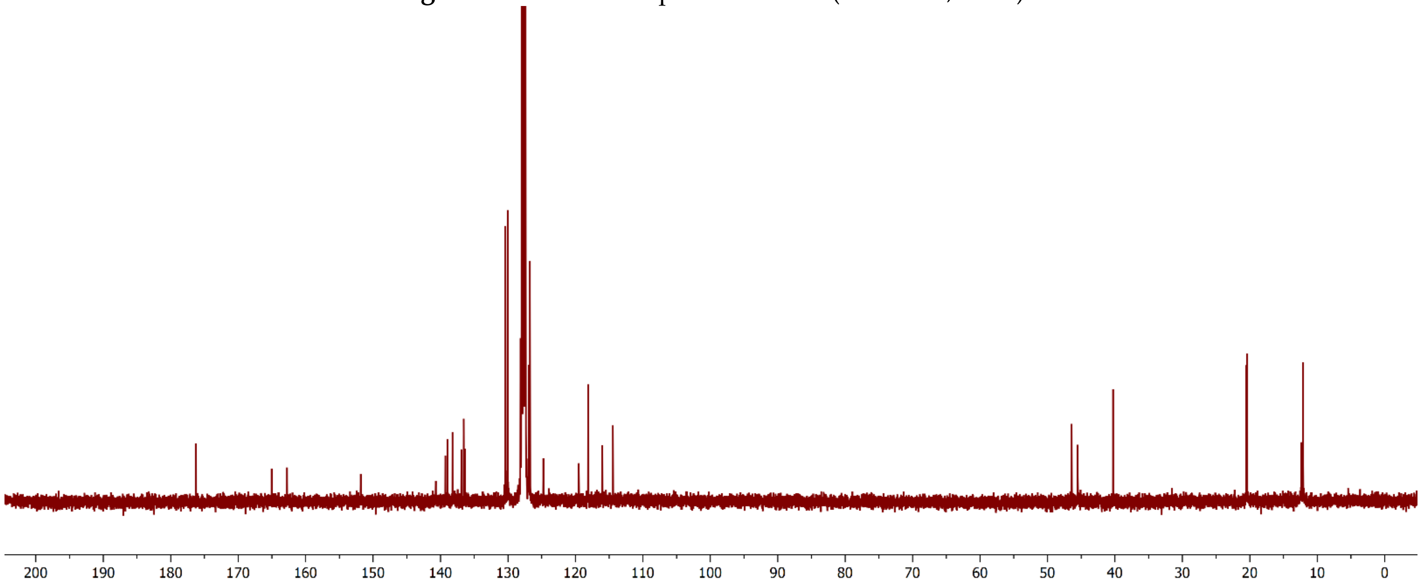

Figure S20. $^{13}C\{^{1}H\}$ NMR spectrum of **4b** (101 MHz, C_6D_6)

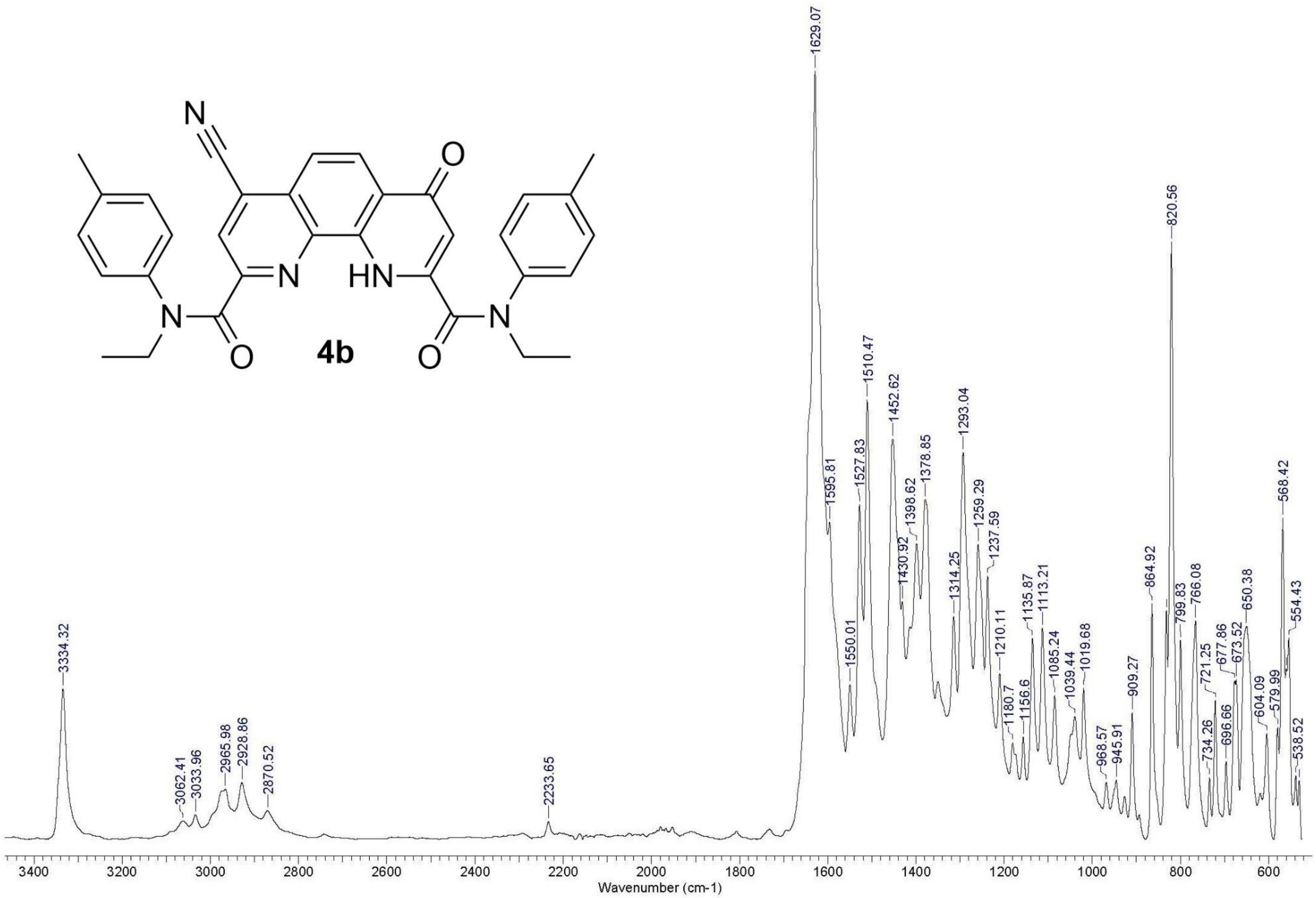

Figure S21. Solid-state IR spectrum of **4b**

N²,N²,N⁹,N⁹-Tetrabutyl-4-oxo-7-(1,4,7,10,13-pentaoxa-16-azacyclooctadecan-16-yl)-1,4-dihydro-1,10-phenanthroline-2,9-dicarboxamide (5a)

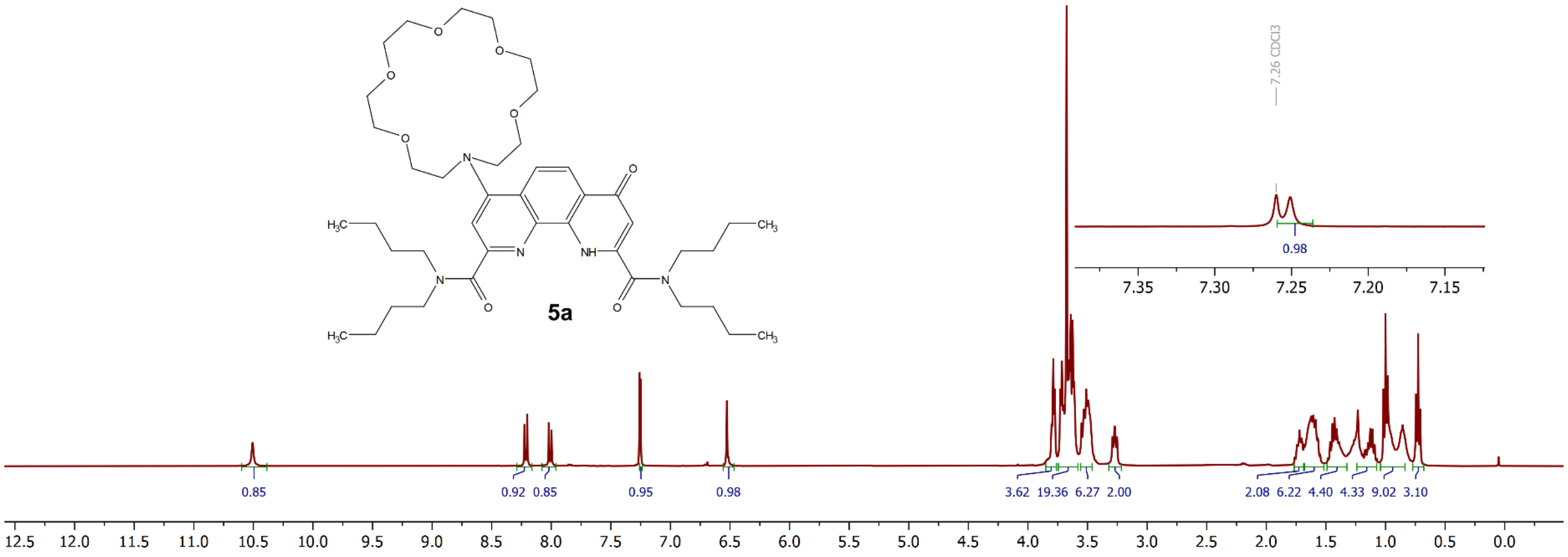

5a

Figure S22. ^{1}H NMR spectrum of **5a** (400 MHz, $CDCl_3$)

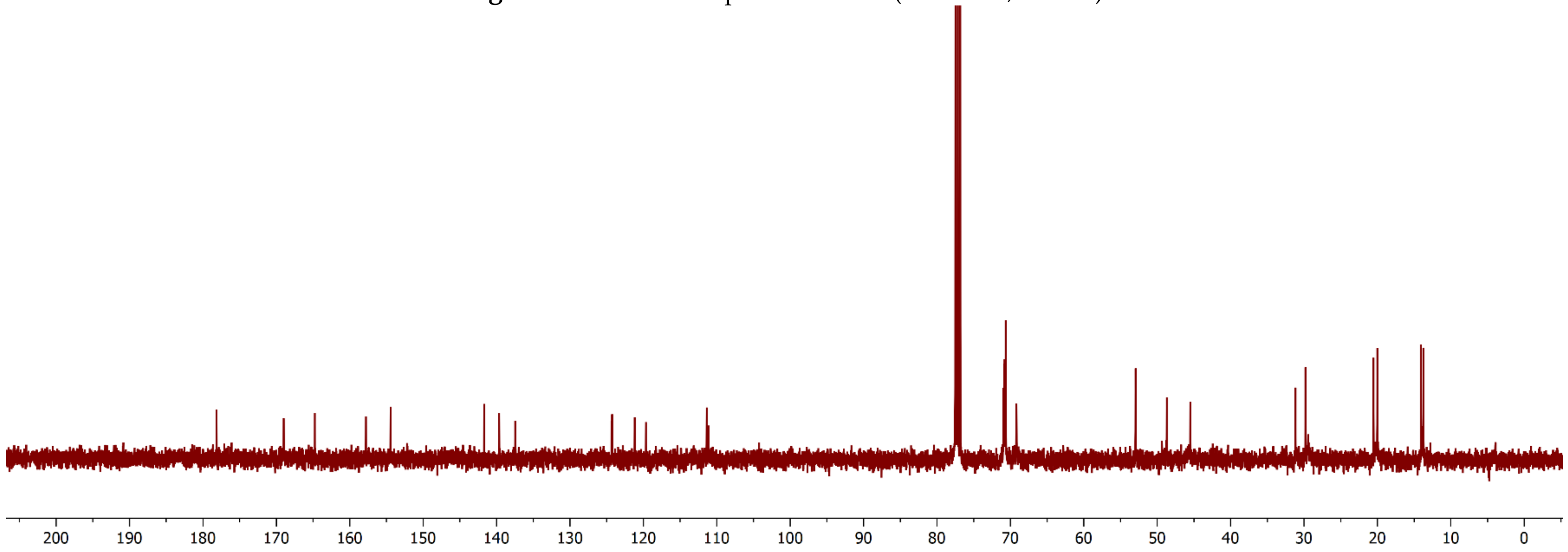

Figure S23. ^{13}C{^{1}H} NMR spectrum of **4b** (101 MHz, $CDCl_3$)

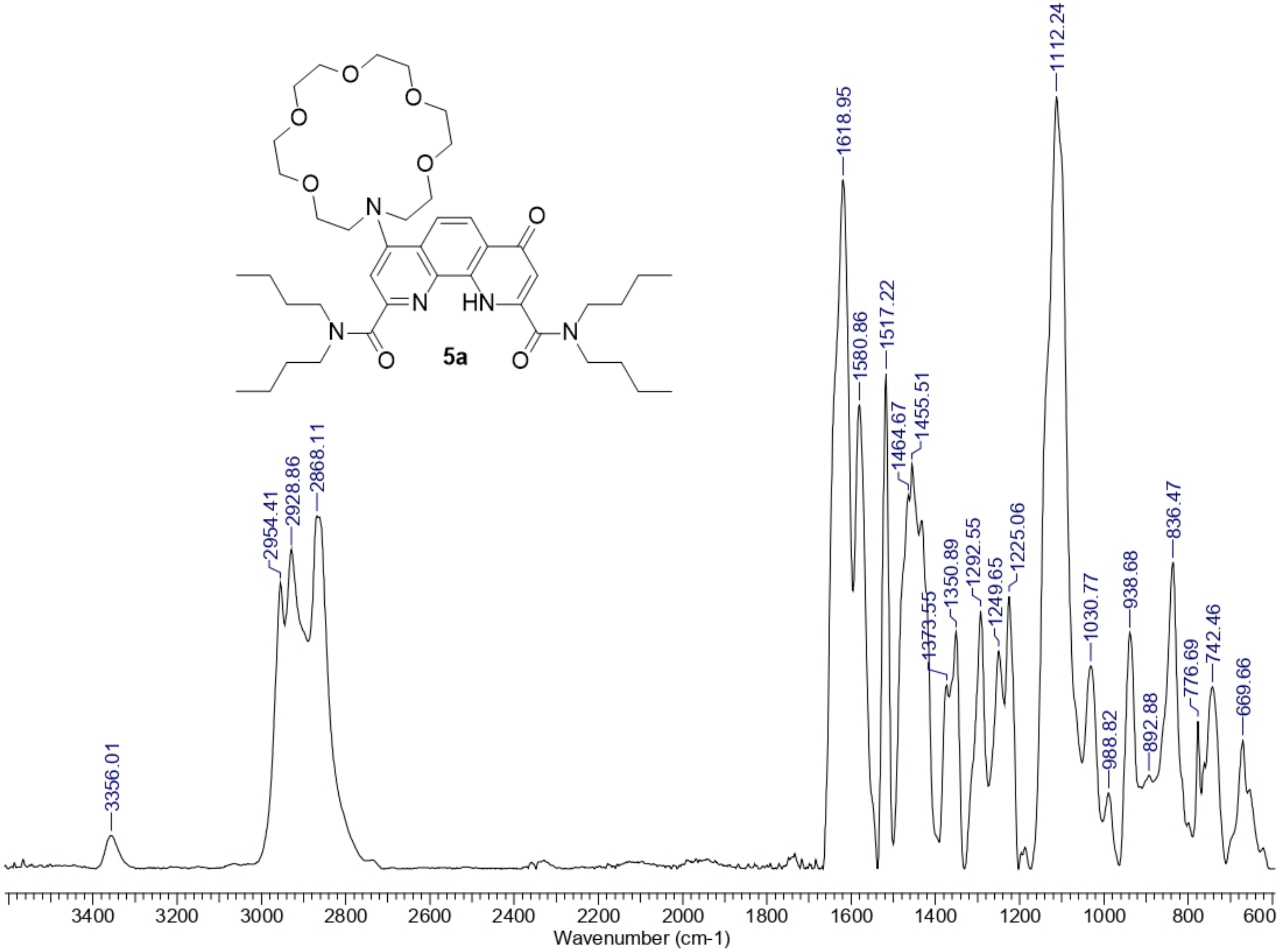

Figure S24. Solid-state IR spectrum of **5a**

7-Azido-N²,N²,N⁹,N⁹-tetrabutyl-4-oxo-1,4-dihydro-1,10-phenanthroline-2,9-dicarboxamide (6a)

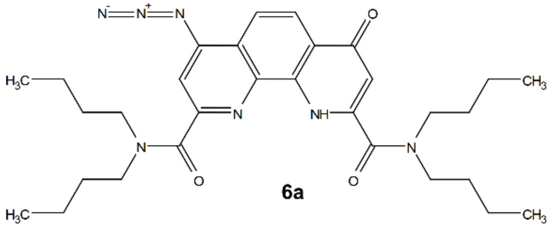

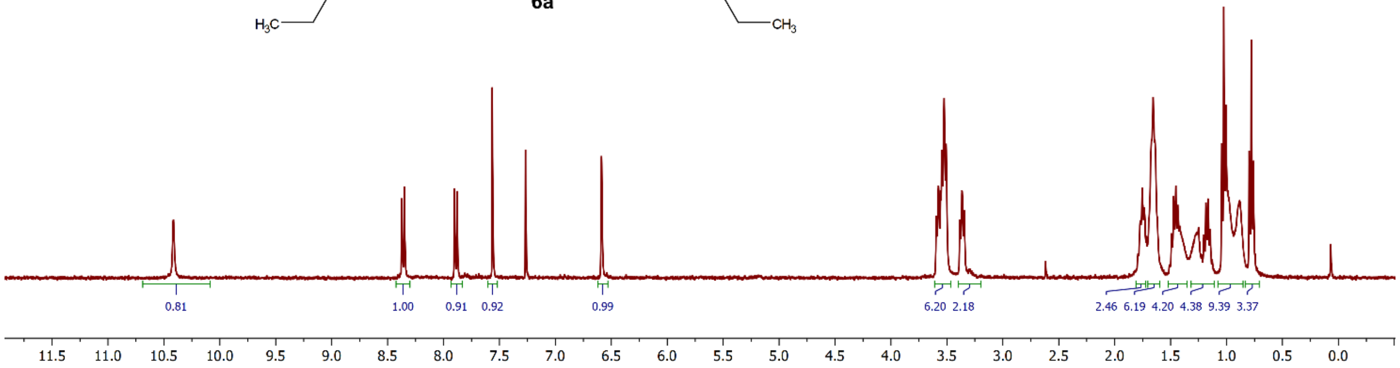

Figure S25. ^{1}H NMR spectrum of **6a** (400 MHz, $CDCl_3$)

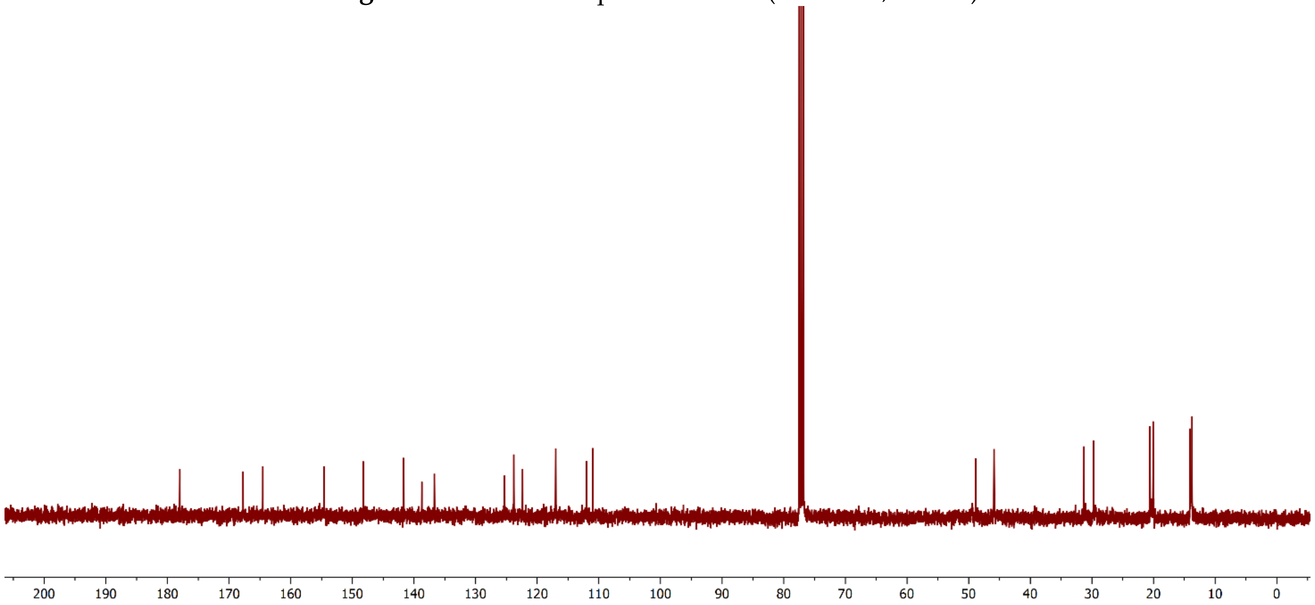

Figure S26. $^{13}C\{^{1}H\}$ NMR spectrum of **6a** (101 MHz, $CDCl_3$)

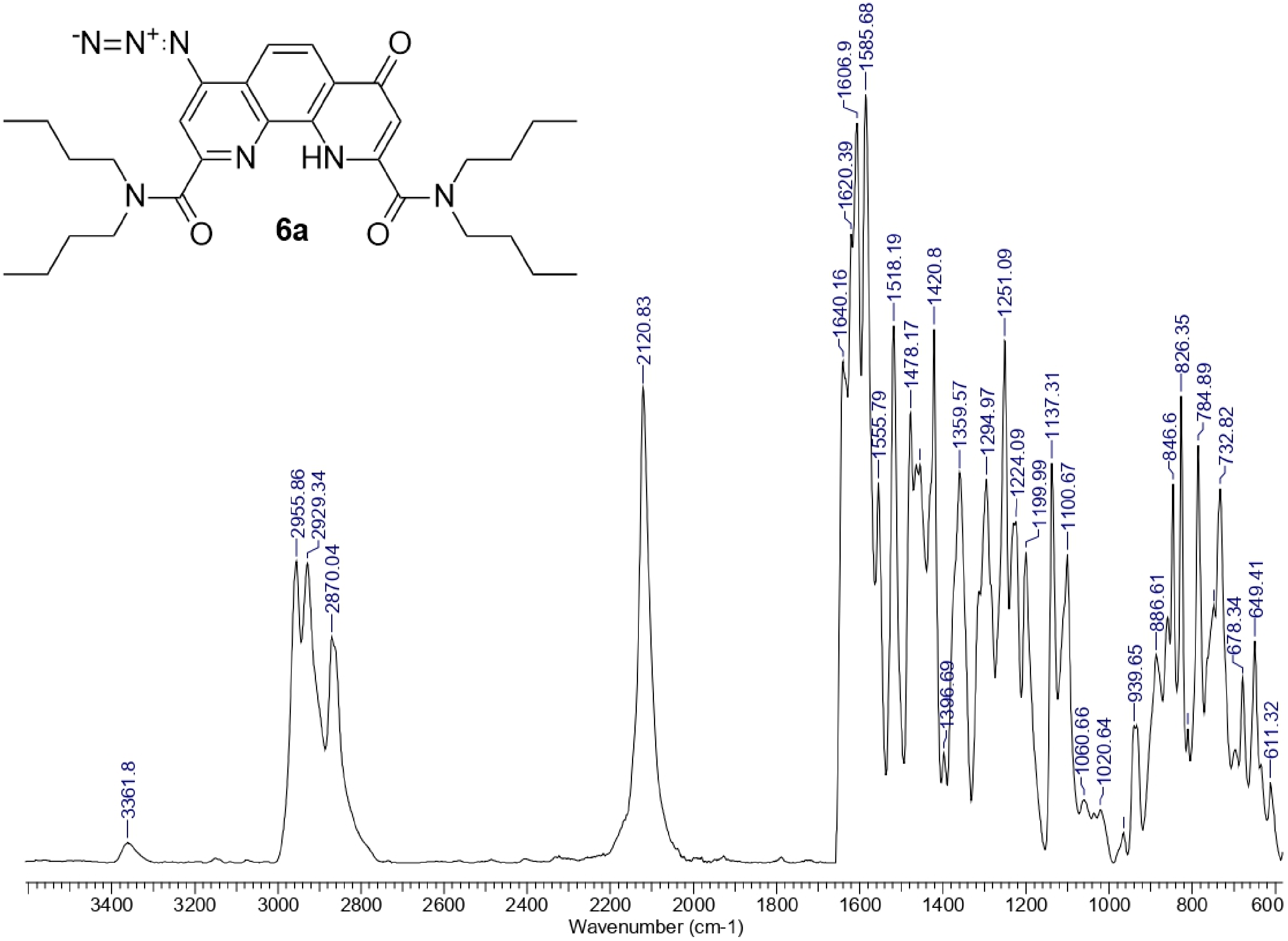

Figure S27. Solid-state IR spectrum of **6a**

7-Azido-N[2],N[9]-diethyl-4-oxo-N[2],N[9]-di-p-tolyl-1,4-dihydro-1,10-phenanthroline-2,9-dicarboxamide (6b)

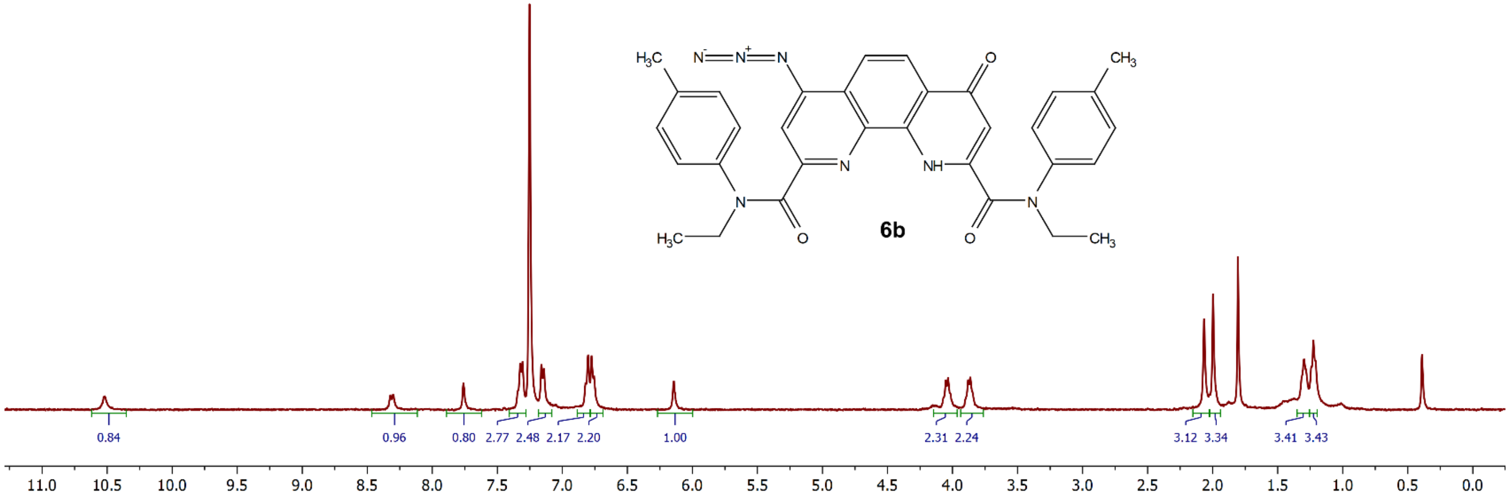

Figure S28. ^{1}H NMR spectrum of **6b** (400 MHz, C_6D_6)

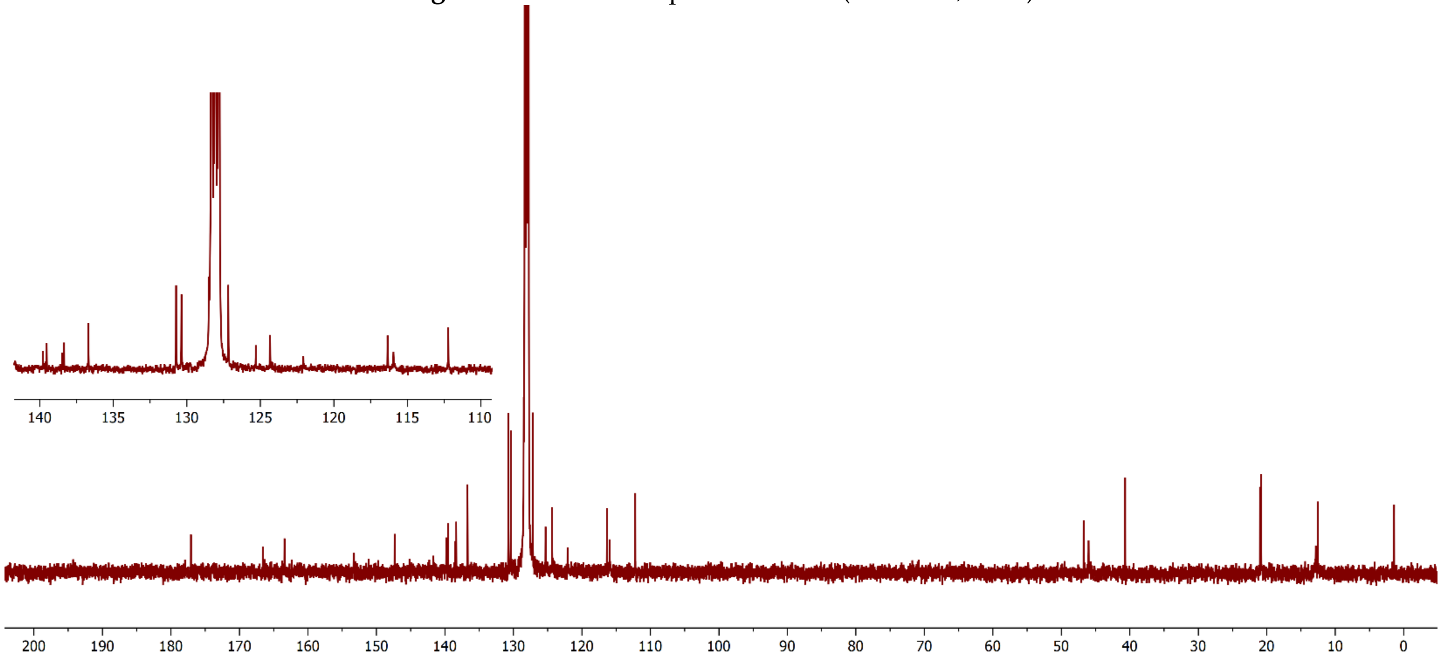

Figure S29. $^{13}C\{^{1}H\}$ NMR spectrum of **6b** (101 MHz, C_6D_6)

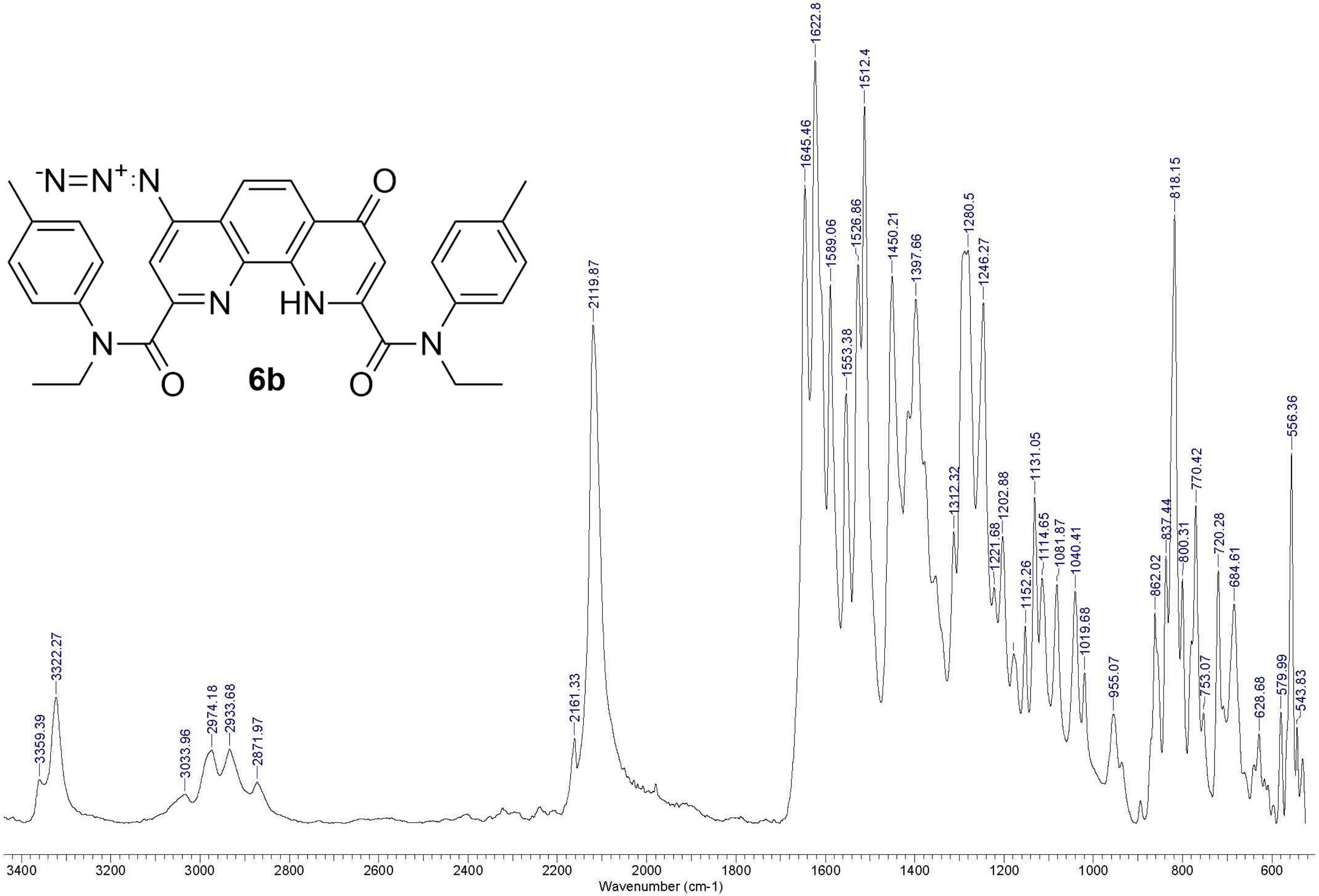

Figure S30. Solid-state IR spectrum of **6b**

N²,N⁹-Diethyl-7-methoxy-4-oxo-N²,N⁹-di-p-tolyl-1,4-dihydro-1,10-phenanthroline-2,9-dicarboxamide (7b)

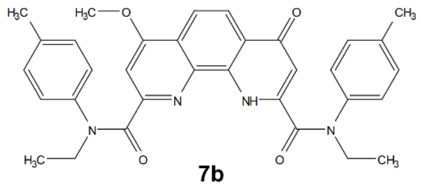

7b

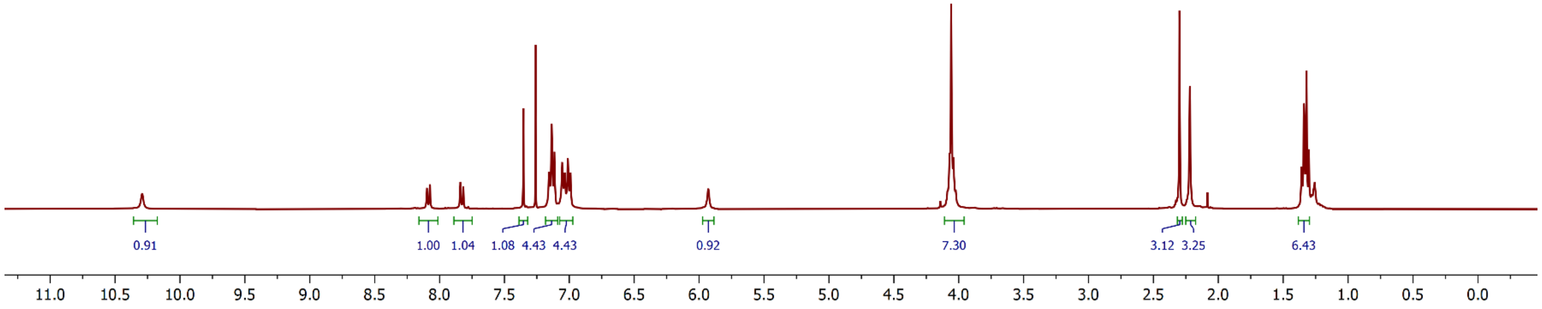

Figure S31. ^{1}H NMR spectrum of **7b** (400 MHz, $CDCl_3$)

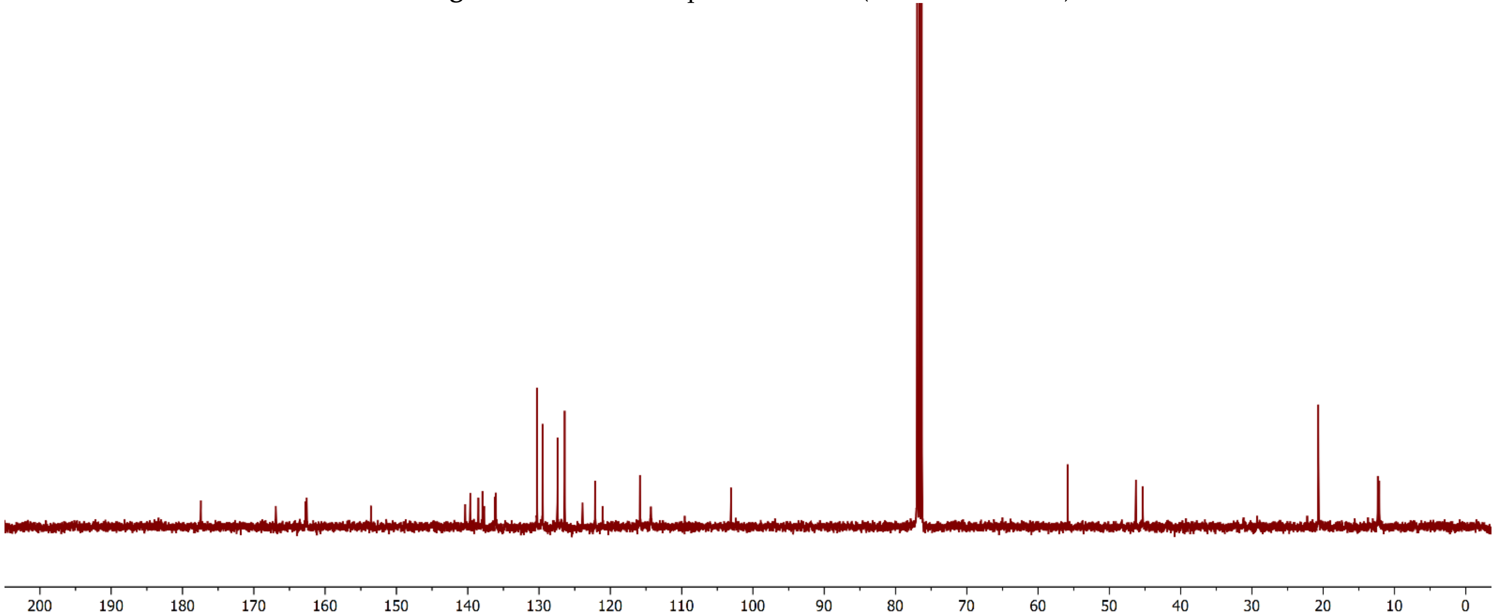

Figure S32. $^{13}C\{^{1}H\}$ NMR spectrum of **7b** (101 MHz, $CDCl_3$)

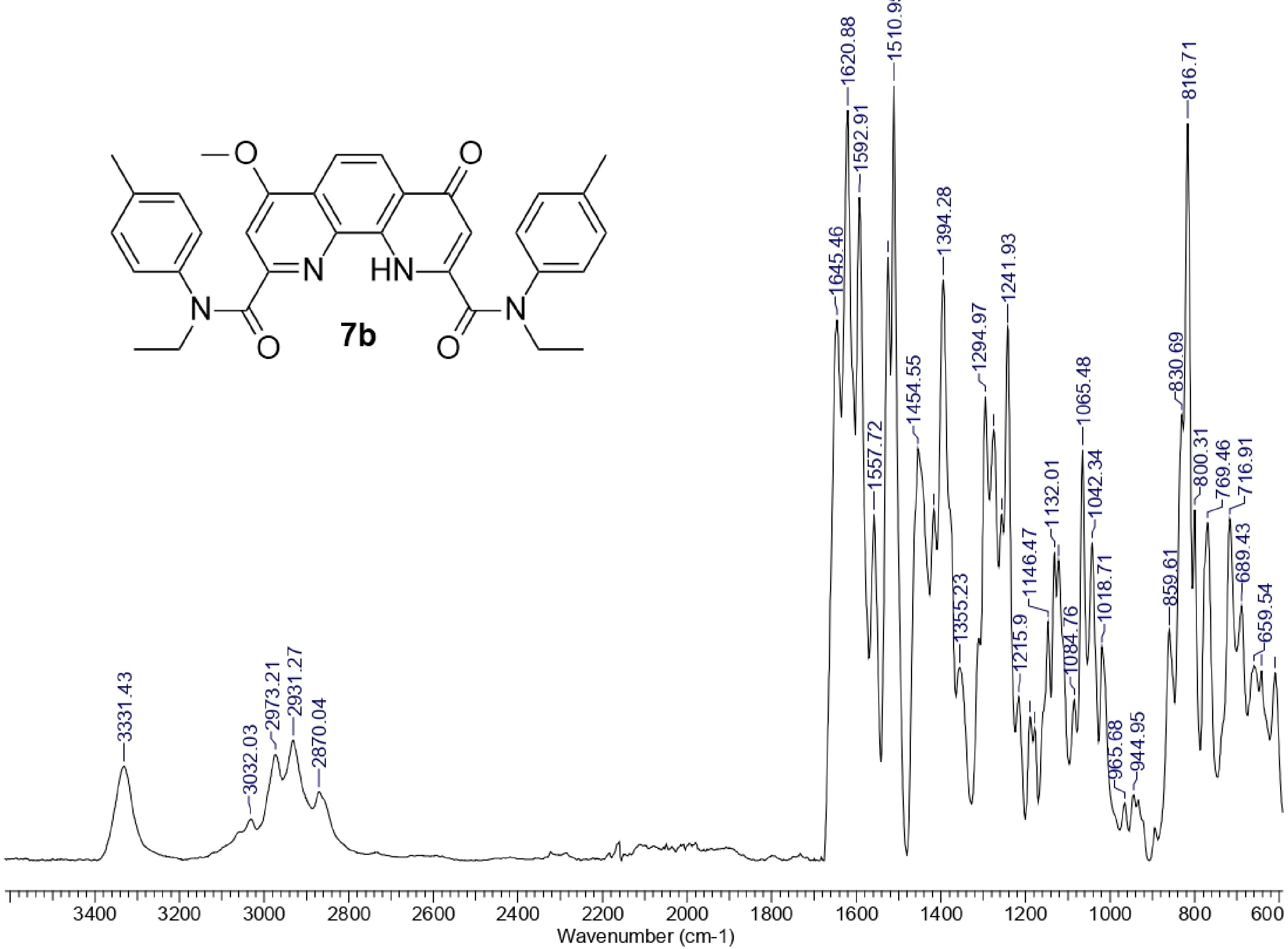

Figure S33. Solid-state IR spectrum of **7b**

N²,N²,N⁹,N⁹-Tetrabutyl-4-oxo-7-((triphenylphosphoranylidene)amino)-1,4-dihydro-1,10-phenanthroline-2,9-dicarboxamide (8a)

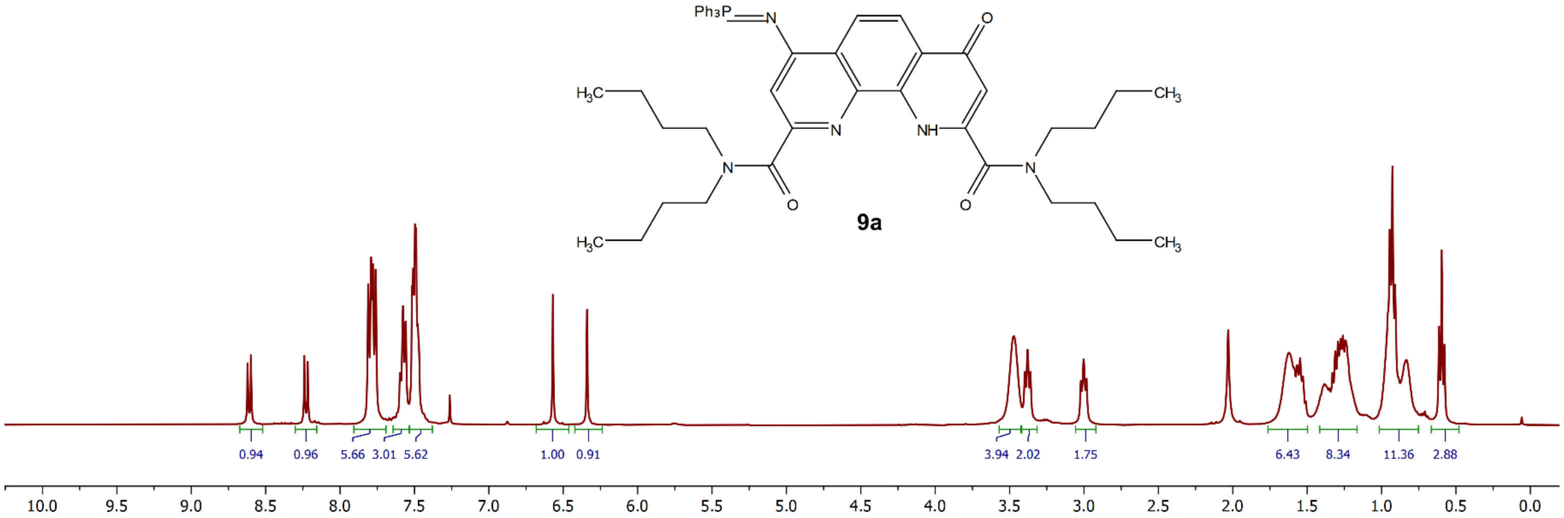

Figure S34. ^{1}H NMR spectrum of **9a** (400 MHz, $CDCl_3$)

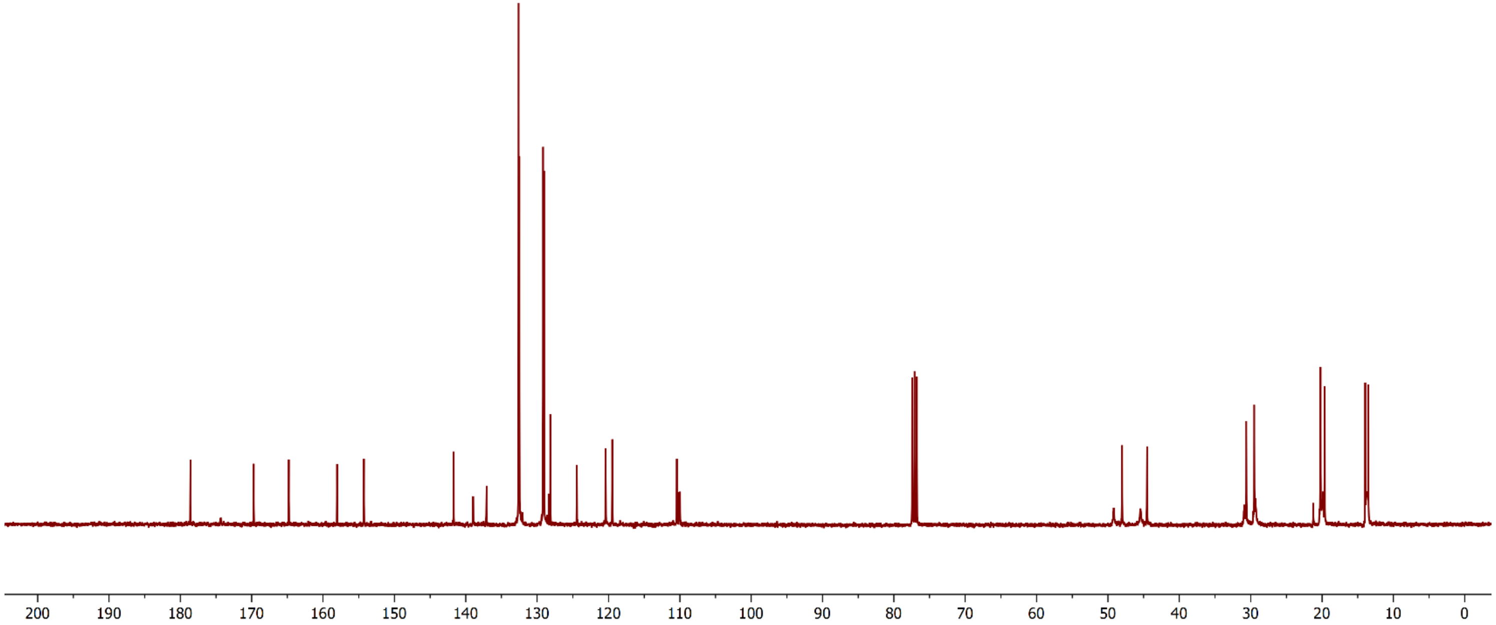

Figure S35. $^{13}C\{^{1}H\}$ NMR spectrum of **9a** (101 MHz, $CDCl_3$)

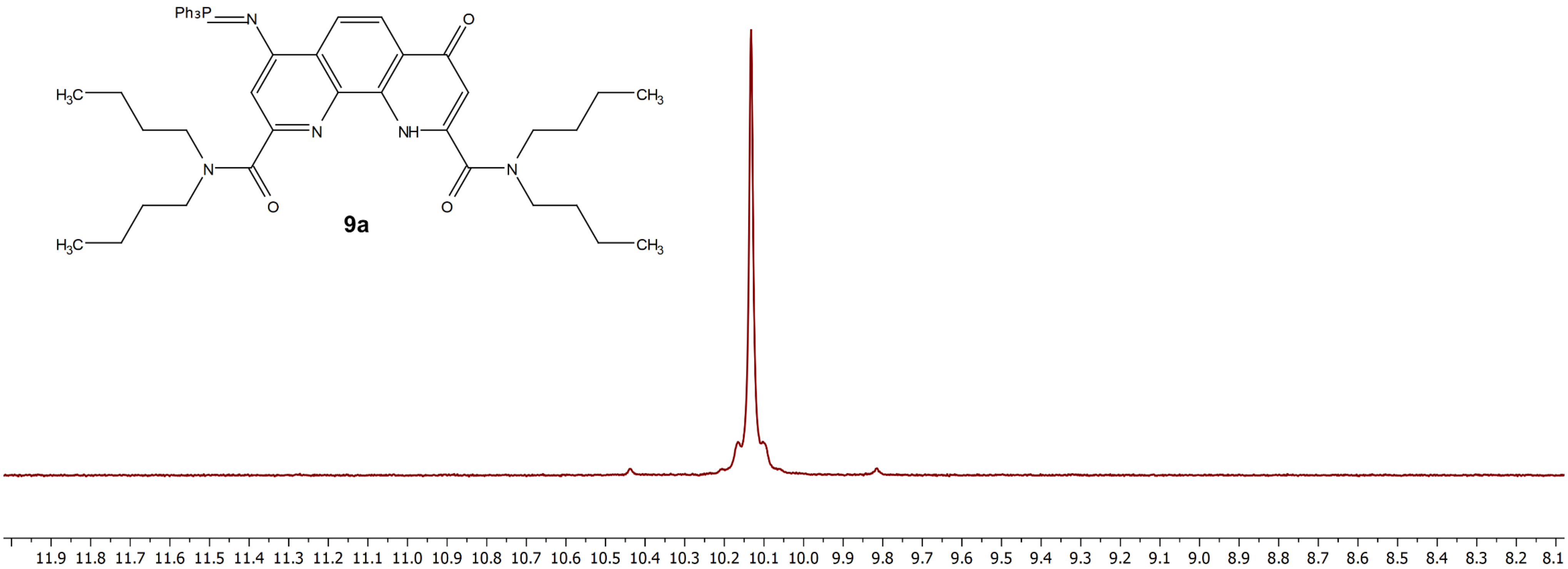

Figure S36. ^{31}P NMR spectrum of **9a** (101 MHz, $CDCl_3$)

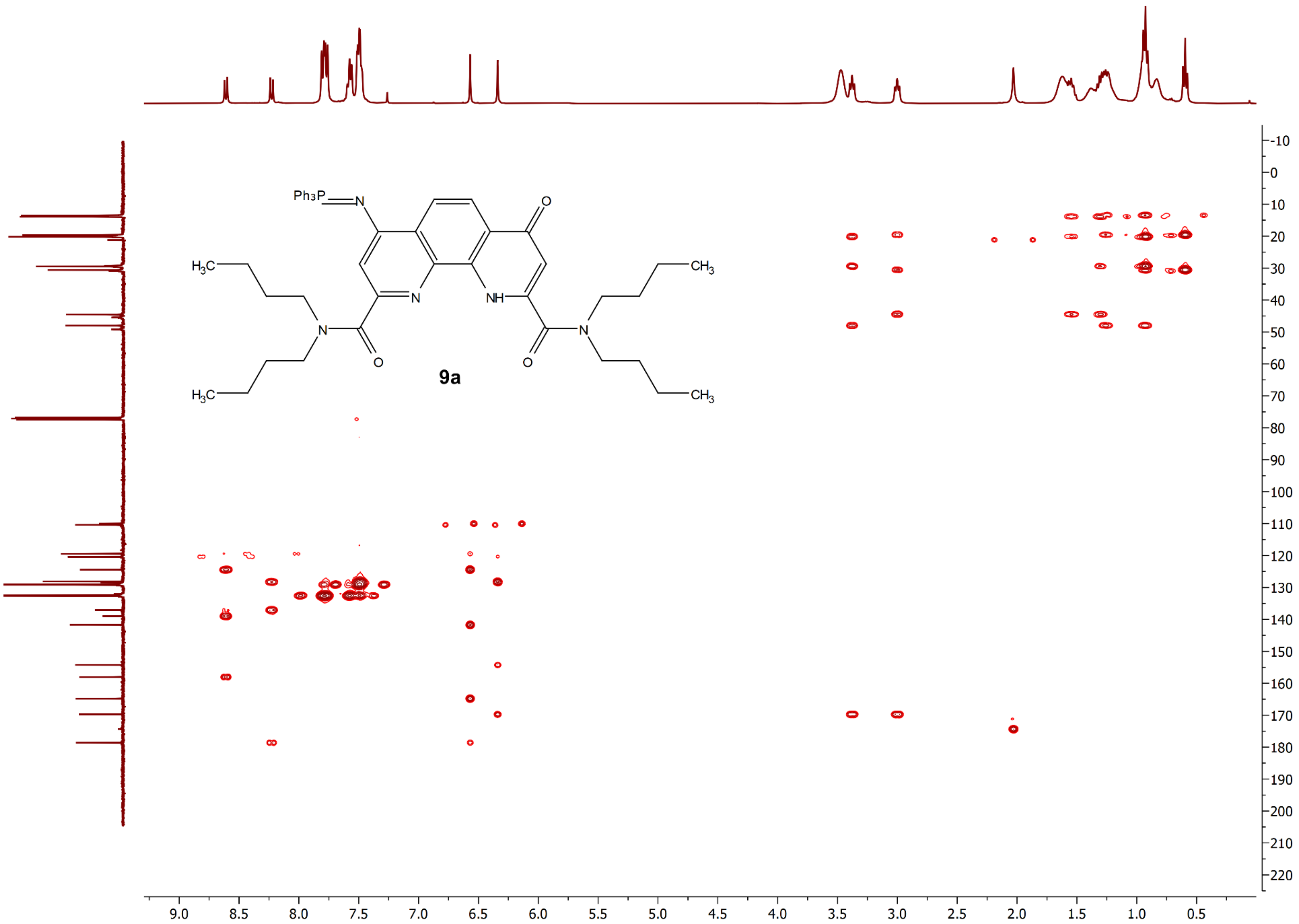

Figure S37. $^{1}H/^{13}C$ HMBC NMR spectrum of **9a** ($CDCl_3$)

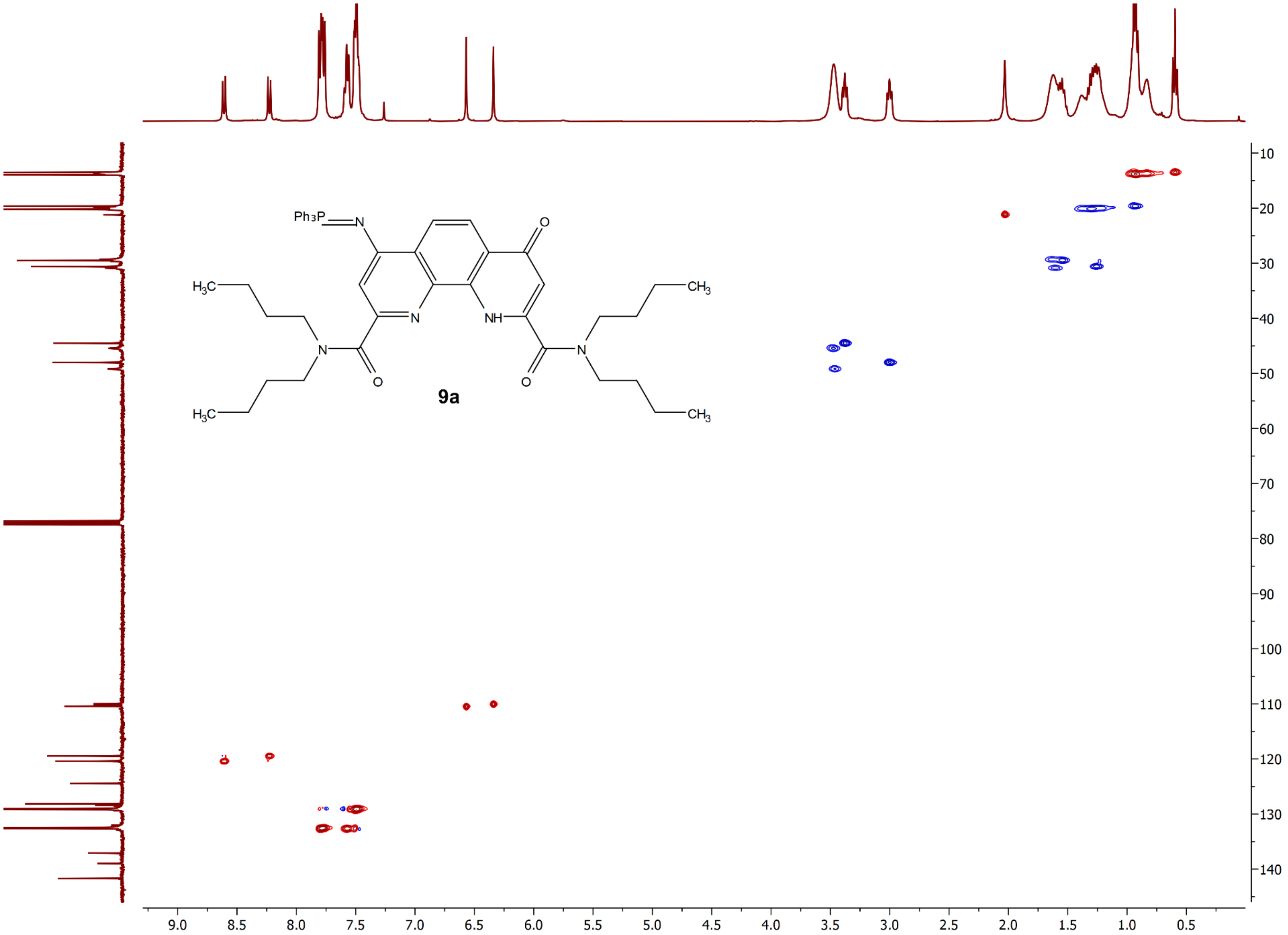

Figure S38. $^{1}H/^{13}C$ HSQC NMR spectrum of **9a** ($CDCl_3$)

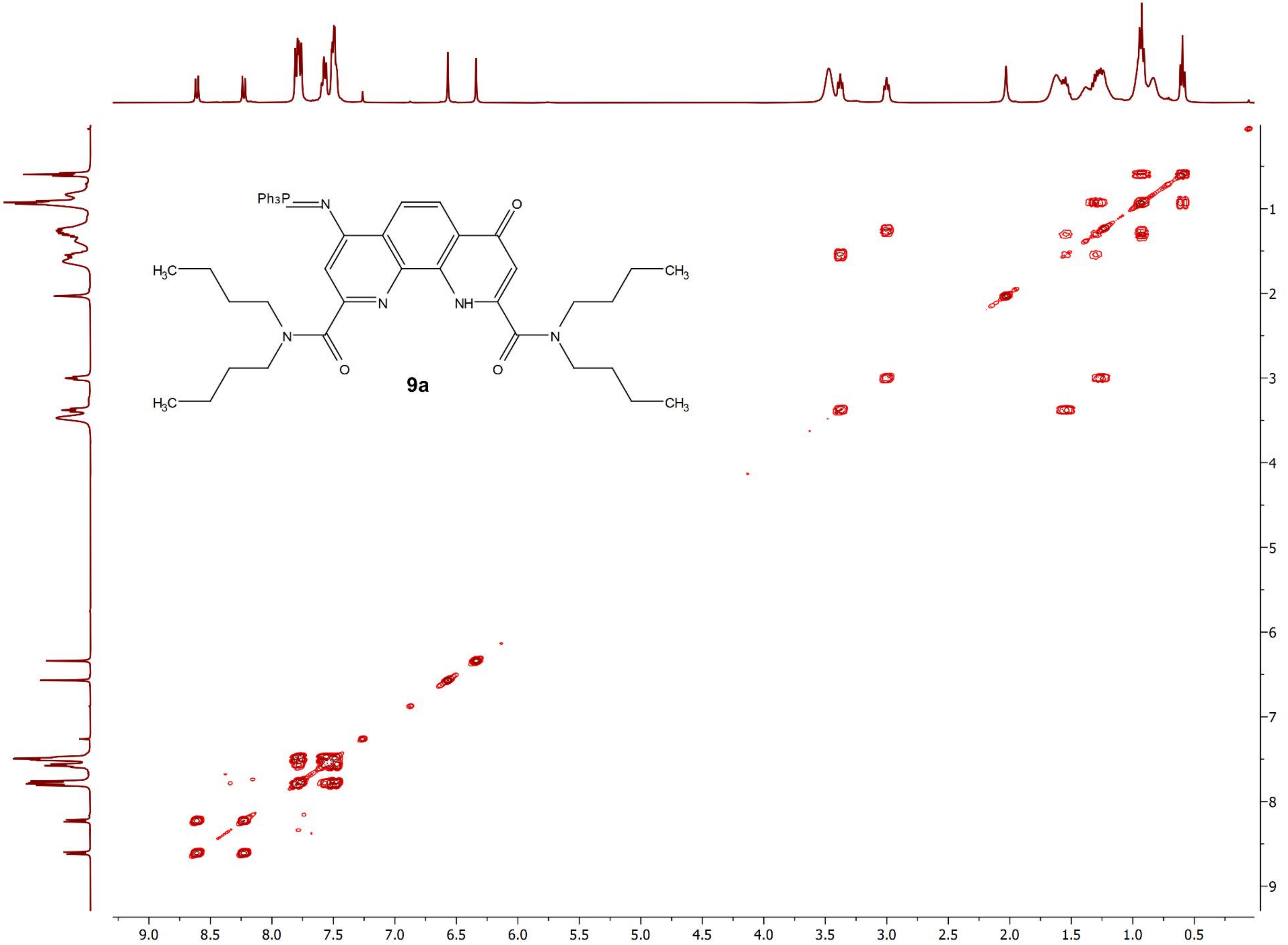

Figure S39. $^1H/^1H$ COSY NMR spectrum of **9a** ($CDCl_3$)

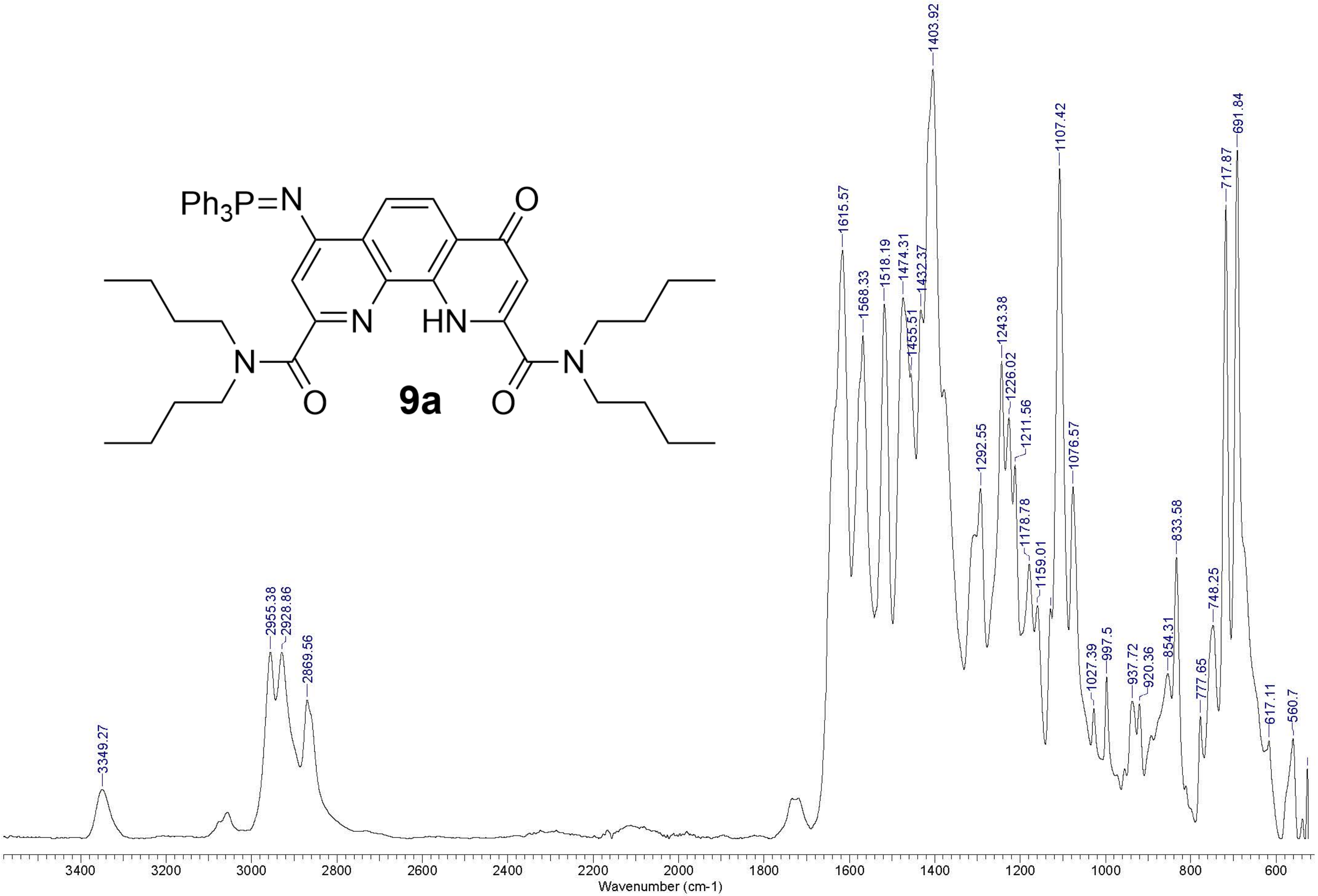

Figure S40. Solid-state IR spectrum of **9a**

N²,N⁹-Diethyl-4-oxo-N²,N⁹-di-p-tolyl-7-((triphenylphosphoranylidene)amino)-1,4-dihydro-1,10-phenanthroline-2,9-dicarboxamide (9b)

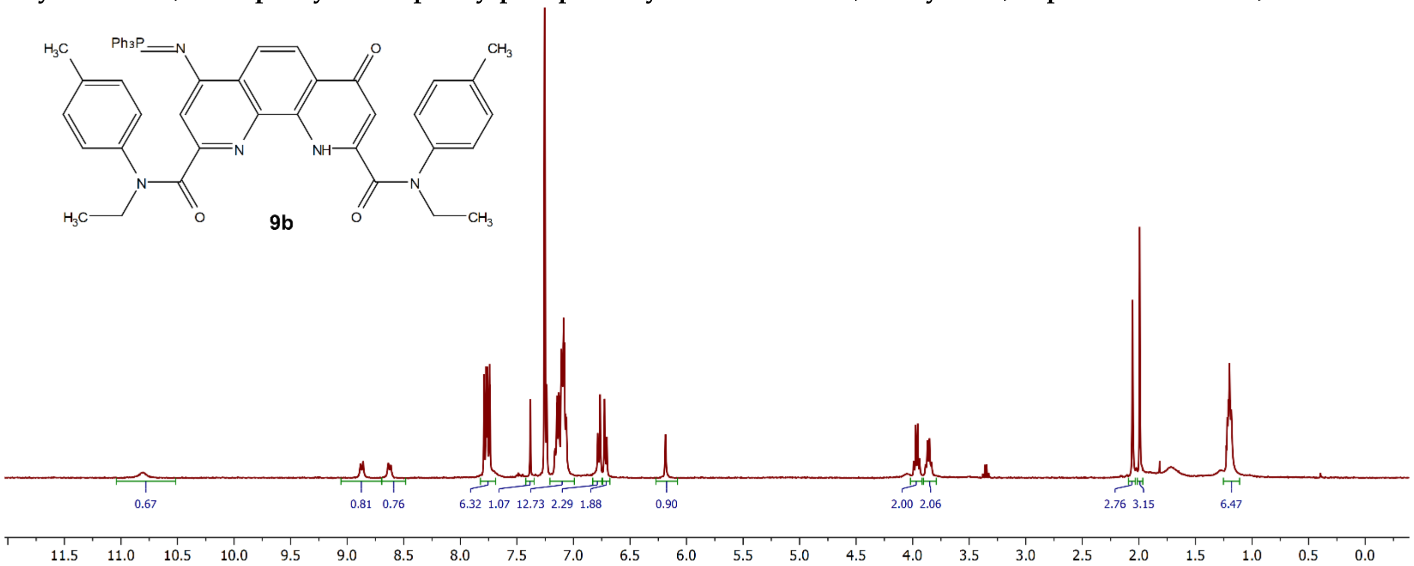

Figure S41. ^{1}H NMR spectrum of **9b** (400 MHz, C_6D_6)

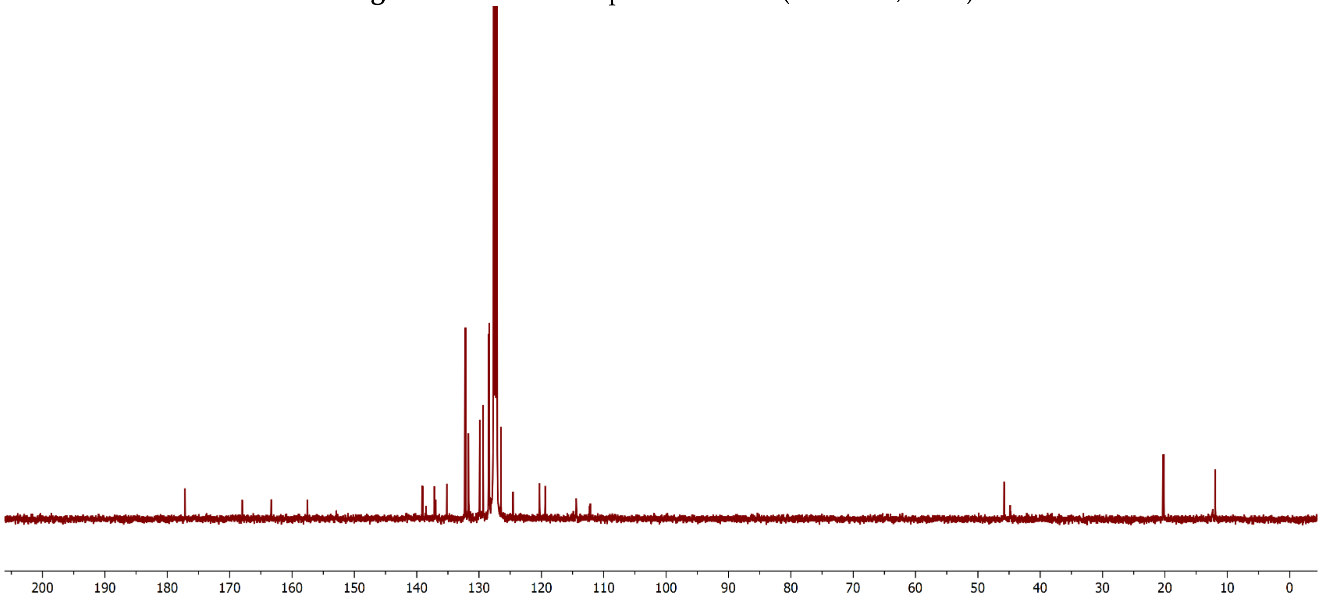

Figure S42. $^{13}C\{^{1}H\}$ NMR spectrum of **9b** (101 MHz, C_6D_6)

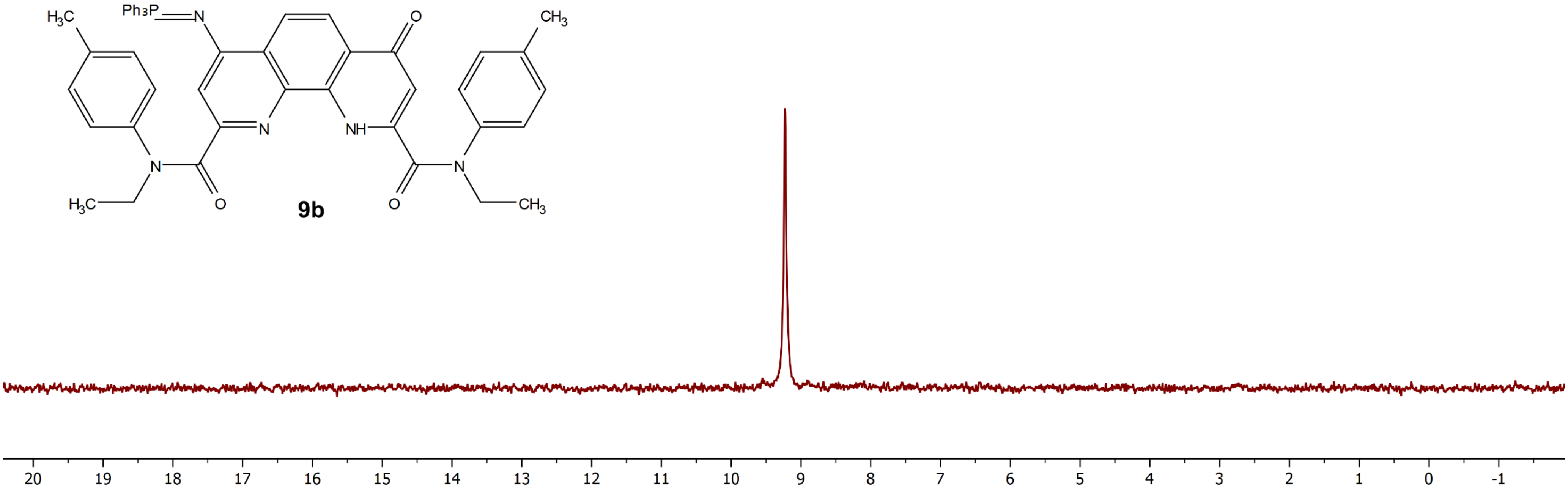

Figure S43. ^{31}P NMR spectrum of **9b** (101 MHz, C_6D_6)

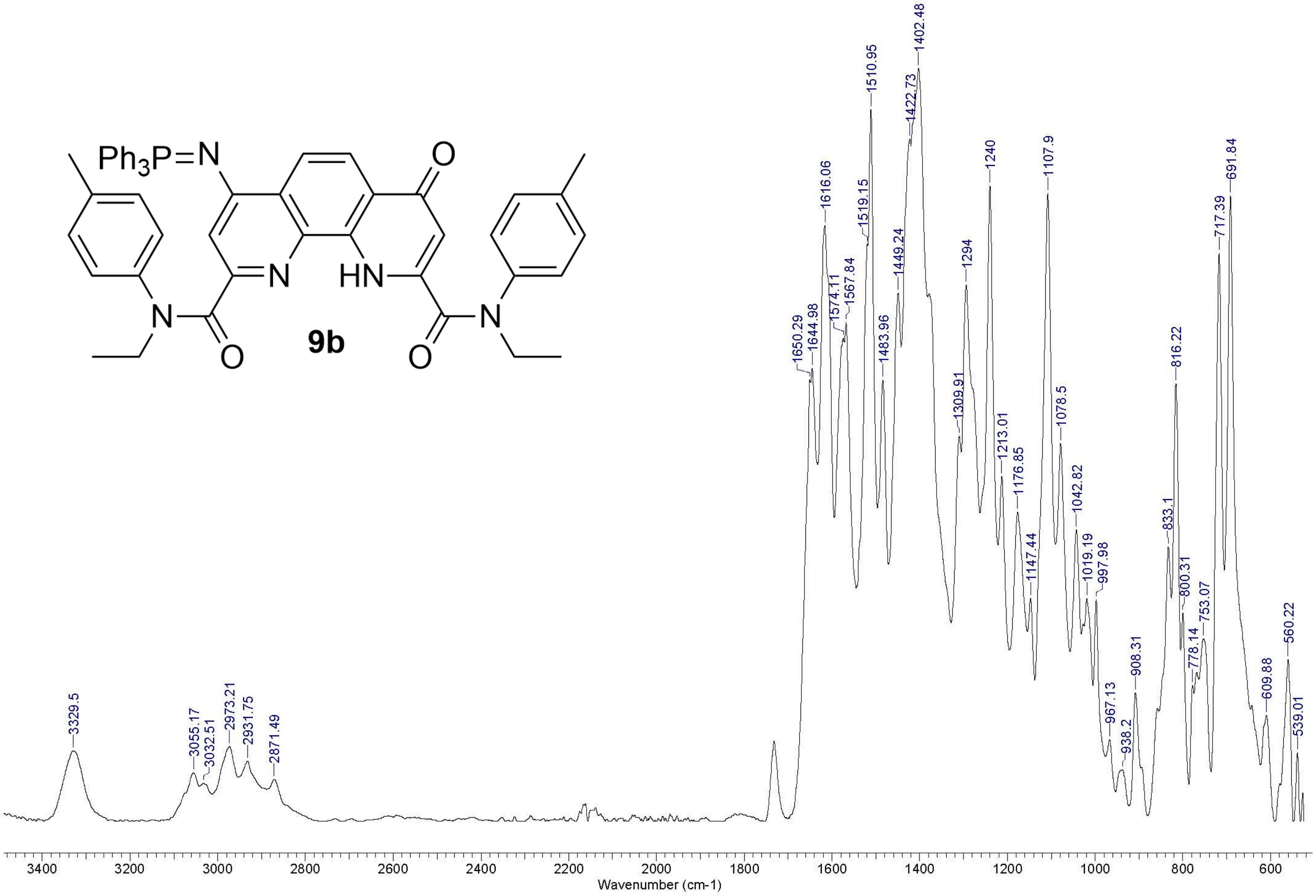

Figure S44. Solid-state IR spectrum of **9b**

Triethyl (2,9-bis(ethyl(p-tolyl)carbamoyl)-7-oxo-7,10-dihydro-1,10-phenanthrolin-4-yl)phosphorimidate (10b)

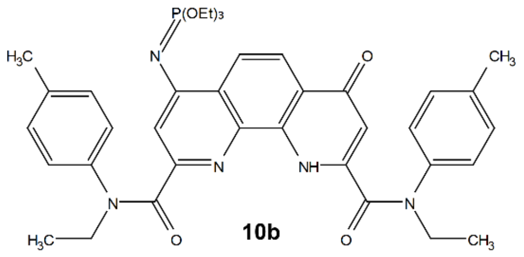

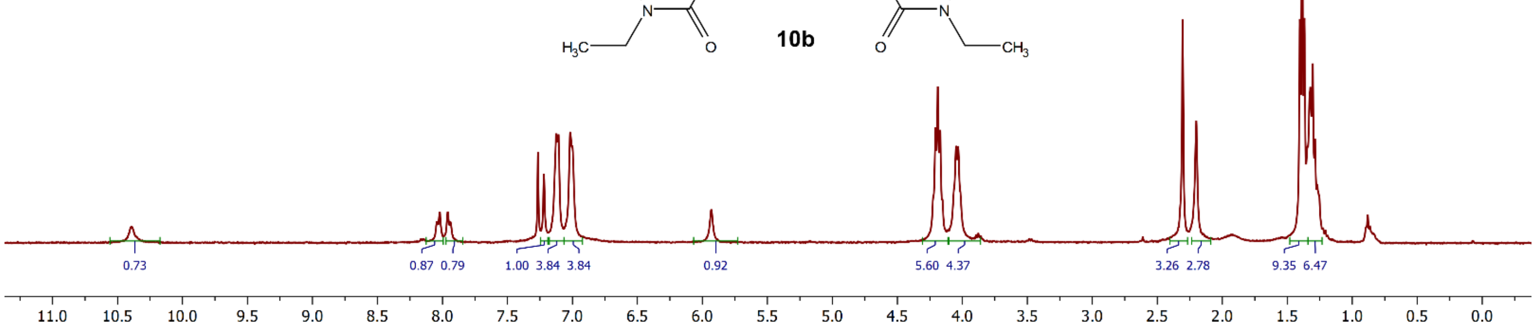

Figure S45. ^{1}H NMR spectrum of **10b** (400 MHz, $CDCl_3$)

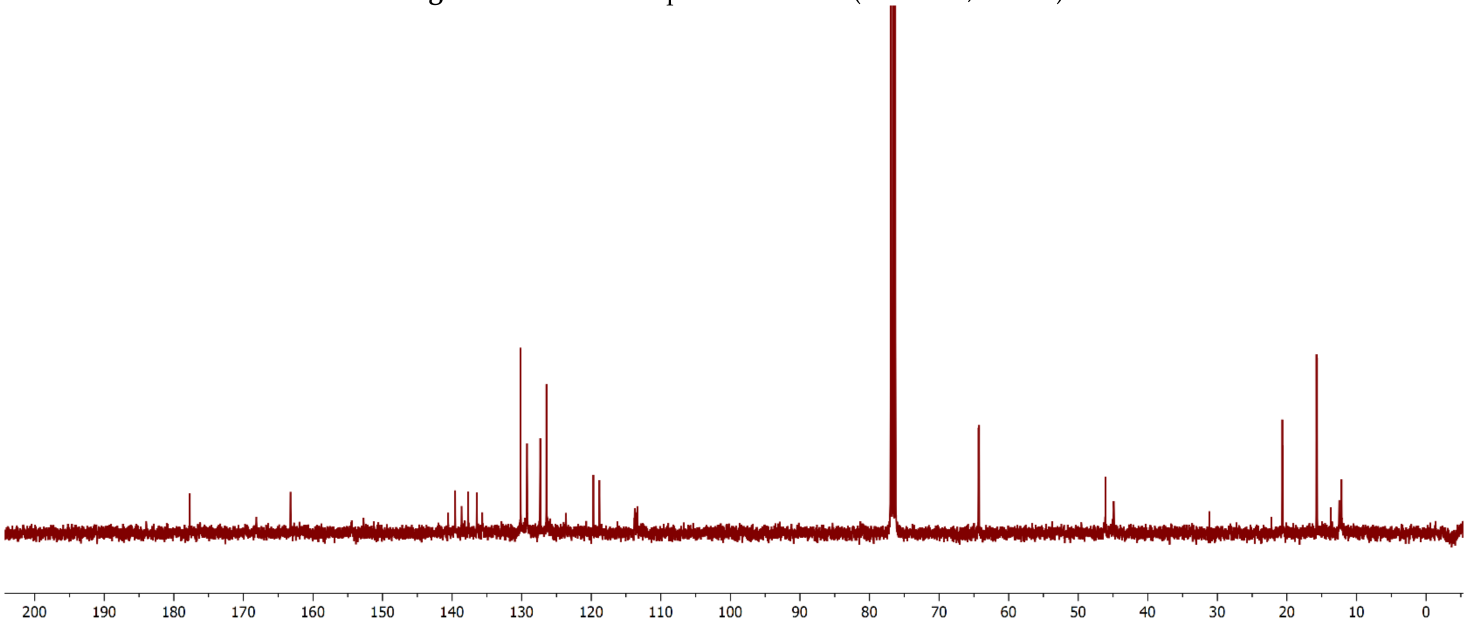

Figure S46. $^{13}C\{^{1}H\}$ NMR spectrum of **10b** (101 MHz, $CDCl_3$)

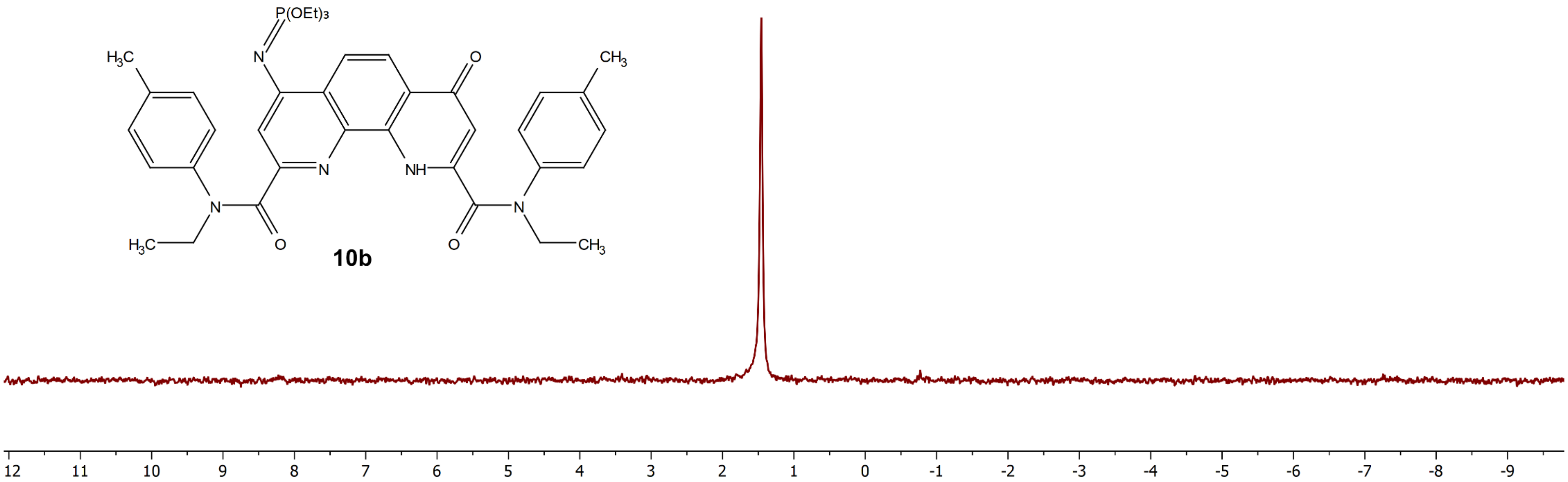

Figure S47. ^{31}P NMR spectrum of **10b** (101 MHz, $CDCl_3$)

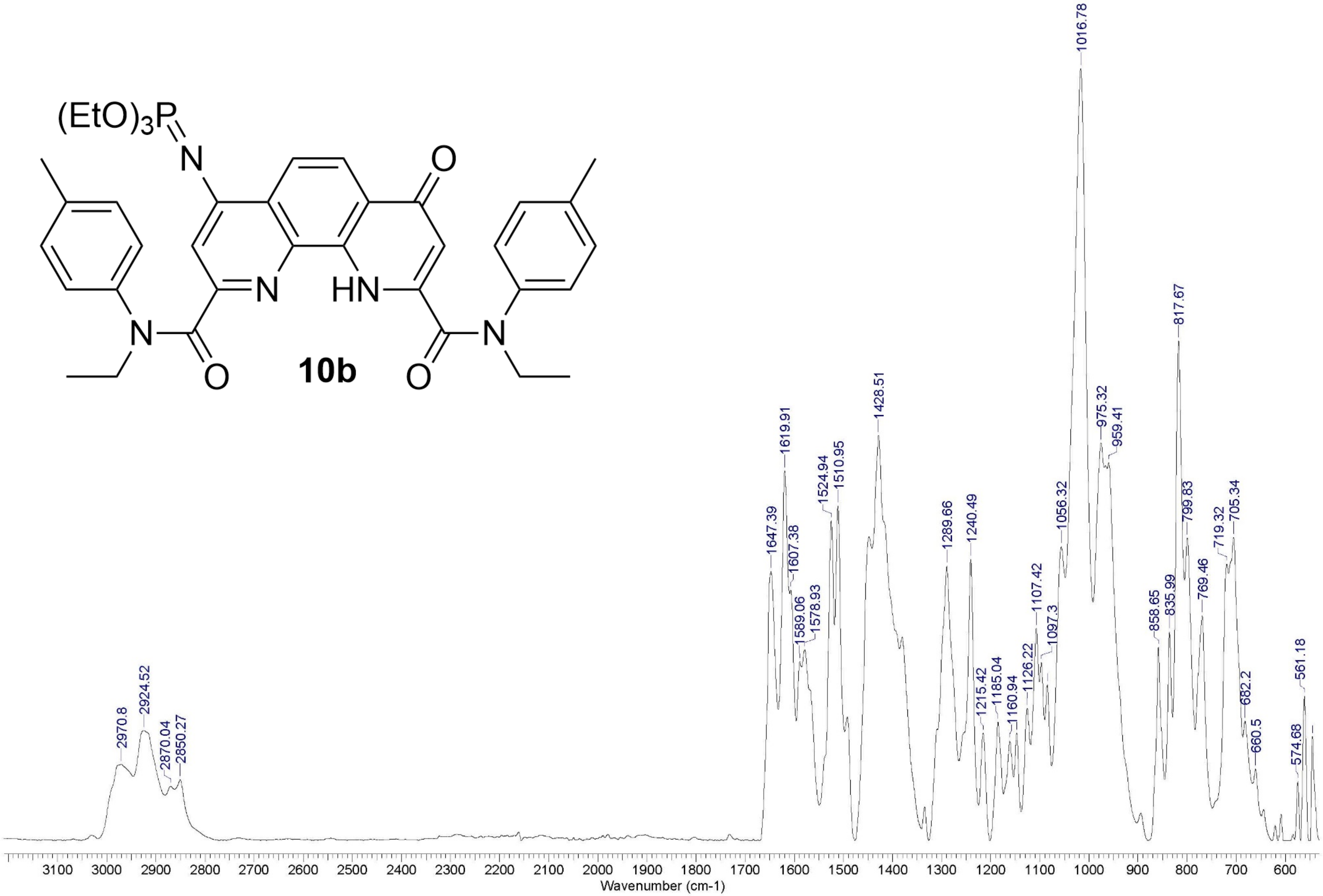

Figure S48. Solid-state IR spectrum of **10b**

Diethyl (2,9-bis(ethyl(p-tolyl)carbamoyl)-7-oxo-7,10-dihydro-1,10-phenanthrolin-4-yl)phosphoramidate (11b)

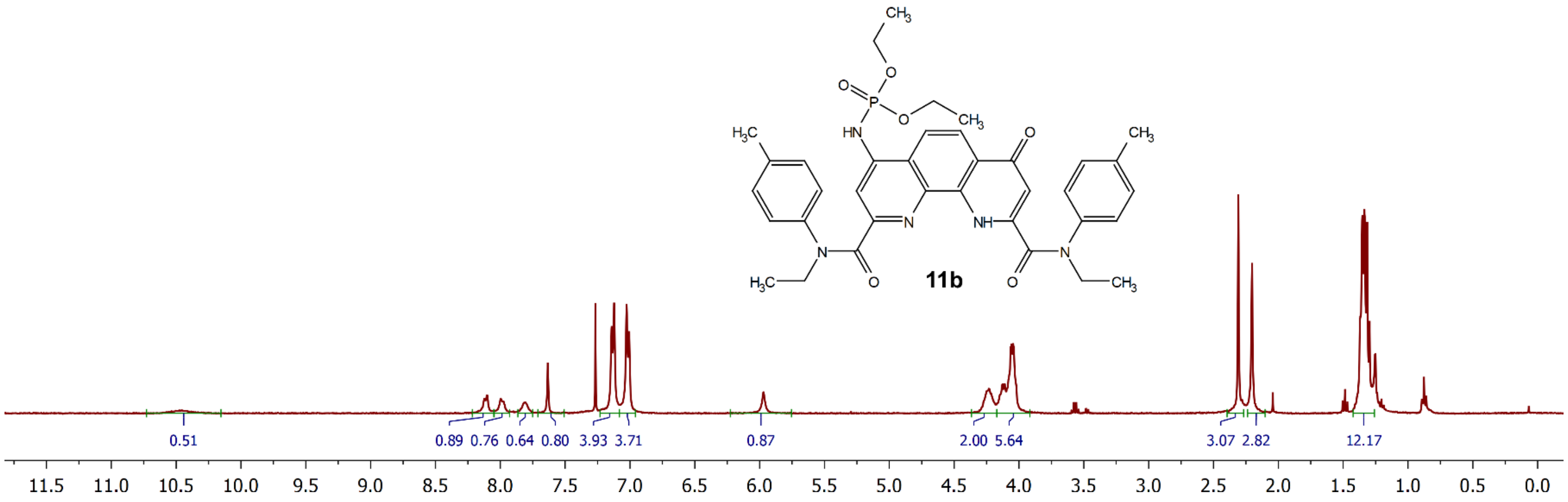

Figure S49. ^{1}H NMR spectrum of **11b** (400 MHz, $CDCl_3$)

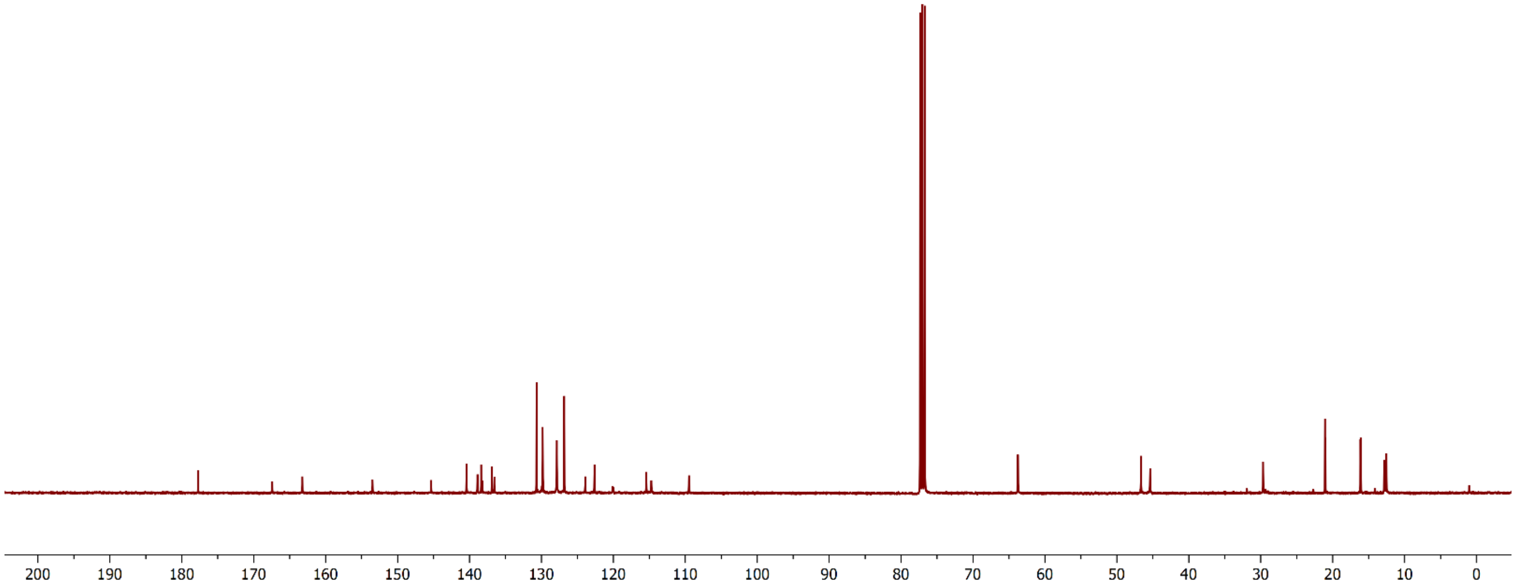

Figure S50. $^{13}C\{^{1}H\}$ NMR spectrum of **11b** (101 MHz, $CDCl_3$)

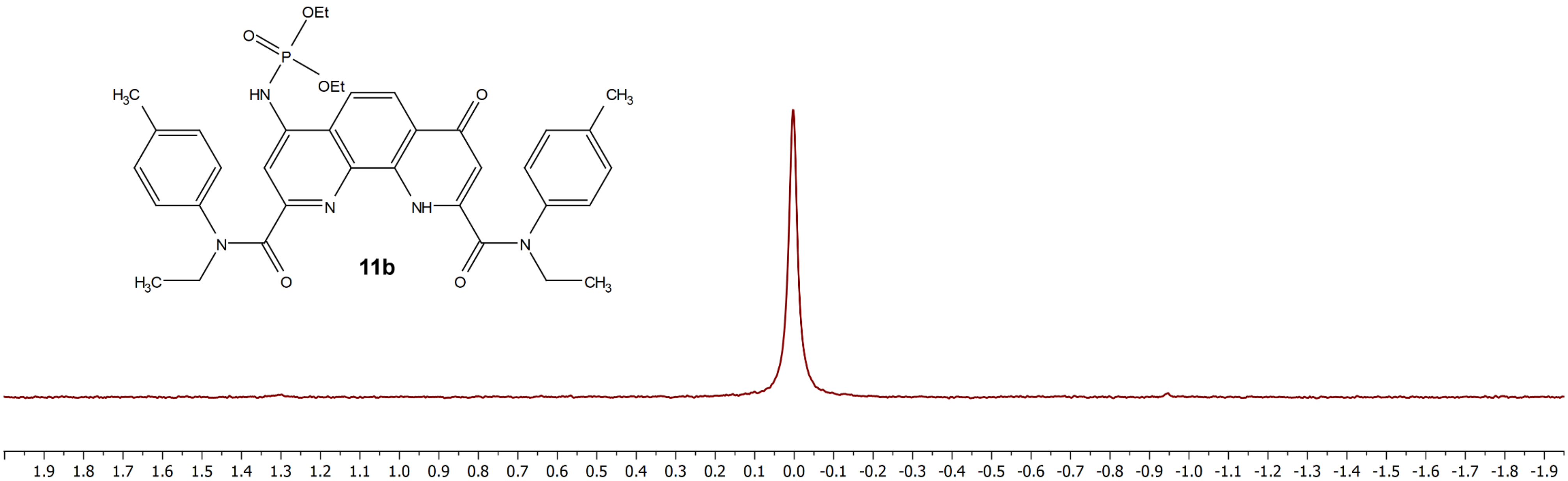

Figure S51. ^{31}P NMR spectrum of **11b** (101 MHz, $CDCl_3$)

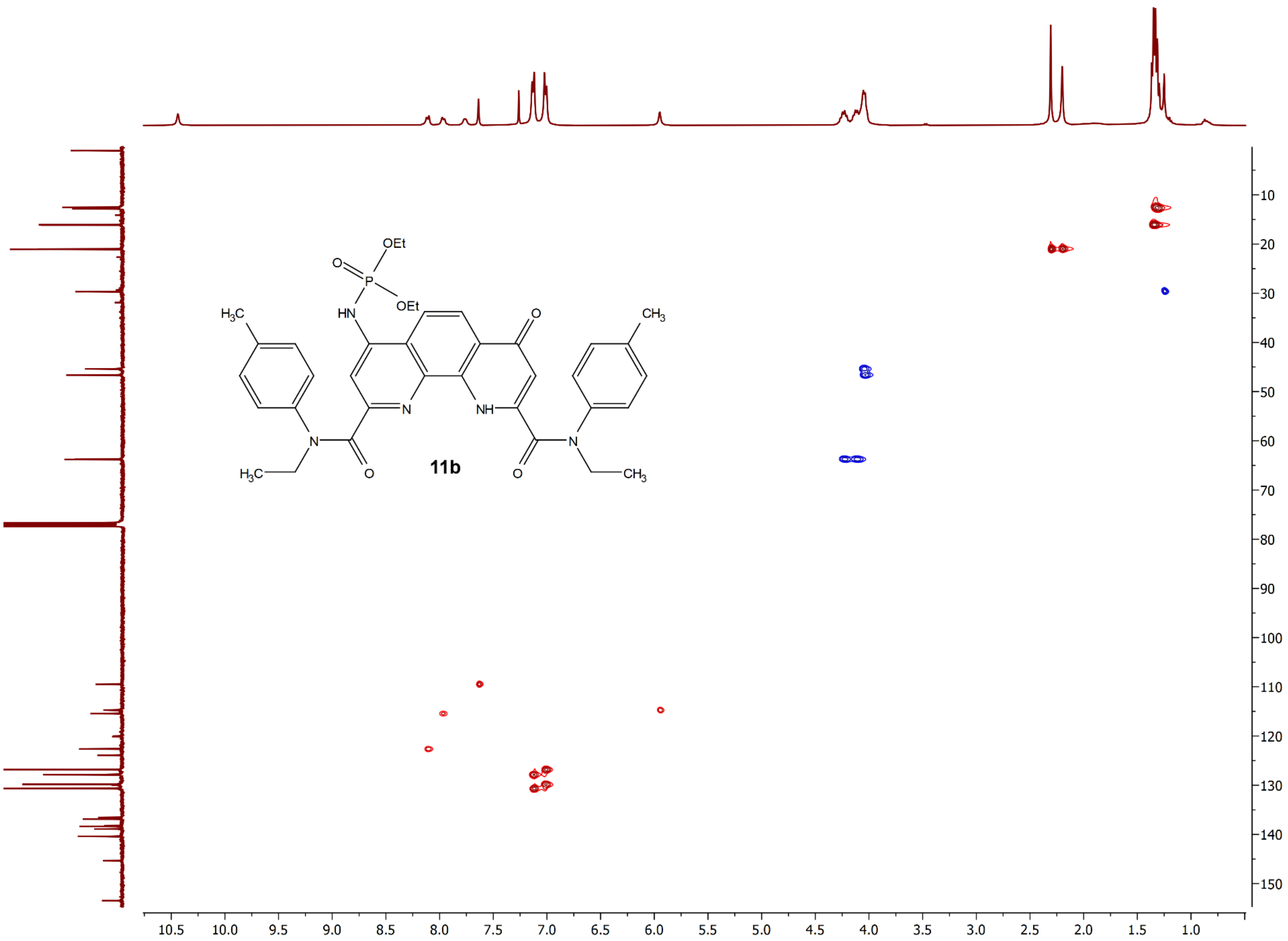

Figure S52. $^{1}H/^{13}C$ HSQC NMR spectrum of **11b** ($CDCl_3$)

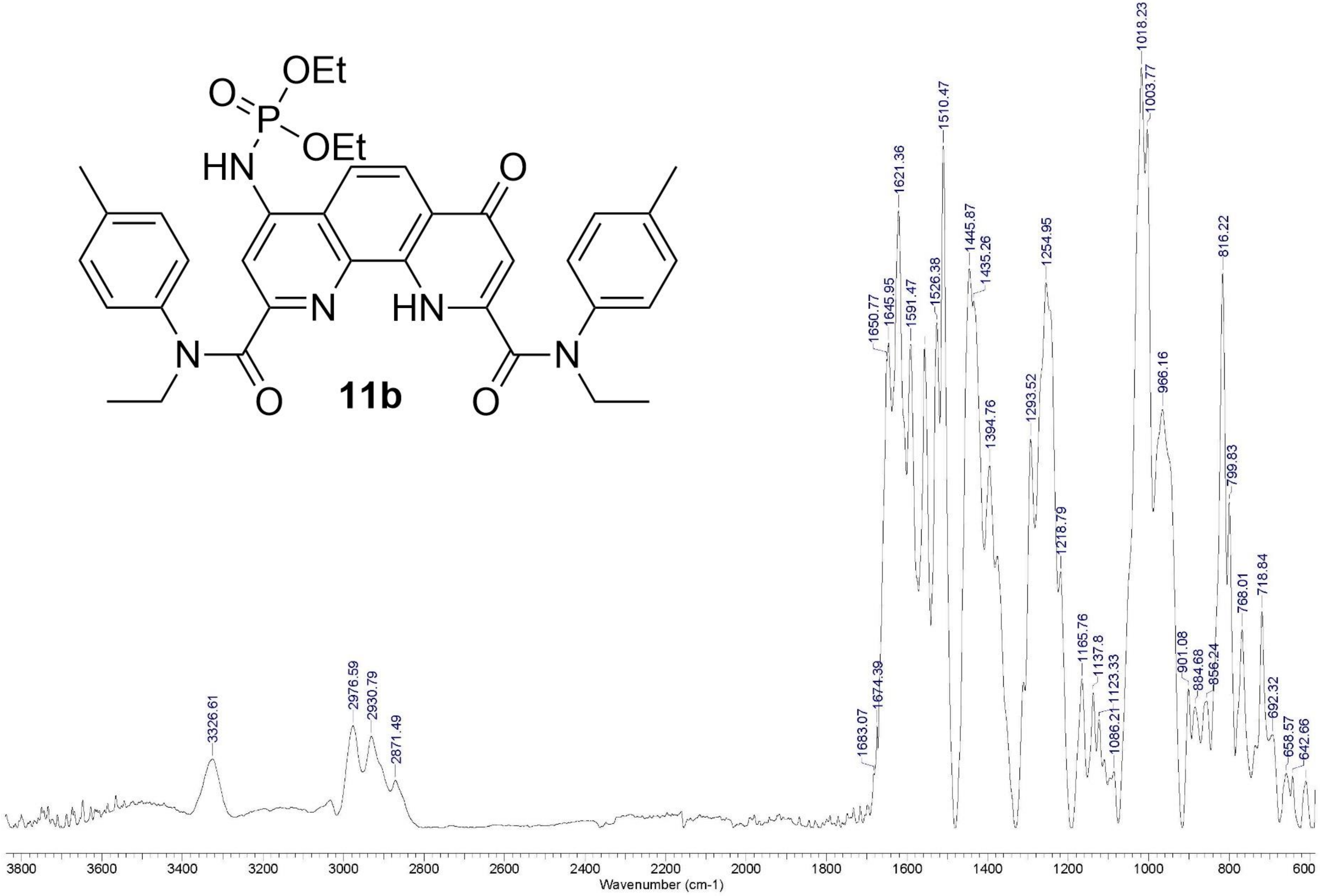

Figure S53. Solid-state IR spectrum of **11b**

7-(5-Amino-4-cyano-1H-1,2,3-triazol-1-yl)-N²,N⁹-diethyl-4-oxo-N²,N⁹-di-p-tolyl-1,4-dihydro-1,10-phenanthroline-2,9-dicarboxamide (12b)

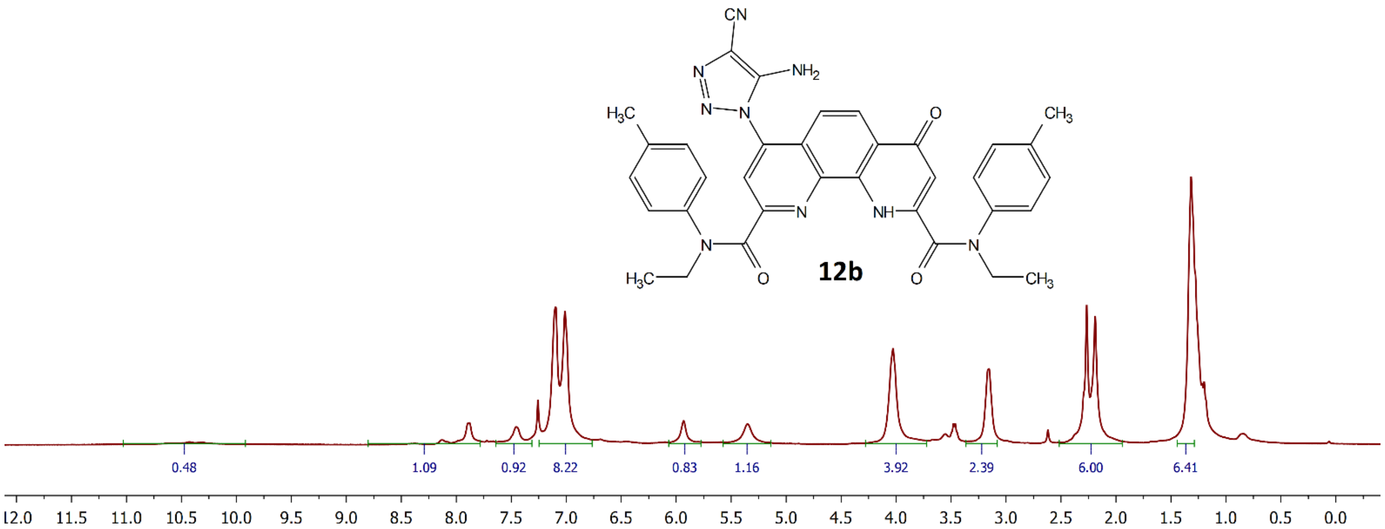

Figure S54. ^{1}H NMR spectrum of **12b** (400 MHz, $CDCl_3$)

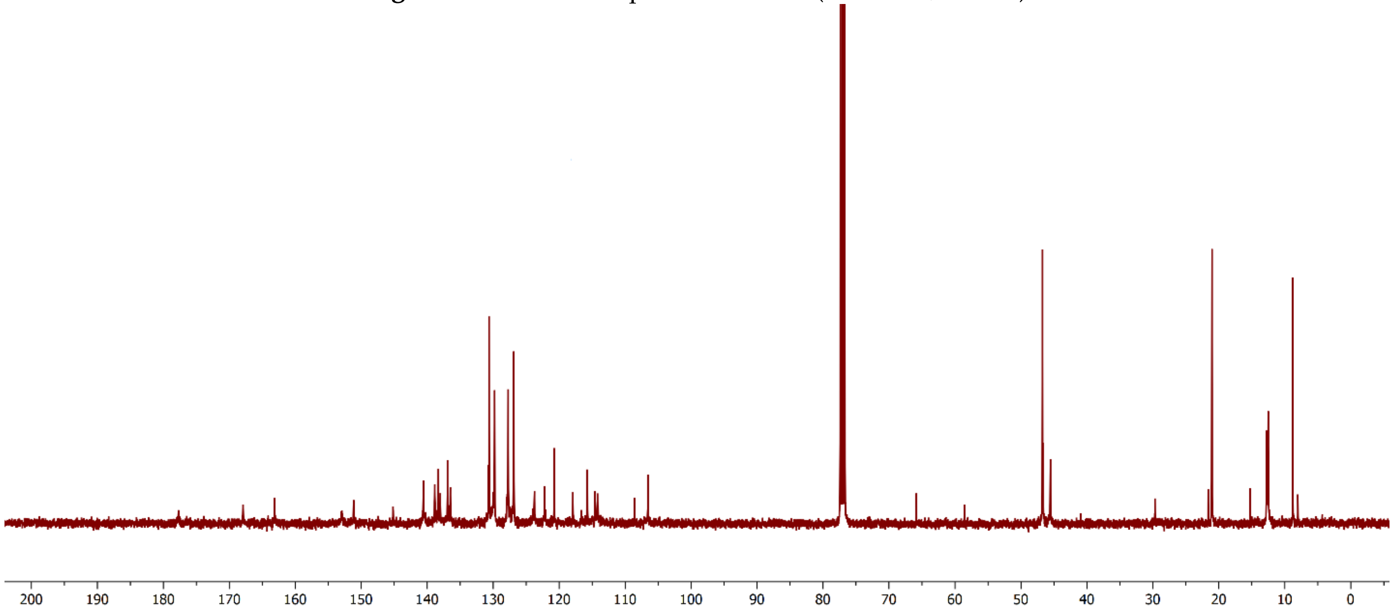

Figure S55. ^{1}H NMR spectrum of **12b** (400 MHz, $CDCl_3$)

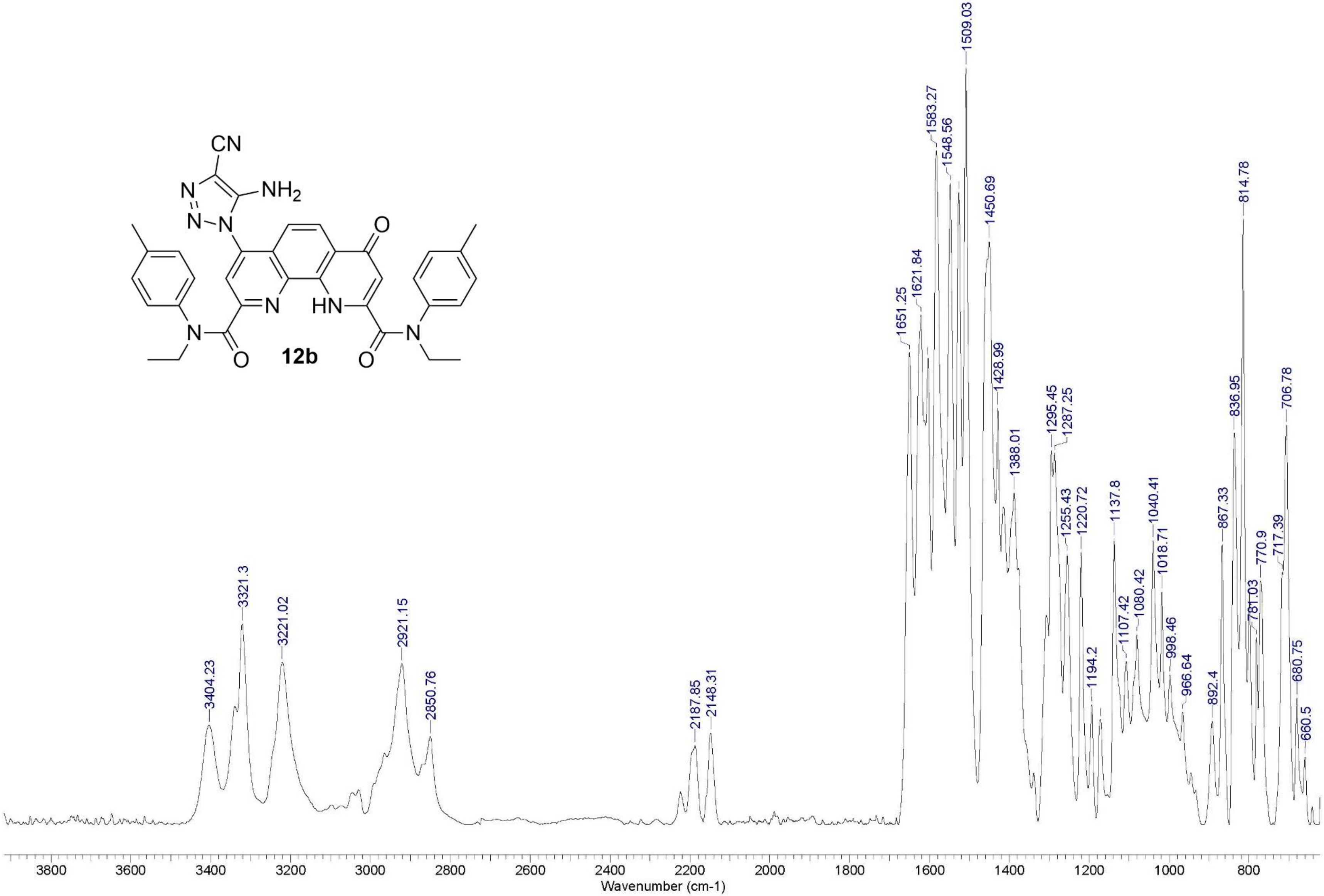

Figure S56. Solid-state IR spectrum of **12b**

N²,N⁹-Diethyl-7-(4,5,6,7,8,9-hexahydro-1H-cycloocta[d][1,2,3]triazol-1-yl)-4-oxo-N²,N⁹-di-p-tolyl-1,4-dihydro-1,10-phenanthroline-2,9-dicarboxamide (13b)

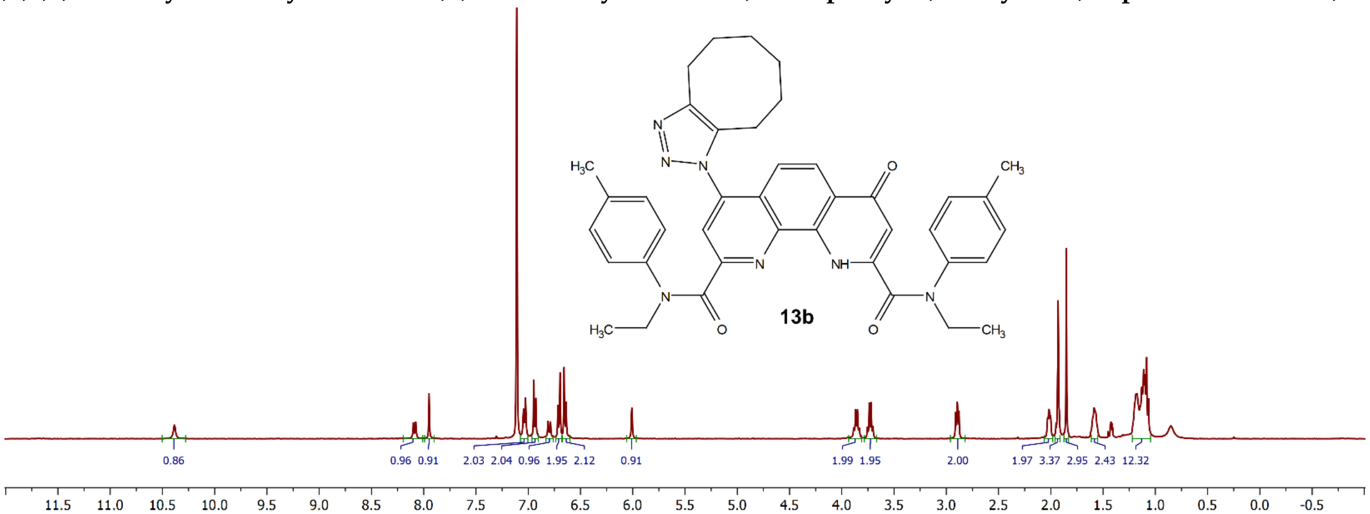

Figure S57. ^{1}H NMR spectrum of **13b** (400 MHz, C_6D_6)

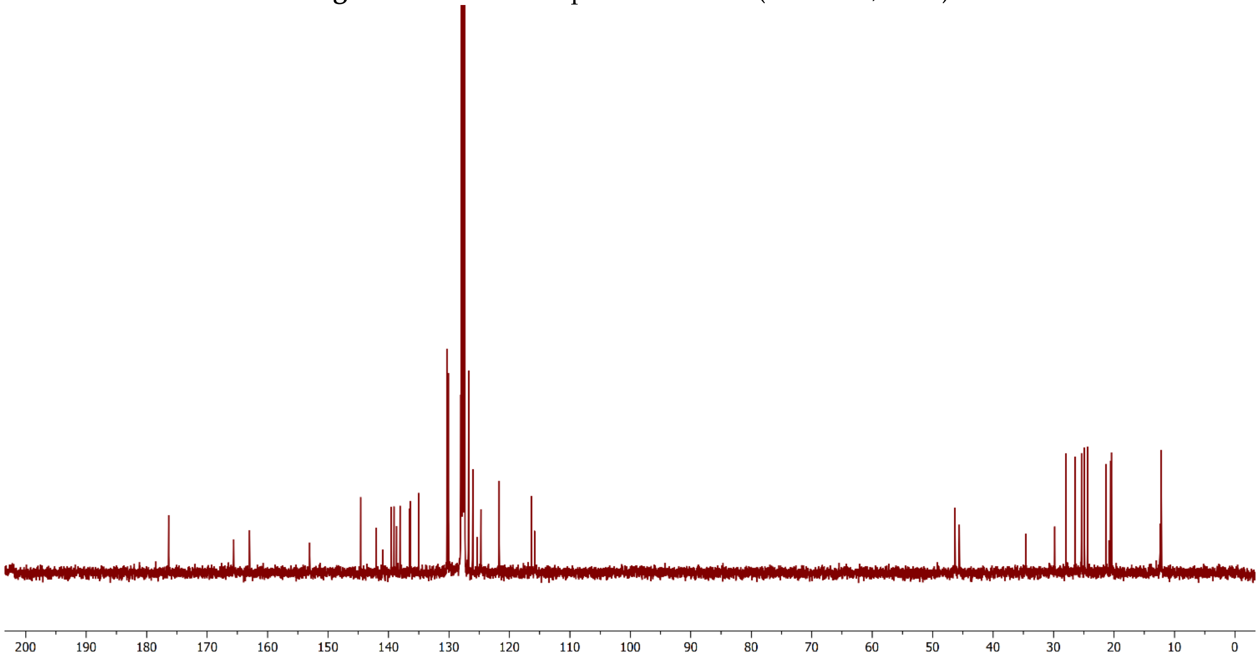

Figure S58. $^{13}C\{^{1}H\}$ NMR spectrum of **13b** (101 MHz, C_6D_6)

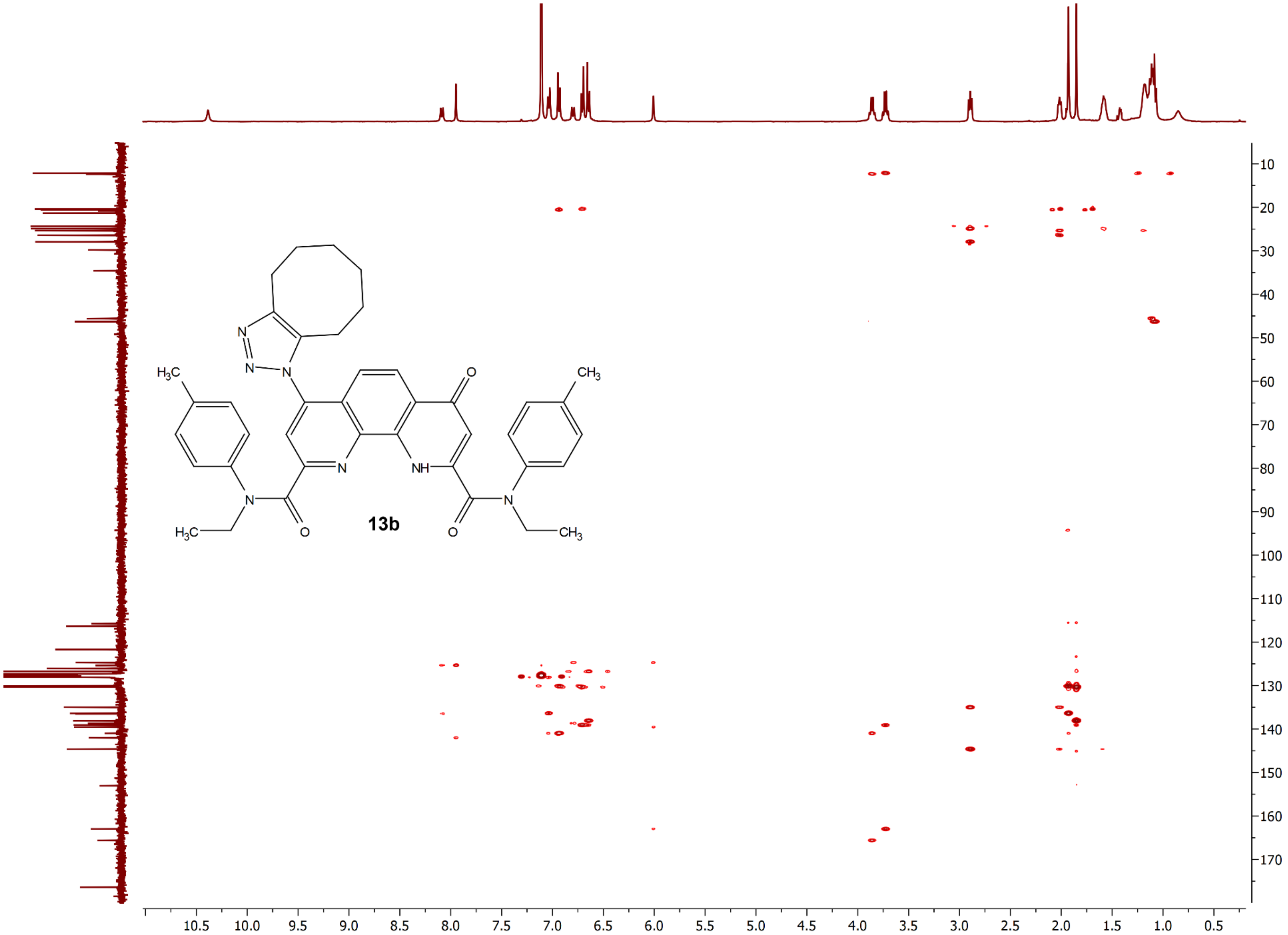

Figure S59. $^{1}H/^{13}C$ HMBC NMR spectrum of **13b** (C_6D_6)

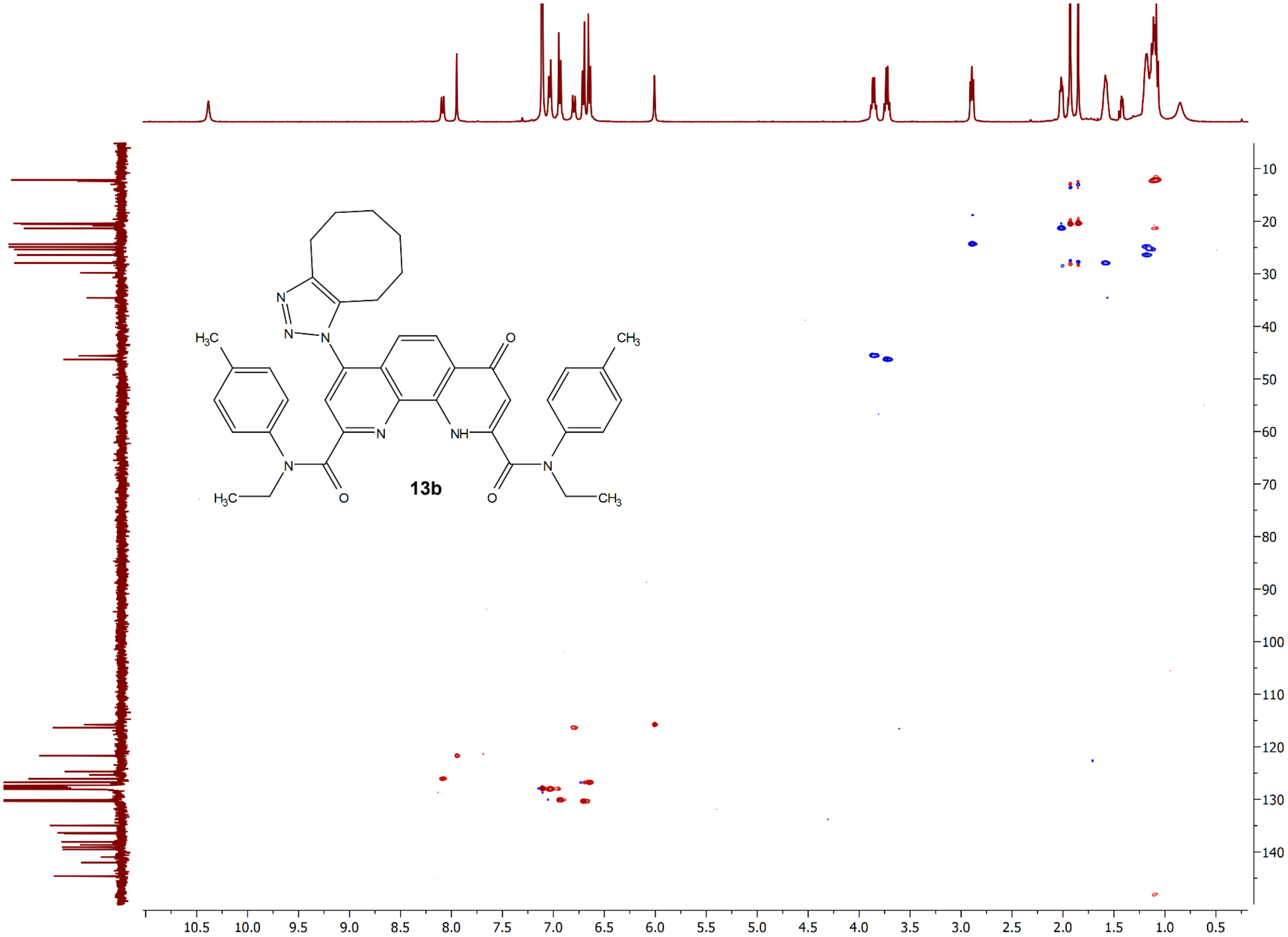

Figure S60. ¹H/¹³C HSQC NMR spectrum of **13b** (C_6D_6)

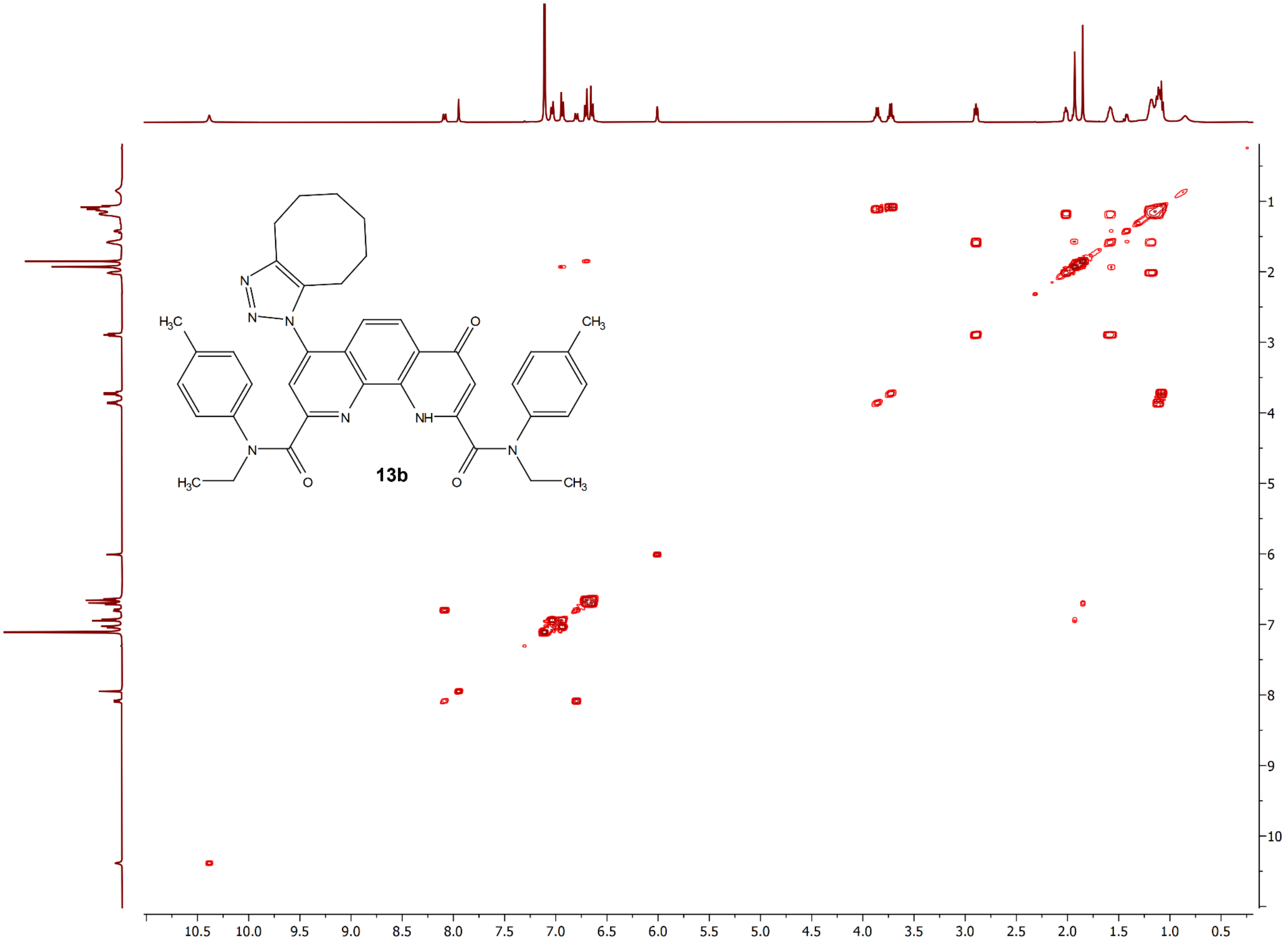

Figure S61. $^1H/^1H$ COSY NMR spectrum of **13b** (C_6D_6)

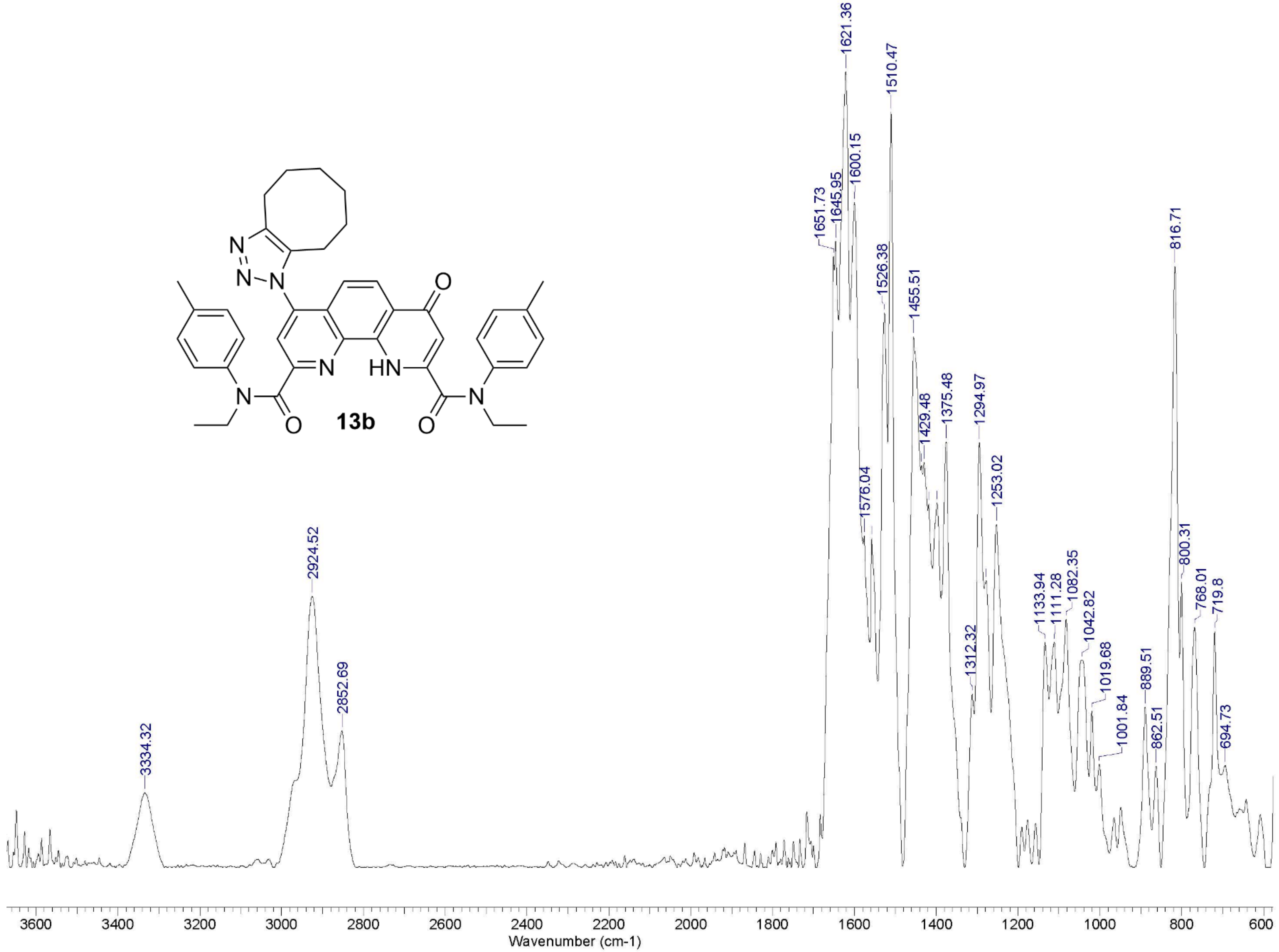

Figure S62. Solid-state IR spectrum of **13b**

2,9-Bis(ethyl(p-tolyl)carbamoyl)-7-oxo-7,10-dihydro-1,10-phenanthrolin-4-yl acetate (14b)

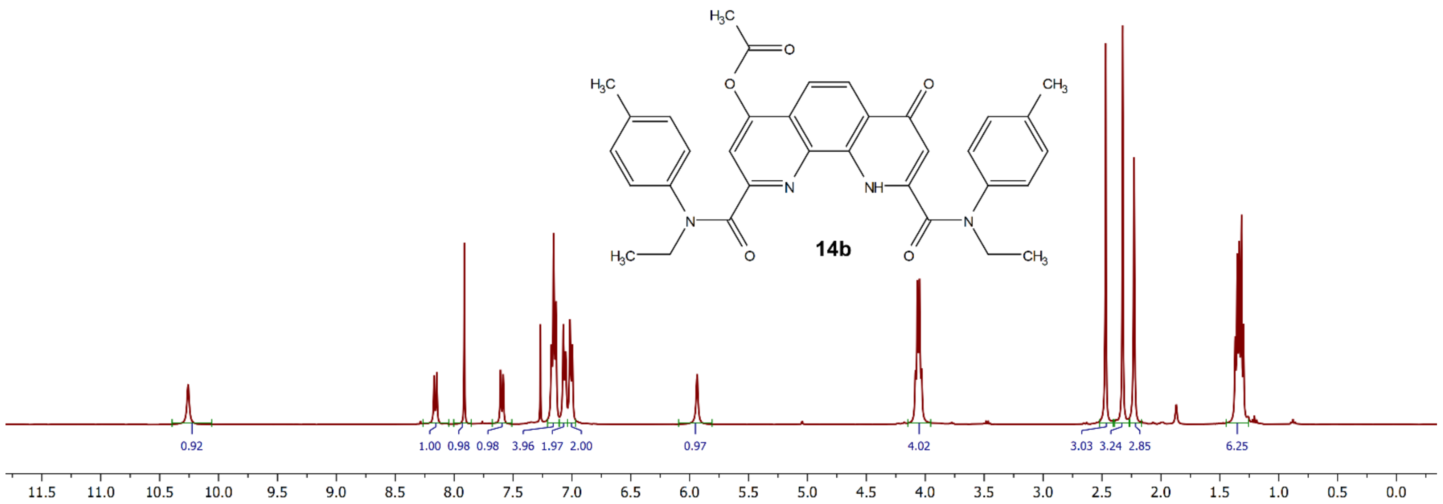

Figure S63. ^{1}H NMR spectrum of **14b** (400 MHz, $CDCl_3$)

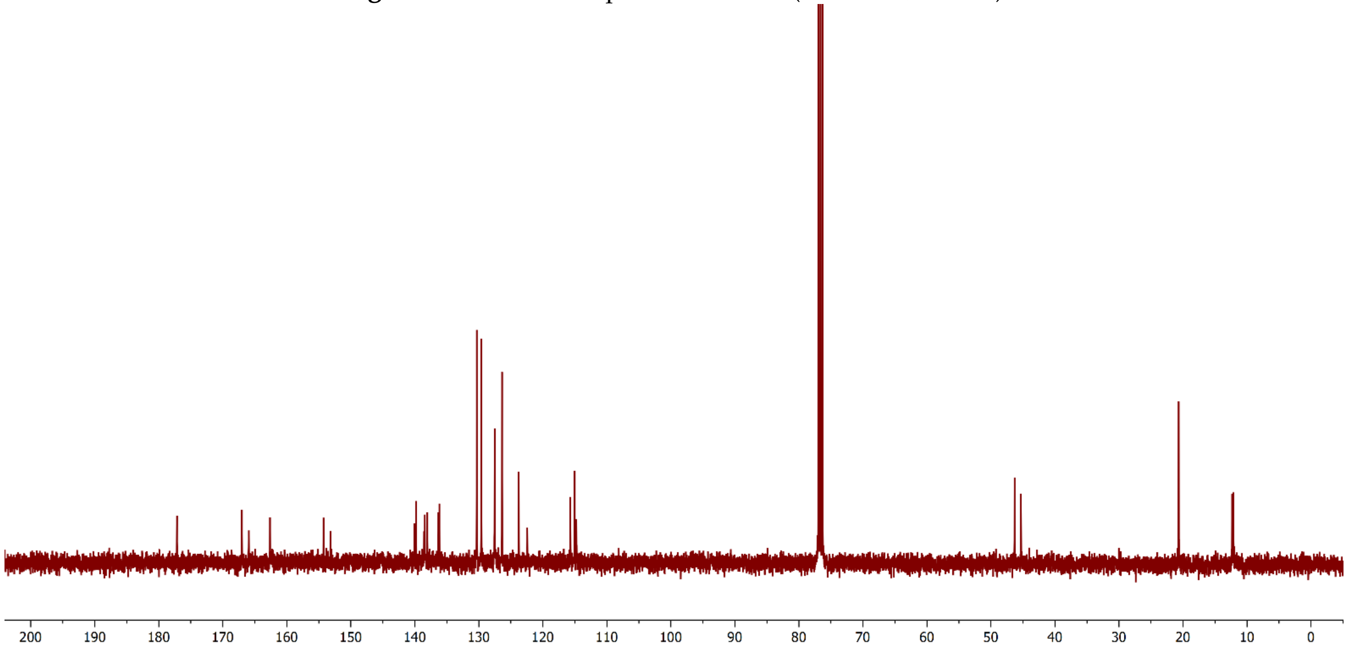

Figure S64. $^{13}C\{^{1}H\}$ NMR spectrum of **14b** (101 MHz, $CDCl_3$)

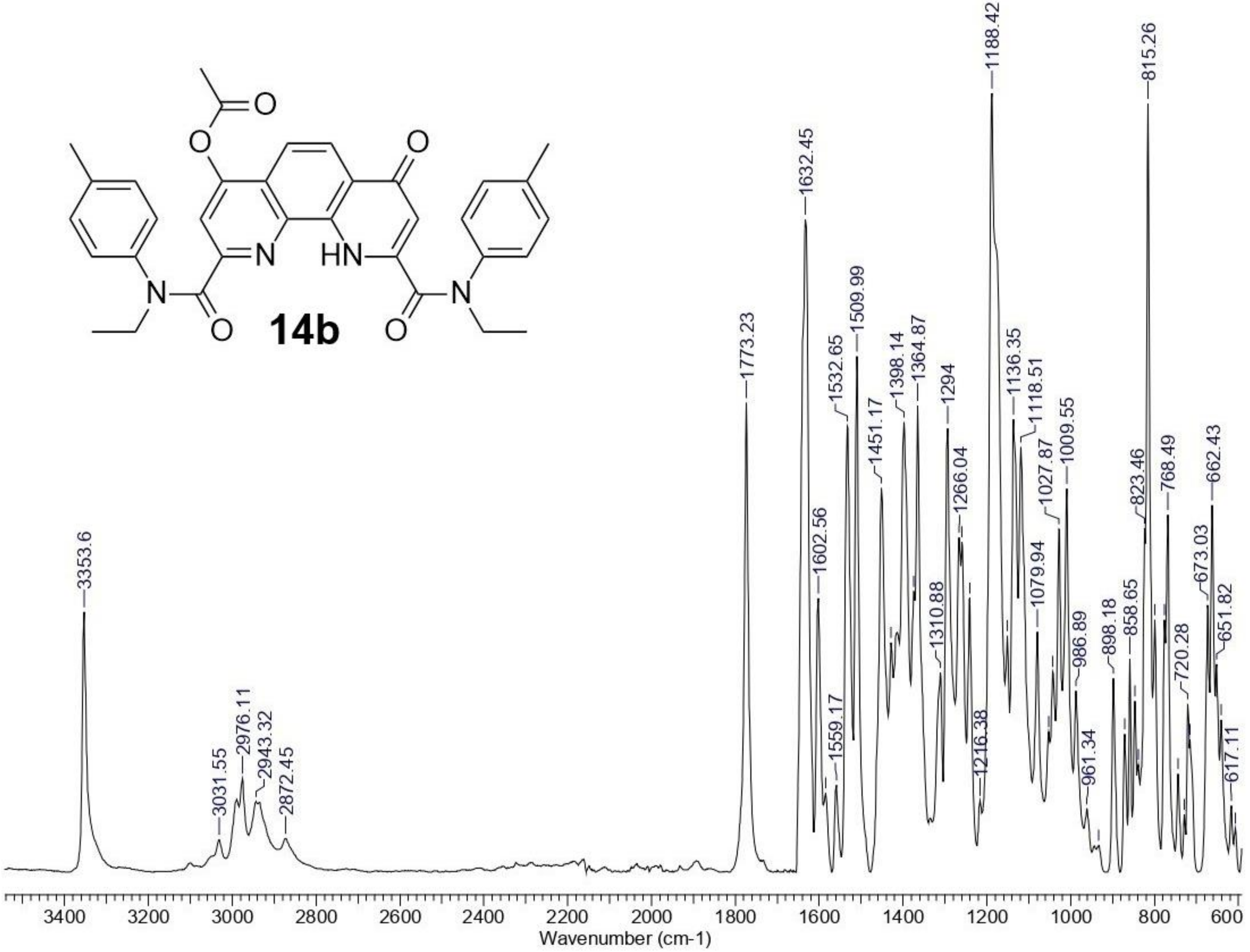

Figure S65. Solid-state IR spectrum of **14b**

2,9-Bis(ethyl(p-tolyl)carbamoyl)-1,10-phenanthroline-4,7-diyl bis(4-methylbenzenesulfonate) (15b)

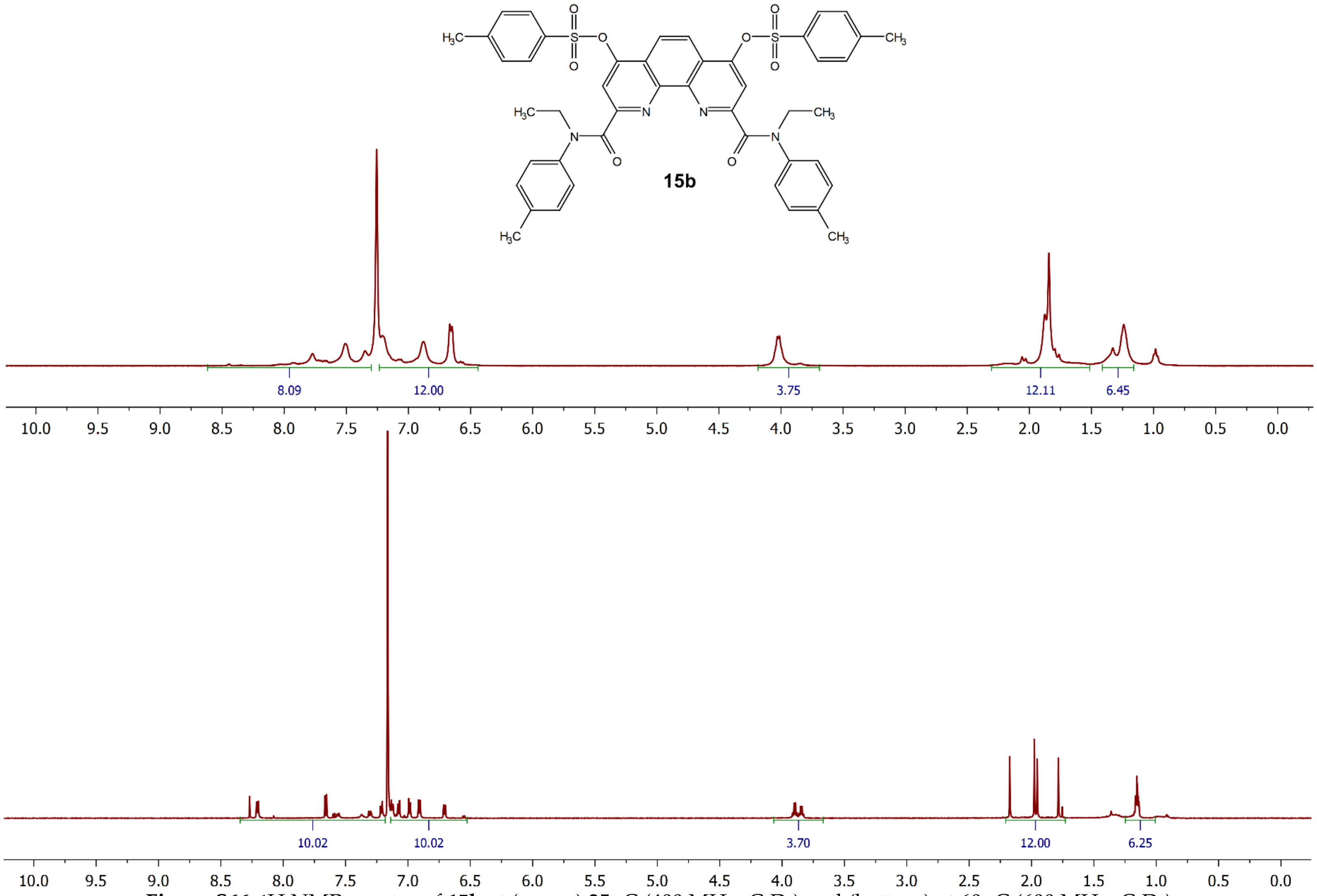

Figure S66. ^{1}H NMR spectra of **15b** at (upper) 25 ^{o}C (400 MHz, C_6D_6) and (bottom) at 60 ^{o}C (600 MHz, C_6D_6)

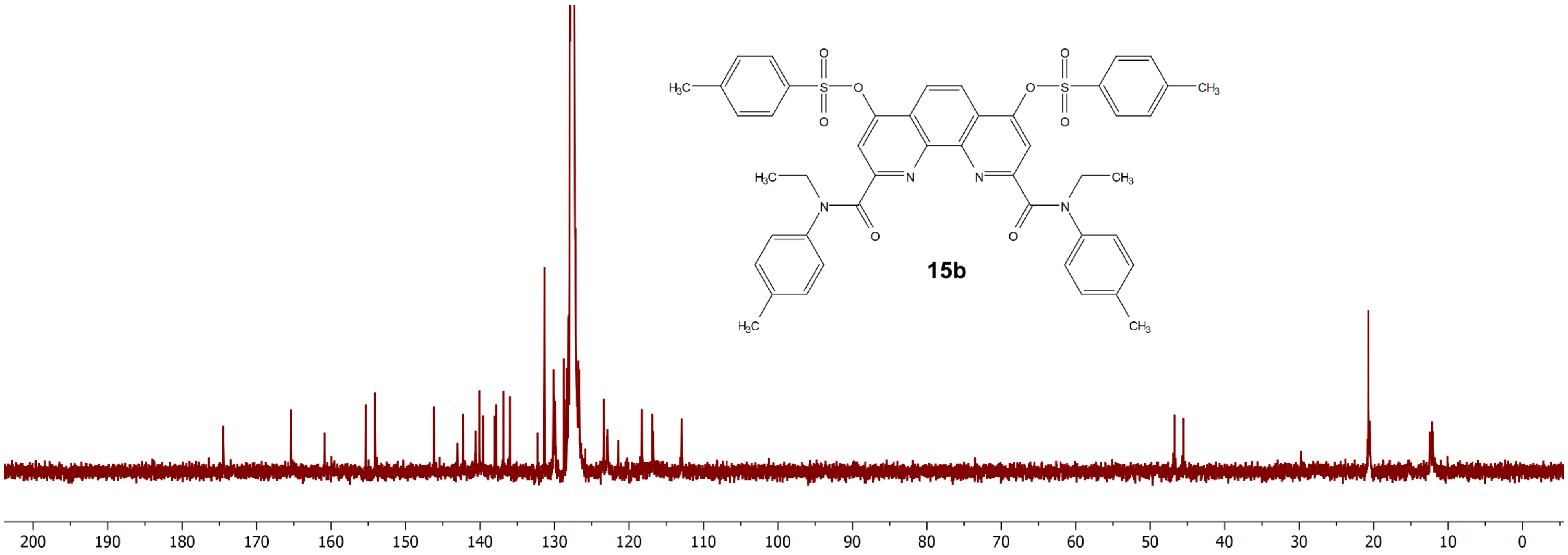

Figure S67. $^{13}C\{^{1}H\}$ NMR spectrum of **15b** at 60 °C (151 MHz, C_6D_6)

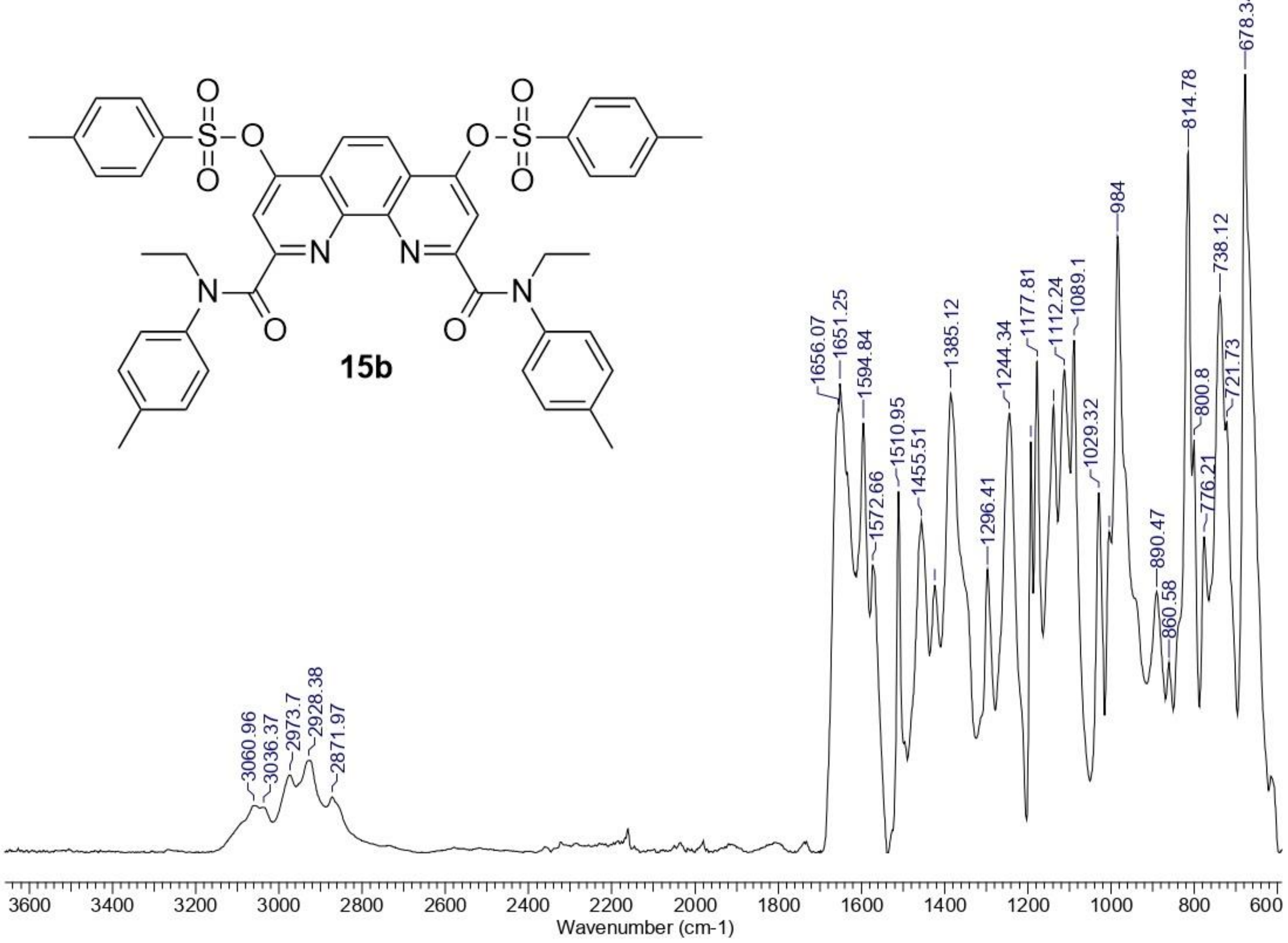

Figure S68. Solid-state IR spectrum of **15b**

N^2,N^2,N^9,N^9-Tetrabutyl-4-fluoro-7-methoxy-1,10-phenanthroline-2,9-dicarboxamide (17a)

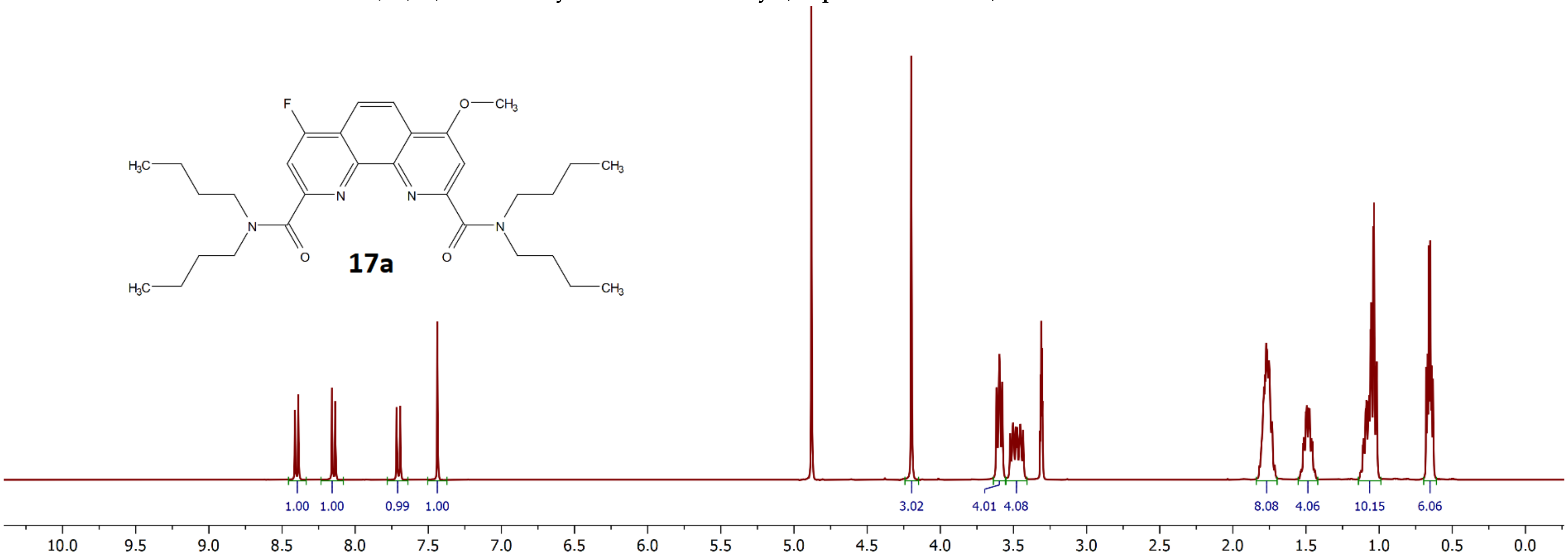

Figure S69. ^{1}H NMR spectrum of **17a** (400 MHz, CD_3OD)

N[2],*N*[2],*N*[9],*N*[9]-Tetrabutyl-4-chloro-7-methoxy-1,10-phenanthroline-2,9-dicarboxamide (18a)

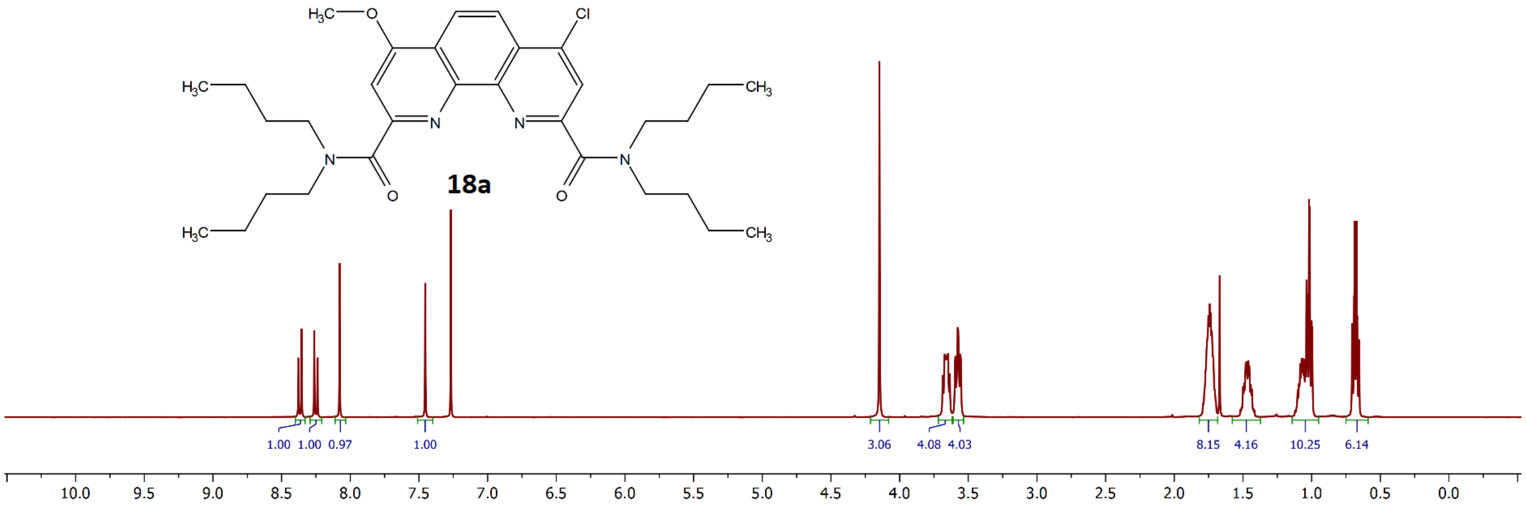

Figure S70. ^{1}H NMR spectrum of **18a** (400 MHz, $CDCl_3$)

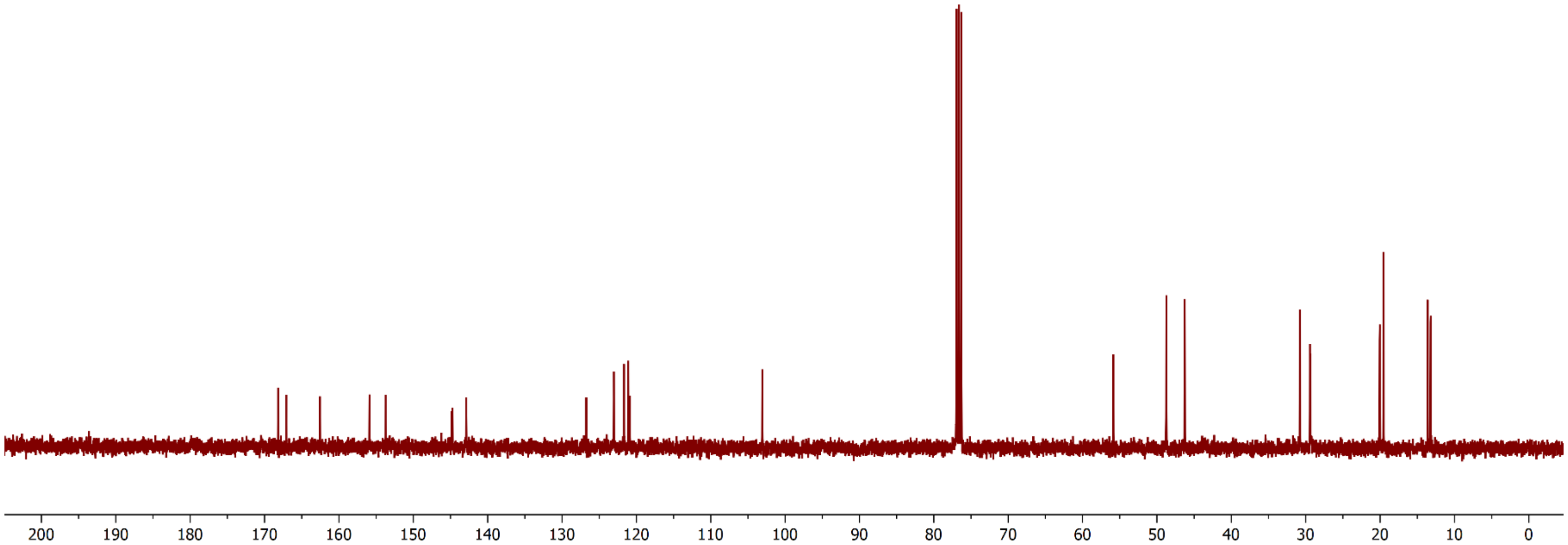

Figure S71. $^{13}C\{^{1}H\}$ NMR spectrum of **18a** (101 MHz, $CDCl_3$)

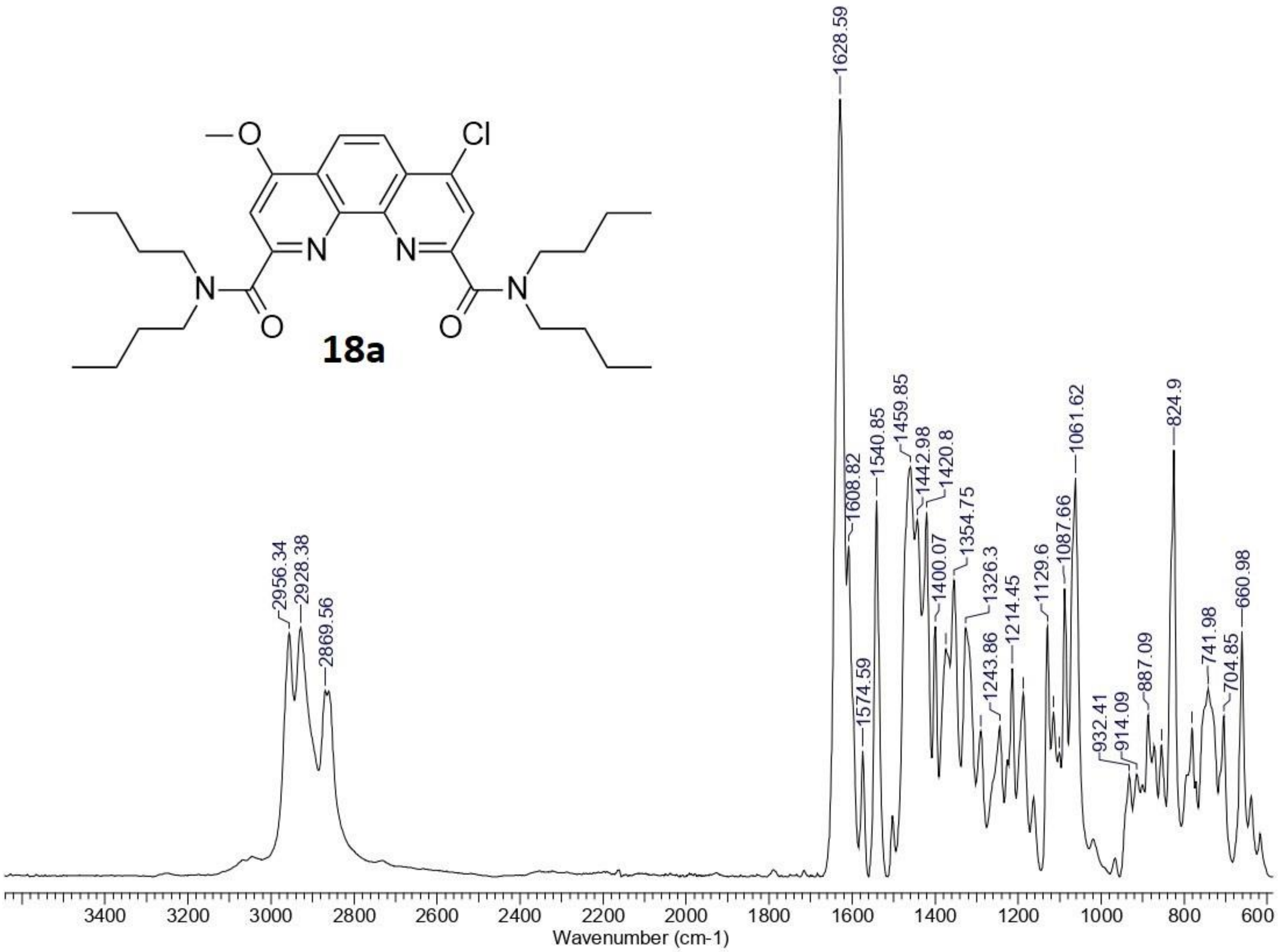

Figure S72. Solid-state IR spectrum of **18a**

***N*²,*N*²,*N*⁹,*N*⁹-Tetrabutyl-4-fluoro-7-(prop-2-yn-1-yloxy)-1,10-phenanthroline-2,9-dicarboxamide (19a)**

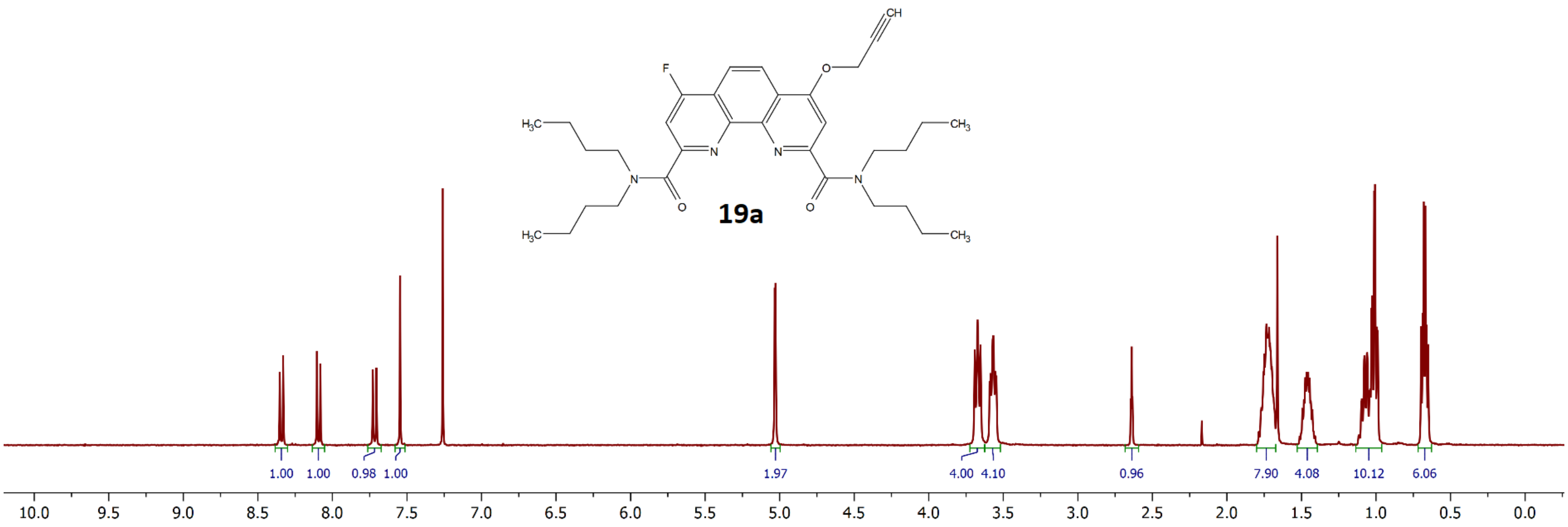

Figure S73. ^{1}H NMR spectrum of **19a** (400 MHz, $CDCl_3$)

N^2,N^2,N^9,N^9-Tetrabutyl-4-chloro-7-(prop-2-yn-1-yloxy)-1,10-phenanthroline-2,9-dicarboxamide (20a)

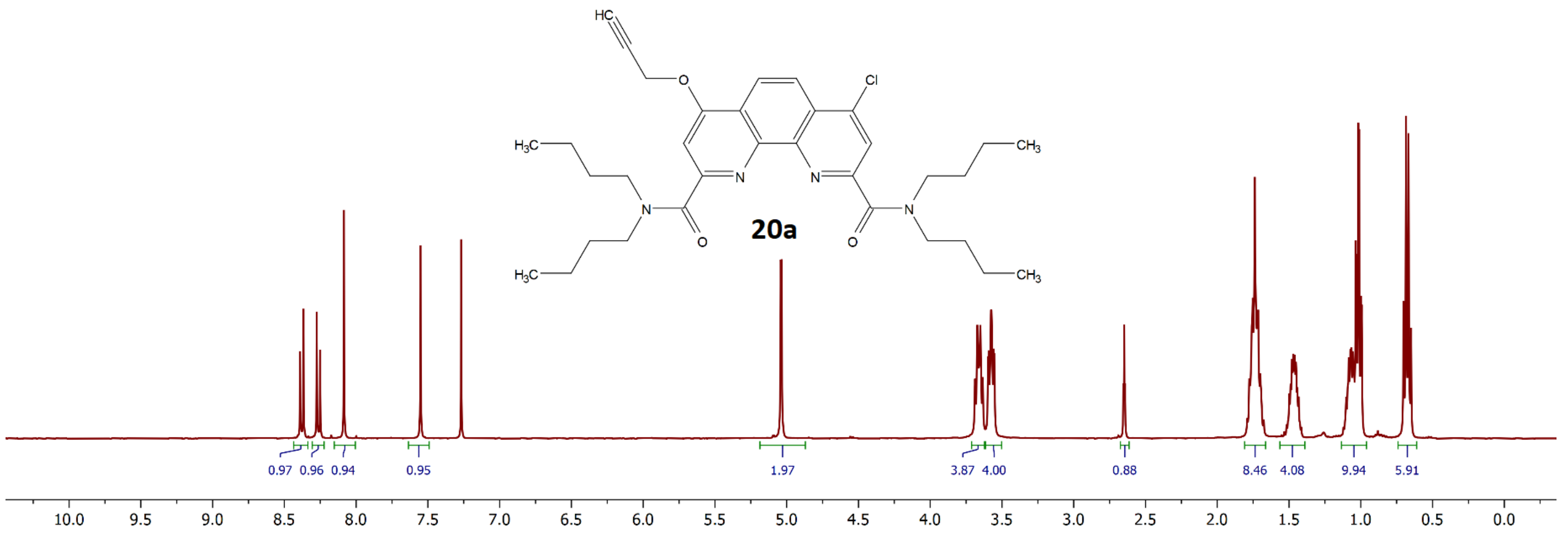

Figure S74. ^{1}H NMR spectrum of **20a** (400 MHz, $CDCl_3$)

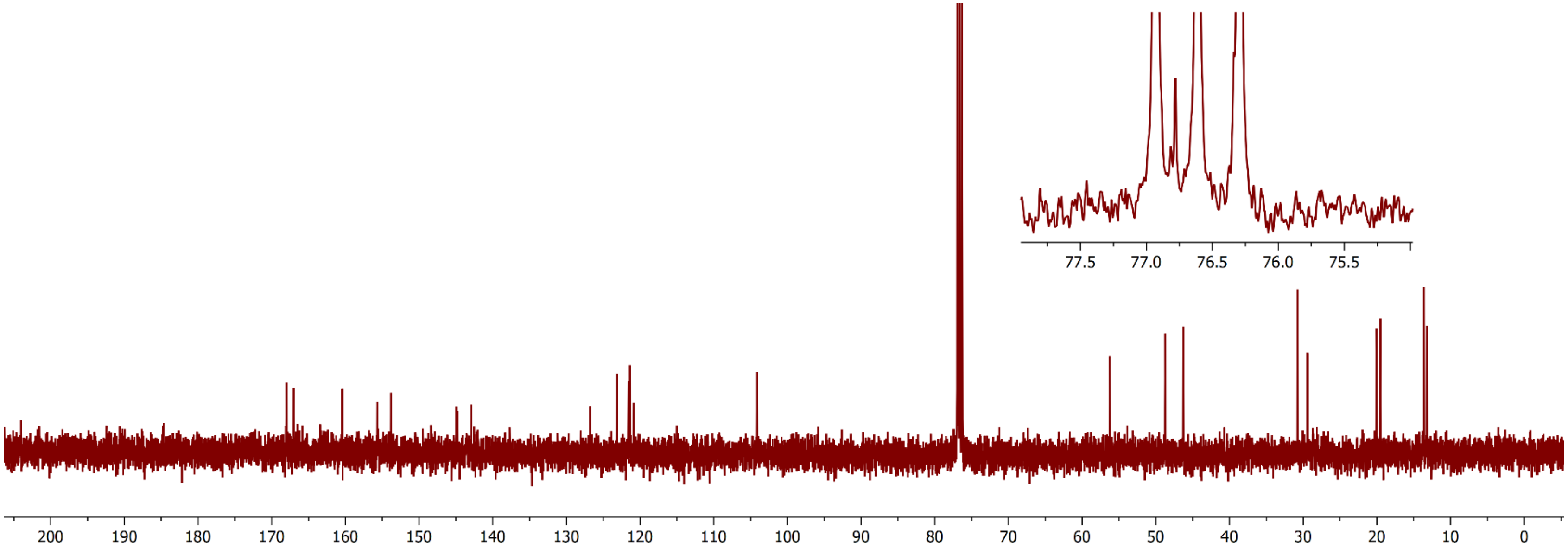

Figure S75. $^{13}C\{^{1}H\}$ NMR spectrum of **20a** (101 MHz, $CDCl_3$)

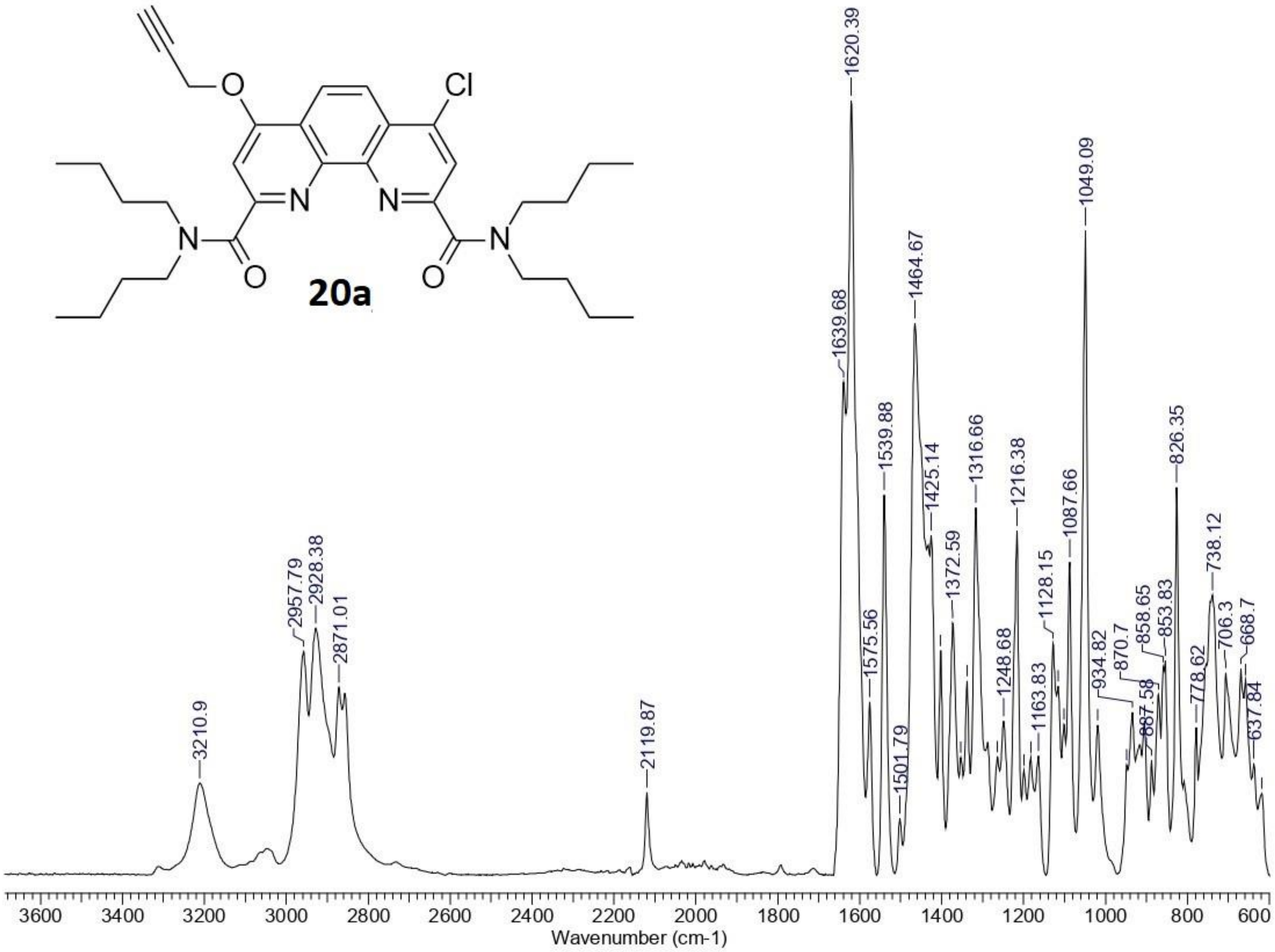

Figure S76. Solid-state IR spectrum of **20a**

2,9-Bis(dibutylcarbamoyl)-7-fluoro-1,10-phenanthrolin-4-yl acetate (21a)

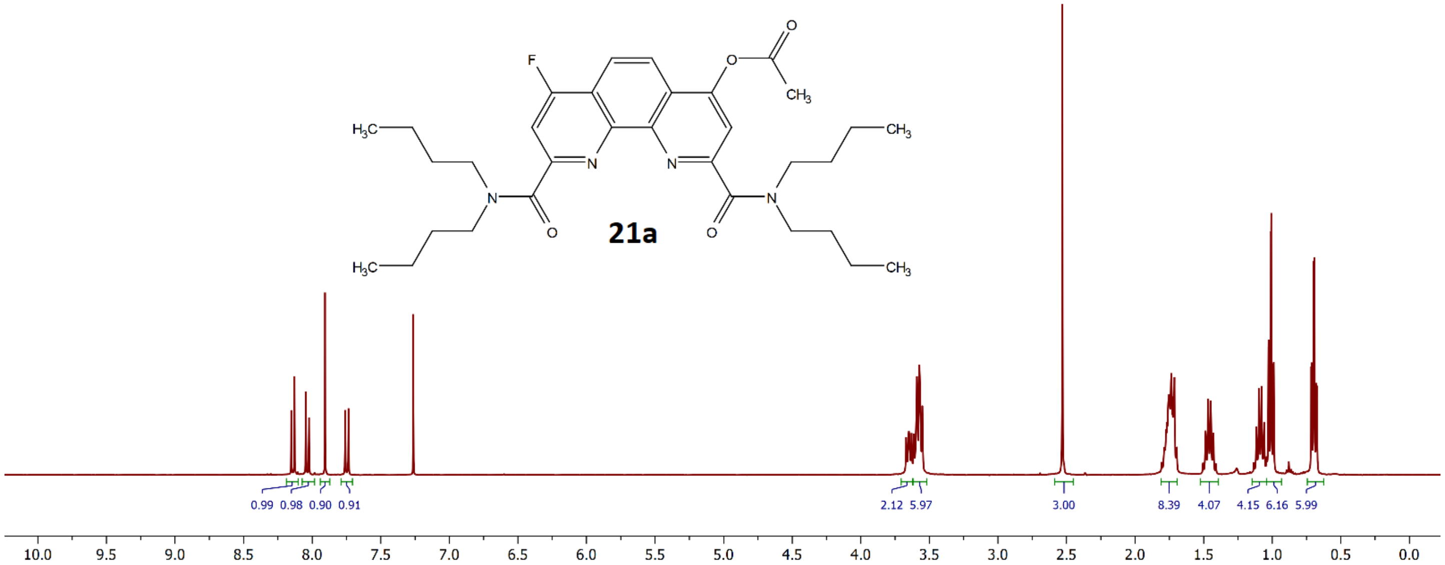

Figure S77. ^{1}H NMR spectrum of **21a** (400 MHz, $CDCl_3$)

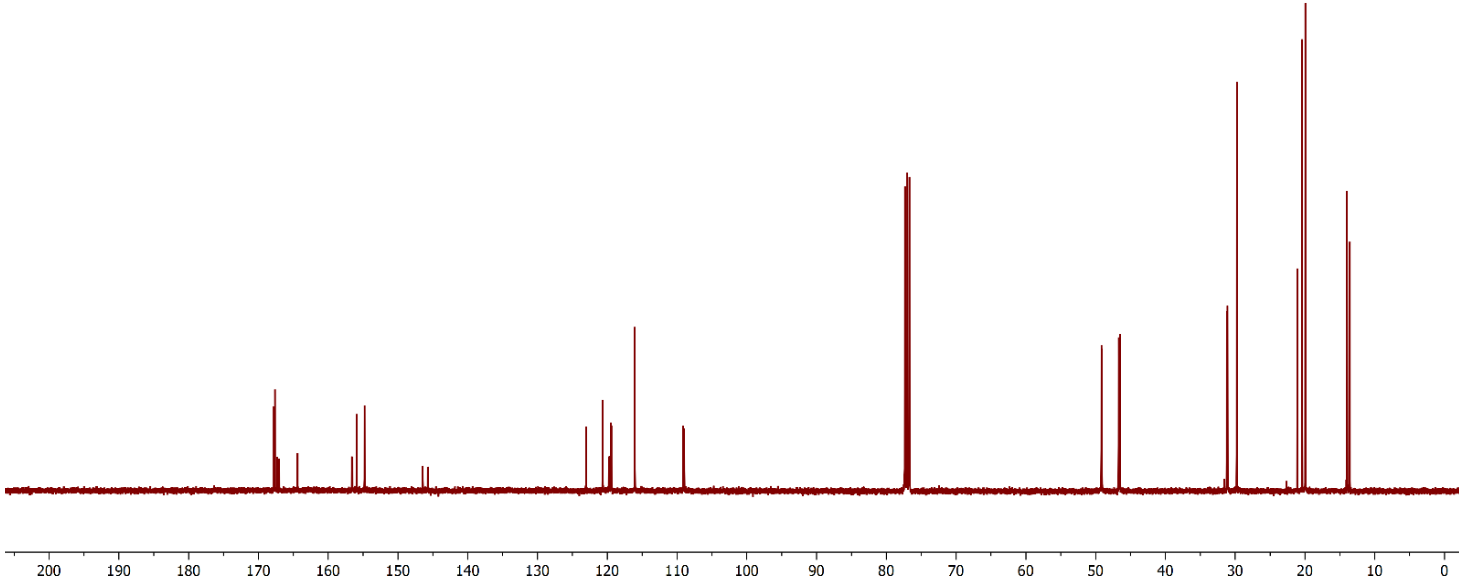

Figure S78. $^{13}C\{^{1}H\}$ NMR spectrum of **21a** (101 MHz, $CDCl_3$)

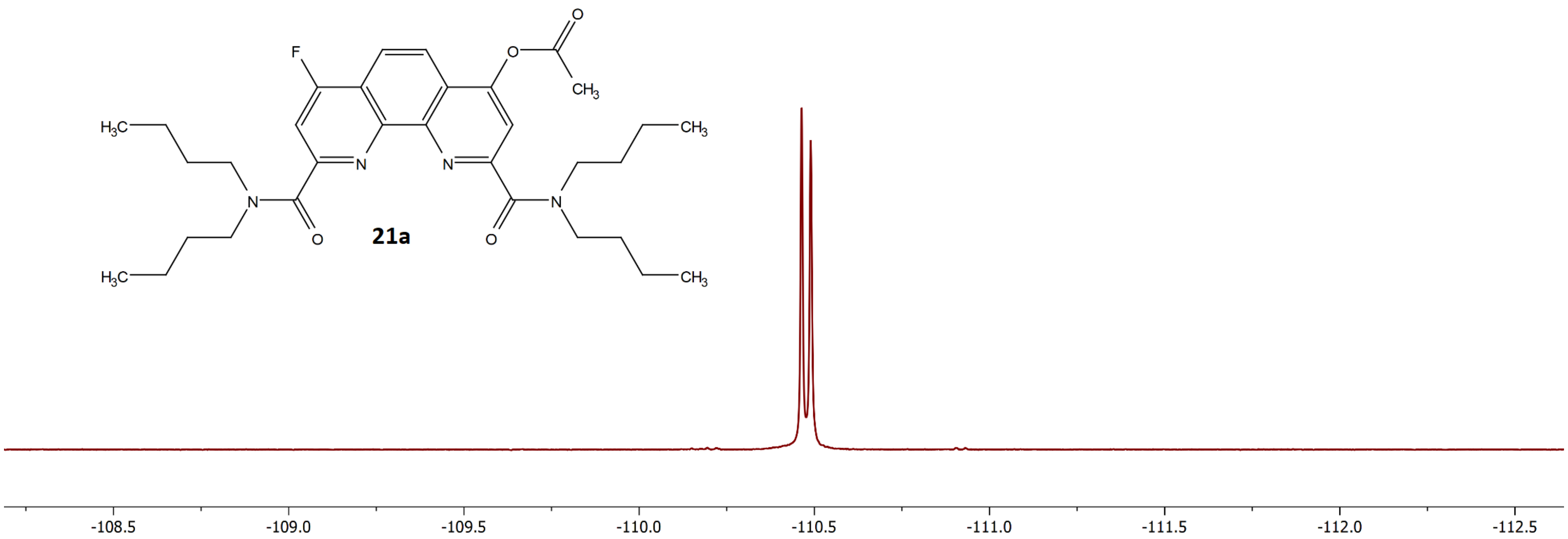

Figure S79. ^{19}F NMR spectrum of **21a** (376 MHz, $CDCl_3$)

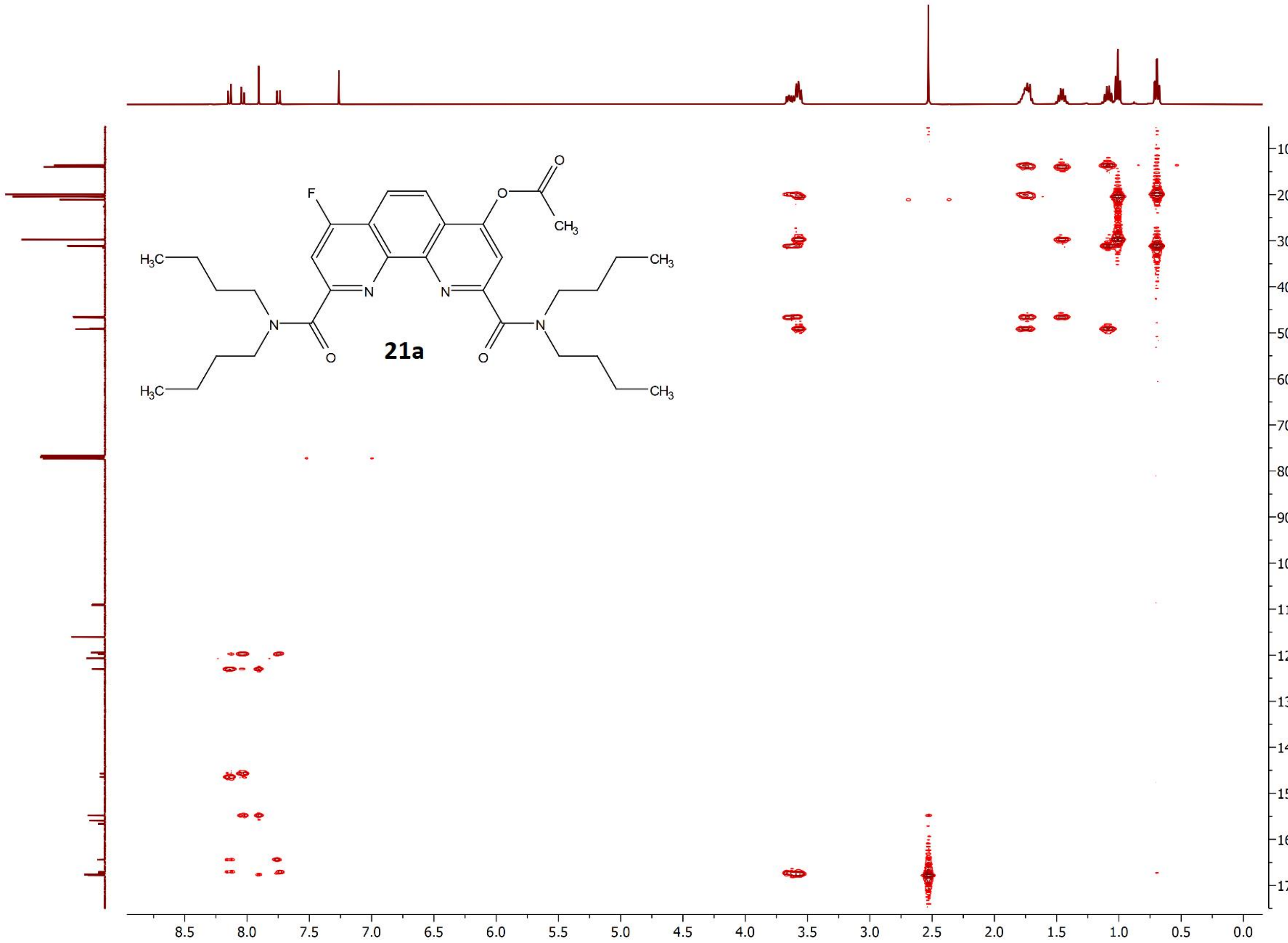

Figure S80. $^{1}H/^{13}C$ HMBC NMR spectrum of **21a** ($CDCl_3$)

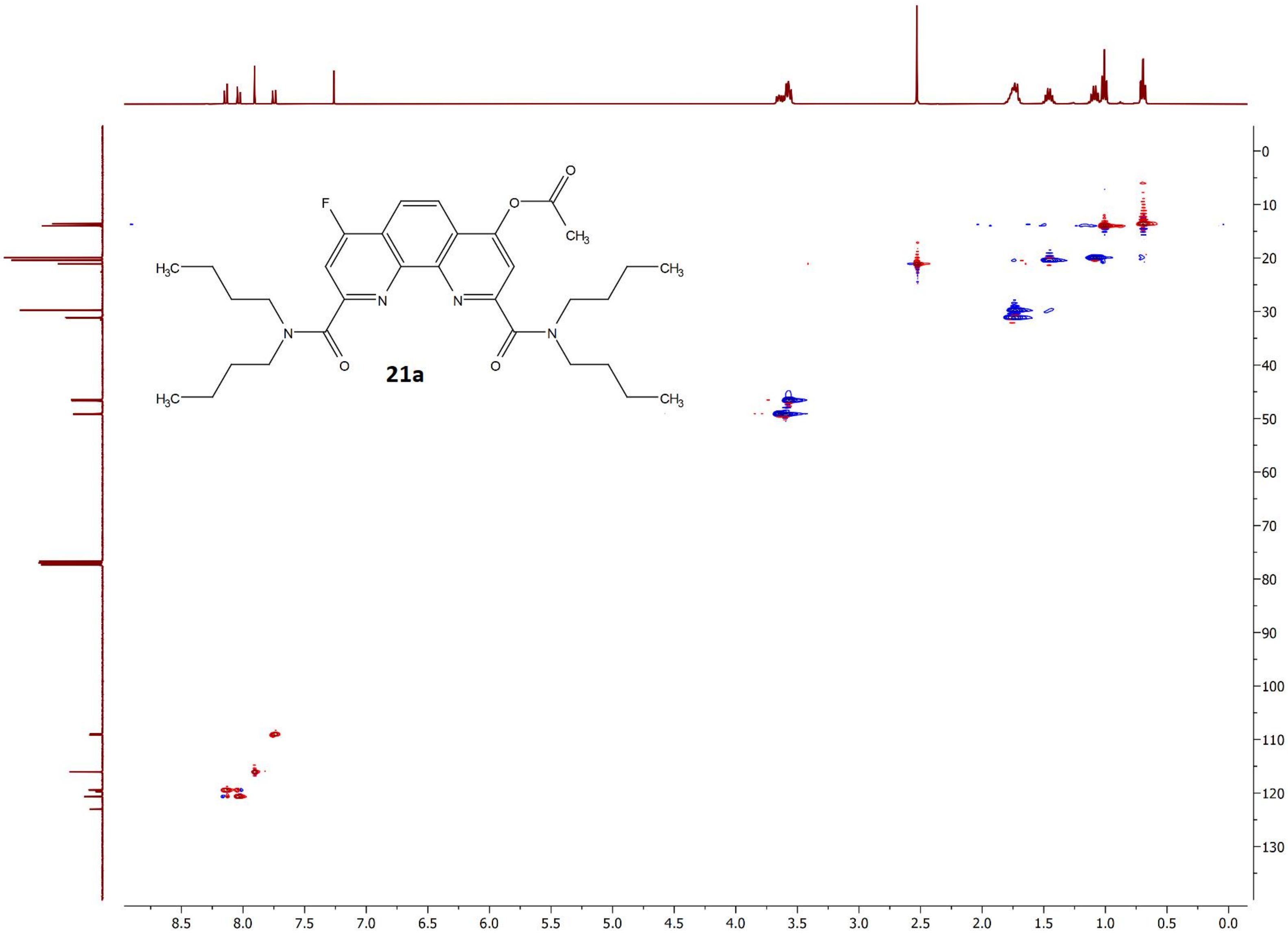

Figure S81. $^{1}H/^{13}C$ HSQC NMR spectrum of **21a** ($CDCl_3$)

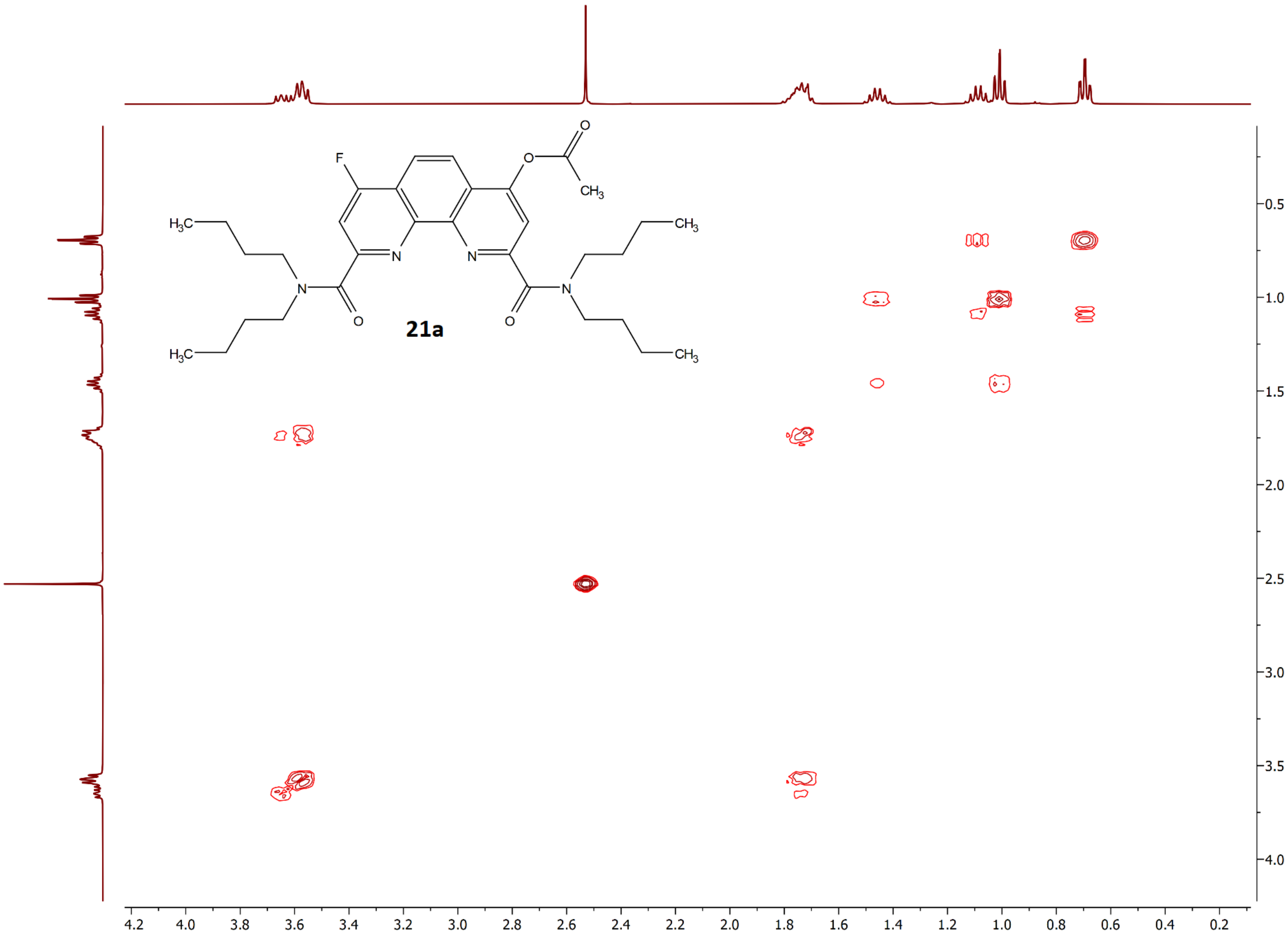

Figure S82. $^1H/^1H$ COSY NMR spectrum of **21a** ($CDCl_3$)

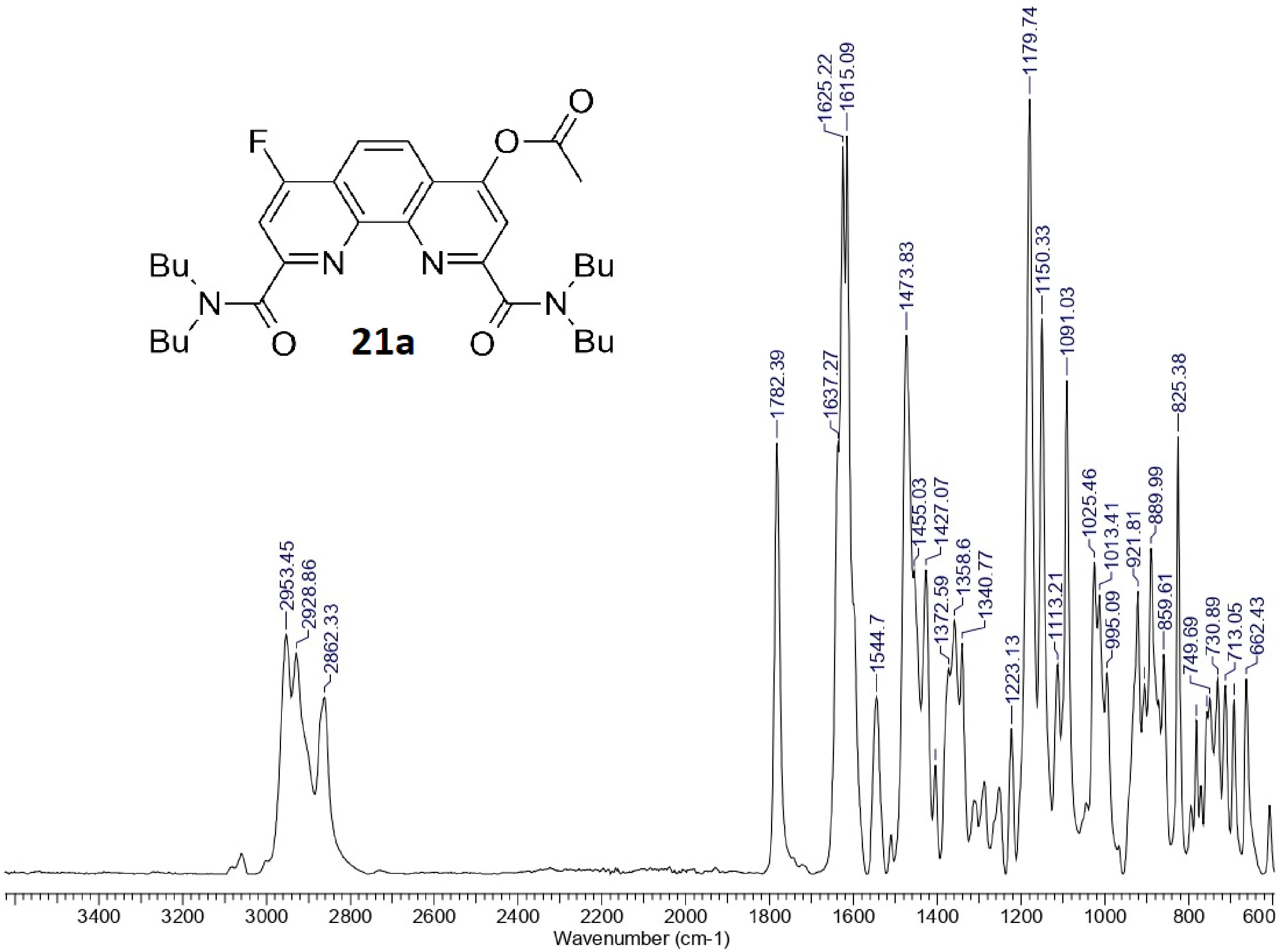

Figure S83. Solid-state IR spectrum of **21a**

4-(Benzyloxy)-N²,N²,N⁹,N⁹-tetrabutyl-7-chloro-1,10-phenanthroline-2,9-dicarboxamide (22a)

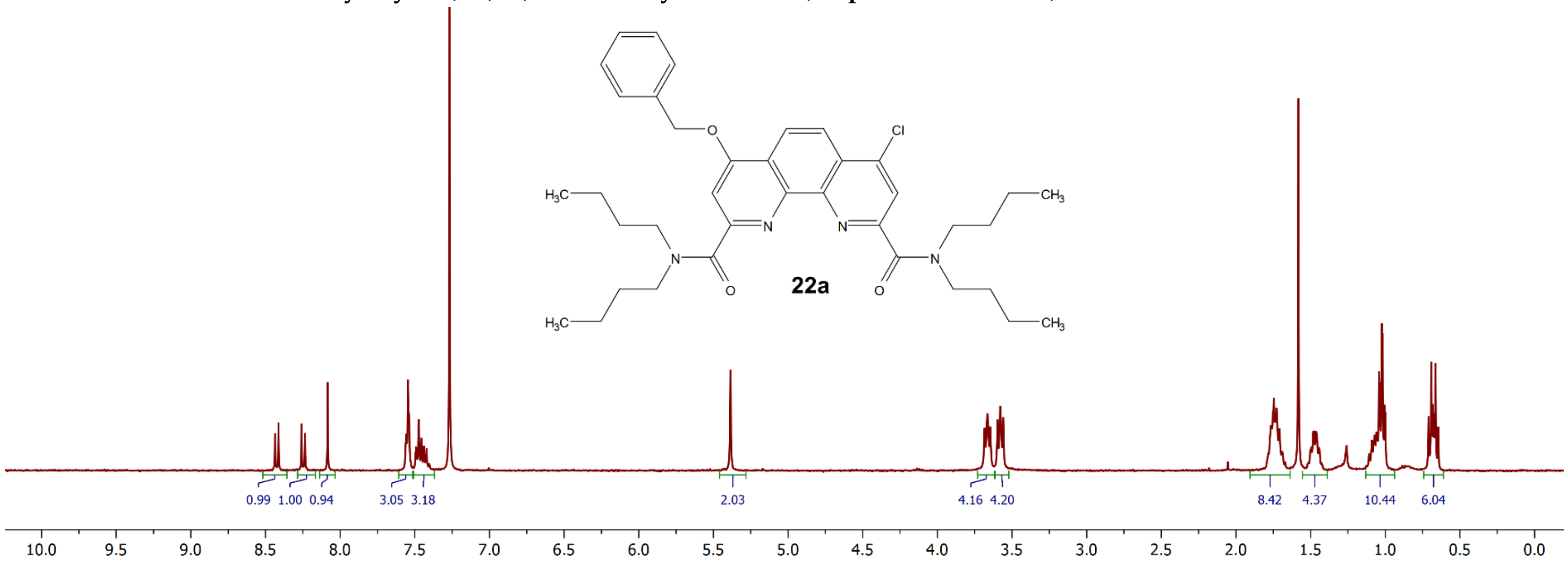

Figure S84. ^{1}H NMR spectrum of **22a** (400 MHz, $CDCl_3$)

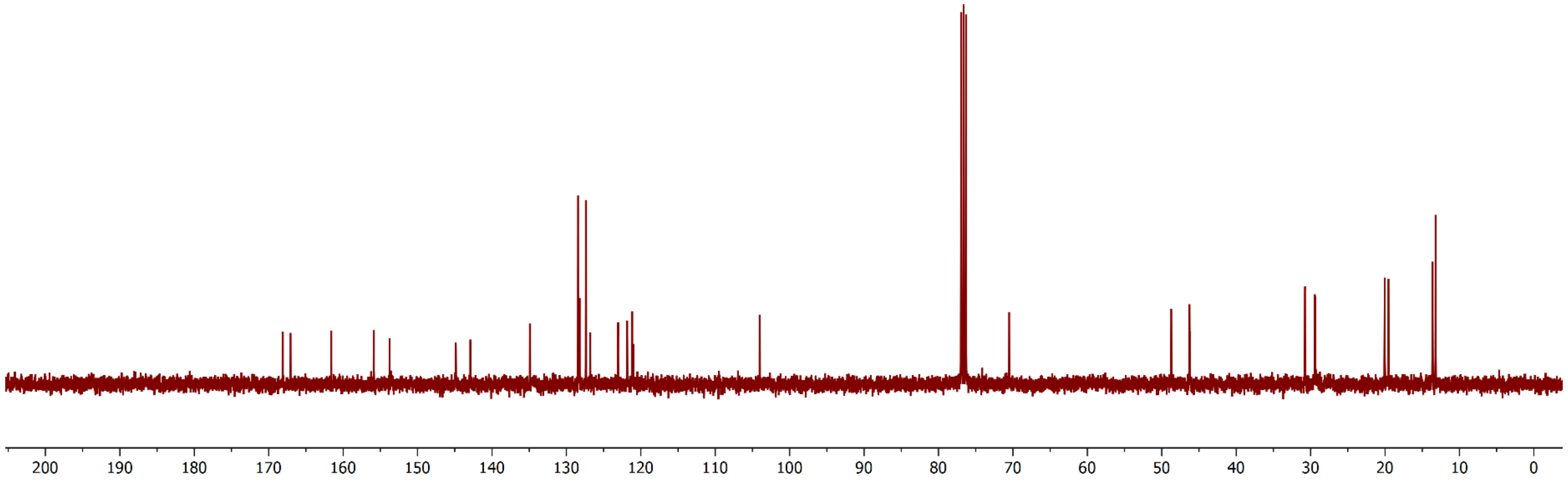

Figure S85. $^{13}C\{^{1}H\}$ NMR spectrum of **22a** (101 MHz, $CDCl_3$)

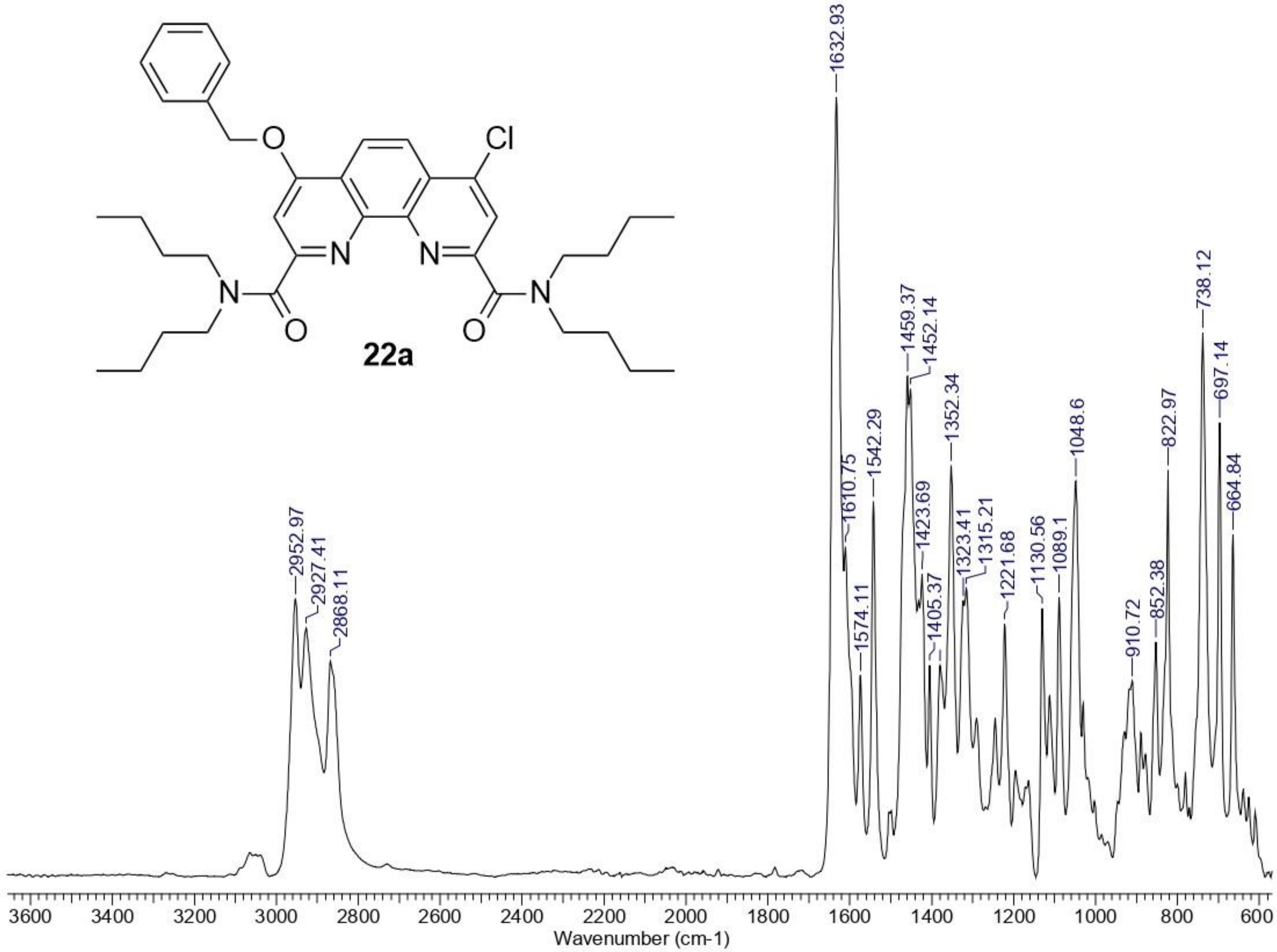

Figure S86. Solid-state IR spectrum of **22a**.

4-(Allyloxy)-*N*[2],*N*[2],*N*[9],*N*[9]-tetrabutyl-7-chloro-1,10-phenanthroline-2,9-dicarboxamide (23a).

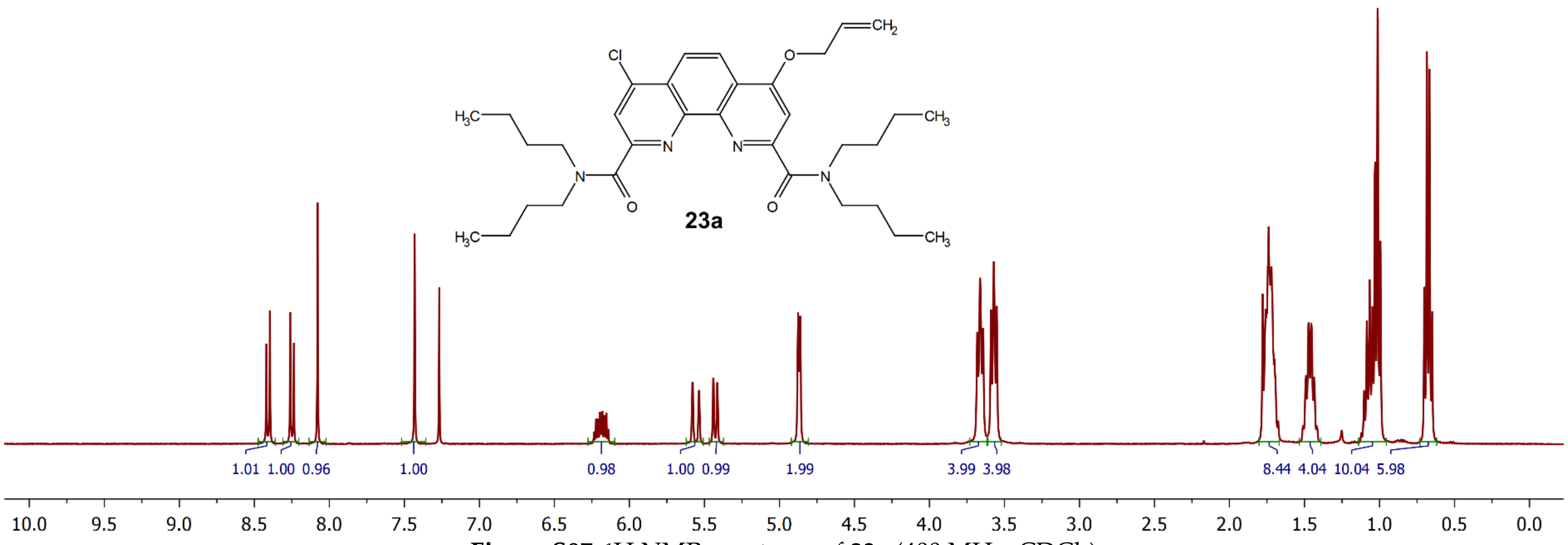

Figure S87. ^{1}H NMR spectrum of **23a** (400 MHz, $CDCl_3$)

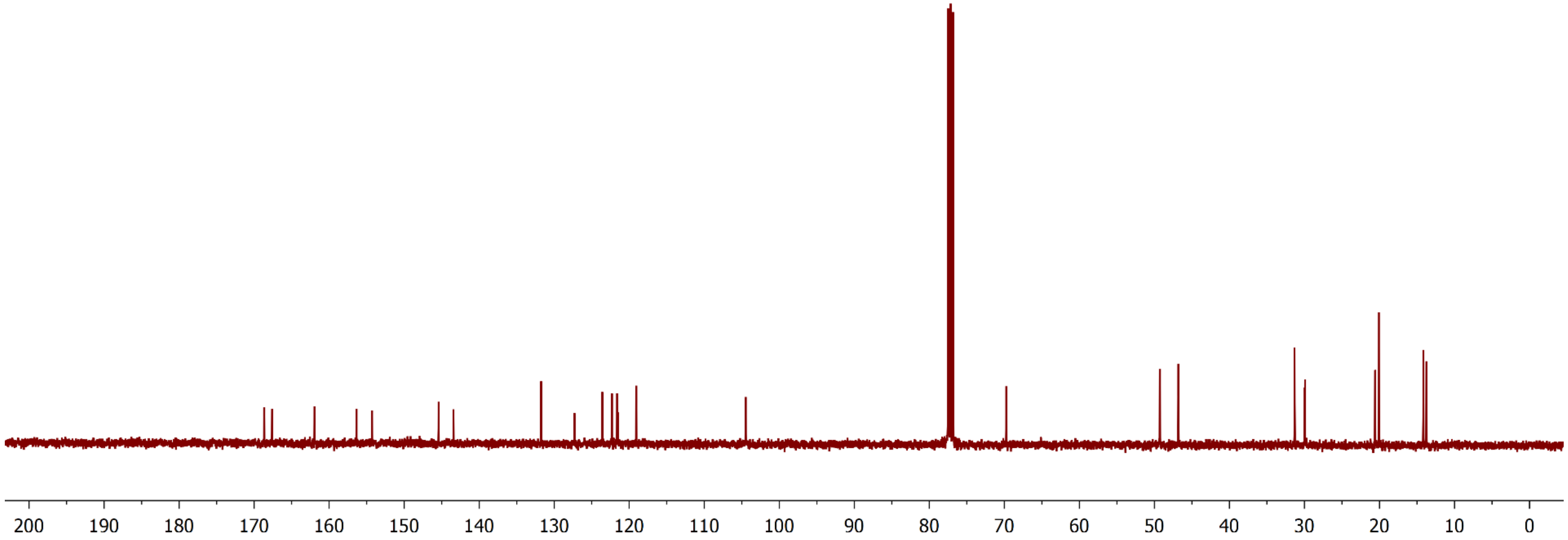

Figure S88. $^{13}C\{^{1}H\}$ NMR spectrum of **23a** (101 MHz, $CDCl_3$)

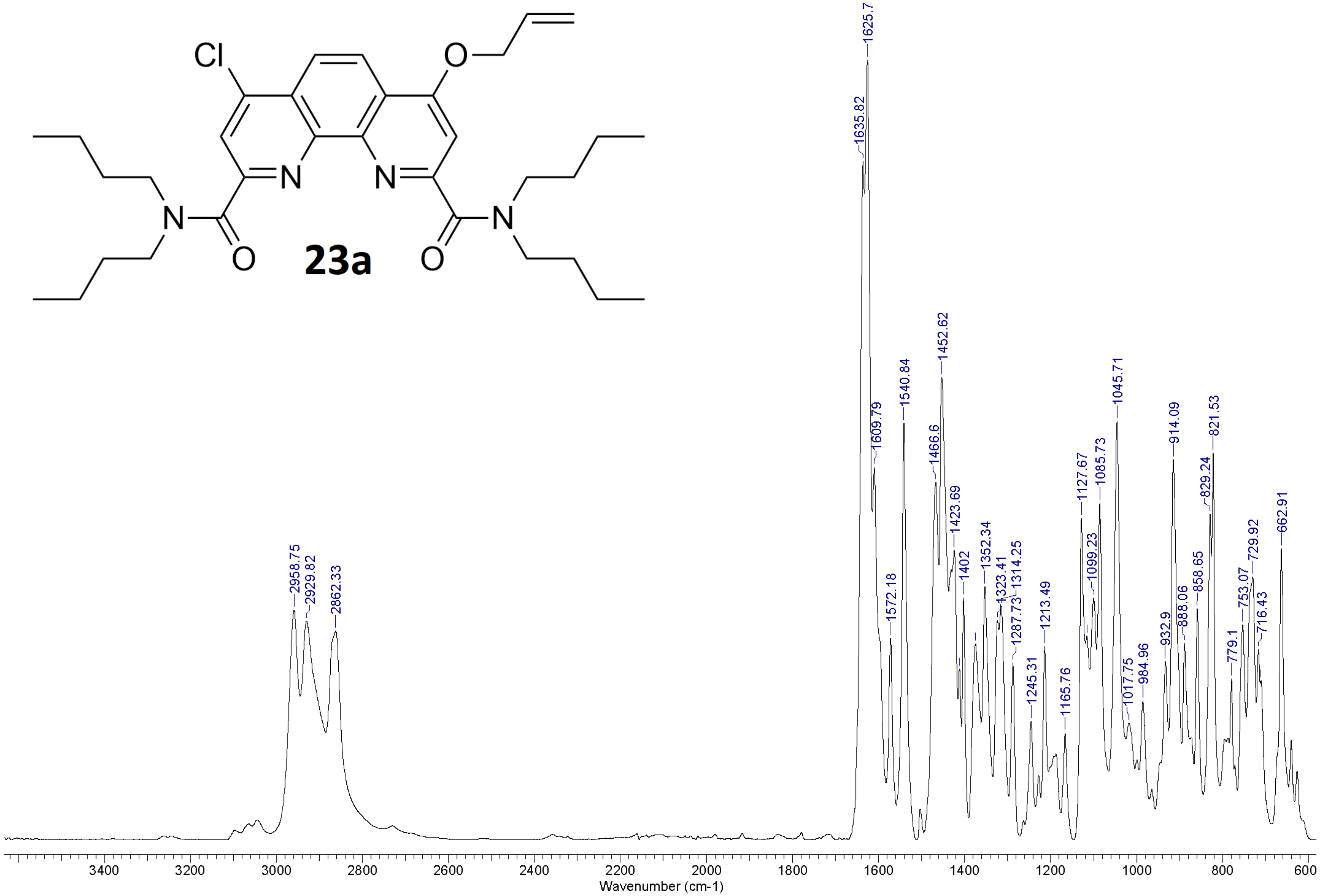

Figure S89. Solid-state IR spectrum of **23a**

N^2,N^2,N^9,N^9-Tetrabutyl-4-fluoro-7-methoxy-1,10-phenanthroline-2,9-dicarboxamide lanthanum trinitrate (17a•La(NO_3)$_3$)

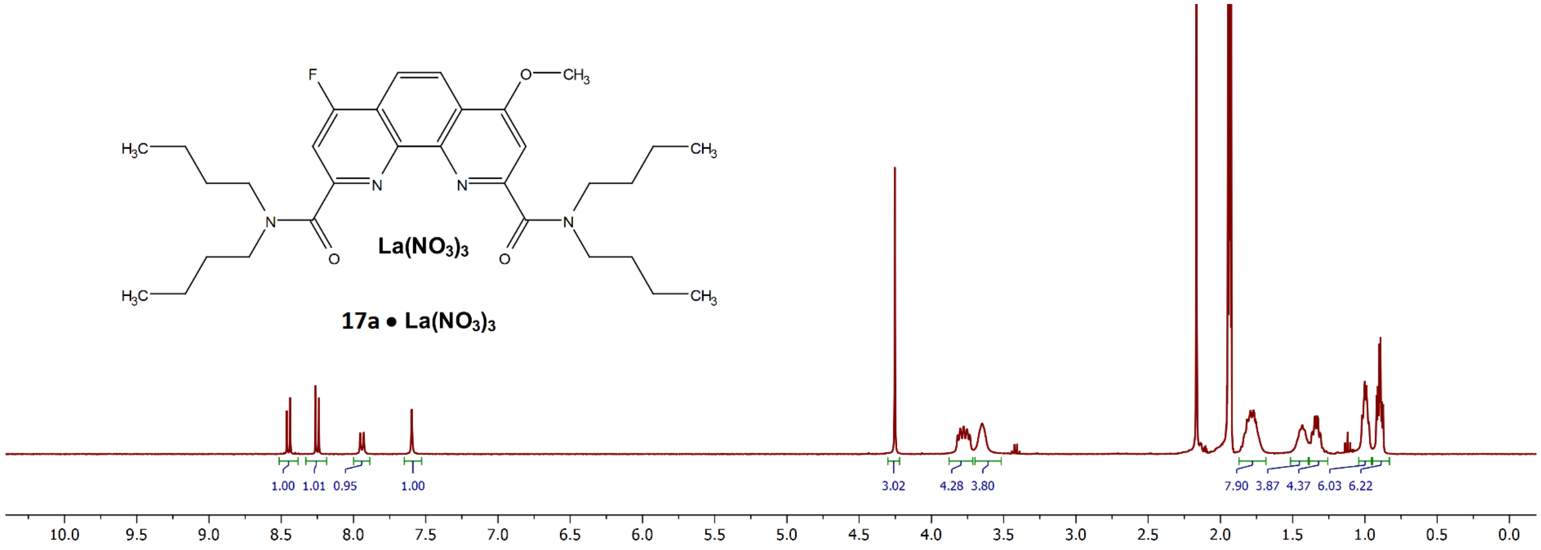

Figure S90. ^{1}H NMR spectrum of **17a•La(NO_3)$_3$** (400 MHz, CD_3CN)

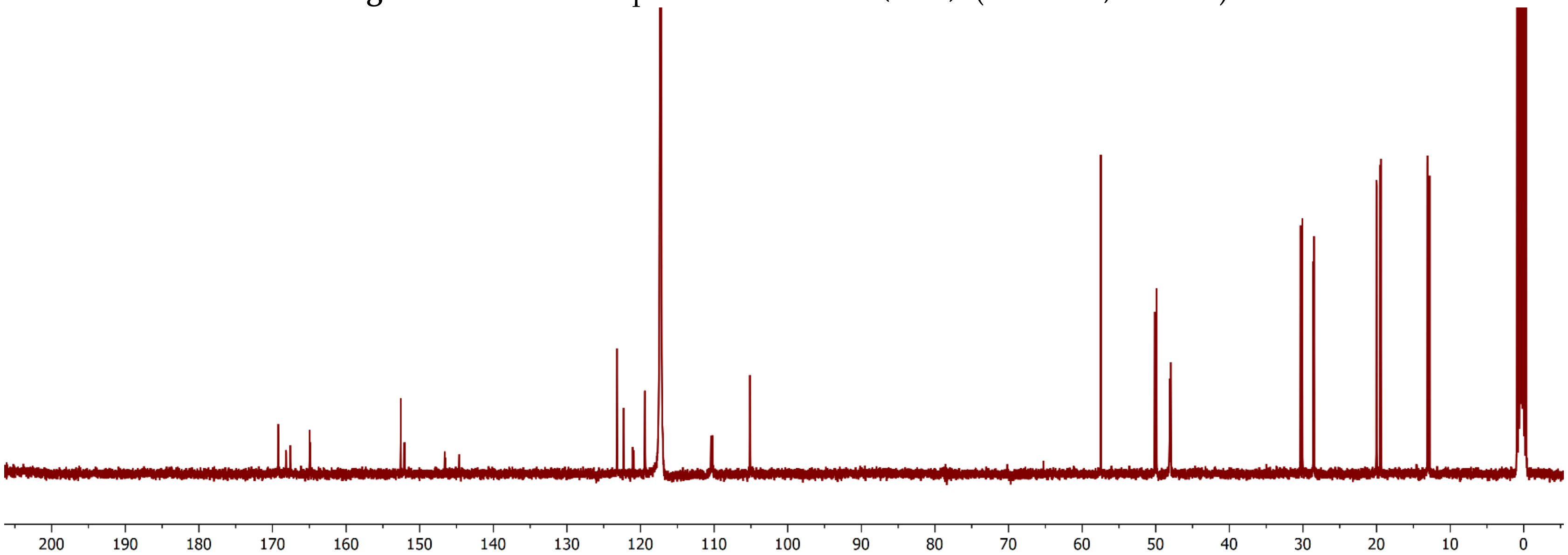

Figure S91. $^{13}C\{^1H\}$ NMR spectrum of **17a•La(NO_3)$_3$** (101 MHz, CD_3CN)

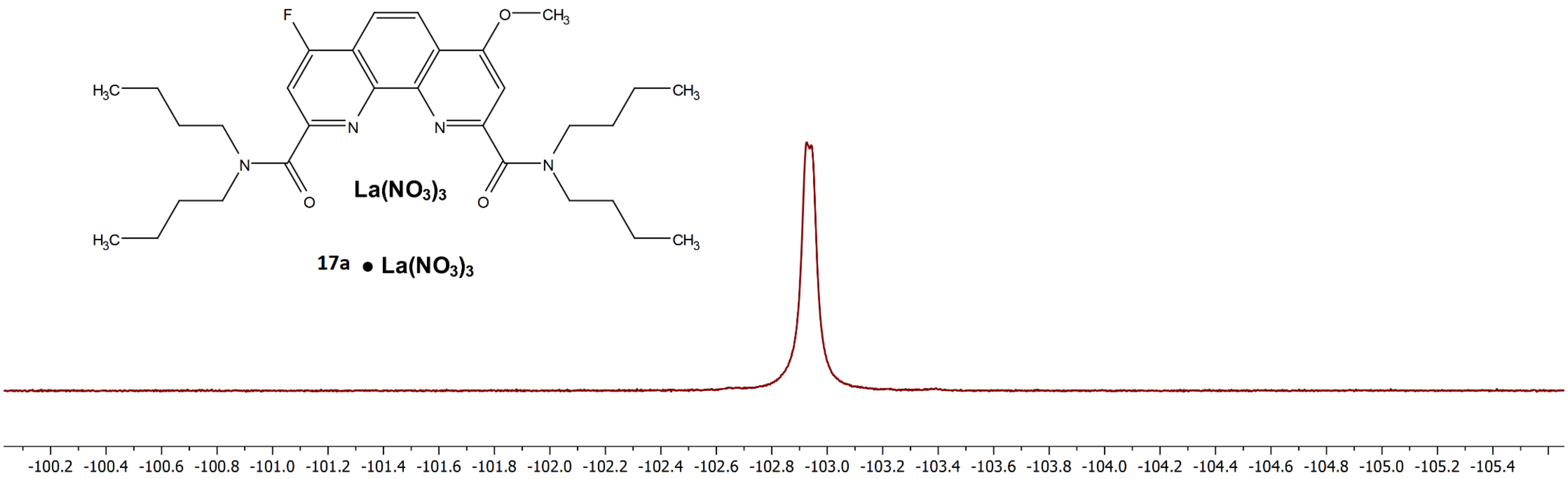

Figure S92. ^{19}F NMR spectrum of **17a•La(NO$_3$)$_3$** (376 MHz, CD_3CN)

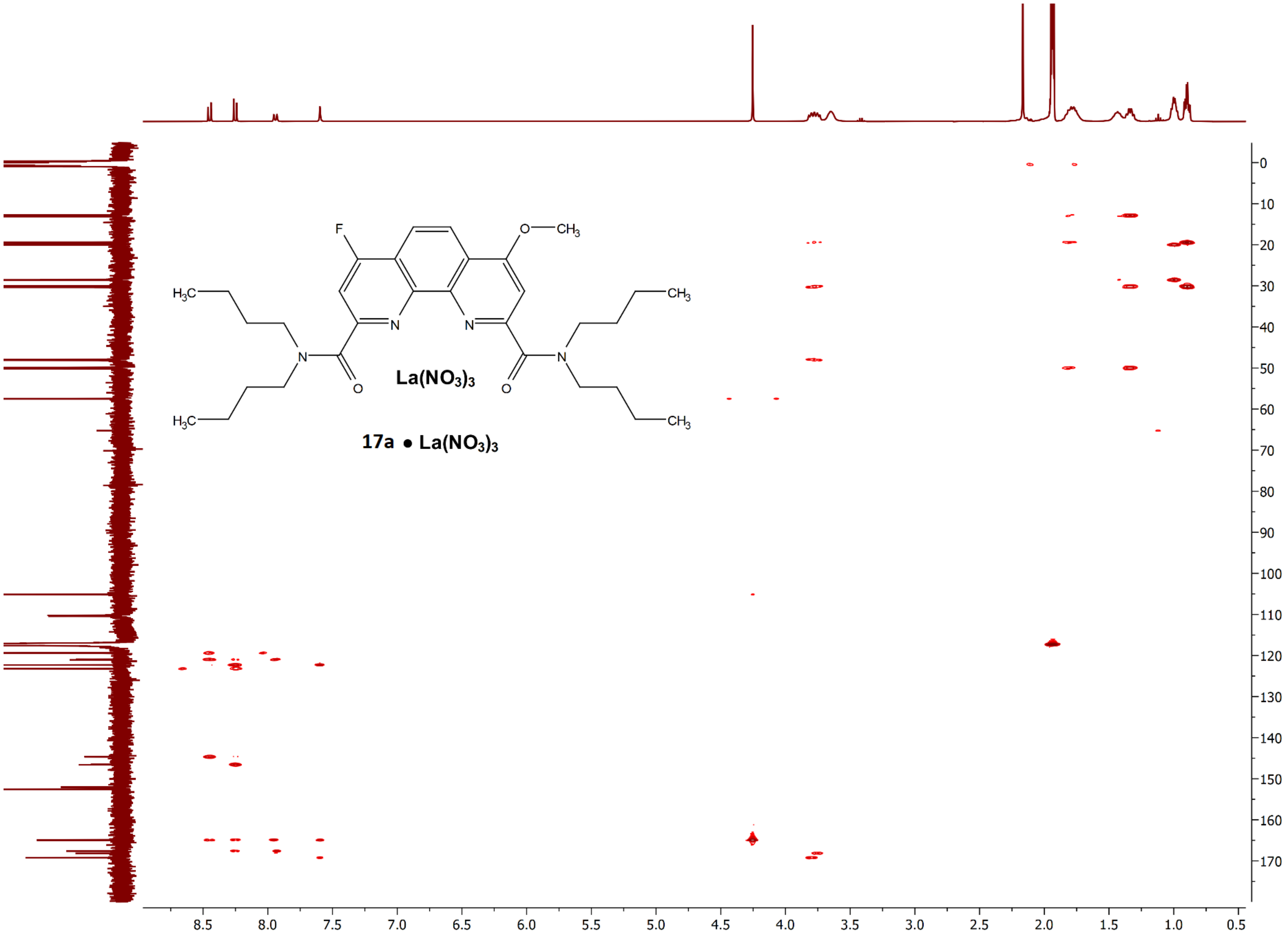

Figure S93. $^{1}H/^{13}C$ HMBC NMR spectrum of **17a•La(NO$_3$)$_3$** (CD_3CN)

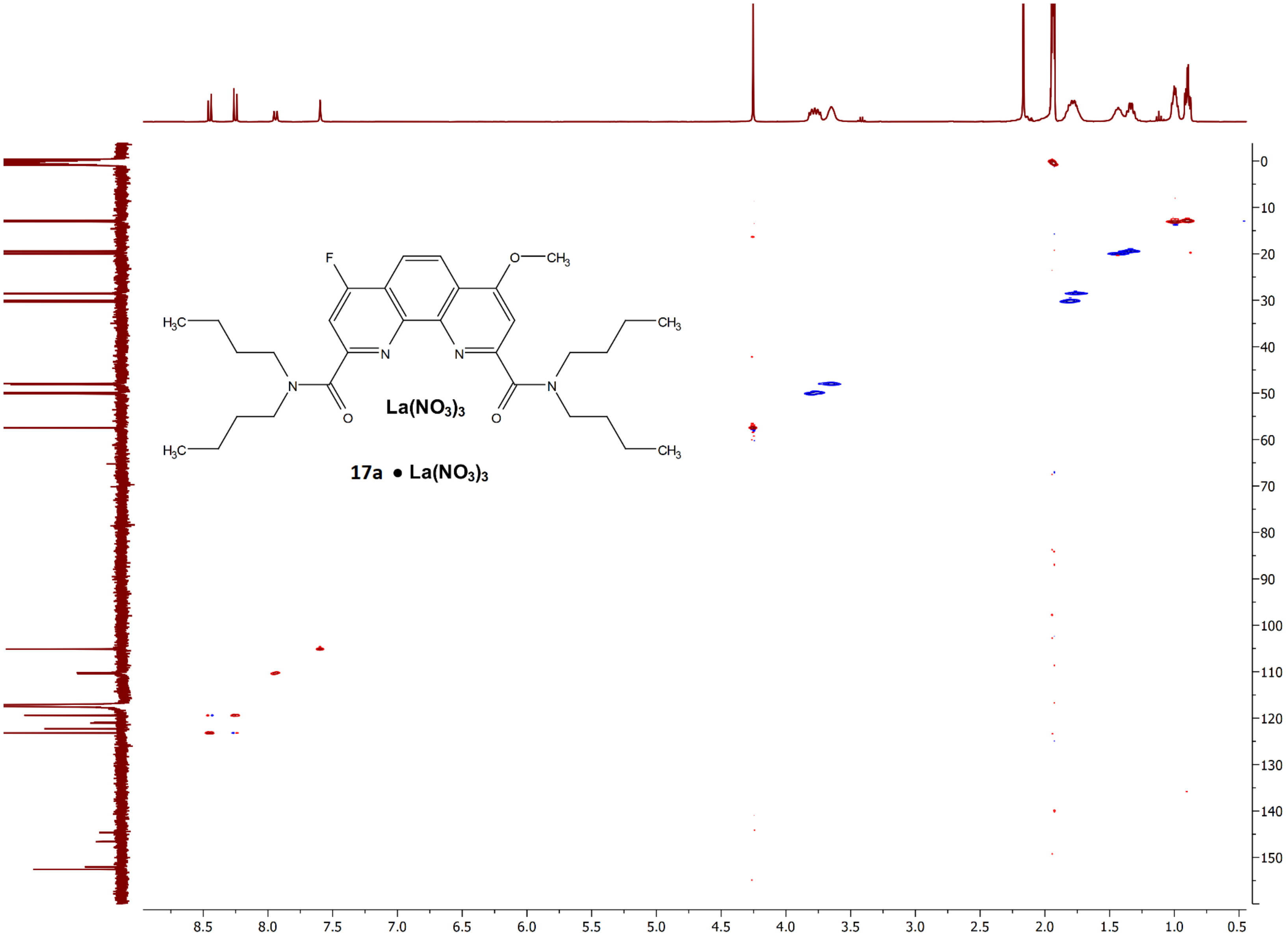

Figure S94. $^{1}H/^{13}C$ HSQC NMR spectrum of **17a●La(NO$_3$)$_3$** (CD_3CN)

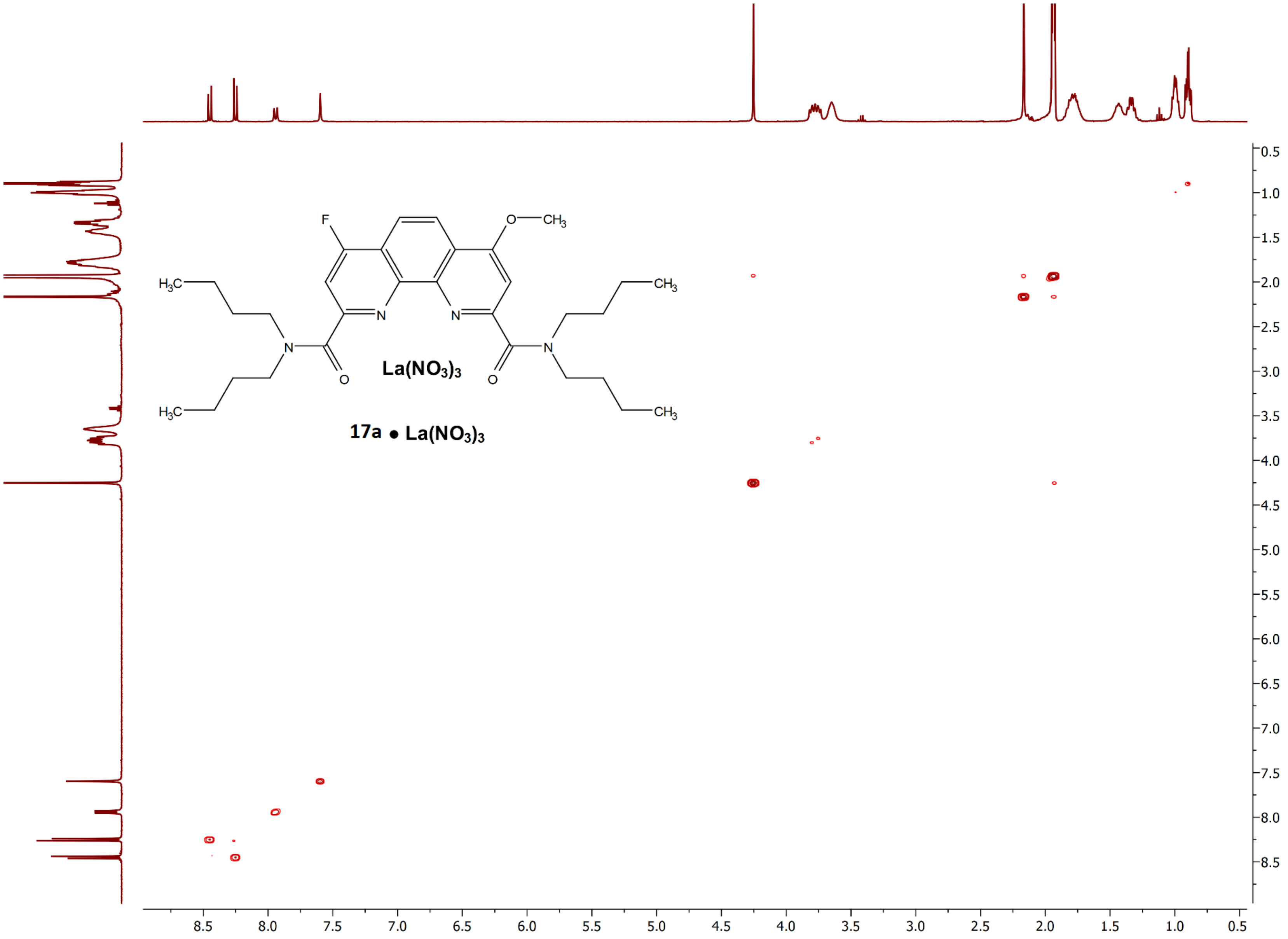

Figure S95. $^1H/^1H$ COSY NMR spectrum of **17a●La(NO$_3$)$_3$** (CD_3CN)

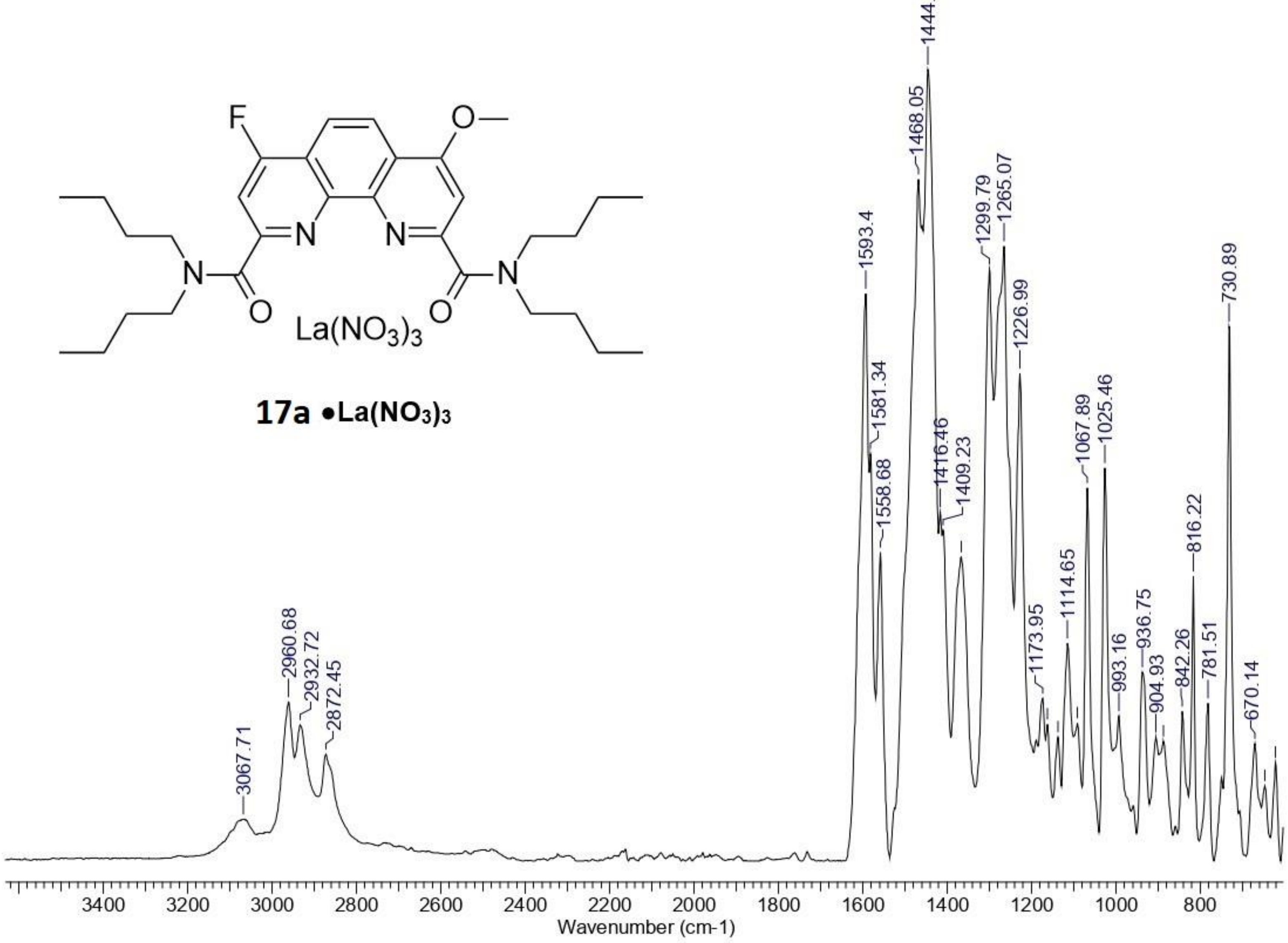

Figure S96. Solid-state IR spectrum of **17a●La(NO$_3$)$_3$**

2. XRD data

Table S1. Crystallographic data for diamides **2b** and **4b**

	**2b • ½C_6H_6**	**4b • C_6H_6 • H_2O**
Empirical formula	$C_{35}H_{34}N_5O_3$	$C_{39}H_{37}N_5O_4$
Formula weight	572.67	639.73
Temperature, K	100	100
Crystal system	orthorhombic	triclinic
Space group	*P*ccn	P-1
Z(Z′)	8(1)	2
a, Å	24.3738(3)	9.6934(2)
b, Å	19.82594(17)	13.1888(3)
c, Å	12.66527(14)	14.1144(3)
α, °	90	69.188(1)
β, °	90	83.656(1)
γ, °	90	75.946(1)
V, Å^3	6120.28(11)	1635.64(6)
D_{calc},g cm^{-3}	1.243	1.299
μ, mm^{-1}	0.646	0.086
F(000)	2424.0	676.0
θmax, °	79.885	27.000
Reflections measured	37581	18918
Independent reflections (R_{int})	6571 (0.0353)	7121 (0.0387)
Observed reflections [$I > 2\sigma(I)$]	5827	5314
Parameters	401	444
R1 with $I>2\sigma(I)$	0.0429	0.0470
wR2 (all data)	0.1180	0.1124
GOF	1.028	1.031
T_{min}/T_{max}	0.601/1.000	0.667/0.746
$\Delta\rho_{max}/\Delta\rho_{min}$, e Å^{-3}	0.295/-0.184	0.254/-0.261
CCDC	2429885	2423617

3. Computational data

Table S2. Conformational analysis of selected ligands

Compound	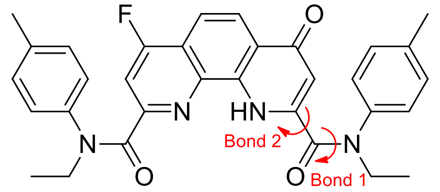	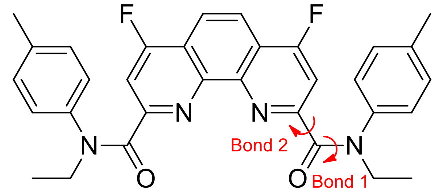
Relative energies of conformers, kcal/mol		
I	**0.0**	**0.0**
II	3.8/3.4*	0.8
III	6.7	2.0
Barrier to rotation, kcal/mol		
Bond 1	13.6/14.7*	17.0
Bond 2	11.0/9.1*	7.4

Table S3. Calculated logP and pKb values for selected ligands

Group 1. Unsubstituted, 4,7-dichlorosubstituted and 4,7- difluorosubstituted ligands

**Structure**	**logP**	**pKb**
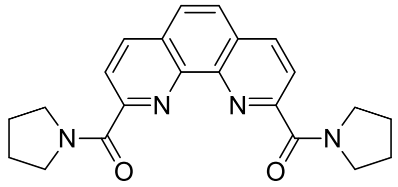	3.3	12.2
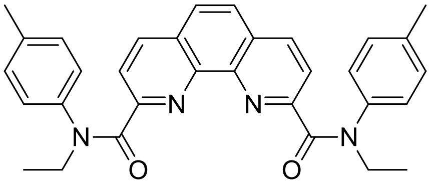	6.7	12.1
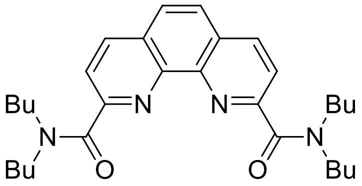	6.9	12.2
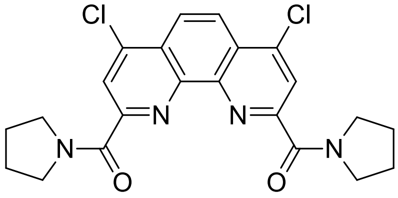	4.6	12.6

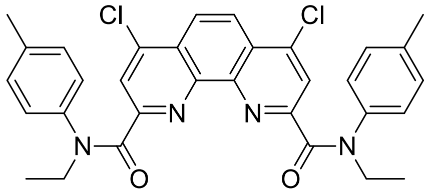	8.0	12.4
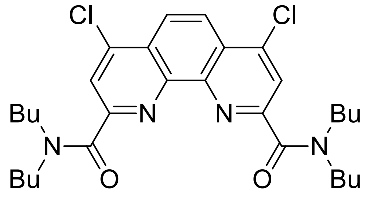	8.2	12.5
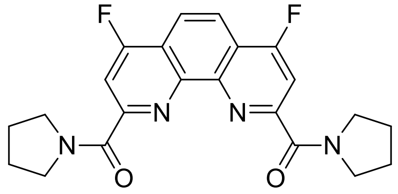	3.5	12.5
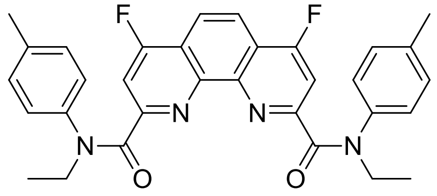	7.0	12.3
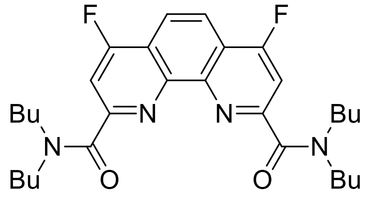	7.1	12.4

Group 2. 4,7-aminosubstituted ligands DAPhen

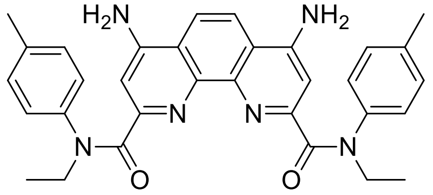	5.9	8.7
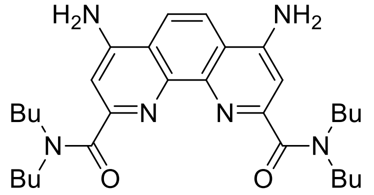	6.0	8.8
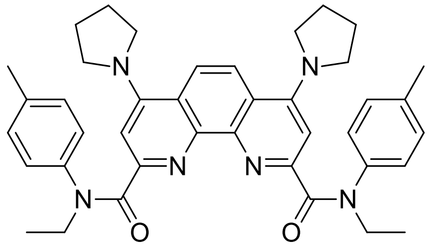	7.9	8.8

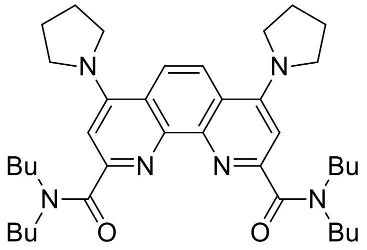	8.1	8.8
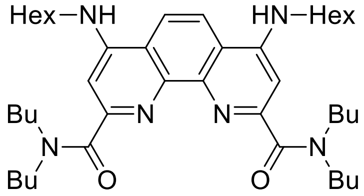	10.9	9.0
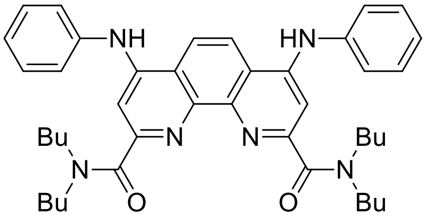	10.4	10.2
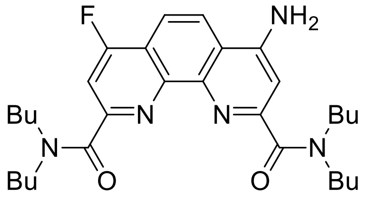	6.6	9.3
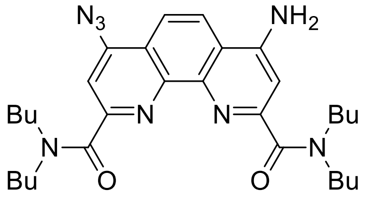	7.4	9.3

Group 3. 4,7-alkoxysubstituted ligands DAPhen (+-SPh)

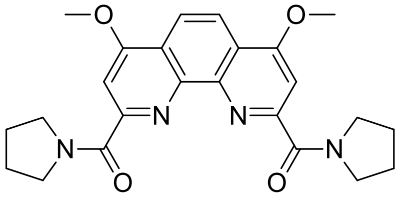	3.3	10.8
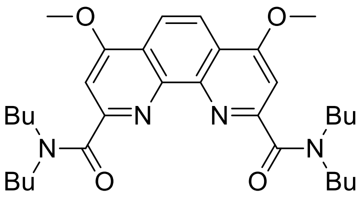	6.9	10.9

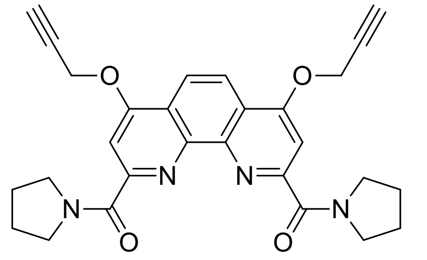	3.3	11.4
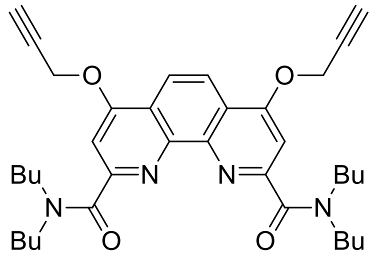	6.9	11.4
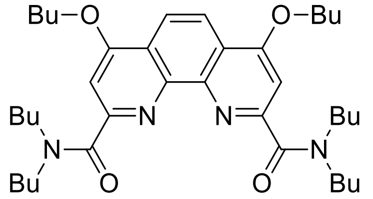	9.2	10.9
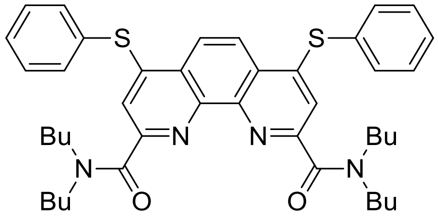	11.2	12.1
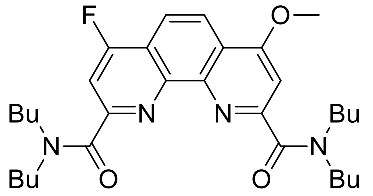	7.1	11.2
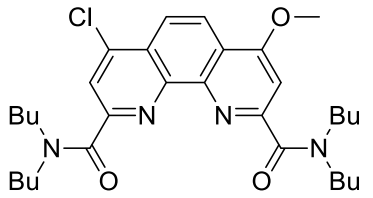	7.5	11.2
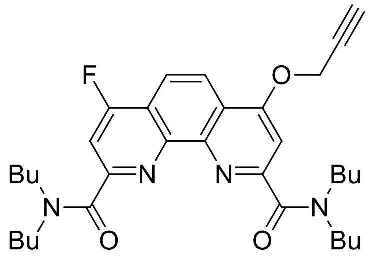	7.0	11.6

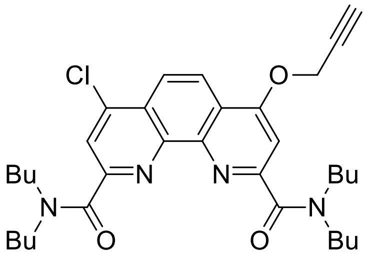	7.5	11.6
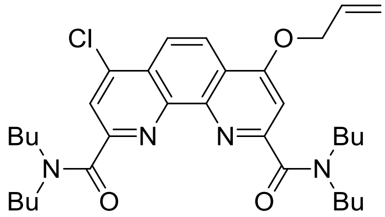	8.1	11.5
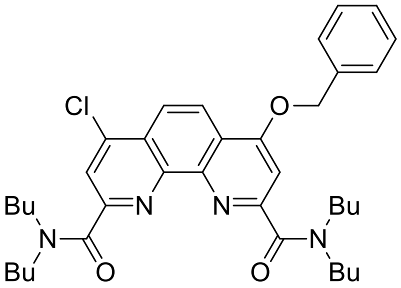	9.1	11.5
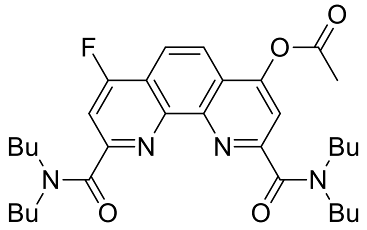	6.9	12.1
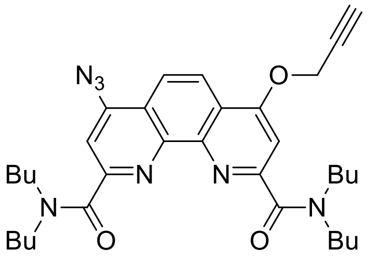	7.9	11.5

Group 4. 4,7-azidosubstituted and tryazolylsubstituted ligands DAPhen

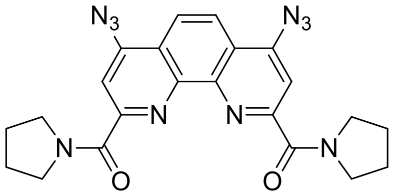	5.1	11.7
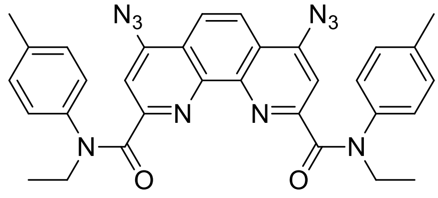	8.6	11.6

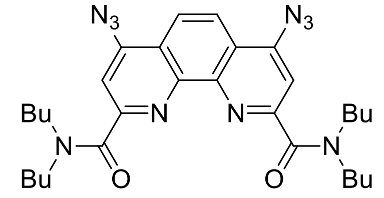	8.8	11.7
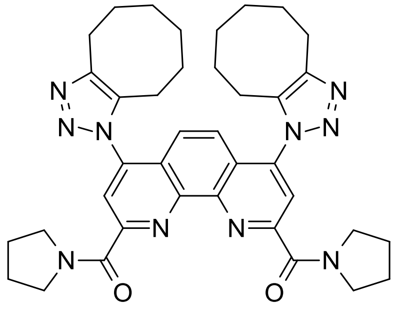	5.7	11.5
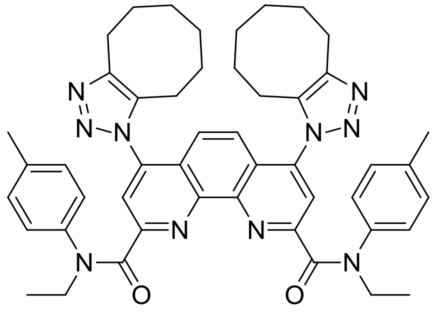	9.2	11.4

Group 5. 4-oxo-7-substituted ligands with predominant "O"-basicity

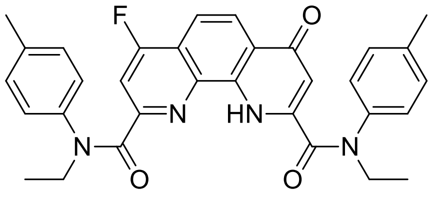	6.2	11.5
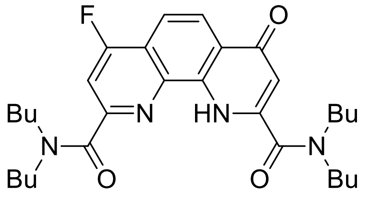	6.3	11.9
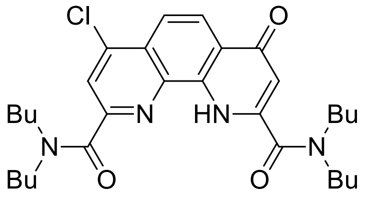	6.8	11.9
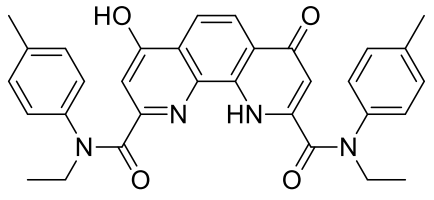	5.7	11.0

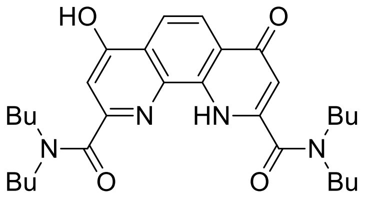	5.9	11.5
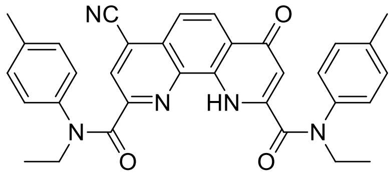	5.9	11.9
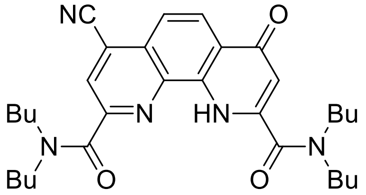	6.0	12.3
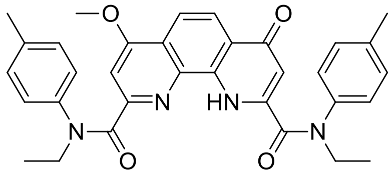	6.0	10.9
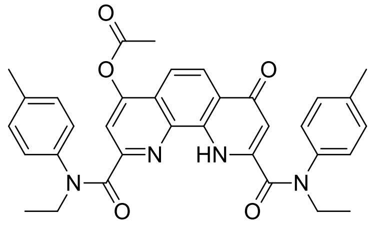	5.9	11.5

Group 6. 4-oxosubstituted ligands with aminogroup at 7 position

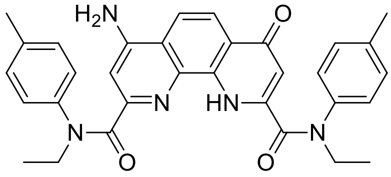	5.6	9.1
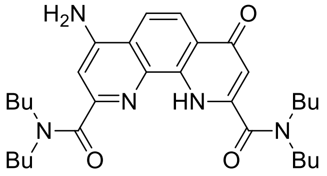	5.7	9.1
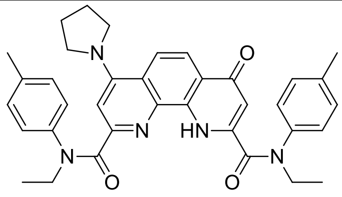	6.6	9.3

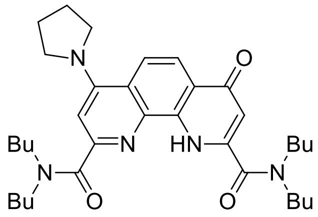	6.8	9.2

4. Cartesian coordinates, number of imaginary frequencies, and energetic parameters

1b (conformer I)

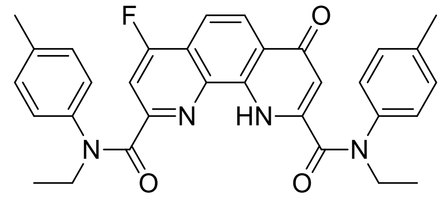

E = -1780.43206174 Eh, G = -1779.94480118 Eh, NImag = 0

F	4.532098	0.037912	5.101947
O	5.836616	-4.461799	3.327715
O	-0.983562	-5.602105	2.381249
O	-2.472221	-0.141297	4.104317
N	2.568978	-3.405533	3.960349
N	-0.143155	-3.445556	3.611917
N	4.124837	-5.861682	3.890994
N	-3.183011	-5.321269	2.898990
C	4.698401	-4.618988	3.769150
C	3.883724	-3.389806	4.116210
C	4.607492	-2.232081	4.492653
H	5.686986	-2.260818	4.567420
C	3.894316	-1.084616	4.729188
C	2.489018	-1.035201	4.580936
C	1.701475	0.133982	4.790723
H	2.191742	1.051406	5.096535
C	0.345709	0.080535	4.592495
H	-0.287877	0.950526	4.732044
C	-0.300201	-1.119840	4.187351
C	-1.762459	-1.142109	3.956688
C	-2.293404	-2.437678	3.538060
H	-3.356317	-2.491583	3.351459
C	-1.491779	-3.534494	3.389933
C	0.456262	-2.278087	3.990690
C	1.882897	-2.259918	4.179790
C	-1.875056	-4.905830	2.875450
C	3.016740	-6.161092	4.750835
C	3.100839	-5.930315	6.128475
H	4.003266	-5.489489	6.542193
C	2.032354	-6.258944	6.962423
H	2.111113	-6.063856	8.029439

C	0.867578	-6.846285	6.451271
C	0.801911	-7.080822	5.070236
H	-0.094594	-7.520593	4.641551
C	1.858507	-6.743977	4.226079
H	1.771910	-6.902667	3.155539
C	-0.271049	-7.236784	7.365004
C	4.901702	-7.006141	3.369987
H	5.410848	-6.664328	2.466824
H	4.181795	-7.780912	3.090362
C	5.919260	-7.553006	4.373885
H	6.439684	-8.417796	3.948169
H	5.429920	-7.873672	5.299313
C	-4.182245	-4.836267	3.807828
C	-3.971965	-4.872431	5.190998
H	-3.030071	-5.247778	5.580285
C	-4.959929	-4.414427	6.060844
H	-4.774908	-4.435233	7.131960
C	-6.186595	-3.934400	5.581574
C	-6.389621	-3.921212	4.194501
H	-7.327944	-3.544545	3.795241
C	-5.403561	-4.363663	3.314276
H	-5.568760	-4.321629	2.241500
C	-7.264098	-3.467811	6.531599
H	-6.836121	-2.982535	7.414133
C	-3.491346	-6.575625	2.175677
H	-2.850200	-6.604966	1.293099
H	-4.531472	-6.505544	1.845379
C	-3.284128	-7.831838	3.023443
H	-3.575382	-8.720350	2.453189
H	-3.889688	-7.800724	3.934935
H	-2.231574	-7.933575	3.299801
H	-7.940706	-2.756595	6.048931
H	-7.871811	-4.310384	6.885707
H	-0.411497	-6.505045	8.167315
H	-1.213689	-7.323896	6.815453
H	-0.083602	-8.207185	7.842333
H	6.662701	-6.788051	4.612104
H	0.438140	-4.254715	3.409442

1b (conformer II)

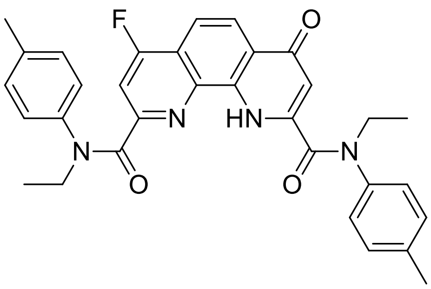

E = -1780.42600858 Eh, G = -1779.93873342 Eh, NImag = 0

F	5.680602	3.682025	-1.797991
O	5.408377	-1.364445	-1.532850
O	-1.482728	-0.290734	0.261350
O	-1.210727	5.281425	-1.501832
N	2.804724	0.845158	-0.960455
N	0.147490	1.479930	-0.938593
N	3.802684	-1.705977	0.057723
N	-3.270942	0.337095	-1.024864
C	4.498380	-0.924872	-0.830504
C	4.088852	0.524188	-1.000914
C	5.112582	1.452881	-1.309000
H	6.144513	1.132169	-1.376741
C	4.745255	2.756194	-1.528395
C	3.392550	3.166765	-1.487630
C	2.952547	4.503367	-1.715005
H	3.688542	5.271590	-1.924280
C	1.613317	4.793111	-1.665433
H	1.238972	5.798753	-1.827377
C	0.644559	3.785979	-1.399818
C	-0.795408	4.129426	-1.341198
C	-1.678077	2.993172	-1.084432
H	-2.739254	3.198689	-1.019477
C	-1.197801	1.725409	-0.910047
C	1.060722	2.471071	-1.178987
C	2.457913	2.130506	-1.206695
C	-2.000971	0.499979	-0.527353
C	3.050866	-1.177766	1.161243
C	3.682339	-0.424125	2.154467
H	4.747548	-0.224412	2.079640
C	2.945895	0.073006	3.232093
H	3.449670	0.665544	3.992101

C	1.575070	-0.181027	3.351533
C	0.955384	-0.947893	2.351453
H	-0.114354	-1.129040	2.392522
C	1.680967	-1.443448	1.272025
H	1.179281	-2.010354	0.493264
C	0.777818	0.343138	4.522917
C	4.120336	-3.147921	0.076691
H	4.327650	-3.444576	-0.953557
H	3.219510	-3.669849	0.413928
C	5.311058	-3.494566	0.973683
H	5.481010	-4.576772	0.971204
H	5.132857	-3.181734	2.007564
C	-4.110213	-0.672467	-0.424421
C	-3.758449	-2.025710	-0.434131
H	-2.815026	-2.331320	-0.871407
C	-4.615907	-2.969617	0.127733
H	-4.331080	-4.019008	0.114288
C	-5.838503	-2.595418	0.703906
C	-6.177580	-1.236467	0.700473
H	-7.119142	-0.917251	1.140602
C	-5.322886	-0.281811	0.148943
H	-5.594150	0.770016	0.168251
C	-6.747744	-3.624869	1.333293
H	-6.463784	-3.818059	2.375669
C	-3.776726	0.934850	-2.281327
H	-3.062337	1.684480	-2.614778
H	-4.718651	1.454343	-2.070032
C	-3.983939	-0.102178	-3.388840
H	-4.353545	0.398424	-4.289879
H	-4.712104	-0.864559	-3.100205
H	-3.042128	-0.601943	-3.637905
H	-7.789843	-3.291322	1.336014
H	-6.698651	-4.580820	0.802278
H	1.329232	1.111733	5.072419
H	-0.172344	0.777090	4.193690
H	0.536322	-0.459870	5.230769
H	6.216226	-3.004512	0.605475
H	0.493377	0.565204	-0.662850

1b (conformer IV)

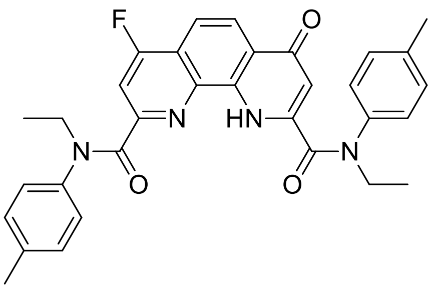

E = -1780.42627060 Eh, G = -1779.93944401 Eh, NImag = 0

F	4.389041	4.678441	0.585383
O	4.500554	0.054632	2.474117
O	-1.377382	-1.051166	-1.145896
O	-2.107927	4.722674	-2.223957
N	2.107802	1.255623	0.289133
N	-0.431602	1.330615	-0.728348
N	3.616231	-1.277214	0.834723
N	-3.543881	-0.416102	-1.427661
C	3.866952	-0.062048	1.427285
C	3.326045	1.218276	0.810492
C	4.138474	2.370495	0.954648
H	5.108698	2.302632	1.429492
C	3.644576	3.564014	0.494356
C	2.354286	3.669042	-0.074609
C	1.789348	4.883319	-0.562168
H	2.372375	5.795520	-0.504075
C	0.526142	4.875391	-1.096057
H	0.063419	5.776779	-1.484791
C	-0.242355	3.681475	-1.167485
C	-1.597450	3.695225	-1.765377
C	-2.277488	2.400654	-1.770904
H	-3.267273	2.373008	-2.202984
C	-1.695243	1.277615	-1.254628
C	0.294622	2.485869	-0.683212
C	1.623130	2.449686	-0.133349
C	-2.211649	-0.147142	-1.256155
C	4.059603	-2.468410	1.515545
C	5.404956	-2.674273	1.837721
H	6.134375	-1.905133	1.611253
C	5.798547	-3.856394	2.462055
H	6.848003	-4.002140	2.707175

C	4.871959	-4.860625	2.776053
C	3.529235	-4.641334	2.443278
H	2.787829	-5.402047	2.675911
C	3.123071	-3.457980	1.826671
H	2.074990	-3.296989	1.590254
C	5.304745	-6.125911	3.479343
C	3.178578	-1.458550	-0.566567
H	2.561985	-0.608988	-0.849144
H	2.534371	-2.342063	-0.595409
C	4.353202	-1.630788	-1.533537
H	3.980866	-1.805528	-2.548754
H	4.981502	-2.480925	-1.250298
C	-4.612171	0.508302	-1.169989
C	-4.777791	1.077455	0.096952
H	-4.076661	0.834447	0.889667
C	-5.835323	1.954235	0.335989
H	-5.947114	2.394431	1.323622
C	-6.759496	2.270394	-0.668587
C	-6.594793	1.671699	-1.926305
H	-7.300196	1.896710	-2.722270
C	-5.535021	0.803221	-2.180182
H	-5.410166	0.363040	-3.165391
C	-7.883424	3.246560	-0.414337
H	-8.168072	3.263549	0.641961
C	-3.915623	-1.842824	-1.568888
H	-3.121577	-2.328974	-2.138232
H	-4.835713	-1.874180	-2.159029
C	-4.113888	-2.548108	-0.225801
H	-4.418360	-3.587120	-0.390987
H	-4.891693	-2.058005	0.368462
H	-3.180884	-2.550222	0.343338
H	-7.585839	4.266344	-0.689017
H	-8.772012	2.997649	-1.002662
H	5.304540	-5.993240	4.568889
H	4.633400	-6.960137	3.253421
H	6.318935	-6.419161	3.189880
H	4.981113	-0.733385	-1.551679
H	0.000563	0.458085	-0.438347

1b (conformer III)

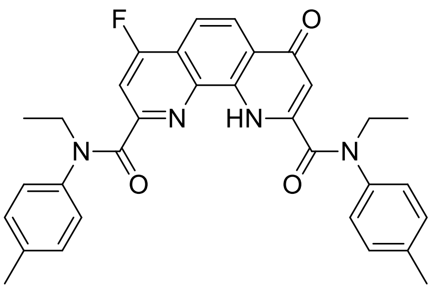

E = -1780.42040578 Eh, G = -1779.93415052 Eh, NImag = 0

F	5.695098	1.792879	-3.671271
O	3.890514	3.292526	0.743916
O	-1.361395	-1.295070	-0.447904
O	0.101915	-1.792718	-6.124573
N	2.432880	0.927031	-1.300887
N	0.256099	-0.381609	-2.309573
N	2.862961	1.441156	1.618752
N	-3.174068	-1.406431	-1.830776
C	3.429786	2.162770	0.595119
C	3.472111	1.589671	-0.812770
C	4.605127	1.929743	-1.593199
H	5.409639	2.514826	-1.166603
C	4.631683	1.514320	-2.899790
C	3.556475	0.800461	-3.478234
C	3.526835	0.349929	-4.830106
H	4.376148	0.555132	-5.471963
C	2.431941	-0.334662	-5.292013
H	2.374520	-0.701400	-6.311729
C	1.314169	-0.598828	-4.453865
C	0.151723	-1.358841	-4.969317
C	-0.925466	-1.554471	-3.999970
H	-1.777186	-2.132188	-4.332194
C	-0.855814	-1.059928	-2.727685
C	1.325334	-0.153405	-3.129937
C	2.462958	0.550218	-2.603221
C	-1.831781	-1.259607	-1.585084
C	2.723292	2.077921	2.904795
C	3.825502	2.590204	3.597007
H	4.809474	2.548450	3.144529
C	3.651938	3.165630	4.854292
H	4.518083	3.560197	5.380408

C	2.387460	3.241836	5.455191
C	1.294569	2.720652	4.751246
H	0.301484	2.766689	5.191823
C	1.455389	2.151061	3.488248
H	0.594146	1.766548	2.949196
C	2.206884	3.892711	6.806789
C	2.626399	-0.018449	1.590704
H	2.457097	-0.321197	0.560621
H	1.696987	-0.213474	2.133892
C	3.777767	-0.813418	2.213146
H	3.539552	-1.882496	2.208240
H	3.957941	-0.509505	3.248753
C	-4.004384	-1.743242	-0.698278
C	-4.077958	-3.065749	-0.255859
H	-3.487188	-3.828284	-0.754079
C	-4.904144	-3.397121	0.817112
H	-4.954257	-4.430699	1.150761
C	-5.675236	-2.424017	1.467501
C	-5.592469	-1.102468	1.007915
H	-6.179617	-0.328275	1.495843
C	-4.763544	-0.759932	-0.059566
H	-4.700079	0.272354	-0.392145
C	-6.548108	-2.783787	2.647065
H	-6.911558	-3.813626	2.577341
C	-3.886023	-1.001185	-3.059739
H	-3.180625	-0.493410	-3.716884
H	-4.641771	-0.261134	-2.771165
C	-4.566694	-2.167967	-3.777381
H	-5.106134	-1.800354	-4.656223
H	-5.285938	-2.664537	-3.119538
H	-3.840502	-2.915888	-4.110447
H	-5.992195	-2.697995	3.589414
H	-7.416168	-2.121523	2.720763
H	2.075656	4.978050	6.709333
H	1.324919	3.503381	7.324589
H	3.077477	3.728967	7.449929
H	4.704940	-0.668551	1.648183
H	0.325429	-0.127869	-1.328448

TS(amide) – 1

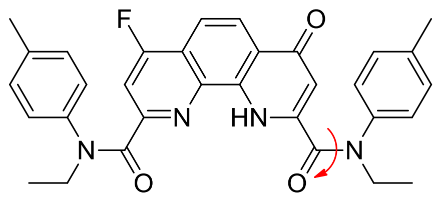

E = -1780.41113128 Eh, G = -1779.92319027 Eh, NImag = 1

F	5.309392	4.500626	-0.358787
O	6.054110	-0.395190	-1.066033
O	-1.073988	-0.967285	-1.408088
O	-1.687601	4.939994	-1.336017
N	2.939014	1.146758	-0.749192
N	0.245502	1.349692	-1.140851
N	4.201469	-1.431277	-0.227644
N	-3.173017	0.034025	-1.632332
C	4.910196	-0.321601	-0.617320
C	4.246782	1.040522	-0.569931
C	5.106460	2.159516	-0.445516
H	6.175042	2.019958	-0.343399
C	4.539439	3.407672	-0.485586
C	3.149122	3.588861	-0.669367
C	2.507138	4.859976	-0.731777
H	3.105612	5.757835	-0.626239
C	1.151528	4.925277	-0.926159
H	0.626690	5.873542	-0.979926
C	0.362837	3.750004	-1.067650
C	-1.101217	3.855901	-1.272292
C	-1.790104	2.570217	-1.389558
H	-2.863639	2.566983	-1.526955
C	-1.110753	1.385536	-1.324410
C	0.978969	2.495956	-1.008006
C	2.399043	2.386776	-0.803729
C	-1.753312	0.043785	-1.441242
C	3.074116	-1.404560	0.659296
C	3.189863	-0.868817	1.946616
H	4.135564	-0.438919	2.264120
C	2.097523	-0.885120	2.814131
H	2.202446	-0.457102	3.808359
C	0.875407	-1.454577	2.432759

C	0.776200	-1.995841	1.142374
H	-0.165363	-2.422407	0.808040
C	1.858261	-1.972638	0.264016
H	1.753631	-2.373195	-0.739866
C	-0.291962	-1.509545	3.390917
C	4.840175	-2.743440	-0.465106
H	5.385768	-2.668957	-1.407635
H	4.035305	-3.474259	-0.586852
C	5.786402	-3.167125	0.660302
H	6.205183	-4.156282	0.445900
H	5.261995	-3.221912	1.619749
C	-3.988392	-0.339350	-0.539435
C	-3.497043	-0.251482	0.778491
H	-2.474545	0.064276	0.961995
C	-4.312597	-0.551309	1.864292
H	-3.899052	-0.463591	2.866812
C	-5.646749	-0.951151	1.700111
C	-6.127000	-1.030074	0.390345
H	-7.157442	-1.331862	0.216478
C	-5.324094	-0.735991	-0.713425
H	-5.753355	-0.816236	-1.705133
C	-6.514754	-1.294058	2.888170
H	-6.438832	-0.534527	3.674466
C	-3.570319	-0.262836	-3.021779
H	-2.905365	0.321375	-3.668253
H	-4.572111	0.152227	-3.173788
C	-3.520110	-1.742962	-3.418337
H	-3.832462	-1.860094	-4.461961
H	-4.183594	-2.345633	-2.792183
H	-2.506350	-2.137501	-3.312464
H	-7.567732	-1.373294	2.601714
H	-6.224034	-2.252218	3.337695
H	-0.325982	-0.623867	4.033632
H	-1.244305	-1.581904	2.856600
H	-0.222439	-2.384784	4.049621
H	6.612466	-2.456600	0.747630
H	0.720963	0.454078	-1.070410

TS(amide) – 2

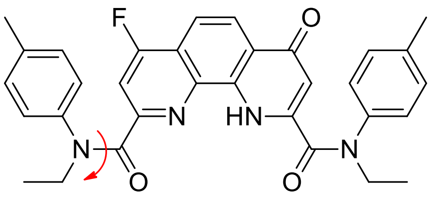

E = -1780.40921930 Eh, G = -1779.92137758 Eh, NImag = 1

F	4.746664	4.895784	0.778053
O	6.126440	0.051800	0.398289
O	-0.430789	-1.069867	-1.774289
O	-2.120172	4.541802	-0.951565
N	2.889699	1.336497	-0.165934
N	0.234287	1.229578	-0.758239
N	4.224864	-1.104205	-0.274717
N	-2.678301	-0.794749	-1.549129
C	4.948506	0.074224	0.108447
C	4.180722	1.374532	0.131817
C	4.866845	2.567985	0.465611
H	5.923142	2.545332	0.702717
C	4.144735	3.735130	0.473275
C	2.764292	3.761399	0.163096
C	1.965351	4.940304	0.140109
H	2.423509	5.892924	0.380923
C	0.636618	4.851532	-0.193960
H	-0.001060	5.729009	-0.232562
C	0.026817	3.606378	-0.509376
C	-1.402925	3.538069	-0.891547
C	-1.890215	2.192612	-1.197455
H	-2.926079	2.106133	-1.492348
C	-1.081187	1.094533	-1.115497
C	0.793012	2.439609	-0.474154
C	2.193335	2.492999	-0.147880
C	-1.387562	-0.343309	-1.485427
C	3.425741	-1.712487	0.716892
C	3.850831	-1.697074	2.060871
H	4.799569	-1.237574	2.318827
C	3.079639	-2.285830	3.057104
H	3.436842	-2.250821	4.084344
C	1.870623	-2.935712	2.768332
C	1.464468	-2.957431	1.430789

H	0.532188	-3.451656	1.164323
C	2.213529	-2.357441	0.416343
H	1.832076	-2.385894	-0.597594
C	1.062077	-3.605748	3.855003
C	3.877899	-1.201258	-1.699675
H	4.423925	-0.406790	-2.219565
H	2.811948	-0.992464	-1.861331
C	4.271602	-2.554627	-2.297635
H	3.996291	-2.593134	-3.357384
H	3.765296	-3.377264	-1.784954
C	-3.796388	-0.190496	-0.880416
C	-3.801551	-0.035479	0.509754
H	-2.940396	-0.356168	1.088283
C	-4.906077	0.531065	1.144683
H	-4.893328	0.651742	2.224949
C	-6.033528	0.937849	0.418709
C	-6.023266	0.752124	-0.971470
H	-6.886927	1.054944	-1.558095
C	-4.920326	0.198176	-1.618419
H	-4.920407	0.078867	-2.698135
C	-7.213471	1.581016	1.107748
H	-7.309455	1.237870	2.142288
C	-2.874166	-2.166052	-2.073782
H	-2.136307	-2.318259	-2.863257
H	-3.872037	-2.195629	-2.520044
C	-2.733534	-3.245632	-0.999085
H	-2.920790	-4.232857	-1.434587
H	-3.450501	-3.092164	-0.186268
H	-1.721846	-3.239666	-0.585233
H	-7.103943	2.672472	1.134419
H	-8.150701	1.360812	0.587559
H	0.969660	-2.967240	4.740975
H	0.051662	-3.845837	3.509419
H	1.526073	-4.544667	4.184024
H	5.350131	-2.713248	-2.208493
H	0.826327	0.403626	-0.778473

TS(CO) – 1

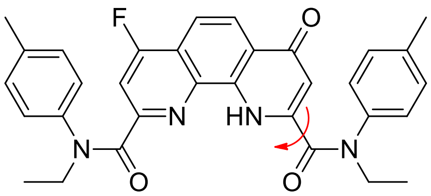

E = -1780.41675447 Eh, G = -1779.92726050 Eh, NImag = 1

F	3.408836	3.598219	1.727859
O	0.673872	1.250240	-2.682821
O	-5.523849	0.247091	2.474708
O	-2.575057	2.872192	5.335558
N	0.269799	1.355800	0.252713
N	-2.072428	1.063718	1.703009
N	2.572390	0.110600	-2.109974
N	-4.371342	-0.789126	0.838419
C	1.480832	0.907788	-1.824871
C	1.403009	1.483130	-0.421926
C	2.510071	2.235091	0.040193
H	3.420851	2.317725	-0.539735
C	2.390784	2.861926	1.252630
C	1.218591	2.756732	2.030795
C	1.066471	3.389095	3.296647
H	1.883092	3.982540	3.691767
C	-0.107418	3.234230	3.982121
H	-0.278089	3.696237	4.949045
C	-1.173740	2.456431	3.457364
C	-2.427066	2.333798	4.233132
C	-3.454791	1.540588	3.582798
H	-4.408824	1.424181	4.077668
C	-3.272002	0.932138	2.372086
C	-1.045130	1.821708	2.218409
C	0.174800	1.965826	1.461840
C	-4.462948	0.123132	1.866301
C	3.289146	-0.611447	-1.105259
C	4.690183	-0.632947	-1.111364
H	5.235573	-0.071887	-1.864533

C	5.385752	-1.365507	-0.151245
H	6.472801	-1.371972	-0.175006
C	4.713688	-2.080608	0.849847
C	3.312608	-2.054585	0.841065
H	2.762719	-2.614174	1.593944
C	2.603988	-1.345153	-0.127224
H	1.518424	-1.363524	-0.132545
C	5.473198	-2.836520	1.914483
C	2.861651	-0.160041	-3.535306
H	1.922471	-0.424514	-4.026971
H	3.514996	-1.034632	-3.567568
C	3.501184	1.029653	-4.254116
H	3.679099	0.778412	-5.305225
H	4.460688	1.305154	-3.804151
C	-3.338957	-0.931760	-0.147166
C	-3.207619	-0.006917	-1.192369
H	-3.816523	0.892654	-1.190059
C	-2.312892	-0.250750	-2.234002
H	-2.204452	0.482228	-3.027419
C	-1.562033	-1.433023	-2.281610
C	-1.679470	-2.335603	-1.214600
H	-1.090915	-3.250101	-1.219616
C	-2.551207	-2.090242	-0.155409
H	-2.646902	-2.802467	0.659208
C	-0.687515	-1.738143	-3.472575
H	-1.257935	-2.264017	-4.249044
C	-5.597973	-1.585145	0.579748
H	-6.043994	-1.816756	1.548399
H	-5.271997	-2.517532	0.110560
C	-6.613018	-0.861512	-0.306693
H	-7.483809	-1.504112	-0.476384
H	-6.183840	-0.610795	-1.282307
H	-6.953030	0.055348	0.180452

H	-0.297080	-0.815157	-3.906888
H	0.155923	-2.380182	-3.199089
H	6.451141	-3.167023	1.551518
H	5.648142	-2.208022	2.797022
H	4.919709	-3.718884	2.250387
H	2.837248	1.897155	-4.221014
H	-1.909885	0.644020	0.794385

TS(CO) – 2

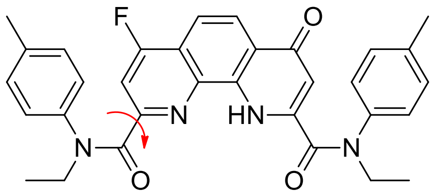

E = -1780.41869216 Eh, G = -1779.93026776 Eh, NImag = 1

F	3.125396	2.375423	3.198402
O	2.254433	-2.000635	-0.806629
O	-1.567044	-0.211894	-3.209794
O	-2.879451	4.502121	0.079318
N	1.302950	0.204877	0.228055
N	-1.001775	1.035381	-0.956408
N	3.900947	-2.330335	0.702909
N	-3.778966	0.336989	-3.190468
C	2.871274	-1.577336	0.163013
C	2.426886	-0.241612	0.777854
C	3.079672	0.486995	1.806301
H	3.996277	0.162255	2.270855
C	2.517733	1.670222	2.226556
C	1.324276	2.178180	1.678481
C	0.692360	3.392493	2.076018
H	1.143011	3.991294	2.859687
C	-0.470137	3.777965	1.459576
H	-0.983427	4.695461	1.729330
C	-1.064309	2.996814	0.428686
C	-2.311481	3.448601	-0.229544
C	-2.808678	2.548988	-1.267593
H	-3.718849	2.839396	-1.773534
C	-2.161179	1.390845	-1.593623
C	-0.455824	1.804888	0.029584

C	0.765343	1.362954	0.653944
C	-2.494194	0.430339	-2.714561
C	4.827811	-1.960952	1.732442
C	5.986300	-1.235984	1.426263
H	6.155203	-0.910815	0.404168
C	6.902428	-0.920683	2.430188
H	7.794061	-0.351851	2.177907
C	6.696030	-1.326957	3.756132
C	5.542424	-2.070648	4.045196
H	5.363386	-2.404719	5.064306
C	4.619519	-2.387938	3.049483
H	3.727105	-2.960427	3.285235
C	7.674880	-0.954519	4.844938
C	4.140776	-3.645572	0.065022
H	3.166459	-4.054227	-0.210391
H	4.587837	-4.289382	0.828566
C	5.039403	-3.570076	-1.171100
H	5.179057	-4.571445	-1.593193
H	6.027962	-3.168776	-0.924410
C	-4.953244	0.674082	-2.437951
C	-5.193860	0.098900	-1.185487
H	-4.473487	-0.598455	-0.768437
C	-6.351694	0.420741	-0.478403
H	-6.520626	-0.029742	0.496535
C	-7.304810	1.302342	-1.004832
C	-7.062471	1.851365	-2.272390
H	-7.787214	2.535609	-2.706601
C	-5.902240	1.547926	-2.981904
H	-5.721038	1.997808	-3.953914
C	-8.542539	1.670820	-0.221528
H	-8.828580	0.874836	0.472616
C	-3.971637	-0.497534	-4.398575
H	-3.100695	-0.343824	-5.038049
H	-4.854219	-0.110837	-4.915781
C	-4.140374	-1.984791	-4.081538
H	-4.312560	-2.547032	-5.005571
H	-4.994496	-2.153629	-3.417777
H	-3.237453	-2.374718	-3.605247

H	-8.375623	2.578181	0.372600
H	-9.392309	1.867356	-0.882682
H	8.693483	-0.867057	4.454832
H	7.414547	0.011784	5.295184
H	7.681027	-1.696584	5.649009
H	4.577565	-2.938292	-1.933723
H	-0.481333	0.225048	-1.287556

EtTolDAPhenFF (I)

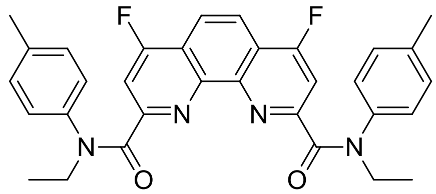

E = -1804.42083555 Eh, G = -1803.94683339 Eh, NImag = 0

O	-1.570363	-2.418989	2.635993
O	3.452342	1.910680	-3.007633
N	-2.631549	-1.843568	0.687407
N	-0.645997	0.444939	1.310638
N	3.049502	-0.066551	-1.944162
N	1.373269	1.564721	-0.205661
C	2.844300	-0.816920	-0.736770
C	-1.915830	-1.563039	1.822313
C	-5.023708	0.444574	-1.036906
H	-6.075748	0.711476	-0.971098
C	0.805460	2.303068	0.771201
C	-1.552924	-0.115521	2.095178
C	1.840385	-1.790814	-0.685802
H	1.207781	-1.950394	-1.554304
C	-0.234069	1.699693	1.595467
C	-4.497138	-0.489293	-0.145213
H	-5.132688	-0.945501	0.609033
C	-3.008966	-3.244258	0.419268
H	-4.094297	-3.281413	0.269426
H	-2.768656	-3.805778	1.323315
C	2.346823	2.102598	-0.924252
C	-0.335714	3.802769	2.924501
H	-0.778051	4.345766	3.752428

C	-1.721371	1.804217	3.481076
C	0.613568	4.379045	2.134175
H	0.946735	5.395656	2.311243
C	2.796457	3.434207	-0.765781
H	3.563634	3.839304	-1.413039
C	-3.143470	-0.837815	-0.207489
C	-2.113930	0.513216	3.229413
H	-2.825281	0.006292	3.870418
C	3.609094	-0.788687	-3.104918
H	3.302836	-0.248315	-4.002638
H	3.146887	-1.779924	-3.119898
C	1.203351	3.640915	1.058904
C	-4.216102	1.051100	-2.008684
C	-0.765162	2.459632	2.676210
C	1.628079	-2.514654	0.481838
H	0.828045	-3.249356	0.520587
C	3.001466	1.302428	-2.035350
C	-2.860454	0.696442	-2.053681
H	-2.209507	1.163712	-2.788568
C	-2.324409	-0.241516	-1.171776
H	-1.267602	-0.481546	-1.194151
C	3.423263	-1.336731	1.555430
H	4.048049	-1.152021	2.426036
C	3.656721	-0.616617	0.381440
H	4.455672	0.118133	0.337862
C	2.214221	4.174663	0.231265
C	2.400431	-2.288140	1.632228
C	-4.792401	2.044079	-2.990939
H	-4.057147	2.806365	-3.266449
H	-5.670273	2.551394	-2.579404
C	5.133929	-0.909084	-3.055148
H	5.592375	0.082820	-3.084924
H	5.495573	-1.481062	-3.916592
H	5.459018	-1.423231	-2.144910
C	2.104062	-3.041363	2.907255
H	2.260166	-4.119815	2.781298
H	1.058039	-2.897685	3.201535
C	-2.292573	-3.835579	-0.795848

H	-2.495645	-3.255570	-1.701425
H	-2.633833	-4.862228	-0.966601
H	-1.210028	-3.858465	-0.638421
H	-5.107317	1.547143	-3.917496
H	2.743909	-2.705995	3.728738
F	-2.244119	2.474313	4.523825
F	2.610881	5.442082	0.448986

EtTolDAPhenFF (II)

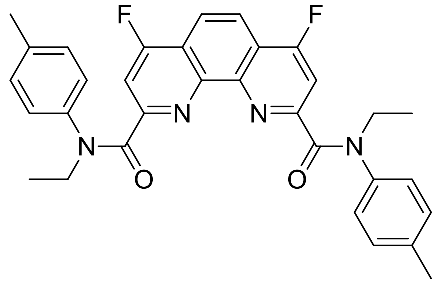

E = -1804.41904467 Eh, G = -1803.94562531 Eh, NImag = 0

O	-3.523175	-0.686662	1.531191
O	4.567528	2.331231	-1.480550
N	-2.571024	0.060472	-0.408805
N	-0.593604	1.212466	1.501556
N	3.380024	0.382923	-1.369983
N	2.028214	1.752965	0.815603
C	2.838031	-0.678734	-0.570006
C	-2.710342	0.039044	0.959621
C	-3.658320	-2.531439	-2.866302
H	-3.192240	-3.238577	-3.548347
C	1.547345	2.228992	1.983962
C	-1.840409	0.922950	1.843314
C	1.564104	-1.187266	-0.849031
H	0.970724	-0.728587	-1.634052
C	0.171588	1.925047	2.356559
C	-2.852153	-1.716389	-2.070472
H	-1.770157	-1.794303	-2.132586
C	-1.810270	1.079837	-1.171823
H	-1.566163	0.625945	-2.135897
H	-0.876077	1.279607	-0.650686
C	3.277043	2.041957	0.480721
C	0.516866	3.175422	4.480833
H	0.103706	3.516567	5.423579
C	-1.642921	2.054811	3.926183

C	1.797315	3.477684	4.124232
H	2.432691	4.070163	4.773266
C	4.137327	2.846024	1.262853
H	5.133982	3.091319	0.918345
C	-3.427099	-0.802516	-1.183466
C	-2.419094	1.310321	3.075770
H	-3.427581	1.011502	3.329988
C	3.799685	0.050825	-2.746426
H	3.798976	0.982311	-3.316009
H	3.036024	-0.612306	-3.163956
C	2.329986	3.013836	2.879196
C	-5.054353	-2.462128	-2.791606
C	-0.311200	2.398906	3.608923
C	1.053140	-2.243865	-0.103248
H	0.054557	-2.616348	-0.319556
C	3.806509	1.585043	-0.864253
C	-5.617713	-1.547582	-1.889359
H	-6.699825	-1.477840	-1.805273
C	-4.821730	-0.723909	-1.096963
H	-5.276616	-0.032921	-0.396461
C	3.063653	-2.305434	1.210029
H	3.658909	-2.736317	2.011464
C	3.590728	-1.253363	0.457123
H	4.586982	-0.874778	0.667531
C	3.645749	3.303762	2.458743
C	1.791042	-2.821026	0.943364
C	-5.930459	-3.330356	-3.664550
H	-6.784959	-3.725884	-3.105424
H	-5.372676	-4.178045	-4.073384
C	5.179689	-0.607432	-2.815556
H	5.943511	0.077236	-2.437819
H	5.425523	-0.858052	-3.853219
H	5.208074	-1.530634	-2.227756
C	-2.611154	2.365589	-1.395661
H	-3.556677	2.161408	-1.908648
H	-2.033925	3.066048	-2.008972
H	-2.836737	2.858841	-0.444246
H	-6.334510	-2.762904	-4.512668

F	-2.142901	2.473075	5.102833
F	4.419719	4.053138	3.264132
C	1.218182	-3.963736	1.748668
H	0.248145	-3.696296	2.182905
H	1.055979	-4.850353	1.123816
H	1.884815	-4.251284	2.566712

EtTolDAPhenFF (III)

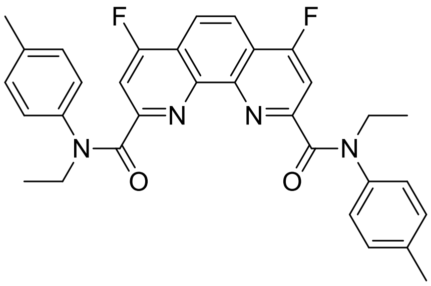

E = -1804.41641147 Eh, G = -1803.94359677 Eh, NImag = 0

O	-2.701325	1.060099	-3.525511
O	1.360021	0.345077	4.558077
N	-0.561877	0.861425	-2.736195
N	-1.874759	1.935042	-0.276780
N	0.413895	-1.118474	3.074606
N	-1.041701	1.356264	2.299591
C	1.536513	-2.009834	3.205272
C	-1.854055	1.324781	-2.674733
C	0.680191	-2.129903	-4.587017
H	1.060226	-3.121190	-4.351444
C	-1.914827	2.370531	2.104481
C	-2.303684	2.189206	-1.505431
C	1.388267	-3.247894	3.837521
H	0.430393	-3.521074	4.271050
C	-2.374765	2.656600	0.751649
C	0.288644	-1.283738	-3.549249
H	0.354340	-1.615540	-2.516738
C	0.566245	1.395306	-1.939607
H	1.282921	0.578415	-1.816833
H	0.199251	1.651795	-0.948162
C	-0.608059	1.118432	3.529920
C	-3.811938	4.468873	1.672900
H	-4.535991	5.253225	1.482370
C	-3.757159	3.916714	-0.758561
C	-3.362287	4.218515	2.935023

H	-3.714850	4.800131	3.779556
C	-0.996503	1.876029	4.659429
H	-0.575888	1.671247	5.635701
C	-0.207880	-0.008318	-3.830268
C	-3.277847	3.170586	-1.804156
H	-3.633279	3.306956	-2.817327
C	-0.736644	-1.608445	2.288585
H	-1.615512	-1.023847	2.558882
H	-0.925886	-2.640205	2.604780
C	-2.411990	3.174410	3.169271
C	0.578324	-1.729812	-5.925198
C	-3.328214	3.697951	0.568543
C	2.470248	-4.122168	3.931337
H	2.338849	-5.079407	4.430499
C	0.466883	0.071273	3.759635
C	0.066497	-0.451643	-6.191400
H	-0.033220	-0.120019	-7.222351
C	-0.319241	0.404504	-5.162040
H	-0.725498	1.384233	-5.387155
C	3.861879	-2.532156	2.790530
H	4.827186	-2.239514	2.384307
C	2.783559	-1.655939	2.682562
H	2.904859	-0.689782	2.203598
C	-1.908492	2.878847	4.455201
C	3.725253	-3.782572	3.408615
C	1.022628	-2.637501	-7.048690
H	0.371291	-2.539880	-7.923234
H	1.019612	-3.687271	-6.740091
C	-0.509226	-1.553379	0.777838
H	-0.450170	-0.514474	0.448114
H	-1.345764	-2.034527	0.258988
H	0.409746	-2.082420	0.503478
C	1.245895	2.592670	-2.609996
H	1.608704	2.336651	-3.610556
H	2.102037	2.922896	-2.011555
H	0.553101	3.436128	-2.703056
H	2.042094	-2.395704	-7.376011
F	-4.665436	4.882970	-0.980108

F	-2.341439	3.618602	5.491094
C	4.887621	-4.744557	3.490767
H	4.867063	-5.318679	4.422716
H	4.865135	-5.467156	2.664729
H	5.846039	-4.219366	3.438237

TS (amide)

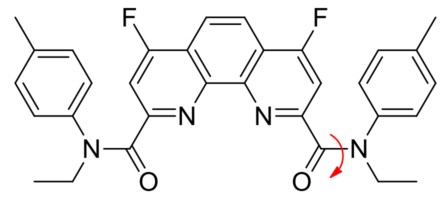

E = -1804.39333566 Eh, G = -1803.91967818 Eh, NImag = 1

O	-4.567505	0.393285	1.655933
O	2.751537	2.804258	-2.368113
N	-2.837763	-0.947249	0.826335
N	-1.206443	1.231405	1.778932
N	2.275450	0.615198	-1.909404
N	1.164831	1.926081	0.511977
C	2.399853	-0.474382	-0.986789
C	-3.379232	0.158308	1.577650
C	-4.035390	-1.544706	-2.688863
H	-4.708679	-2.209025	-3.226442
C	0.902305	2.425535	1.740329
C	-2.388890	1.008790	2.336150
C	1.388646	-1.437026	-0.893308
H	0.469823	-1.307025	-1.456736
C	-0.351829	2.072788	2.398945
C	-3.907424	-1.685811	-1.305261
H	-4.487947	-2.446783	-0.794089
C	-3.144825	-2.218258	1.508536
H	-4.197577	-2.512531	1.372176
H	-3.020784	-2.034102	2.582635
C	2.265001	2.329155	-0.108427
C	0.300992	3.519808	4.321867
H	0.047261	3.923045	5.295817
C	-1.901760	2.365823	4.220483
C	1.477207	3.838364	3.712634
H	2.190084	4.503218	4.187686
C	3.194944	3.241700	0.439903

H	4.054683	3.575455	-0.127726
C	-3.040302	-0.851536	-0.588808
C	-2.809073	1.576876	3.560498
H	-3.800921	1.391510	3.953598
C	2.301821	0.300017	-3.352385
H	1.838694	1.141387	-3.871521
H	1.675797	-0.584322	-3.501125
C	1.782847	3.320999	2.413636
C	-3.330472	-0.562731	-3.393924
C	-0.636530	2.659005	3.668305
C	1.551612	-2.537342	-0.055720
H	0.760824	-3.280579	-0.000377
C	2.471057	1.924198	-1.555342
C	-2.466036	0.267243	-2.663770
H	-1.891654	1.030962	-3.184088
C	-2.308233	0.123448	-1.287404
H	-1.602653	0.739755	-0.736810
C	3.699663	-1.718348	0.637674
H	4.605152	-1.816344	1.231799
C	3.561003	-0.627120	-0.220891
H	4.358314	0.105828	-0.304018
C	2.946952	3.695712	1.709072
C	2.705189	-2.699156	0.726743
C	-3.507744	-0.387269	-4.884376
H	-4.258695	0.381304	-5.109313
H	-3.839733	-1.314724	-5.361460
C	3.713930	0.060369	-3.891442
H	4.318518	0.964227	-3.780456
H	3.671710	-0.196814	-4.955388
H	4.205653	-0.762813	-3.362940
C	2.877145	-3.909470	1.614795
H	3.271857	-4.764000	1.050098
H	1.923894	-4.224268	2.051498
C	-2.200142	-3.342563	1.089910
H	-2.278521	-3.556272	0.020311
H	-2.442095	-4.259449	1.638077
H	-1.166022	-3.062928	1.308451
H	-2.574920	-0.075241	-5.365692

H	3.574991	-3.710232	2.433757
F	-2.215808	2.903357	5.412087
F	3.806359	4.542009	2.304109

TS (C=O)

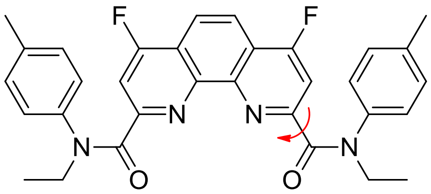

E = -1804.41023012 Eh, G = -1803.93511129 Eh, NImag = 1

O	-0.674704	-1.347311	-1.424992
O	5.773141	1.426239	-1.798695
N	-2.866518	-1.342675	-0.901412
N	-0.088345	0.995437	-0.385731
N	4.597763	-0.382337	-1.042517
N	2.602269	1.615765	-0.424632
C	3.824650	-0.993374	0.008541
C	-1.623462	-0.733599	-0.947769
C	-5.756943	-0.514480	1.312107
H	-6.055008	-0.664136	2.347000
C	1.731506	2.522483	0.059836
C	-1.379120	0.678162	-0.388123
C	2.549535	-1.507482	-0.250103
H	2.083393	-1.355365	-1.216511
C	0.308099	2.194623	0.075035
C	-4.523834	-0.999809	0.878754
H	-3.863345	-1.521287	1.565440
C	-2.927398	-2.727790	-1.425764
H	-3.764026	-3.219517	-0.920731
H	-2.001323	-3.227799	-1.134606
C	3.889987	1.923108	-0.458186
C	-0.142686	4.437971	1.070239
H	-0.873714	5.145770	1.445299
C	-1.952642	2.801170	0.546699
C	1.186568	4.737742	1.060345
H	1.546218	5.692841	1.427333
C	4.414731	3.160142	-0.020043
H	5.471197	3.379405	-0.109927
C	-4.114264	-0.802275	-0.445358

C	-2.370148	1.579098	0.073906
H	-3.425616	1.361666	0.070583
C	5.402655	-1.276317	-1.897034
H	5.569346	-0.751564	-2.840510
H	4.788615	-2.160357	-2.095772
C	2.138698	3.794819	0.553687
C	-6.617267	0.166975	0.438649
C	-0.598052	3.172684	0.578264
C	1.846081	-2.152278	0.762196
H	0.847409	-2.519793	0.543247
C	4.846161	0.960442	-1.134001
C	-6.200641	0.347991	-0.887786
H	-6.847584	0.875981	-1.584133
C	-4.968900	-0.134647	-1.331167
H	-4.652075	0.023345	-2.357544
C	3.655109	-1.766188	2.298243
H	4.092212	-1.856027	3.290085
C	4.373173	-1.121369	1.287754
H	5.359651	-0.713766	1.490301
C	3.522608	4.066784	0.494047
C	2.386106	-2.300033	2.049821
C	-7.963910	0.667106	0.906153
H	-8.279579	1.551323	0.344341
H	-7.947706	0.927291	1.968855
C	6.745496	-1.679455	-1.282363
H	7.365470	-0.794303	-1.117063
H	7.281027	-2.352284	-1.961373
H	6.607032	-2.201206	-0.330149
C	1.614181	-3.019456	3.132042
H	1.580371	-4.100433	2.945928
H	0.576415	-2.670994	3.181260
C	-3.098319	-2.797285	-2.944575
H	-4.026355	-2.312769	-3.265295
H	-3.135499	-3.843178	-3.268761
H	-2.253552	-2.314394	-3.441674
H	-8.737699	-0.099116	0.769986
H	2.067172	-2.868980	4.116555
F	-2.869374	3.679969	0.996633

F	3.955565	5.259096	0.945140

4b (oxo-form)

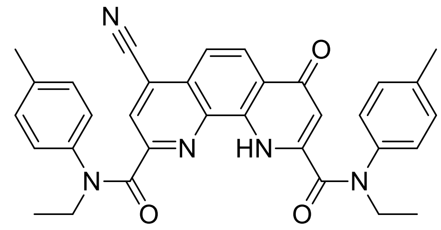

E = -1773.42102560 Eh, G = -1772.92871261 Eh, NImag = 0

O	5.996116	-0.689510	-0.858199
O	-0.794509	-1.183093	-2.036932
O	-1.953639	4.179007	0.195088
N	2.820628	0.562306	-0.152586
N	0.135895	0.772939	-0.584268
N	4.166390	-2.000748	-0.469586
N	-2.995865	-0.764890	-1.637274
C	4.842656	-0.806601	-0.444991
C	4.125557	0.439579	0.031220
C	4.925039	1.483392	0.552424
H	5.994114	1.336547	0.644123
C	4.320219	2.675364	0.914738
C	2.912948	2.846718	0.728218
C	2.200962	4.039381	1.041946
H	2.740014	4.881249	1.464720
C	0.852859	4.116054	0.796255
H	0.284949	5.015132	1.013427
C	0.131606	3.025933	0.239451
C	-1.317185	3.147234	-0.040821
C	-1.926953	1.951626	-0.620554
H	-2.981420	2.006104	-0.848767
C	-1.205793	0.816637	-0.862241
C	0.805492	1.840115	-0.060070
C	2.222317	1.728896	0.174847
C	-1.662500	-0.461994	-1.535166
C	3.030188	-2.298304	0.354272
C	3.126857	-2.231507	1.748537
H	4.063207	-1.924569	2.205697
C	2.028328	-2.554668	2.545161
H	2.118379	-2.488848	3.626888
C	0.819039	-2.974592	1.976392
C	0.740260	-3.045104	0.577663
H	-0.189829	-3.351580	0.106333
C	1.827301	-2.711902	-0.228218
H	1.732183	-2.741459	-1.309301

C	-0.354096	-3.364400	2.845477
C	4.848086	-3.140186	-1.119702
H	5.408410	-2.737316	-1.965461
H	4.067106	-3.802424	-1.505083
C	5.785189	-3.901209	-0.179235
H	6.235068	-4.750433	-0.704653
H	5.244858	-4.288288	0.690640
C	-4.022930	-0.269940	-0.764729
C	-3.941829	-0.462714	0.618824
H	-3.078472	-0.970010	1.039289
C	-4.955732	0.008915	1.450862
H	-4.870804	-0.134764	2.525087
C	-6.082325	0.659266	0.928631
C	-6.158048	0.828317	-0.461164
H	-7.016381	1.337919	-0.891577
C	-5.144009	0.372671	-1.301959
H	-5.206856	0.535420	-2.374087
C	-7.190226	1.142961	1.833974
H	-6.798815	1.481829	2.797934
C	-3.349450	-1.920447	-2.493056
H	-2.648386	-1.930348	-3.329274
H	-4.352720	-1.730019	-2.884334
C	-3.310824	-3.258335	-1.752507
H	-3.629068	-4.064828	-2.421604
H	-3.980430	-3.252878	-0.886535
H	-2.294497	-3.477080	-1.414714
H	-7.741574	1.972378	1.381490
H	-7.912064	0.342014	2.038951
H	-0.403989	-2.751390	3.750984
H	-1.302869	-3.258111	2.310087
H	-0.279552	-4.411242	3.166698
H	6.589474	-3.246170	0.165775
H	0.656907	-0.056933	-0.855960
C	5.107197	3.740785	1.466540
N	5.737343	4.607098	1.919796

4b (oxy-form)

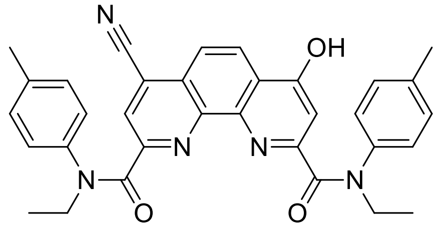

E = -1773.40353522 Eh, G = -1772.91094542 Eh, NImag = 0

O	0.522955	2.102921	-3.412176

O	-2.260275	-2.794866	3.452356
O	-0.820215	1.448072	5.756206
N	0.552442	1.337161	-0.118236
N	-0.785358	-0.026401	1.858286
N	1.535656	0.188683	-2.676048
N	-2.583201	-2.258389	1.262980
C	1.032959	1.448423	-2.501472
C	1.074196	2.042769	-1.109400
C	1.560850	3.357494	-0.958617
H	1.925947	3.907676	-1.817930
C	1.568266	3.910854	0.311875
C	1.024213	3.175136	1.407067
C	0.994300	3.683261	2.743891
H	1.430729	4.656820	2.944865
C	0.420724	2.959610	3.747856
H	0.398186	3.343067	4.761824
C	-0.185531	1.690902	3.483854
C	-0.835618	0.937475	4.499084
C	-1.454506	-0.251848	4.167317
H	-1.957215	-0.873193	4.901380
C	-1.421395	-0.675164	2.821884
C	-0.177182	1.137650	2.173714
C	0.484922	1.889056	1.112023
C	-2.128607	-1.986380	2.530017
C	2.403349	-0.453848	-1.727087
C	3.709083	0.008312	-1.536620
H	4.055648	0.876609	-2.089782
C	4.558322	-0.640253	-0.638078
H	5.568371	-0.264483	-0.493525
C	4.132837	-1.768667	0.073110
C	2.819607	-2.220434	-0.130314
H	2.459081	-3.081604	0.427040
C	1.960975	-1.572699	-1.014178
H	0.934287	-1.902463	-1.130801
C	5.062885	-2.492239	1.018721
C	1.379172	-0.434922	-4.003965
H	0.424689	-0.090493	-4.408006
H	1.321869	-1.516623	-3.847020

C	2.512657	-0.089616	-4.972629
H	2.355136	-0.594791	-5.931714
H	3.482771	-0.407832	-4.577647
C	-2.756162	-1.294129	0.213799
C	-3.574027	-0.174778	0.384253
H	-4.079962	-0.020014	1.332908
C	-3.719938	0.751503	-0.650340
H	-4.342090	1.629394	-0.493389
C	-3.086285	0.570500	-1.885037
C	-2.312249	-0.587678	-2.058110
H	-1.818923	-0.760636	-3.011492
C	-2.143438	-1.505077	-1.026714
H	-1.518699	-2.382148	-1.170488
C	-3.204389	1.586344	-2.996937
H	-3.858625	2.415797	-2.713188
C	-3.184499	-3.592826	1.055201
H	-2.626136	-4.300606	1.670433
H	-3.038486	-3.854019	0.003404
C	-4.670952	-3.637763	1.416964
H	-5.078750	-4.633954	1.213601
H	-5.241644	-2.910305	0.830624
H	-4.806867	-3.421470	2.479770
H	-2.220807	1.999512	-3.250444
H	-3.611998	1.135512	-3.909771
H	5.840037	-1.825989	1.405184
H	4.519089	-2.910347	1.871649
H	5.568553	-3.326600	0.515803
H	2.539604	0.988334	-5.153644
C	2.110021	5.223215	0.520063
N	2.551455	6.285198	0.695413
H	-1.291812	0.848288	6.350379

7b (oxo-form)

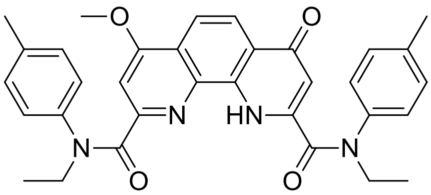

E = -1795.69984250 Eh, G = -1795.17324337 Eh, NImag = 0

O	5.845812	-4.484769	3.389625
O	-0.966944	-5.585624	2.359507

O	-2.455284	-0.131586	4.098866
N	2.573449	-3.413215	3.986659
N	-0.124109	-3.437704	3.620625
N	4.113977	-5.870117	3.920330
N	-3.164397	-5.303025	2.884980
C	4.697715	-4.629768	3.811962
C	3.885509	-3.396102	4.149062
C	4.616667	-2.245779	4.527586
H	5.693356	-2.324864	4.588363
C	3.930990	-1.068816	4.769158
C	2.510566	-1.040007	4.608468
C	1.719966	0.128227	4.812855
H	2.210672	1.044009	5.121318
C	0.363918	0.083326	4.608327
H	-0.264685	0.957228	4.745792
C	-0.284213	-1.112470	4.198329
C	-1.743970	-1.132719	3.955690
C	-2.272080	-2.426516	3.529202
H	-3.333245	-2.479610	3.332102
C	-1.469647	-3.523989	3.385774
C	0.473956	-2.270456	4.006980
C	1.898945	-2.257315	4.205870
C	-1.854297	-4.890300	2.861884
C	2.996324	-6.169056	4.767642
C	3.067318	-5.944244	6.146848
H	3.965135	-5.503330	6.570301
C	1.991573	-6.277151	6.969789
H	2.060163	-6.086081	8.038262
C	0.832303	-6.863620	6.445889
C	0.779004	-7.091303	5.063082
H	-0.113714	-7.529033	4.624411
C	1.842791	-6.749961	4.230080
H	1.765361	-6.902622	3.157989
C	-0.313585	-7.261629	7.347268
C	4.890634	-7.016953	3.405506
H	5.412626	-6.675534	2.509541
H	4.170426	-7.787459	3.114965
C	5.893632	-7.572777	4.419359
H	6.414323	-8.439373	3.997437
H	5.391754	-7.893154	5.338184
C	-4.157735	-4.827677	3.804751
C	-3.935526	-4.869182	5.185935
H	-2.987921	-5.240557	5.564926
C	-4.918245	-4.420400	6.066545

H	-4.723765	-4.444685	7.135936
C	-6.151546	-3.944742	5.600504
C	-6.366430	-3.925424	4.215312
H	-7.309632	-3.550860	3.825560
C	-5.385698	-4.358219	3.324557
H	-5.560547	-4.310421	2.253549
C	-7.224047	-3.489633	6.561761
H	-6.791271	-3.015773	7.448088
C	-3.477881	-6.548089	2.148785
H	-2.840668	-6.568237	1.263052
H	-4.519315	-6.473081	1.823607
C	-3.268852	-7.814904	2.980294
H	-3.565955	-8.696051	2.401558
H	-3.868624	-7.792871	3.895914
H	-2.214843	-7.922255	3.248721
H	-7.902662	-2.771903	6.091487
H	-7.831387	-4.335933	6.907759
H	-0.434020	-6.558733	8.177928
H	-1.259524	-7.304000	6.797927
H	-0.149619	-8.254784	7.785076
H	6.638005	-6.812128	4.668094
H	0.461984	-4.244264	3.422444
O	4.502506	0.097710	5.145453
C	5.919542	0.141043	5.292625
H	6.260253	-0.555765	6.067675
H	6.424260	-0.091870	4.347824
H	6.156299	1.162820	5.591409

7b (oxy-form)

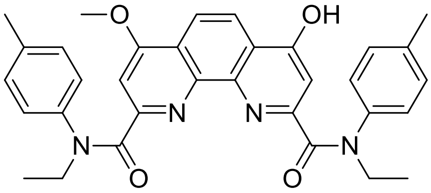

E = -1795.68044880 Eh, G = -1795.15434961 Eh, NImag = 0

O	0.420692	1.852911	-3.398735
O	-2.336973	-3.035174	3.526477
O	-0.917688	1.239605	5.791253
N	0.428070	1.087049	-0.096968
N	-0.882317	-0.269016	1.906026
N	1.418816	-0.053882	-2.632874
N	-2.662242	-2.518272	1.333802
C	0.915100	1.209973	-2.470129
C	0.938994	1.808110	-1.079586
C	1.412865	3.131058	-0.943726

H	1.757082	3.659933	-1.822690
C	1.413818	3.692213	0.320391
C	0.882008	2.945096	1.414537
C	0.849446	3.469627	2.744837
H	1.273022	4.450899	2.925535
C	0.292855	2.750980	3.762466
H	0.269443	3.144347	4.772563
C	-0.295287	1.472168	3.512021
C	-0.931579	0.718461	4.535813
C	-1.538398	-0.480297	4.219800
H	-2.031368	-1.099109	4.962654
C	-1.506693	-0.915287	2.877421
C	-0.284164	0.906785	2.205410
C	0.362780	1.652387	1.131291
C	-2.205806	-2.232332	2.598761
C	2.282421	-0.692364	-1.678498
C	3.585602	-0.226460	-1.480733
H	3.929503	0.646153	-2.028586
C	4.433884	-0.874021	-0.580432
H	5.441866	-0.494619	-0.430075
C	4.010228	-2.005991	0.125616
C	2.699633	-2.462081	-0.084827
H	2.339571	-3.325404	0.469550
C	1.842332	-1.815012	-0.970106
H	0.816955	-2.146906	-1.090224
C	4.938543	-2.728531	1.074058
C	1.271508	-0.684886	-3.957208
H	0.319244	-0.344241	-4.369393
H	1.215358	-1.766095	-3.795840
C	2.409738	-0.343547	-4.922277
H	2.260147	-0.855330	-5.879264
H	3.378269	-0.656051	-4.518771
C	-2.840550	-1.566423	0.274572
C	-3.661581	-0.447665	0.433552
H	-4.163985	-0.282488	1.382234
C	-3.812221	0.466230	-0.611138
H	-4.435248	1.344964	-0.462289
C	-3.180274	0.273312	-1.844774
C	-2.404828	-0.885157	-2.006806
H	-1.912161	-1.067076	-2.958865
C	-2.231398	-1.790129	-0.965365
H	-1.604294	-2.666857	-1.100322
C	-3.298358	1.278993	-2.966049
H	-3.966755	2.101997	-2.696433

C	-3.258239	-3.856504	1.140367
H	-2.695264	-4.556522	1.760173
H	-3.115285	-4.127106	0.090505
C	-4.743491	-3.904882	1.507513
H	-5.148160	-4.904524	1.314588
H	-5.318869	-3.184886	0.916536
H	-4.876235	-3.679177	2.568709
H	-2.317009	1.703104	-3.210293
H	-3.688727	0.816317	-3.880541
H	5.718138	-2.063077	1.457117
H	4.393714	-3.140020	1.929601
H	5.441188	-3.567656	0.575903
H	2.434009	0.733440	-5.109100
H	-1.380241	0.635632	6.388021
O	1.876928	4.930598	0.620001
C	2.391020	5.732624	-0.438148
H	3.255762	5.256025	-0.915616
H	1.623598	5.932707	-1.195300
H	2.701538	6.671359	0.022311

2b (oxo-amino-form)

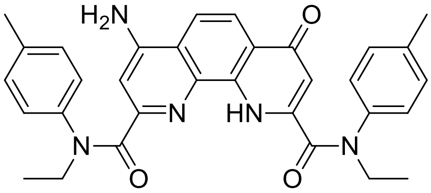

E = -1736.56380277 Eh, G = -1736.05202038 Eh, NImag = 0

O	5.985411	-0.893251	-0.791239
O	-0.788834	-1.143445	-2.201669
O	-1.867749	4.190713	0.114806
N	2.825655	0.466810	-0.186596
N	0.179320	0.755968	-0.661071
N	4.097347	-2.122019	-0.434399
N	-2.973971	-0.727105	-1.713321
C	4.816066	-0.948449	-0.408851
C	4.126840	0.327051	0.029047
C	4.944292	1.348570	0.543995
H	6.009894	1.166877	0.632409
C	4.387836	2.576219	0.888250
C	2.977005	2.754870	0.698499
C	2.272038	3.953476	1.024214
H	2.793500	4.778399	1.500427
C	0.929326	4.068436	0.767219
H	0.376247	4.972008	1.002214
C	0.197655	3.001801	0.181813

C	-1.243711	3.148718	-0.117805
C	-1.863622	1.967253	-0.713288
H	-2.914842	2.036009	-0.954508
C	-1.155325	0.822851	-0.954505
C	0.858831	1.808699	-0.116304
C	2.268512	1.656956	0.137139
C	-1.633305	-0.433668	-1.648739
C	2.931279	-2.373970	0.360812
C	2.985322	-2.273581	1.755694
H	3.910920	-1.965073	2.232977
C	1.858882	-2.560375	2.525975
H	1.916859	-2.467141	3.608033
C	0.661246	-2.978789	1.931576
C	0.624168	-3.083895	0.533763
H	-0.295547	-3.392492	0.043393
C	1.740087	-2.785583	-0.246560
H	1.677284	-2.839797	-1.329059
C	-0.543038	-3.329447	2.774824
C	4.759184	-3.293258	-1.044589
H	5.354084	-2.924740	-1.882321
H	3.969503	-3.940277	-1.438300
C	5.647913	-4.064751	-0.066039
H	6.084910	-4.938175	-0.562346
H	5.073419	-4.417131	0.796854
C	-3.956740	-0.275395	-0.770525
C	-3.789524	-0.494844	0.601678
H	-2.891464	-0.989587	0.959691
C	-4.761708	-0.064574	1.502757
H	-4.608536	-0.228687	2.566559
C	-5.932218	0.569238	1.063680
C	-6.094383	0.765804	-0.314779
H	-6.987929	1.264298	-0.682021
C	-5.122075	0.352927	-1.224243
H	-5.252840	0.538437	-2.286494
C	-6.994685	1.006242	2.044359
H	-6.553380	1.342265	2.987816
C	-3.362345	-1.848702	-2.597430
H	-2.701507	-1.819283	-3.465533
H	-4.383807	-1.650620	-2.933985
C	-3.279813	-3.215962	-1.916156
H	-3.626281	-3.997434	-2.600896
H	-3.904855	-3.250769	-1.017999
H	-2.246275	-3.438070	-1.638851
H	-7.596392	1.825981	1.641245

H	-7.679518	0.181784	2.281138
H	-0.644220	-2.654005	3.630680
H	-1.468382	-3.280353	2.192205
H	-0.466154	-4.348550	3.175236
H	6.462193	-3.426729	0.287222
H	0.703561	-0.073955	-0.926121
N	5.160636	3.582958	1.430095
H	4.847919	4.531858	1.289310
H	6.159813	3.472887	1.332331

2b (oxy-amino-form)

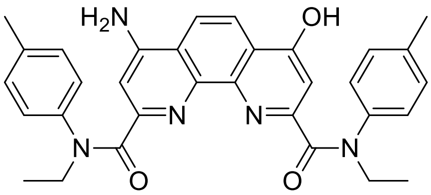

E = -1736.54281604 Eh, G = -1736.03169360 Eh, NImag = 0

O	0.501964	2.113432	-3.427833
O	-2.223411	-2.795492	3.445092
O	-0.851898	1.478552	5.742013
N	0.529582	1.349016	-0.133684
N	-0.776448	-0.012260	1.850923
N	1.518245	0.211390	-2.675705
N	-2.551622	-2.264296	1.256176
C	1.011841	1.473922	-2.506013
C	1.056681	2.068556	-1.114435
C	1.572246	3.364635	-0.961287
H	1.954590	3.898101	-1.826139
C	1.601828	3.935116	0.306110
C	1.018993	3.196744	1.386727
C	0.946204	3.713987	2.719791
H	1.327215	4.708062	2.933848
C	0.369505	3.000132	3.729410
H	0.316506	3.401803	4.734988
C	-0.207781	1.719473	3.469980
C	-0.853012	0.961426	4.484461
C	-1.453805	-0.237285	4.157861
H	-1.951272	-0.861216	4.893385
C	-1.408001	-0.665012	2.813498
C	-0.181770	1.162900	2.160515
C	0.478071	1.911437	1.093342
C	-2.098835	-1.984343	2.523515
C	2.388857	-0.430000	-1.728464
C	3.726494	-0.038357	-1.622110
H	4.091931	0.783856	-2.230642

C	4.582257	-0.694623	-0.734723
H	5.618957	-0.374944	-0.658149
C	4.128102	-1.758694	0.053046
C	2.780763	-2.135880	-0.060677
H	2.397657	-2.942339	0.560015
C	1.917985	-1.481445	-0.935278
H	0.867308	-1.745143	-0.979730
C	5.060827	-2.493326	0.987664
C	1.345444	-0.426602	-3.992870
H	0.385424	-0.086525	-4.387846
H	1.290593	-1.506989	-3.824190
C	2.461538	-0.093269	-4.986299
H	2.284921	-0.605670	-5.938482
H	3.438563	-0.410705	-4.607834
C	-2.737288	-1.304529	0.205344
C	-3.555631	-0.186001	0.379010
H	-4.052242	-0.028165	1.332007
C	-3.711022	0.737201	-0.656660
H	-4.331185	1.615891	-0.496090
C	-3.086890	0.554215	-1.895802
C	-2.315430	-0.604588	-2.073064
H	-1.829283	-0.779529	-3.029723
C	-2.137210	-1.519090	-1.040698
H	-1.512880	-2.395858	-1.188490
C	-3.207192	1.571544	-3.005930
H	-3.884471	2.385512	-2.731046
C	-3.138023	-3.605200	1.052672
H	-2.569619	-4.306182	1.666538
H	-2.993953	-3.866780	0.000716
C	-4.622544	-3.667071	1.420640
H	-5.020528	-4.667833	1.219586
H	-5.203468	-2.945998	0.836378
H	-4.755770	-3.451300	2.483858
H	-2.227707	2.006665	-3.238046
H	-3.587015	1.115930	-3.928340
H	5.916881	-1.873095	1.270401
H	4.547403	-2.800632	1.904426
H	5.457032	-3.403277	0.518666
H	2.486893	0.983326	-5.175645
H	-1.321904	0.873138	6.331483
N	2.122325	5.201966	0.505229
H	2.680804	5.566477	-0.253184
H	2.517338	5.400252	1.412278

2b (oxy-imino-form)

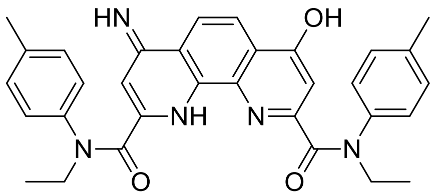

E = -1736.54058199 Eh, G = -1736.02820847 Eh, NImag = 0

O	6.029094	-0.872143	-0.703389
O	-0.809511	-1.183496	-2.173557
N	-1.830941	4.273343	0.067165
N	2.841081	0.466585	-0.165282
N	0.183371	0.733811	-0.648540
N	4.139060	-2.115998	-0.417416
N	-2.989037	-0.730876	-1.689741
C	4.846789	-0.937589	-0.363472
C	4.139140	0.334261	0.053991
C	4.951352	1.367708	0.572751
H	6.016703	1.190948	0.681047
C	4.369094	2.578314	0.893776
C	2.973955	2.769052	0.676426
C	2.289761	3.985265	0.953482
H	2.845309	4.818318	1.368815
C	0.947548	4.092960	0.683458
H	0.401986	5.010536	0.875365
C	0.208468	3.011260	0.133487
C	-1.231742	3.150515	-0.176339
C	-1.848397	1.959384	-0.753194
H	-2.896827	2.008069	-1.017142
C	-1.149910	0.806689	-0.959303
C	0.862756	1.805992	-0.129133
C	2.268520	1.659337	0.133057
C	-1.641776	-0.452584	-1.630605
C	2.951586	-2.383679	0.340317
C	2.961819	-2.292884	1.736936
H	3.868995	-1.978352	2.244726
C	1.815532	-2.597565	2.469869
H	1.838956	-2.511869	3.553851
C	0.641312	-3.024239	1.835542
C	0.648406	-3.119469	0.436750
H	-0.252048	-3.432359	-0.085099
C	1.784679	-2.803592	-0.306408
H	1.756348	-2.850974	-1.390611
C	-0.584787	-3.392645	2.638922
C	4.831294	-3.278893	-1.010426
H	5.449393	-2.901436	-1.827140

H	4.060835	-3.931416	-1.432011
C	5.695709	-4.045950	-0.007082
H	6.158224	-4.911808	-0.493407
H	5.097644	-4.409459	0.834939
C	-3.961307	-0.266840	-0.743349
C	-3.775598	-0.456196	0.631353
H	-2.869600	-0.936970	0.987890
C	-4.739212	-0.014549	1.536145
H	-4.571372	-0.156928	2.600872
C	-5.921037	0.600590	1.100276
C	-6.102115	0.767870	-0.279693
H	-7.005765	1.248854	-0.646037
C	-5.137890	0.344114	-1.193256
H	-5.285980	0.505300	-2.257267
C	-6.975965	1.046077	2.085444
H	-6.527544	1.396838	3.020084
C	-3.391503	-1.856611	-2.562075
H	-2.725037	-1.848575	-3.426165
H	-4.407522	-1.647058	-2.908276
C	-3.335707	-3.216853	-1.864278
H	-3.688938	-4.000978	-2.542519
H	-3.967817	-3.231725	-0.970505
H	-2.308043	-3.451687	-1.575948
H	-7.586743	1.856909	1.677445
H	-7.654639	0.221756	2.339555
H	-0.726325	-2.715599	3.487847
H	-1.490206	-3.361947	2.024732
H	-0.503970	-4.408530	3.046628
H	6.491267	-3.401285	0.375505
H	0.705987	-0.099713	-0.899081
O	5.061394	3.626733	1.405955
H	5.994349	3.384558	1.484391
H	-2.815564	4.209073	-0.204206

2b (oxo-imino-form)

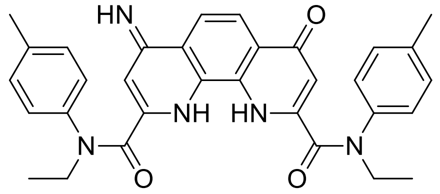

E = -1736.52955984 Eh, G = -1736.01791060 Eh, NImag = 0

O	6.052049	-0.962027	-0.933596
O	-0.867951	-1.101159	-2.186881
N	-1.860959	4.175227	0.455460
N	2.888465	0.445573	-0.220834

N	0.116258	0.720564	-0.651108
N	4.171505	-2.174150	-0.494123
N	-3.058156	-0.700174	-1.731220
C	4.905307	-0.998735	-0.503625
C	4.245369	0.286399	-0.049372
C	5.018383	1.304169	0.422148
H	6.090192	1.166990	0.487602
C	4.446485	2.578181	0.842850
C	2.978045	2.696752	0.649988
C	2.309595	3.885628	1.010319
H	2.912964	4.686934	1.423263
C	0.946562	4.003721	0.846595
H	0.410008	4.903160	1.126478
C	0.192801	2.947044	0.288644
C	-1.271024	3.081887	0.093628
C	-1.930724	1.928543	-0.514031
H	-2.996641	1.989851	-0.686445
C	-1.239157	0.813163	-0.872362
C	0.841733	1.763756	-0.095616
C	2.243586	1.626070	0.116434
C	-1.724511	-0.405465	-1.624171
C	3.067594	-2.419292	0.387123
C	3.193751	-2.237971	1.771455
H	4.140514	-1.900769	2.182435
C	2.107930	-2.478786	2.610963
H	2.221239	-2.322424	3.680895
C	0.878949	-2.926304	2.104376
C	0.767729	-3.111610	0.719838
H	-0.175699	-3.446224	0.296321
C	1.843018	-2.861771	-0.133775
H	1.727268	-2.992186	-1.206256
C	-0.279700	-3.217427	3.028733
C	4.806227	-3.353895	-1.124274
H	5.354688	-2.992157	-1.995697
H	3.998741	-4.006412	-1.469544
C	5.746642	-4.109244	-0.183258
H	6.158303	-4.987564	-0.691846
H	5.220404	-4.453196	0.713189
C	-4.080758	-0.236242	-0.837384
C	-4.010850	-0.504263	0.534376
H	-3.156571	-1.044352	0.931355
C	-5.022422	-0.062744	1.385506
H	-4.946558	-0.266160	2.450740
C	-6.136222	0.632446	0.893639

C	-6.200561	0.878681	-0.485239
H	-7.049618	1.422022	-0.892293
C	-5.188254	0.453037	-1.344333
H	-5.242741	0.672714	-2.406758
C	-7.243634	1.080743	1.817831
H	-6.854034	1.366339	2.799722
C	-3.422035	-1.810764	-2.641081
H	-2.740599	-1.768898	-3.492396
H	-4.435690	-1.609333	-2.998214
C	-3.352815	-3.185187	-1.973042
H	-3.670949	-3.960385	-2.678172
H	-4.007737	-3.235140	-1.097164
H	-2.327630	-3.404651	-1.663672
H	-7.785803	1.936771	1.405663
H	-7.973844	0.277399	1.978518
H	-0.363559	-2.461826	3.816546
H	-1.228251	-3.248013	2.484349
H	-0.155515	-4.188578	3.524081
H	6.576866	-3.465618	0.117809
H	0.550653	-0.009591	-1.206431
O	5.121989	3.494775	1.318627
H	-2.863252	4.114174	0.257450
H	2.336004	-0.384260	-0.376668

2a (anion)

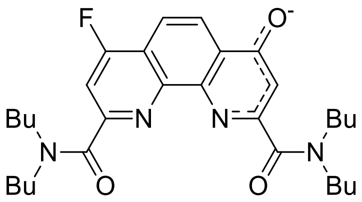

E = -1712.235593 Eh, G = -1711.644493 Eh, NImag = 0

F	4.987858	3.153359	-0.090467
O	4.387272	-3.894598	0.028213
O	-0.901262	-4.019719	-0.764883
O	-0.078311	4.211352	0.709934
N	-0.888873	2.928959	-0.999016
N	1.057319	-1.393885	-0.065350
N	1.325056	1.296645	-0.227253
N	-1.279018	-2.676738	1.035347
C	2.307691	-0.891740	-0.110867
C	2.449206	0.552470	-0.167472
C	1.415233	2.616525	-0.223972
C	3.750407	1.134594	-0.136847
C	0.956789	-2.730679	0.032041
C	0.094885	3.342410	-0.154742

C	2.640645	3.311736	-0.148501
H	2.682467	4.393019	-0.113881
C	3.483346	-1.680761	-0.067718
C	-0.473053	-3.217138	0.076869
C	-0.796090	-1.886304	2.170763
H	0.279208	-2.033925	2.248644
H	-1.246510	-2.309042	3.080032
C	-2.265202	3.320443	-0.690202
H	-2.847726	3.242412	-1.613303
H	-2.270972	4.366990	-0.373367
C	2.018250	-3.621493	0.045400
H	1.838687	-4.690127	0.106806
C	3.781879	2.546212	-0.129566
C	3.375728	-3.147632	0.000210
C	-1.120147	-0.392237	2.081495
H	-0.688337	-0.008714	1.153356
H	-2.207521	-0.261867	2.031524
C	4.910938	0.306447	-0.099650
H	5.891418	0.768344	-0.087299
C	-0.661200	2.124876	-2.204017
H	0.406718	2.137224	-2.422514
H	-1.159083	2.644713	-3.034081
C	-0.580660	0.407949	3.274896
H	-0.954791	-0.027923	4.212403
H	-0.995315	1.422643	3.226315
C	-2.722588	-2.884178	0.917953
H	-3.164361	-2.693516	1.901213
H	-2.908592	-3.931313	0.661974
C	-1.149006	0.675242	-2.138187
H	-2.239988	0.652044	-2.018513
H	-0.696947	0.188509	-1.269824
C	4.760417	-1.056742	-0.071855
H	5.623154	-1.714977	-0.039457
C	-0.759528	-0.085377	-3.410033
H	-1.117178	0.462655	-4.293528
H	0.336103	-0.104559	-3.485024
C	-3.357097	-1.970148	-0.136134
H	-2.951441	-2.239460	-1.115799
H	-3.046177	-0.937598	0.055033
C	-2.872290	2.433175	0.401798
H	-2.290705	2.568934	1.320936
H	-2.752532	1.384825	0.111091
C	-4.349425	2.720427	0.672178
H	-4.473085	3.766888	0.980792

H	-4.918717	2.606160	-0.260540
C	0.948212	0.502239	3.319764
H	1.416885	-0.482621	3.413033
H	1.280670	1.106351	4.170596
H	1.329435	0.960886	2.403982
C	-4.884069	-2.043702	-0.164256
H	-5.201468	-3.074838	-0.368643
H	-5.279994	-1.793076	0.829697
C	-5.488415	-1.100668	-1.207568
H	-5.127326	-1.345700	-2.212632
H	-5.208906	-0.060837	-1.002575
H	-6.581672	-1.156996	-1.220059
C	-4.930625	1.792768	1.742233
H	-4.389093	1.898883	2.688760
H	-4.852583	0.744024	1.434479
H	-5.986973	2.007333	1.933042
C	-1.295348	-1.517782	-3.446754
H	-0.917767	-2.109984	-2.608377
H	-1.005637	-2.025966	-4.372873
H	-2.390341	-1.526790	-3.392966

TS (O-alkylation)

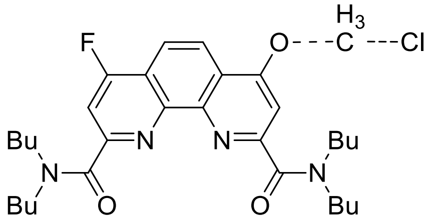

E = -2212.338102 Eh, G = -2211.715180 Eh, NImag = 1

F	0.208795	-6.043257	0.180779
O	-5.025138	-1.416609	-0.321192
O	-1.957463	2.872902	-1.252571
O	4.174166	-2.827244	0.799638
N	3.760237	-1.405834	-0.935018
N	-1.055403	-0.143250	-0.157756
N	0.982759	-2.006011	-0.152563
N	-1.076368	2.720462	0.841073
C	-1.378886	-1.458341	-0.162802
C	-0.299502	-2.440034	-0.108278
C	1.967790	-2.888024	-0.070184
C	-0.631603	-3.828922	-0.022565
C	-2.074172	0.719288	-0.224911
C	3.389759	-2.372742	-0.044849
C	1.765245	-4.279243	0.078062
H	2.598082	-4.962661	0.188339
C	-2.716201	-1.935092	-0.196443

C	-1.699952	2.188648	-0.255905
C	-0.874534	1.998983	2.102523
H	-1.497994	1.105804	2.086571
H	-1.250277	2.637671	2.915799
C	5.113138	-0.853744	-0.787345
H	5.387751	-0.404780	-1.747959
H	5.804288	-1.680712	-0.587743
C	-3.427738	0.380850	-0.308272
H	-4.171011	1.164467	-0.393895
C	0.463994	-4.710724	0.077921
C	-3.810905	-0.980131	-0.281192
C	0.586911	1.615484	2.376171
H	0.917637	0.944460	1.575255
H	1.209449	2.518331	2.325005
C	-1.992503	-4.261267	-0.039089
H	-2.212357	-5.321304	0.021980
C	3.009545	-1.056985	-2.150961
H	2.222255	-1.796598	-2.296847
H	3.705166	-1.159889	-2.997476
C	0.797396	0.949484	3.747260
H	0.432238	1.620073	4.539881
H	1.877057	0.843232	3.914956
C	-0.739046	4.147932	0.794567
H	-0.645672	4.493680	1.830594
H	-1.580416	4.678426	0.334927
C	2.391652	0.345476	-2.157795
H	3.171304	1.096233	-1.965057
H	1.659167	0.406171	-1.345896
C	-2.993201	-3.332496	-0.135444
H	-4.035853	-3.631662	-0.158713
C	1.716242	0.649978	-3.502802
H	2.448702	0.526220	-4.315262
H	0.927976	-0.093573	-3.682830
C	0.542068	4.478371	0.014588
H	0.433174	4.098594	-1.006582
H	1.394472	3.953176	0.464453
C	5.236228	0.189117	0.332619
H	4.921300	-0.273840	1.274354
H	4.545493	1.018579	0.134644
C	6.666808	0.724715	0.473170
H	7.349500	-0.114391	0.666406
H	6.988656	1.169065	-0.480074
C	0.137158	-0.428701	3.895761
H	-0.954753	-0.372111	3.832200

H	0.385349	-0.878899	4.863785
H	0.478278	-1.110479	3.109476
C	0.823145	5.986170	-0.020452
H	-0.034695	6.502293	-0.472885
H	0.904183	6.369668	1.007351
C	2.096784	6.343299	-0.796169
H	2.030697	6.005048	-1.836759
H	2.978644	5.867894	-0.349712
H	2.270434	7.425578	-0.806628
C	6.811095	1.764373	1.591019
H	6.530299	1.339304	2.561701
H	6.164357	2.631279	1.410818
H	7.842116	2.127671	1.670484
C	1.106026	2.054230	-3.575025
H	0.315252	2.184712	-2.829784
H	0.662904	2.242251	-4.559584
H	1.868573	2.824165	-3.400844
C	-6.550966	-0.216674	-0.198703
H	-7.178588	-1.088125	-0.170888
H	-6.210040	0.226021	0.720401
H	-6.349744	0.264558	-1.139354
Cl	-8.504956	1.108475	-0.010330

TS (N-alkylation)

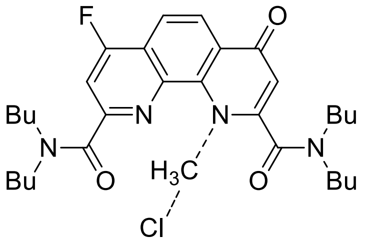

E = -2212.326531 Eh, G = -2211.700623 Eh, NImag = 1

F	1.063224	6.128396	0.444592
O	3.921299	2.404420	-1.189098
O	-3.631863	-1.405782	-1.939945
O	-5.348036	3.380019	0.092112
N	0.584869	2.085215	-0.241677
N	-1.996857	0.944726	-0.438018
N	3.143692	0.516724	-0.165403
N	-3.287619	-1.873741	0.270061
C	3.039463	1.834531	-0.539730
C	1.792559	2.637449	-0.200497
C	2.011765	4.018140	0.011455
H	3.023469	4.422919	-0.010502
C	0.908166	4.804886	0.207443
C	-0.403771	4.282805	0.156175

C	-1.550360	5.099317	0.336200
H	-1.419601	6.165858	0.528231
C	-2.791128	4.520713	0.256887
H	-3.719956	5.087206	0.383583
C	-2.963841	3.143570	-0.005070
C	-4.362349	2.642011	-0.073870
C	-4.419632	1.226433	-0.339099
H	-5.399646	0.745660	-0.410315
C	-3.268716	0.477882	-0.496418
C	-1.849755	2.277855	-0.195371
C	-0.513620	2.870092	-0.097530
C	-3.421635	-1.004994	-0.798714
C	4.322866	-0.194112	-0.667082
H	4.573139	0.260355	-1.636983
H	4.037660	-1.242893	-0.844602
C	5.543602	-0.103738	0.259165
H	5.289048	-0.493438	1.261963
C	-3.405405	-3.304930	-0.049295
H	-4.209418	-3.415878	-0.798712
H	-3.708222	-3.829236	0.872884
C	-2.119579	-3.913247	-0.623777
H	-1.305536	-3.842510	0.119499
H	-1.801244	-3.320103	-1.496756
H	5.808639	0.961351	0.377423
C	2.373510	-0.094041	0.930202
H	1.492693	0.541274	1.075957
H	2.976549	-0.062772	1.861631
C	-3.376382	-1.419737	1.661022
H	-3.738888	-0.381716	1.633194
H	-4.158560	-2.018656	2.168423
C	-2.299580	-5.374622	-1.047501
H	-2.652339	-5.977429	-0.188503
H	-3.094766	-5.429085	-1.813835
C	-1.004416	-5.974586	-1.607857
H	-1.156310	-7.006322	-1.966332
H	-0.626749	-5.365451	-2.445102
H	-0.215025	-5.995050	-0.837777
C	-2.085801	-1.459733	2.496777
H	-1.274227	-0.994934	1.910113
H	-2.253488	-0.800135	3.369726
C	-1.665180	-2.840863	3.012541
H	-2.528308	-3.313164	3.519223
H	-1.403588	-3.497971	2.165751
C	-0.481288	-2.769953	3.984095

H	-0.726906	-2.143621	4.858557
H	-0.199849	-3.770442	4.351491
H	0.404000	-2.328875	3.497200
C	6.742901	-0.879524	-0.296088
H	6.459966	-1.939607	-0.434665
H	6.988599	-0.492724	-1.301941
C	7.979882	-0.791720	0.604248
H	8.828559	-1.355574	0.183164
H	8.300422	0.255936	0.731698
H	7.768725	-1.199210	1.607462
C	1.909591	-1.540946	0.669881
H	0.909905	-1.652397	1.127863
H	1.756939	-1.691920	-0.415058
C	2.809961	-2.645540	1.242698
H	2.924264	-2.499676	2.334061
H	3.826606	-2.573623	0.817065
C	2.244974	-4.042919	0.961664
H	2.907362	-4.835051	1.348464
H	1.256047	-4.168660	1.434258
H	2.114299	-4.195547	-0.122121
C	-0.579038	-0.443088	-1.667057
H	-1.304660	-0.351591	-2.467995
H	0.182337	0.312947	-1.505502
H	-0.777122	-1.155569	-0.876236
Cl	0.760576	-1.972489	-2.832915

Product (O-alkylation)

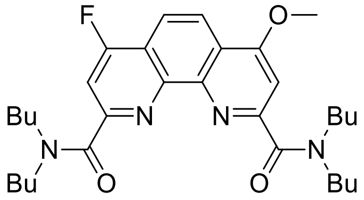

E = -1752.034578 Eh, G = -1751.404599 Eh, NImag = 0

F	3.846569	-4.448591	0.148202
O	5.122603	2.432623	0.010840
O	0.092181	4.017330	0.668836
O	-1.321784	-4.122017	-0.782084
N	-1.764227	-2.745491	0.991256
N	1.242001	0.949175	-0.059670
N	0.785616	-1.706516	0.149819
N	-0.751455	2.783645	-1.059375
C	2.313786	0.134058	0.033140
C	2.066296	-1.290833	0.105261
C	0.533278	-3.005065	0.183003
C	3.169134	-2.183517	0.123356

C	1.458781	2.251888	-0.134805
C	-0.933377	-3.363159	0.113145
C	1.537269	-3.995361	0.151290
H	1.295833	-5.050623	0.144688
C	3.650374	0.599021	0.037235
C	0.214520	3.109616	-0.164096
C	-0.560936	1.879342	-2.199703
H	0.507381	1.719802	-2.332995
H	-0.917837	2.407215	-3.093972
C	-3.203117	-2.782686	0.716297
H	-3.725360	-2.602888	1.660697
H	-3.465846	-3.785403	0.368995
C	2.734290	2.846411	-0.085404
H	2.818251	3.923869	-0.112528
C	2.840352	-3.556896	0.142896
C	3.842163	2.013770	-0.013237
C	-1.288197	0.538979	-2.066919
H	-0.943692	0.048513	-1.152634
H	-2.363450	0.723199	-1.965373
C	4.507283	-1.679390	0.116655
H	5.331957	-2.381842	0.139498
C	-1.311536	-2.036413	2.192614
H	-0.278428	-2.325645	2.388310
H	-1.906392	-2.415565	3.033440
C	-1.054408	-0.383802	-3.271119
H	-1.320766	0.144305	-4.197277
H	-1.746443	-1.231126	-3.196158
C	-2.077655	3.384529	-0.886030
H	-2.598558	3.309077	-1.845015
H	-1.954164	4.445343	-0.651637
C	-1.410706	-0.508798	2.135549
H	-2.463496	-0.202667	2.090759
H	-0.916727	-0.155307	1.226863
C	4.736214	-0.333055	0.083817
H	5.748343	0.052785	0.083674
C	-0.744668	0.125175	3.360972
H	-1.177097	-0.300144	4.277224
H	0.317729	-0.153834	3.366413
C	-2.885274	2.692170	0.216995
H	-2.383636	2.861185	1.174624
H	-2.869682	1.610563	0.044938
C	-3.606017	-1.737270	-0.328654
H	-3.093073	-1.972204	-1.268214
H	-3.237565	-0.757305	-0.008995

C	-5.114672	-1.659278	-0.564383
H	-5.486373	-2.633273	-0.908223
H	-5.621808	-1.455274	0.388369
C	0.375836	-0.924042	-3.372132
H	1.111735	-0.120940	-3.483954
H	0.484410	-1.590096	-4.234113
H	0.635156	-1.486769	-2.471607
C	-4.335079	3.172550	0.293978
H	-4.357158	4.253947	0.481805
H	-4.820997	3.020225	-0.679452
C	-5.127260	2.441979	1.380815
H	-4.674764	2.596062	2.366696
H	-5.146105	1.362452	1.191889
H	-6.163431	2.791143	1.428479
C	-5.481854	-0.575798	-1.581478
H	-4.999119	-0.764375	-2.546819
H	-5.154427	0.411534	-1.236332
H	-6.562321	-0.529210	-1.749434
C	-0.870128	1.649170	3.397537
H	-0.400534	2.112436	2.524852
H	-0.394350	2.064732	4.291994
H	-1.922349	1.955357	3.409319
C	5.374742	3.840018	-0.031893
H	6.458012	3.947093	-0.002235
H	4.984558	4.278898	-0.955612
H	4.929316	4.343016	0.832093

Product (N-alkylation)

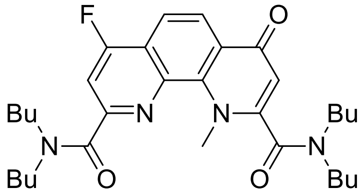

E = -1752.030735 Eh, G = -1751.399959 Eh, NImag = 0

F	-4.217791	3.974232	-1.417446
O	-4.852393	-1.089611	-1.563525
O	1.877693	-1.043490	2.006502
O	2.435069	4.391826	0.697177
N	-2.065387	0.601433	-0.499291
N	0.724230	0.731669	-0.028956
N	-3.683554	-1.681588	0.317681
N	3.672633	-0.920411	0.584010
C	-4.028605	-0.823253	-0.677278
C	-3.362654	0.531844	-0.731909
C	-4.147976	1.647755	-1.100390

H	-5.199905	1.541487	-1.328721
C	-3.519908	2.864375	-1.124740
C	-2.146110	3.010557	-0.821740
C	-1.507406	4.273773	-0.711544
H	-2.069416	5.174971	-0.923431
C	-0.210690	4.321356	-0.281312
H	0.304120	5.262172	-0.124275
C	0.531850	3.141465	-0.009669
C	1.895770	3.285437	0.540341
C	2.511759	2.034387	0.918291
H	3.460396	2.059457	1.439669
C	1.939012	0.838605	0.605563
C	-0.037114	1.877365	-0.222344
C	-1.440123	1.799969	-0.555259
C	2.512562	-0.468047	1.115615
C	-4.175001	-3.061857	0.251786
H	-4.930117	-3.094801	-0.531836
H	-4.664154	-3.303141	1.202167
C	-3.047380	-4.069546	-0.023688
H	-3.505924	-5.064017	-0.097725
C	4.253384	-2.160847	1.118219
H	3.632455	-2.447135	1.965163
H	5.255885	-1.940919	1.499423
C	4.332501	-3.298446	0.089869
H	4.680232	-4.187057	0.631179
H	5.108307	-3.076633	-0.652248
H	-2.373377	-4.107306	0.840706
C	-2.852073	-1.328655	1.482828
H	-1.871200	-1.003957	1.146157
H	-2.713238	-2.249888	2.052920
C	4.369604	-0.263766	-0.526791
H	4.564131	-1.019561	-1.292111
H	3.696983	0.470193	-0.972240
C	3.016694	-3.615183	-0.632426
H	2.713379	-2.744806	-1.227909
H	3.210524	-4.415756	-1.356061
C	1.868269	-4.029873	0.292174
H	1.604679	-3.232443	0.992085
H	0.972836	-4.276349	-0.287856
H	2.139912	-4.915409	0.878193
C	5.672413	0.421419	-0.105022
H	5.453716	1.110315	0.718112
H	6.374615	-0.324657	0.286035
C	6.334685	1.189802	-1.256329

H	5.634512	1.947009	-1.632927
H	7.192174	1.740802	-0.853707
C	6.805133	0.300566	-2.411876
H	7.484028	-0.481284	-2.052866
H	5.968735	-0.192664	-2.917130
H	7.339915	0.887739	-3.164553
C	-2.225557	-3.778430	-1.285651
H	-1.767929	-2.785683	-1.192665
H	-1.395704	-4.494721	-1.326532
C	-3.028078	-3.850643	-2.587988
H	-3.806891	-3.083998	-2.618134
H	-2.377750	-3.700718	-3.455941
H	-3.508898	-4.829732	-2.697556
C	-3.479770	-0.271547	2.394951
H	-3.721703	0.624206	1.812640
H	-4.428777	-0.658387	2.784791
C	-2.554151	0.122670	3.555050
H	-2.301799	-0.771763	4.140363
H	-3.113712	0.779491	4.231331
C	-1.264923	0.831378	3.118894
H	-0.712312	1.197519	3.989959
H	-0.589592	0.172936	2.565298
H	-1.488195	1.693513	2.480875
C	0.399516	-0.565395	-0.659516
H	-0.156117	-1.213599	0.014502
H	-0.183573	-0.396693	-1.558413
H	1.336667	-1.047744	-0.933698
